# Supplementary material for: Non-coding RNAs in drug and radiation resistance of bone and soft-tissue sarcoma: a systematic review
Source: eLife. 2022 Nov 3;11:e79655. doi: 10.7554/eLife.79655 (PMC9691005; doi:10.7554/eLife.79655)
Supplement: Supplementary file 1. [file elife-79655-supp1.docx]

**Supplementary Table 1. Summary of non-coding RNAs in sarcoma therapeutic resistance.**

| First author  Year  [Reference] | Pathological type  (subtype) | Setting | Non-coding RNA investigated | Intervention | Methods | Number of replicates/  subjects | Genes and pathways | Major conclusion | W-MeQS |
| --- | --- | --- | --- | --- | --- | --- | --- | --- | --- |
| Chemotherapy (OS) | | | | | | | | | |
| Xie  2020  [1] | OS | in vitro | lncRNA NORAD | cisplatin | RT-qPCR | 3 | miR-410-3p/NF-κB | Inhibition of lncRNA NORAD expression can increase sensitivity to cisplatin. | 4 |
| Li  2019  [2] | OS | in vitro | lncRNA ANRIL | cisplatin | RT-qPCR | 3 | miR-125a-5p/STAT3 | ANRIL knockdown sensitizes OS cells to cisplatin. | 3 |
| Lee  2021  [3] | OS | in vitro | lncRNA ANRIL | cisplatin  doxorubicin | RT-qPCR | 3 | - | The over-expression of ANRIL in OS cells led to an increased resistance to both agents. | 5 |
| Wang  2016  [4] | OS | in vitro | LINC00161 | cisplatin | RT-qPCR  microarray | 3 | miR-645/IFIT2 | LINC00161 sensitizes OS cells to cisplatin. | 7 |
| Zhang  2021  [5] | OS | in vitro | lncRNA FOXD2-AS1 | cisplatin | RT-PCR | - | miR-143/ (Bax&Bcl-2) | LncRNA FOXD2-AS1 knockdown inhibits the resistance of human OS cells to cisplatin. | 3 |
| Li  2021  [6] | OS | in vitro | LINC01116 | doxorubicin | RT-qPCR | 3 | miR-424-5p/HMAG2 | LINC01116 promotes doxorubicin resistance in OS. | 5 |
| Cheng  2019  [7] | OS | in vitro | lncRNA NCK-AS1 | cisplatin | RT-qPCR | 3 | miR-137/ (Bax, Bcl-2 &cleaved caspase 3) | NCK-AS1 knockdown enhanced DDP sensitivity of OS cells. | 4 |
| Zhang  2017  [8] | OS | in vitro | LINC00473 | cisplatin | RT-qPCR | 3 | C/EBPβ-IL24 | Elevated ZBTB7A inhibits cisplatin-induced apoptosis by repressing LINC00473 expression in OS cells. | 7 |
| Sun  2022  [9] | OS | in vitro | lncRNA SNHG15 | cisplatin | RT-qPCR | 3 | miR-335-3p/ZNF32 | SNHG15 suppresses cisplatin-induced apoptosis and ROS accumulation through the miR-335-3p/ZNF32 pathway. | 7 |
| Wang  2021  [10] | OS | in vitro | lncRNA DICER1-AS1 | cisplatin  carboplatin  etoposide  methotrexate  doxorubicin | RT-qPCR | 3 | miR-34a-5p/GADD45A | DICER1-AS1 indeed involves in the inhibition of the multi-drugresistance of OS cells. | 9 |
| Song  2019  [11] | OS | in vitro  in vivo | lncRNA OIP5-AS1 | cisplatin | qPCR | 3 | miR-340-5p/ LPAATβ/PI3K/AKT/mTOR | Lnc RNA OIP5AS1 causes cisplatin resistance in OS. | 6 |
| Han  2018  [12] | OS | in vitro  in vivo | lncRNA LUCAT1 | methotrexate | RT-qPCR | 3 | miR-200c/ABCB1 | LUCAT1 knockdown decreased the expression levels drug resistance related genes. | 5 |
| Zhou  2019  [13] | OS | in vitro  in vivo | lncRNA TUG1 | cisplatin | RT-qPCR | - | MET/AKT | Knockdown of TUG1 inhibited the cisplatin resistance. | 4 |
| Hu  2019  [14] | OS | in vitro  in vivo | lncRNA TUG1 | doxorubicin | RT-qPCR | - | AKT | Down-regulating of lncRNA TUG1 promotes apoptosis of doxorubicin resistant OS cells. | 7 |
| Sun  2019  [15] | OS | in vitro  in vivo | lncRNA PVT1 | gemcitabine | RT-qPCR | 3 | miR-152/c-MET/PI3K/AKT | LncRNAPVT1 targets miR-152 to enhance chemoresistance of OS to gemcitabine. | 6 |
| Liu  2021  [16] | OS | in vitro  in vivo | lnc MALAT-1 | doxorubicin | RT-qPCR | 3 | ERK | Downregulating MALAT-1 in the doxorubicin resistance OS cells could reverse the resistance and may improve chemotherapeutic efficiency. | 7 |
| Pu  2021  [17] | OS | in vitro  in vivo | lncRNA LAMTOR5-AS1 | cisplatin | RT-qPCR | 3 | NRF2 | LAMTOR5-AS1 significantly inhibits the proliferation and drug resistance of OS cells. | 9 |
| Shen  2020  [18] | OS | in vitro  in vivo | lnc ARSR | adriamycin  paclitaxel  cisplatin | RT-qPCR  microarray | 3 | MRP1 | The reduction of lnc ARSR overcame the resistance to adriamycin. | 9 |
| Wang  2020  [19] | OS  (N/A) | in vitro  humans | LINC00426 | doxorubicin | RT-qPCR | 3/  OS tissues:50  normal tissues:50 | miR-4319/caspase 3 | LINC00426 contributes to doxorubicin resistance. | 4 |
| Zhu  2019  [20] | OS  (N/A) | in vitro  humans | lncRNA MEG3 | doxorubicin  cisplatin  methotrexate | RT-qPCR | 3/  chemo-sensitive OS tissue:48  chemo-resistance OS tissue:32 | hsa-miR-200b-3p/AKT2 | LncRNA MEG3 could promote the OS doxorubicin resistance through miR-200b-3p/AKT2 axis. | 9 |
|  |  |  | hsa_circ_0001258 |  |  |  | hsa-miR-744-3p/GSTM | Hsa_circ_0001258 suppressed the doxorubicin resistance of OS cells through hsa-miR-744-3p /GSTM2 axis. |  |
| Fu  2019  [21] | OS  (N/A) | in vitro  humans | lncRNA TTN-AS1 | cisplatin | qPCR | -/  OS tissues:55  normal tissues:9 | miR-134-5p/MBTD1 | Downregulation of lncRNA TTN-AS1 reduced drug resistance. | 8 |
| Zhang  2020  [22] | OS  (N/A) | in vitro  humans | lncRNA MSC-AS1 | cisplatin | RT-qPCR | 3/  OS tissues:45  normal tissues:45 | miR-142/PI3K/AKT | Slience MSC-AS1 made OS cells more sensitive to cisplatin. | 4 |
| Liu  2020  [23] | OS  (N/A) | in vitro  in vivo  humans | lncRNA OIP5-AS1 | cisplatin | RT-qPCR | 3/  cisplatin-resistant OS tissue: 30  cisplatin-sensitive OS tissue: 17 | miR-377-3p/FOSL2 | LncRNA OIP5-AS1 positively modulated FOSL2 expression to decrease cisplatin sensitivity in OS. | 6 |
| Gu  2020  [24] | OS  (N/A) | in vitro  in vivo  humans | LINC00922 | doxorubicin | RT-qPCR  microarray | -/  OS tissues:40  normal tissues:40 | miR-424-5p/TFAP2C | LINC00922 accelerated OS doxorubicin-resistance. | 4 |
| Sun  2020  [25] | OS  (N/A) | in vitro  in vivo  humans | lncRNA OIP5-AS1 | doxorubicin | RT-qPCR | 3/  OS tissues:56  normal tissues:16 | miR-137-3p/PTN | LncRNA OIP5-AS1 promotes resistance to doxorubicin by regulating miR-137-3p/PTN axis in OS. | 6 |
| Meng  2020  [26] | OS  (osteoblastic/ chondroblastic/ fibroblastic/ mixed OS) | in vitro  in vivo  humans | lncRNA MIR17HG | cisplatin | RT-qPCR | 3/  OS tissues:40  (15 osteoblastic, 10 chondroblastic, 9 fibroblastic and 6 mixed OS)  normal tissues:40 | miR-130a-3p/SP1 | MIR17HG promoted the  proliferation, invasion and cisplatin resistance of OS cells in vitro. | 5 |
| Liang  2018  [27] | OS  (osteoblastoma/other OS) | in vitro  in vivo  humans | lncRNA DNAJC3‐AS1 | cisplatin | RT-qPCR | 3/  tumor tissues: 30  (19 osteoblastoma and 11 other OS)  normal tissues: 30 | DNAJC3 | DNAJC3‐AS1 reduced sensitivity of OS to cisplatin. | 7 |
| Shi  2020  [28] | OS  (N/A) | in vitro  in vivo  humans | lncRNA PWRN1 | cisplatin | RT-qPCR | -/  OS tissues: 54  normal tissues: 54 | miR-214-5p | PWRN1 overexpression inhibited cisplatin chemoresistance in OS cells. | 4 |
| Zhu  2020  [29] | OS  (N/A) | in vitro  in vivo  humans | lncRNA Sox2OT-V7 | doxorubicin | RT-qPCR | 3/  OS tissues: 32  normal tissues: 32 | miR-142/miR-22 | Sox2OT-V7 promotes doxorubicin-induced chemoresistance in OS. | 8 |
| Wen  2020  [30] | OS  (N/A) | in vitro  in vivo  humans | lncRNA-SARCC | cisplatin | RT-qPCR | 3/  cisplatin-sensitive OS tissue:20  cisplatin-resistant OS tissue:20 | miR-143/Hexokinase 2 | LncRNA-SARCC sensitizes OS to cisplatin. | 4 |
| Wang  2017  [31] | OS  (N/A) | in vitro  in vivo  humans | lncRNA CTA | doxorubicin | RT-qPCR | 3/  OS tissues: 30  normal tissues: 30 | miR-210/ (p62, cleaved caspase 3& Bcl-2) | LncRNA CTA sensitizes OS cells to doxorubicin. | 6 |
| Guo  2020  [32] | OS  (N/A) | in vitro  humans | lncRNA HOTAIR | cisplatin | qPCR | 3/  sensitive cases:20 resistant cases:20 | miR-106a-5p/STAT3 | HOTAIR enhanced cisplatin resistance of OS. | 5 |
| Zhou  2018  [33] | OS  (N/A) | in vitro  humans | lncRNA SNHG12 | doxorubicin | RT-qPCR | 3/  sensitive cases:32 resistant cases:32 | miR-320a/MCL1 | Knockdown of SNHG12  improved the sensitivity of doxorubicin. | 5 |
| Zhang  2016  [34] | OS  (N/A) | in vitro  humans | lncRNA ODRUL | doxorubicin | RT-qPCR | -/  sensitive cases:30 resistant cases:30 | ABCB1 | LncRNA ODRUL contributes to doxorubicin resistance of OS. | 5 |
| Cheng  2019  [35] | OS  (N/A) | in vitro  humans | lncRNA ROR | cisplatin | RT-qPCR | 3/  primary OS tissues: 25  relapsed OS tissues: 25 | miR-153-3p/ABCB1 | LncRNA ROR induce cisplatin resistance in OS. | 3 |
| Zhang  2017  [36] | OS  (N/A) | in vitro  in vivo  humans | lncRNA FOXC2-AS1 | doxorubicin | RT-qPCR | -/  chemosensitive OS tissue: 34  chemoresistant OS group: 34 | FOXC2/ABCB1 | LncRNA FOXC2-AS1 promotes doxorubicin resistance in OS | 6 |
| Zhu  2019  [37] | OS  (N/A) | in vitro  in vivo  humans | lncRNA OIP5‐AS1 | doxorubicin | RT-qPCR | -/  chemo‐resistant:32  chemo‐sensitive:48 | miR-200b-3p/Fibronectin‐1 | Lnc RNA OIP5‐AS1 modulates Fibronectin‐1 contributes to doxorubicin resistance of OS cells. | 8 |
| Li  2018  [38] | OS  (N/A) | in vitro  humans | lncRNA B4GALT1-AS1 | adriamycin | RT-qPCR | 3/  tumor sample:39  normal tissue:39 | YAP | Knockdown of B4GALT1-AS1 inhibited OS cells chemotherapeutic sensitivity. | 7 |
| Hu  2018  [39] | OS  (N/A) | in vitro  in vivo  humans | lncRNA NEAT1 | cisplatin | RT–qPCR | -/  OS tissue:40  normal tissue:20 | miR-34c/ (Bcl-2, CCND1) | Knockdown of lncRNA NEAT1 improves the sensitivity to cisplatin in OS. | 6 |
| Li  2016  [40] | OS  (N/A) | in vitro  humans | lnc HOTTIP | cisplatin | RT-qPCR | 3/  OS tissue:21  normal tissue:21 | Wnt/β-catenin | Overexpression of lnc HOTTIP increases chemoresistance of OS. | 3 |
| Chen  2018  [41] | OS  (osteoblastoma/other OS) | in vitro  in vivo  humans | lnc RAB11B-AS1 | cisplatin | RT-qPCR | 3/  OS tissue: 24  (14 osteoblastoma and 10 other OS)  normal tissue:24 | RAB11B | The reduction of RAB11B-AS1 results in lower sensitivity to cisplatin in OS cells. | 7 |
| Zhu  2017  [42] | OS  (N/A) | in vitro  in vivo  humans | lncRNA FENDRR | doxorubicin | RT-qPCR | 3/  chemoresistant group:40  chemosensitive group:40 | ABCB1  ABCC1 | LncRNA FENDRR sensitizes doxorubicin-resistance of OS cells. | 5 |
| Zhang  2020  [43] | OS  (N/A) | in vitro  in vivo  humans | lncRNA SNHG15 | doxorubicin | RT-qPCR | 3/  chemoresistant group:30  chemosensitive group:30 | miR-381-3p/GFRA1 | LncRNA SNHG15 contributes to doxorubicin resistance of OS cells. | 4 |
| Tang  2022  [44] | OS  (N/A) | in vitro  in vivo  humans | LINC00641 | cisplatin | RT-qPCR | OS tissues and paracancerous tissues:58 pairs | miR-320d/MCL1 | Knock-down of LINC00641 gene represses DDP-resistance of DDP-resistant OS cells via modulating miR-320d. | 5 |
| Liu  2020  [45] | OS | in vitro | miR-187 | doxorubicin | RT-qPCR | 3 | MAPK7 | MiR-187 enhanced the chemosensitivity of the OS cells to doxorubicin | 3 |
| Patil  2019  [46] | OS | in vitro | miR-509-3p | cisplatin | RT-qPCR | 3 | AXL  ARHGAP1 | MicroRNA-509-3p sensitizes OS to cisplatin. | 8 |
| Wang  2020  [47] | OS | in vitro | miR-410-3p | cisplatin | RT-qPCR | 3 | HMGB1/NF-κB | Overexpression of miR-410-3p increased cisplatin sensitivity of OS cells via suppressing HMGB1/NF-κB pathway. | 4 |
| Lou  2019  [48] | OS | in vitro | miR-29b | doxorubicin | RT-qPCR | 3 | MMP-9 | MiR-29b sensitizes OS cells to doxorubicin. | 3 |
| Li  2020  [49] | OS | in vitro | miR-29b | doxorubicin | RT-qPCR | 3 | spin1/PI3K/AKT  spin1/STAT3 | Down-regulating of miR-29b promotes OS cell drug resistance. | 8 |
| Vanas  2016  [50] | OS | in vitro | miR-21 | cisplatin | northern blot | 3 | Spry2 | MiRNA-21 increases cisplatin sensitivity of OS-derived cells | 5 |
| Ziyan  2014  [51] | OS | in vitro | miR-21 | cisplatin | RT-qPCR | 3 | Bcl-2 | Suppression of miR-21 in OS cells led to enhanced cisplatin cytotoxicity. | 3 |
| Bazavar  2020  [52] | OS | in vitro | miR-192 | methotrexate | RT-qPCR | 4 | MMP-9  c-Myc  KRAS  CXCR-4  ADAMTS | MiR-192 enhances sensitivity of methotrexate to OS cells. | 3 |
| Xu  2016  [53] | OS | in vitro | miR-30a | doxorubicin | RT-qPCR | 3 | Beclin-1 | MicroRNA-30a downregulation contributes to chemoresistance of OS cell. | 4 |
| Wang  2019  [54] | OS | in vitro | miR-22 | cisplatin | RT-qPCR | 3 | MTDH | MiR-22 sensitized OS cells to cisplatin treatment. | 4 |
| Li  2014  [55] | OS | in vitro | miR-22 | doxorubicin  cisplatin | RT-qPCR | 3 | HMGB1 | Overexpressed miR-22 against chemotherapy resistance in OS. | 4 |
| Zhu  2020  [56] | OS | in vitro | miR-4779 | doxorubicin | - | - | PLK1 | MiR-4779 negatively regulates  the expression of PLK1 and possesses a cancer-inhibiting role in drug-resistant OS cells. | 3 |
| Zhang  2015  [57] | OS | in vitro | miR-217 | cisplatin | RT-qPCR | 3 | KRAS | Overexpression of miR-217 enhanced cisplatin sensitivity of 143B OS cells. | 6 |
| Song  2017  [58] | OS | in vitro | miR-340-5p | cisplatin | RT-qPCR | 2 | LPAATβ | MiR-340-5p enhanced the sensitivity to CDDP. | 3 |
| Song  2017  [59] | OS | in vitro | miR-214 | cisplatin | RT-qPCR | 3 | HK2  PKM2  LDHA | MiR-214 contributes to cisplatin resistance in OS cells. | 3 |
| Fiore  2016  [60] | OS | in vitro | Let-7d microRNA | doxorubicin  cisplatin  paclitaxel  etoposide | RT-qPCR | 4 | Bcl-2  caspase3  E2F2  CCND2 | Let-7d- overexpression reduced cell sensitivity to apoptosis induced by various chemotherapy drugs. | 6 |
| Zhang  2015  [61] | OS | in vitro | miR-301a | doxorubicin | RT-qPCR  northern blot | 3 | AMPKα1 | Up-regulation of miR-301a contributed to chemoresistance of OS cells. | 6 |
| Meng  2020  [62] | OS | in vitro  in vivo | miR-22 | cisplatin | RT-qPCR | 3 | MTDH | MiR-22 promoted cisplatin sensitivity. | 4 |
| Meng  2020  [63] | OS | in vitro  in vivo | miR‑22 | cisplatin | RT‑qPCR | 3 | PI3K/AKT/mTOR | MiR‑22 leads to an improvement in the sensitivity of cisplatin in OS. | 7 |
| Yuan  2018  [64] | OS | in vitro  in vivo | miR-20a | doxorubicin  cisplatin | RT-qPCR | 3 | TAK1 | Overexpression of miR-20a sensitizes the OS cells to chemotherapeutic drugs. | 5 |
| Lin  2016  [65] | OS | in vitro  in vivo | miR-184 | doxorubicin | RT-qPCR | 3 | BCL2L1 | MiR-184 leads to poor response to doxorubicin therapy. | 4 |
| Lei  2018  [66] | OS | in vitro  in vivo | miR-199a-3p | cisplatin  carboplatin  doxorubicin | RT-qPCR | 3 | AK4/NF-кB | MiR-199a-3p promoted multi-drug resistance in OS cells. | 6 |
| Pu  2016  [67] | OS | in vitro  in vivo | miR-34a-5p | doxorubicin  etoposide  methotrexate  cisplatin  carboplatin | RT-qPCR | 3 | MEF2 | MiR-34a-5p promotes multi-drug resistance in OS cells. | 7 |
| Song  2010  [68] | OS | in vitro | miR-215 | methotrexate | RT–qPCR | 2 | DTL | MiR-215 increase in chemoresistance to methotrexate in OS cells. | 7 |
| Li  2017  [69] | OS | in vitro, | miR-34a | cisplatin | RT–qPCR | 3 | c-Myc  Bim | MiR-34a increases cisplatin sensitivity of OS cells in vitro. | 4 |
| Zhou  2016  [70] | OS | in vitro | miR-34b | doxorubicin  gemcitabine  methotrexate | RT-qPCR | 3 | ABCB1  PAK1 | miR-34b overexpression reverses multidrug resistance in human OS cells in vitro. | 6 |
| Chang  2014  [71] | OS | in vitro | miR-101 | doxorubicin | - | 3 | LC3-Ⅰ  LC3-Ⅱ | Blocked autophagy by miR-101 enhances OS cell chemosensitivity. | 3 |
| Gao  2015  [72] | OS | in vitro | miR-199a-3p | doxorubicin | microarray | 3 | CD44 | MiR-199a-3p transfection increased drug sensitivity in OS. | 7 |
| Fiore  2014  [73] | OS | in vitro | miR‐29b-1 | cisplatin  doxorubicin  etoposide | RT-qPCR | 3 | Oct3/4  SOX2  Nanog  CD133  N-Myc | MiR-29b-1 overexpression sensitized OS cells to chemotherapeutic drug-induced apoptosis. | 4 |
| Wei  2016  [74] | OS | in vitro | miR-140-5p | cisplatin  doxorubicin | RT-qPCR | 3 | IP3K2 | The increased miR-140-5p expression levels up-regulated anticancer drug-induced autophagy in OS cells. | 3 |
| Xie  2018  [75] | OS | in vitro | miR-149 | doxorubicin | RT-qPCR | 3/  tumor tissue:41  normal tissue:36 | BMP9 | Overexpression of miR-149 conferred chemoresistance in OS cells. | 4 |
| Novello  2014  [76] | OS | in vitro | miR-34a | etoposide | RT-qPCR | 3 | - | MiR-34a basal levels were lower in p53-deficient OS cells and with a higher sensitivity to etoposide. | 5 |
| Chen  2014  [77] | OS | in vitro | miR-155 | cisplatin | qPCR | 3 | LC3-Ⅰ  LC3-Ⅱ | The increased miR-155 expression levels upregulated anti­cancer drug-induced autophagy in OS cells. | 3 |
| Li  2015  [78] | OS | in vitro | miR-199a-5p | cisplatin | RT-qPCR | 3 | LC3-Ⅰ  LC3-Ⅱ | MicroRNA-199a-5p inhibits cisplatin-induced drug resistance in OS cells. | 3 |
| Huang  2021  [79] | OS | in vitro | miR-203 | cisplatin | RT-qPCR | 3 | RUNX2 | Knockdown of microRNA-203 reduces cisplatin chemosensitivity to OS cell lines. | 4 |
| Zou  2018  [80] | OS | in vitro | miR-133b | cisplatin | RT-qPCR | 3 | - | MiR-133b induces chemoresistance of OS cells to cisplatin treatment. | 3 |
| Zhu  2021  [81] | OS | in vitro | miR-128-3p | cisplatin | RT-qPCR | 3 | ZC3H12D | MiR-128-3p overexpression also improved resistance to cisplatin in OS cells. | 4 |
| Zhang  2019  [82] | OS | in vitro | miR-19a-3p | cisplatin | RT-qPCR | 3 | PTEN | Silencing of miR‑19a‑3p enhances OS cells chemosensitivity. | 5 |
| Jiang  2015  [83] | OS | in vitro | miR-126 | cisplatin  methotrexate | qPCR | - | - | MicroRNA-126 enhances the sensitivity of OS cells to cisplatin and methotrexate. | 5 |
| Chen  2016  [84] | OS | in vitro | miR-34a  miR-203 | cisplatin | RT-qPCR | 3 | survivin | MiR-34a and miR-203 enhanced cell sensitivity to cisplatin. | 7 |
| Yu  2019  [85] | OS | in vitro | miR-26a-5p | paclitaxel | RT-qPCR | 3 | HOXA5 | Knock-down of miR-26a-5p increased OS cell sensitivity to chemotherapeutic drug paclitaxel. | 3 |
| Pu  2017  [86] | OS | in vitro  in vivo | miR-34a-5p | doxorubicin  etoposide  methotrexate  cisplatin | RT-qPCR | 3 | DLL1 | MiR-34a-5p promotes multi-chemoresistance of OS. | 4 |
| Song  2009  [87] | OS | in vitro  in vivo | miR-140 | methotrexate | RT–qPCR | 2 | HDAC4 | Overexpression of miR-140 causes chemoresistance to methotrexate in OS. | 7 |
| Pu  2017  [88] | OS | in vitro  in vivo | miR-34a-5p | doxorubicin cisplatin  carboplatin  etoposide | RT-qPCR | 3 | AGTR1 | The miR-34a-5p promotes the multi-chemoresistance of OS. | 6 |
| Osaki  2016  [89] | OS | in vitro  in vivo | miR‐29 | cisplatin  doxorubicin; | RT-qPCR | 3 | MCL1 | Upregulation of miR-29 enhanced chemotherapy- induced apoptosis in OS cells. | 6 |
| Zhang  2021  [90] | OS | in vitro  in vivo | miR-134 | doxorubicin | RT-qPCR | 3/  resistant group:23  sensitive group:23 | PTBP1 | Downregulation of miR-134 promoted chemoresistance of OS cells to doxorubicin by upregulating PTBP1 expression. | 6 |
| Zhao  2017  [91] | OS | in vitro  in vivo | miR‑20a‑5p | etoposide  methotrexate  cisplatin  doxorubicin | RT-qPCR | 3 | SDC2 | MiR‑20a‑5p represses the multi‑drug resistance of OS. | 5 |
| Wang  2019  [92] | OS | in vitro  in vivo | miR-193a | doxorubicin | RT-qPCR | 3 | IRS2 | MiR-193a-3p suppresses both growth and doxorubicin drug resistance of OS in vivo. | 4 |
| Gao  2017  [93] | OS | in vitro  in vivo | miR-335 | cisplatin | RT-qPCR | 3 | POU5F1 | Pre-miR-335 resulted in tumor enhanced sensitivity to traditional chemotherapy. | 6 |
| Cheng  2020  [94] | OS  (N/A) | in vitro  in vivo  humans | miR‑487b‑3p | doxorubicin | RT-qPCR | -/  patients:40  healthy bone:24 | ALDH1A3 | MiR‑487b‑3p inhibits OS chemoresistance. | 6 |
| Yu  2019  [95] | OS  (N/A) | in vitro  humans | miR-221 | cisplatin | RT-qPCR | 3/  OS tissues:15  normal tissues:15 | PPP2R2A | Overexpression of miR-221 promoted OS cell cisplatin resistance. | 5 |
| Tsai  2018  [96] | OS  (N/A) | in vitro  in vivo  humans | miR‐519d | doxorubicin | RT-qPCR | - | ABCG2 | Downregulation of miR‐519d increases ABCG2 expression and promotes drug resistance. | 6 |
| Meng  2017  [97] | OS  (N/A) | in vitro  in vivo  humans | miR-140-5p | cisplatin  doxorubicin  methotrexate | RT-qPCR  microarray | 3/  OS tissues: 40  normal tissues: 40 | HMGN5 | Knockdown of miR-140-5p enhanced OS cells resistance to multiple chemotherapeutics. | 8 |
| Zhang  2020  [98] | OS  (N/A) | in vitro  humans | miR-129-5p | 5-flurouracil | RT-qPCR | -/  OS tissues: 30  normal tissues: 30 | LARP1 | Down-regulation of miR-129-5p promoted proliferation, invasion, and drug resistance in OS. | 3 |
| Liu  2019  [99] | OS  (N/A) | in vitro  humans | miR-16 | cisplatin | RT-qPCR | 3/  OS tissues: 30  normal tissues: 30 | ATG4B | Down-regulation of miR-16 enhances cisplatin resistance of OS. | 5 |
| Keremu  2019  [100] | OS  (N/A) | in vitro  in vivo  humans | miR-199a | cisplatin | RT-qPCR | 3/  OS tissues: 20  normal tissues: 20 | HIF-1α | Overexpression ofmiR-199a resensitizes cisplatin resistant cells to cisplatin. | 5 |
| Yan  2018  [101] | OS  (N/A) | in vitro  humans | miR-340 | cisplatin | RT-qPCR | -/  tumor tissues: 20  normal tissues: 20 | ZEB1 | Overexpression of miR-340 enhanced sensitivity to DDP in OS cells. | 4 |
| Xiao  2017  [102] | OS  (N/A) | in vitro  humans | miR-100 | doxorubicin | RT-qPCR | 5/  tumor tissues: 28  normal tissues: 28 | ZNRF2 | MiR-100 suppresses OS cell chemoresistance | 3 |
| Wang  2017  [103] | OS  (osteoblastic/ chondroblastic/ fibroblastic/ telangiectatic/ other OS) | in vitro  in vivo  humans | miR-491 | cisplatin | RT-qPCR | -/  OS patients:102  (60 osteoblastic, 13 chondroblastic, 18 fibroblastic, 6 telangiectatic and 5 other OS)  healthy control:20 | CRYAB | MiR-491 inhibits OS lung metastasis and chemoresistance | 8 |
| Zhou  2018  [104] | OS  (parosteal/ conventional/ chondroblastic/osteoblastoma/telangiectatic OS) | in vitro  humans | miR-22 | cisplatin | RT-qPCR | 3/  OS patients:7  (1 parosteal, 3 conventional,1 chondroblastic, 1 osteoblastoma and 1 telangiectatic OS)  healthy control:7 | S100A11 | MiR-22 increase the cisplatin sensitivity of OS cells. | 4 |
| Zhang  2020  [105] | OS  (N/A) | in vitro  in vivo  humans | miR-429 | adriamycin | qPCR | 2/  OS tissues:10  normal tissues:10 | SOX2 | Upregulation of miR-429 increased adriamycin sensitivity through down regulating SOX2 in CD133+ OSCs. | 3 |
| Xu  2014  [106] | OS  (N/A) | in vitro  humans | miR-34c | cisplatin  doxorubicin  methotrexate | RT-qPCR | 3/  metastasis:25  chemosensitive:76  chemoresistance:21 | Notch1  LEF1 | MiR-34c inhibits OS chemoresistance. | 5 |
| Duan  2016  [107] | OS  (N/A) | in vitro  in vivo  humans | miR-15b | doxorubicin | RT-qPCR  microarray | 4/  samples from survivors; 14  samples from nonsurvivors:35 | Wee1 | MDR in OS is associated with downregulation of miR-15b. | 9 |
| Maximov  2019  [108] | OS  (osteoblatic/osteoblatic/chondroblastic/high-grade surface OS) | in vitro  in vivo  humans | miR-16-1-3p  miR-16-2-3p | cisplatin  doxorubicin | RT-qPCR | 3/  OS samples:18  (15 osteoblatic, 2 osteoblatic/chondroblastic and 1 high-grade surface OS ) | FGFR2 | MiR-16-1-3p and miR-16-2-3p overexpression enhances chemosensitivity. | 8 |
| Chen  2017  [109] | OS  (N/A) | in vitro  humans | miR-410 | cisplatin | RT-qPCR | 3/  OS sample:40  normal tissue:40 | ATG16L1 | MiR-410 enhances chemosensitivity in OS. | 5 |
| Lin  2015  [110] | OS  (osteoblastic/ fibroblastic OS) | in vitro  humans | miR‑202 | doxorubicin | RT-qPCR | 3/  OS sample:8  (6 osteoblastic and 2 fibroblastic OS)  normal tissue:8 | TGF-β 1 | MiR-202 promotes chemotherapy resistance in OS cells. | 6 |
| Zhao  2013  [111] | OS  (N/A) | in vitro  humans | miR-221 | cisplatin | RT-qPCR | 3/  OS tissue:60  normal tissue:25 | PI3K/AKT | MicroRNA-221 Induces cisplatin resistance in human OS. | 5 |
| Zhou  2016  [112] | OS  (N/A) | in vitro  humans | miR-488 | doxorubicin | RT-qPCR | 3/  OS tissue:5  normal tissue:5 | Bim | Over-expression of miR-488 decreases the sensitivity to doxorubicin of OS cells. | 6 |
| Wang  2021  [113] | OS  (N/A) | in vitro  humans | miR-376c | cisplatin | RT-qPCR | 3/  tumor tissue:26  normal tissue:26 | TGFA | Cisplatin inhibited the prolif­eration of OS cells by upregulating miR-376c and downregulating *TGFA* expression. | 3 |
| Yang  2020  [114] | OS  (N/A) | in vitro  in vivo  humans | miR-216b | cisplatin | RT-qPCR | 3/  OS tissue:60  normal tissue:60 | JMJD2C//HIF-1α/HES1 | MiR-216b enhances cisplatin-induced apoptosis in OS cells | 9 |
| Xu  2018  [115] | OS  (N/A) | in vitro  humans | miR-29 | methotrexate | RT-qPCR | 3/  poor-response:18  good-response:18 | COL3A1  MCL1 | MiR-29 inhibits resistance to methotrexate in OS. | 6 |
| Zhou  2015  [116] | OS  (N/A) | in vitro  in vivo  humans | miR-143 | doxorubicin | RT–qPCR | -/  OS patients:45  health controls:13 | ATG2B  LC3-I  Bcl-2 | MiR-143 expression significantly reversed chemoresistance in OS resistance cells. | 6 |
| Liu  2015  [117] | OS  (N/A) | in vitro  humans | miR-100 | cisplatin | RT–qPCR | 3/  OS sample:20  normal sample:20 | IGFIR/PI3K/AKT  IGFIR/MAPK/ERK | MiR-100 enhances chemosensitivity in OS cells. | 4 |
| Shao  2015  [118] | OS  (N/A) | in vitro  in vivo  humans | miR-497 | cisplatin | RT–qPCR | 3/  OS tissue:14  normal tissue:14 | PI3K/AKT | The down regulation of miR-497 contributes to cisplatin resistance in OS. | 5 |
| Xu  2016  [119] | OS  (N/A) | in vitro  humans | miR-146b-5p | doxorubicin  cisplatin  methotrexate | RT-qPCR | 3/  OS tissues:35  normal tissue;35 | zinc and ring finger 3 | MiR-146b-5p promotes invasion and metastasis contributing to chemoresistance in OS. | 5 |
| Zhou  2014  [120] | OS  (N/A) | in vitro  humans | miR-33a | cisplatin | RT-qPCR | 2/  poor responder:35  good responder:35; | TWIST | MiR-33a promotes OS cell resistance to cisplatin. | 7 |
| Liu  2019  [121] | OS  (N/A) | in vitro  humans | miR-342-5p | doxorubicin | RT-qPCR | 3/  OS tissues:6  normal tissues:6 | Wnt/β-catenin | MiR-342-5p inhibits OS cell growth, migration, invasion, and sensitivity to doxorubicin. | 6 |
| Liu  2018  [122] | OS  (N/A) | in vitro  in vivo  humans | miR-92a | cisplatin | RT-qPCR | 3/  tumor tissue;25  nontumor tissue:25 | Notch1 | MiR-92a inhibits the progress of OS cells and increases the cisplatin sensitivity. | 4 |
| Li  2021  [123] | OS  (N/A) | in vitro  humans | miR-329-3p | cisplatin | RT-qPCR | 3/  tumor tissue: 30  normal tissue:30 | LDHA | Overexpression of miR-329-3p sensitizes OS cells to cisplatin. | 4 |
| Tang  2018  [124] | OS  (N/A) | in vitro  humans | miR-223 | cisplatin | RT-qPCR | 3/  OS tissue: 20  normal tissue:20 | JNK/JUN | MiR-223 overexpression further promoted CDDP-induced OS cell apoptosis. | 5 |
| Xu  2014  [125] | OS  (osteoblastic/ chondroblastic/ fibroblastic/ telangiectatic/other OS) | in vitro  in vivo  humans | miR-382 | cisplatin  doxorubicin  methotrexate | RT-qPCR | 3/  OS tissues:115  (29 osteoblastic, 6 chondroblastic, 11 fibroblastic, 3 telangiectatic and 2 other OS)  normal bone:107 | KLF12  HIPK3 | MiR-382 inhibits tumor growth and enhance chemosensitivity in OS. | 4 |
| Liu  2018  [126] | OS  (N/A) | in vitro  humans | miR-377 | cisplatin | RT-qPCR | 3/  poor responder:21  good responder:21 | XIAP | Down-regulation of miR-377 contributes to cisplatin resistance in OS. | 4 |
| Liu  2017  [127] | OS  (N/A) | in vitro  in vivo  humans | miR-200c | cisplatin | RT-qPCR | 3/  OS tissue:35  normal tissue:35 | AKT2 | Overexpression of miR-200c increases chemosensitivity of OS cells to cisplatin. | 7 |
| Zhu  2016  [128] | OS  (N/A) | in vitro  humans | miR-138 | cisplatin | RT-qPCR | 3/  OS tissue:20  normal tissue:20 | EZH2 | MiR-138 enhances cisplatin-induced apoptosis in OS cells. | 7 |
| Long  2018  [129] | OS  (N/A) | in vitro  humans | miR‐590 | doxorubicin | qPCR | 3/  OS tissue:18  normal tissue:18 | WIP1/ATM-p53 | MiR‐590 overexpression could enhance the cytotoxicity of doxorubicin. | 5 |
| Li  2016  [130] | OS  (N/A) | in vitro  humans | miR-381 | cisplatin | RT-qPCR | 3/  OS patients: 60  chondroma patients:7 | mTOR | Low expression of miR-381 enhances the chemosensitivity of OS. | 6 |
| Wang  2016  [131] | OS  (N/A) | in vitro  humans | miR-367 | adriamycin | RT-qPCR | 3/  tumor tissue:40  normal tissue:40 | KLF4 | MiR-367 negatively regulates apoptosis induced by adriamycin in OS cells. | 4 |
| Li  2021  [132] | OS  (N/A) | in vitro  in vivo  humans | miR-26a | doxorubicin  methotrexate  cisplatin | RT-qPCR | 5/  chemoresistant group:12  chemosensitive group:9; | MCL1 | MiR-26a reverses resistance  to doxorubicin in OS multidrug resistance cells. | 7 |
| Jin  2017  [133] | OS  (conventional/ non-conventional OS) | in vitro  humans | miR-610 | cisplatin | RT-qPCR | -/  tumor tissue:21  (13 conventional and 17 non-conventional OS)  normal tissue:21; | TWIST1 | Overexpression of miR-610 increased sensitivity of OS cells to cisplatin. | 4 |
| Li  2020  [134] | OS  (osteoblastic/ fibroblastic/ chondroblastic/telangiectatic OS) | in vitro  humans | miR-584 | cisplatin | RT-qPCR | 3/  OS tissue:37  (19 osteoblastic, 8 fibroblastic, 6 chondroblastic and 4 telangiectatic OS)  normal tissue:37; | CCN2/IκBα/NF-κB | MicroRNA-584 sensitizes OS cells to cisplatin. | 8 |
| Chen  2019  [135] | OS  (N/A) | in vitro  humans | miR-504 | cisplatin | RT-qPCR | 3/  OS tissue:10  normal tissue:10; | p53 | MiR-504 contributes to cisplatin resistance in OS cells. | 4 |
| Zhou  2018  [136] | OS  (osteoblastic/ chondroblastic fibroblastic OS) | in vitro  humans | miR‑192‑5p | cisplatin | RT-qPCR | 3/  OS tissue:25  (13 osteoblastic, 9 chondroblastic, and 3 fibroblastic OS)  normal tissue:25; | USP1 | Ectopic expression of miR-192-5p increased the sensitivity of osteosarcoma cells to cisplatin. | 4 |
| Ling  2020  [137] | OS  (N/A) | in vitro  humans | miR-150 | doxorubicin | RT-qPCR | 3/  OS tissue: 26  normal tissue:26; | RUNX2 | MicroRNA-150 functions as a tumor suppressor and sensitizes OS to doxorubicin-induced apoptosis. | 4 |
| Sun  2016  [138] | OS  (N/A) | in vitro  humans | miR-24 | doxorubicin | RT-qPCR | 3/  OS tissue: 45  normal tissue:45; | BIM-Smac/DIABLO | Knockdown of miR-24 reverses the doxorubicin-resistance in OS cells. | 5 |
| Zhi  2022  [139] | OS  (N/A) | in vitro  humans | miR-140 | doxorubicin | RT-qPCR | 3/  OS tissues:50  normal tissues:50 | Wnt1 | Overexpression of miR-140 inhibits OS cell proliferation and enhances drug sensitivity by suppressing Wnt1. | 5 |
| Zhou  2021  [140] | OS  (N/A) | in vitro  humans | miR-141-3p | cisplatin | RT-qPCR | 3/  OS and adjacent normal tissues:31 pairs | glutaminase | MiR-141-3p promotes the cisplatin sensitivity of OS cell through suppressing the glutaminase-mediated glutamine metabolism. | 4 |
| Liang  2019  [141] | OS  (N/A) | in vitro  in vivo  humans | miR-765 | cisplatin | RT-qPCR | 3/  negative: 19  positive: 24 | APE1 | MicroRNA-765 sensitizes OS cells to cisplatin. | 6 |
| Gao  2020  [142] | OS  (N/A) | in vitro  in vivo  humans | miR-375 | cisplatin | RT-qPCR | 3/  chemosensitive group: 35  chemoresistant group: 35 | ATG2B | MiR-375 suppresses autophagy and tumorigenesis in cisplatin-resistant OS cells | 5 |
| Wang  2021  [143] | OS  (N/A) | in vitro  in vivo  humans | miR‐519d‐3p | cisplatin | RT-qPCR | OS and adjacent normal tissues:40 pairs | PD‐L1 | MiR‐519d‐3p antagonizes OS resistance  against cisplatin by targeting PD‐L1. | 8 |
| Wang  2022  [144] | OS  (N/A) | in vitro  in vivo  humans | miR-19a-3p | cisplatin | RT-PCR | OS tissues:85 | PHLDA3/AKT/GSK3β | MiR-19a-3p promotes tumor growth and chemoresistance in OS by downregulating PHLDA3. | 5 |
| Zhan  2022  [145] | OS | in vitro  in vivo | miR-579-3p | cisplatin | RT-qPCR | 3 | MSH6 | Increasing expression  of miR-579 could inhibit the development and cisplatin resistance of OS. | 7 |
| Zhang  2021  [146] | OS | in vitro | circ-CHI3L1.2 | cisplatin | RT-qPCR | 3 | miR-340-5p/LPAATβ | Circ-CHI3L1.2 knockdown sensitized cisplatin-resistant OS cells to cisplatin. | 7 |
| Zhang  2020  [147] | OS | in vitro  in vivo | circTADA2A | cisplatin | RT-qPCR | 3 | miR-129-5p/(TRPS1, YAP1) | CircTADA2A knockdown inhibited cell proliferation and reduced cisplatin resistance in OS cells. | 7 |
| Feng  2021  [148] | OS | in vitro  in vivo | circPRKAR1B | cisplatin | RT-qPCR | 3 | miR-361-3p/FZD4/ Wnt/β-catenin | Overexpression of circPRKAR1B suppresses the sensitivity of OS cells to cisplatin. | 8 |
| Dong  2020  [149] | OS  (N/A) | in vitro  humans | circUBAP2 | cisplatin | RT-qPCR | -/  cisplatin-response:30  cisplatin-resistance:30 | miR-506-3p/SEMA6D/Wnt/β-catenin | CircUBAP2 promotes SEMA6D expression to enhance the cisplatin resistance in OS. | 6 |
| Wei  2021  [150] | OS  (N/A) | in vitro  in vivo  humans | circ_0081001 | methotrexate | RT-qPCR | 3/  sensitive group:35  resistant group:28 | miR-494-3p/TGM2 | Circ_0081001 knockdown enhances methotrexate sensitivity in OS cells. | 4 |
| Hu  2019  [151] | OS  (chondroblastic/osteoblastic/ conventional/ telangiectatic OS) | in vitro  humans | circ‐LARP4 | cisplatin  doxorubicin | RT-qPCR | -/  OS tissue:72  (10 chondroblastic, 47 osteoblastic,9 conventional and 6 telangiectatic OS)  normal tissue: 72 | miR-424 | Circ‐LARP4 elevates chemosensitivity to cisplatin and doxorubicin. | 5 |
| Li  2020  [152] | OS  (N/A) | in vitro  in vivo  humans | hsa_circ_0000073 | methotrexate | RT-qPCR | -/  tumor tissues: 25  normal tissues: 25; | (miR-145-5P, miR-151-3p)/NRAS | Hsa_circ_0000073 contributes to OS methotrexate resistance. | 7 |
| Li  2021  [153] | OS  (N/A) | in vitro  in vivo  humans | circPTV1 | doxorubicin | RT-qPCR | 3/  chemoresistant group:21  chemosensitive group:31 | miR-137/TRIAP1 | CircPVT1 contributes to doxorubicin resistance of OS cells. | 5 |
| Zhu  2018  [154] | OS  (N/A) | in vitro  humans | circPVT1 | doxorubicin  cisplatin | RT–qPCR | 3/  OS patients: 80  benign bone tumor:20  healthy control:20 | ABCB1 | Overexpressed circPVT1 contributes to doxorubicin and cisplatin resistance of OS cells. | 7 |
| Wang  2022  [155] | OS  (N/A) | in vitro  humans | circ PVT1 | cisplatin  doxorubicin  methotrexate | RT-qPCR | OS and adjacent normal tissues:80 pairs | miR‑24‑3p/KLF8 | CircRNA PVT1 promotes proliferation and chemoresistance of OS cells via the miR‑24‑3p/KLF8 axis. | 4 |
| Pan  2021  [156] | OS  (N/A) | in vitro  humans | hsa_circ_103801 | cisplatin | RT–qPCR | 3/  OS patients: 43  healthy control:15 | MRP1  P-gp | Hsa_circ_103801 increased the resistance of OS cells to cisplatin. | 5 |
| Zhang  2018  [157] | OS  (N/A) | in vitro  humans | circ_001569 | cisplatin | RT-qPCR | 3/  tumor tissues:36  normal tissues:36 | Wnt/β-catenin | Expression of circ_001569 is upregulated in OS promotes cisplatin resistance. | 4 |
| Xie  2020  [158] | OS  (N/A) | in vitro  humans | hsa_circ_0003496 | doxorubicin | RT-qPCR | -/  primary OS patients: 35  recurrent OS patients:35; | miR-370/KLF12 | Hsa_circ_0003496 contributes to chemoresistance in OS. | 8 |
| Lin  2022  [159] | OS  (N/A) | in vitro  humans | has_circ_0001982 | paclitaxel  cisplatin  doxorubicin  methotrexate | RT-qPCR | 3/  OS tissue:20  drug sensitive:10  drug resistant:10 | miR-143 | Hsa_circ_0001982 improves multidrug resistance of OS cells. | 3 |
| Ma  2021  [160] | OS  (N/A) | in vitro  in vivo  humans | circRNA_0004674 | doxorubicin | RT-qPCR | 3/  OS tissues and paracancerous tissues:80 pairs | miR-142-5p/MCL1 | circRNA_0004674 facilitates OS progression and chemoresistance. | 7 |
| Yuan  2021  [161] | OS  (N/A) | in vitro  in vivo  humans | circPRDM2 | doxorubicin | RT-qPCR | -/  OS tissue:43  normal tissue:43 | miR-760/EZH2 | CircPRDM2 contributes to doxorubicin resistance of OS. | 8 |
| Wei  2020  [162] | OS  (N/A) | in vitro  in vivo  humans | circSAMD4A | doxorubicin | RT-qPCR | 3/  chemoresistant group:36  chemosensitive group:24; | miR-218-5p/KLF8 | CircSAMD4A contributes to cell doxorubicin  resistance in OS. | 4 |
| Zhou  2021  [163] | OS  (osteoblastic/  fibroblastic OS) | in vitro  in vivo  humans | circ ITCH | doxorubicin | RT-qPCR | 3/  OS tissue:40  (32 osteoblastic and 8 fibroblastic cancer tissues)  normal tissue:40; | miR-524/RASSF6 | Down-regulation of circ ITCH promotes OS development and resistance to doxorubicin. | 6 |
| Bai  2021  [164] | OS  (N/A) | in vitro  in vivo  humans | hsa_circ_0004674 | doxorubicin | RT-qPCR | 3/  OS tissue:41  drug sensitive:23  drug resistant:18 | miR-342-3p/FBN1/Wnt/β-catenin | Hsa_circ_0004674 promotes OS doxorubicin resistance by regulating the miR-342-3p/FBN1 axis. | 7 |
| Li  2021  [165] | OS  (N/A) | in vitro  in vivo  humans | circDOCK1 | cisplatin | RT-qPCR | 3/  OS tissues and paracancerous tissues:3 pairs  Blood sample:  OS patients:70  normal control:70 | miR-339-3p/IGF1R | CircDOCK1 promotes cisplatin resistance of OS  via the miR-339-3p/IGF1R axis. | 10 |
| Tang  2022  [166] | OS  (N/A) | in vitro  in vivo  humans | circ_ANKIB1 | doxorubicin | RT-qPCR | 3/  OS tissues and paracancerous tissues:61 pairs | miR-26b-5p/EZH2 | Circ RNA_ANKIB1 accelerates chemo-resistance of OS. | 7 |
| Chemotherapy (EWS) | | | | | | | | | |
| Jacques  2016  [167] | sarcoma  (OS/EWS) | in vitro | miR-193a-5p | cisplatin | RT-qPCR | 3 | TAp73β | MiRNA-193a-5p repression of p73 controls cisplatin chemoresistance in primary bone tumors. | 5 |
| Nakatani  2012  [168] | EWS | in vitro  humans | miR-34a | doxorubicin  vincristine | RT–qPCR | 2/  EWS patients:49 | p53 | Restoration of miR-34a activity increase tumour sensitivity to current drugs. | 9 |
| Robin  2012  [169] | EWS | in vitro  humans | miR-708 | doxorubicin  etoposide | RT-qPCR | 3/  EWS sample:23  bone marrow:2 | EYA3 | Downregulation of miR-708 resulted in increased chemoresistance in EWS. | 4 |
| Iida  2013  [170] | EWS | in vitro  humans | miR-125b | doxorubicin  etoposide  vincristine | RT-qPCR | 3/  EWS tissue:5 | p53  Bak | The overexpression of miR-125b in parental EWS cells resulted in enhanced drug resistance. | 4 |
| Chemotherapy (chondrosarcoma) | | | | | | | | | |
| Zhu  2014  [171] | chondrosarcoma  (N/A) | in vitro  humans | miR-100 | cisplatin | RT-qPCR | 3/  chondrosarcoma tissues:6 | mTOR | Overexpression of miR-100 in chondrosarcoma enhances the sensitivity to cisplatin. | 3 |
| Huang  2017  [172] | chondrosarcoma  (N/A) | in vitro  humans | miR-23b | cisplatin | RT-qPCR | 3/  tumor sample:20  nontumor sample:10 | Src-AKT | MiR-23b increases the cisplatin sensitivity of chondrosarcoma cells | 5 |
| Tang  2016  [173] | chondrosarcoma  (N/A) | in vitro  humans | miR-125b | doxorubicin | RT-qPCR | 3/  tumor tissue:20  normal tissue:20 | ErbB2 | MiR-125b acts as a tumor suppressor in chondrosarcoma cells by the sensitization to doxorubicin. | 4 |
| Chemotherapy (RMS, synovial sarcoma, GIST, ULMS, fibrosarcoma and malignant fibrous histiocytoma) | | | | | | | | | |
| Bharathy  2019  [174] | RMS | in vitro  in vivo | miR-27a | vincristine | RT-qPCR | 3 | PAX3:FOXO1 fusion oncogene | Re-expression of miR-27a led to PAX3:FOXO1 mRNA destabilization and chemotherapy sensitization in RMS cells in culture and in vivo. | 8 |
| Minami  2014  [175] | synovial sarcoma  (N/A) | in vitro  in vivo  humans | miR-17 | doxorubicin | RT-PCR | 3/  tumor tissue:7 | p21WAF1⁄CIP1 | MiR-17 induces drug resistance in synovial sarcoma cells. | 7 |
| Xu  2018  [176] | GIST | in vitro | miR‐22-3p | cisplatin | RT-qPCR | 3 | PTEN/PI3K/AKT | MiR-22-3p enhances the chemosensitivity of GIST. | 3 |
| Zhang  2020  [177] | ULMS | in vitro  in vivo  humans | miR-34a | doxorubicin | RT-qPCR | -/  ULMS tissues:27  myometrium:24  uterine leiomyoma:40 | JAK2/STAT3 | Inhibition of miR-34a/JAK2/STAT3 pathway promoted ULMS chemoresistance to doxorubicin. | 9 |
| Jain  2022  [178] | fibrosarcoma | in vitro | miR-197-5p | doxorubicin | RT-qPCR | 3 | ABCC1  MVP  p53 | MiR-197-5p increases doxorubicin-mediated anticancer cytotoxicity of fibrosarcoma cells. | 3 |
| Li  2021  [179] | malignant fibrous histiocytoma | in vitro | miR-206 | docetaxel  gemcitabine | qPCR | 3 | - | Has-miR-206 was lowly expressed in docetaxel-resistant MFH cells and inhibited the growth of MFH cells. | 7 |
| Targeted therapy (OS, GIST and synovial sarcoma) | | | | | | | | | |
| Wang  2021  [180] | OS  (N/A) | in vitro  in vivo  humans | miR-34a | cabozantinib | RT-qPCR | OS samples: N/A  adjacent normal samples: N/A | Notch pathway | MiR-34a enhanced sensitivity of OS cell to cabozantinib by inhibiting Notch signaling pathway. | 7 |
| Wang  2019  [181] | OS  (N/A) | in vitro  in vivo  humans | miR-596 | anlotinib | qPCR | -/  OS tissue:74  normal tissue:74; | Survivin | MiR-596 enhances the sensitivity of OS cells. | 7 |
| Wang  2019  [182] | OS  (N/A) | in vitro  in vivo  humans | miR-499a | erlotinib | RT–qPCR | 3/  OS patients: 10 | SHKBP1 | Down-regukation of miR-499 resulted in upregulation of SHKBP1, and increased erlotinib resistance. | 7 |
| Cao  2018  [183] | GIST | in vitro | lncRNA CCDC26 | imatinib | RT-qPCR | 3 | c-KIT | CCDC26 knockdown enhances resistance of GIST to imatinib. | 4 |
| Yan  2019  [184] | GIST | in vitro | lncRNA CCDC26 | imatinib | RT-qPCR | - | IGF-1R | Downregulation of lncRNA CCDC26 contributes to  imatinib resistance in human GIST | 3 |
| Zhang  2021  [185] | GIST | in vitro  in vivo | lncRNA-HOTAIR | imatinib | RT-qPCR | 3 | miR-130a/ATG2B | LncRNA-HOTAIR promotes the imatinib resistance of GIST cells | 7 |
| Shao  2021  [186] | GIST | in vitro  in vivo  humans | lncRNA RP11-616M22.7 | imatinib | RT-qPCR | GIST samples:20  adjacent normal samples: 20 | RASSF1 | RP11-616M22.7 overexpression induces resistance of GIST cells to imatinib. | 6 |
| Fan  2014  [187] | GIST | in vitro | miR-218 | imatinib | RT–qPCR | - | PI3K/AKT pathway | MicroRNA-218 increase the sensitivity of GIST to imatinib | 4 |
| Chen  2020  [188] | GIST | in vitro  in vivo | miR-30a | imatinib | RT-qPCR | 3 | beclin-1 | MiRNA-30a sensitizes GIST cells to imatinib | 7 |
| Shi  2015  [189] | GIST | in vitro  humans | miR-518a-5p | imatinib | RT-qPCR | -/  tumor tissue:20  normal tissue:20 | PIK3C2A | Low expression of miR-518a-5p is likely to cause resistance to imatinib in GISTs. | 5 |
| Huang  2018  [190] | GIST | in vitro  humans | miR-125a-5p | imatinib | RT–qPCR | 3/  Imatinib- resistant:13  Imatinib sensitivy:15 | pFAK | MiR-125a-5p contributes to imatinib resistance in GIST | 8 |
| Akcakaya  2014  [191] | GIST | in vitro  humans | miR-125a-5p  miR-107 | imatinib | RT–qPCR | 3/  imatinib resistant:10  imatinib sensitive:14 | PTPN18 | Overexpression of miR-125a-5p and miR-107 were associated with imatinib resistance in GIST. | 9 |
| Cao  2016  [192] | GIST | in vitro  humans | miR-21 | imatinib | RT-qPCR | 3/  GIST specimens: 31 | bcl-2 | MiRNA-21 sensitizes GIST cells to imatinib | 8 |
| Shiozawa  2017  [193] | synovial sarcoma | in vitro | miR-761 | pazopanib | RT-qPCR | 6 | TRIP6  LMNA  SIRT3 | MicroRNA-761 enhances pazopanib resistance in synovial sarcoma | 6 |
| Immunotherapy (sarcoma) | | | | | | | | |  |
| Pang  2021  [194] | sarcoma | - | ADAM6  C5orf58  CXCR2P1  FCGR2C  HCP5  HLA-H  NAPSB  NCF1B  NCF1C | immune checkpoint inhibitor | - | - | - | High expression of these lncRNAs reduced sensitivity to immune checkpoint inhibitors in sarcoma. | 6 |
| Radiotherapy (OS, chondrosarcoma and atypical teratoid/rhabdoid tumor) | | | | | | | | | |
| He  2020  [195] | OS  (N/A) | in vitro  humans | LINC00210 | X ray | RT-qPCR | -/  tumor tissues:53  adjacent normal tissues:53 | miR-342-3p/GFRA1 | LINC00210 knockdown improved the radiosensitivity of OS cells. | 3 |
| Yang  2018  [196] | OS | in vitro  in vivo | miR‑328‑3p | X-ray | RT-qPCR | - | H2AX | MiR‑328‑3p enhances the radiosensitivity of OS. | 4 |
| Li  2019  [197] | OS  (N/A) | in vitro,  in vivo  humans | miR-214 | X ray | qPCR | 3/  OS tissue:30  normal tissue:30; | PI3K/AKT | Upregulation of miR-214 induced radioresistance of OS. | 4 |
| Dai  2018  [198] | OS  (N/A) | in vitro  in vivo  humans | miR-513a-5p | X ray | RT-qPCR | -/  OS tissue:30  healthy controls:9 | APE1 | MiR-513a-5p could directly lead to radiosensitization. | 6 |
| Vares  2020  [199] | chondrosarcoma | in vitro  in vivo | miR-34 | carbon ions irradiation | RT-qPCR | 3 | FOXO3/KLF4 | MiR-34 overexpression may overcome treatment resistance of high-grade chondrosarcoma to carbon-ion irradiation. | 7 |
| Lee  2014  [200] | atypicalteratoid/rhabdoid tumor | in vitro  in vivo | miR142-3p | γradiation (IR) | RT-qPCR | 3 | SOX2  ADCY9 | Silencing of endogenous miR142-3p promoted the mesenchymal transitional and radioresistant properties of ATRT cells. | 7 |
| Biomarkers for monitoring treatment response | | | | | | | | | |
| Polvani  2022  [201] | OS  (osteoblastic/  telangiectatic) | humans | lncRNA growth  arrest-specific 5 (GAS5) | - | RT-qPCR | OS tissue:10  osteoblastic OS:8  telangiectatic OS:2 | - | GAS5 is significantly increased in patients with a good prognosis and is expressed differently between chemosensitive and chemoresistant osteosarcoma patients. | 4 |
| Zhu  2015  [202] | OS  (N/A) | in vitro  humans | lncRNA ENST00000563280 | doxorubicin | RT-qPCR | chemosesitive group:30  chemoresistant group:30 | - | LncRNA ENST00000563280 was distinctly increased in specimens of OS patients with a poor chemoresponse compared to those with a good chemoresponse. | 6 |
| Yuan  2012  [203] | OS  (N/A) | humans | miR-21 | methotrexate  cisplatin  adriamycin  bleomycin/  cyclo-phosphamide/  dactinomycin | RT-qPCR | OS patients: 65  healthy controls: 30 | - | High serum miR-21 was significantly correlated with advanced Enneking stage and chemotherapeutic resistance in patients with OS. | 5 |
| Lou  2016  [204] | OS  (N/A) | humans | miR-125b | - | RT-qPCR | resectable OS: 82  unresectable OS: 56 | - | Negative correlation was found between miR-125b expression and response to chemotherapy. | 4 |
| Han  2020  [205] | OS | in vitro | hsa_circ_0008336  hsa_circ_0004664  hsa_circ_0003302 | cisplatin | RNA sequencing  RT-qPCR | - | - | Hsa_circ_0008336, hsa_circ_0004664, and hsa_circ_0003302 were upregulated in cisplatin-resistant cells and may be involved in the pathology of cisplatin resistance in OS. | 3 |
| Zhu  2018  [206] | OS  (N/A) | in vitro  humans | hsa_circ_0004674 | cisplatin  doxorubicin | RT-qPCR | OS tissue: 60  normal tissue:40 | - | Hsa_circ_0004674 was distinctly increased in OS chemoresistant cells and tissues, related to poor prognosis. | 8 |
| YAMADA  2021  [207] | sarcoma | in vitro  humans | lncRNA HAR1B | pazopanib | RT-qPCR  microarray | -/  responder: 16  non-responder:23 | - | LncRNA HAR1B expression is higher in responder than in non-responder in patients with sarcoma. | 4 |
| Yan  2017  [208] | GIST | humans | lncRNAs | imatinib | RT-qPCR | 3/  normal tissues:3  primaryGIST samples:3  imatinib-resistant samples:3 | - | LncRNAs may serve as potential biomarkers or drug targets for imatinib-resistant GISTs. | 5 |
| Amirnasr  2019  [209] | GIST | humans | miRNAs | imatinib | RT-qPCR | imatinib-naïve (IM-n) tissue:33  imatinib-resistant (IM-r) tissue:20 | - | Differentially expressed of miRNAs identified between IM-n and IM-r GIST and highlighted the key miRNAs might be putative treatment targets. | 7 |
| Kou  2018  [210] | GIST | humans | miR-518e-5p | imatinib | PCR | serum samples:  imatinib resistant GIST patients:39,  imatinib-sensitive GIST patients:37,  healthy controls:28 | - | Serum miR-518e-5p is a potential biomarker for secondary imatinib-resistant GIST. | 4 |
| Gao  2013  [211] | GIST | humans | miR-320a | imatinib | RT-qPCR | 3/  pre-imatinib:3  imatinib-resistant:12 | - | MiR-320a downregulation is associated with imatinib resistance in GIST. | 4 |
| Zhang  2018  [212] | GIST | Gene Expression Omnibus database | miR-28-5p  miR-125a-5p | imatinib | - | - | GnRH | Hsa-miR-28-5p and hsa-miR-125a-5p may be able to serve as prognostic markers for imatinib-response in GIST patients. | 5 |

Histological subtype of sarcomas (OS, chondrosarcoma and synovial sarcoma) among the studies explored human tissues have been identified. Abbreviations:ABCG2: ATP binding cassette subfamily G member 2; ADCY9: adenylate cyclase 9; ALDH1A3: aldehyde dehydrogenase 1 family member A3; ATG2B: autophagy-related 2B; ATG4B: autophagy-related 4B; AGTR1: angiotensin II type 1 receptor; AMPKα1: AMP-activated protein kinase alpha 1; BCL2L1: Bcl-2-like protein 1; BMP9: bone morphogenetic protein 9; CD44: cluster of differentiation 44;circ-CHI3L1.2: circRNA circ-chitinase 3-like 1.2; CRYAB: aB-crystallin; DNMT1:Kcnq1/DNA methyltransferase 1; DTL: denticleless protein homolog; DLL1: Delta-like ligand 1; FBN1: fibrillin-1; ERK: extraneous signal regulated kinase; EWS : Ewing’s sarcoma; FGFR: fibroblast growth factor receptor; FOSL2: FOS like 2;EZH2: zeste homolog 2; FZD4: Frizzled class receptor 4; GADD45A: DNA damage-inducible alpha; GFRA1: GDNF family receptor alpha-1; GIST: gastrointestinal stromal tumor; GFRA1: GDNF receptor alpha 1.HDAC4: histone deacetylase 4; HMGB1:high-mobility group box 1; HIF1: hypoxia‑inducible factor-1; IP3K2: inositol 1,4,5-trisphosphate kinase 2; IRS2: insulin receptor substrate 2;KEAP1: kelch-like ECH-associated protein 1;KLF8: Krüppel-like factor 8; KLF12: Krüppel-like factor 12; KCNQ1OT1:lncRNA KCNQ1 opposite strand/antisense transcript 1; LC3: rabbit anti‑light chain 3; LPAATb: lysophosphatidic acid acyltransferase; LPAATβ: lysophosphatidic acid acyltransferase; MAPK7: mitogen activated protein kinase 7; MCL1: myeloid cell leukemia 1; MRP1: multidrug resistance. associated protein-1; MTDH: metadherin; MCL1: myeloid cell leukemia-1; NRF2: nuclearfactor erythroid 2-related factor 2; OS: osteosarcoma; PD‐L1: programmed cell death receptor‐1; PTN: pleiotrophin; PI3K: phosphatidylinositol 3-kinase; PLK1: Polo-like kinase 1; PPP2R2A: PP2A subunit B;RAB11B-AS1: lncRNA RAB11B antisense RNA; RUNX2: Runt-related transcription factor 2; RASSF6: Ras association (RA) domain family; RMS: Rhabdomyosarcoma; S100A11: S100 calcium‑binding protein A11; SDC2: homo sapiens syndecan 2; SOX2: sex-determiningregion Y Box 2; TRIAP1: TP53-regulated inhibitor of apoptosis 1; TGM2: transglutaminase-2;USP1: ubiquitin‑specific protease 1; ULMS: uterine leiomyosarcoma;XIAP: X-linked inhibitor of apoptosis protein; ZNF32:zinc finger protein 32

Reference

1. Xie X, Liu W, Duan Z, Li X, Zhang L and Yang G (2020) LncRNA NORAD targets miR-410-3p to regulate drug resistance sensitivity of osteosarcoma. Cellular and Molecular Biology 66. doi: 10.14715/cmb/2020.66.3.22

2. Li G and Zhu Y (2019) Effect of lncRNA ANRIL knockdown on proliferation and cisplatin chemoresistance of osteosarcoma cells in vitro. Pathol Res Pract 215:931-938. doi: 10.1016/j.prp.2019.01.042

3. Lee AM, Ferdjallah A, Moore E, Kim DC, Nath A, Greengard E and Huang RS (2021) Long Non-Coding RNA ANRIL as a Potential Biomarker of Chemosensitivity and Clinical Outcomes in Osteosarcoma. Int J Mol Sci 22. doi: 10.3390/ijms222011168

4. Wang Y, Zhang L, Zheng X, Zhong W, Tian X, Yin B, Tian K and Zhang W (2016) Long non-coding RNA LINC00161 sensitises osteosarcoma cells to cisplatin-induced apoptosis by regulating the miR-645-IFIT2 axis. Cancer Letters 382:137-146. doi: 10.1016/j.canlet.2016.08.024

5. Zhang QQ, Xu SL, Ding C, Ma CC, Yuan TS, Hua CC and Wang XH (2021) LncRNA FOXD2-AS1 knockdown inhibits the resistance of human osteosarcoma cells to cisplatin by inhibiting miR-143 expression. European review for medical and pharmacological sciences 25:678-686. doi: 10.26355/eurrev_202101_24629

6. Li R, Ruan Q, Zheng J, Zhang B and Yang H (2021) LINC01116 Promotes Doxorubicin Resistance in Osteosarcoma by Epigenetically Silencing miR-424-5p and Inducing Epithelial-Mesenchymal Transition. Frontiers in Pharmacology 12. doi: 10.3389/fphar.2021.632206

7. Cheng Y, Shen X, Zheng M, Zou G and Shen Y (2019) Knockdown Of lncRNA NCK-AS1 Regulates Cisplatin Resistance Through Modulating miR-137 In Osteosarcoma Cells. Onco Targets Ther 12:11057-11068. doi: 10.2147/OTT.S228199

8. Zhang L, Wang Y, Li X, Xia X, Li N, He R, He H, Han C and Zhao W (2017) ZBTB7A Enhances Osteosarcoma Chemoresistance by Transcriptionally Repressing lncRNALINC00473-IL24 Activity. Neoplasia 19:908-918. doi: 10.1016/j.neo.2017.08.008

9. Sun YF, Wang Y, Li XD and Wang H (2022) SNHG15, a p53-regulated lncRNA, suppresses cisplatin-induced apoptosis and ROS accumulation through the miR-335-3p/ZNF32 axis. Am J Cancer Res 12:816-828.

10. Wang F, Kong L, Pu Y, Chao F, Zang C, Qin W, Zhao F and Cai S (2021) Long Noncoding RNA DICER1-AS1 Functions in Methylation Regulation on the Multi-Drugresistance of Osteosarcoma Cells via miR-34a-5p and GADD45A. Frontiers in Oncology 11. doi: 10.3389/fonc.2021.685881

11. Song L, Zhou Z, Gan Y, Li P, Xu Y, Zhang Z, Luo F, Xu J, Zhou Q and Dai F (2019) Long noncoding RNA OIP5-AS1 causes cisplatin resistance in osteosarcoma through inducing the LPAATbeta/PI3K/AKT/mTOR signaling pathway by sponging the miR-340-5p. J Cell Biochem 120:9656-9666. doi: 10.1002/jcb.28244

12. Han Z and Shi L (2018) Long non-coding RNA LUCAT1 modulates methotrexate resistance in osteosarcoma via miR-200c/ABCB1 axis. Biochemical and Biophysical Research Communications 495:947-953. doi: 10.1016/j.bbrc.2017.11.121

13. Zhou Q, Hu T and Xu Y (2019) Anticancer potential of TUG1 knockdown in cisplatin-resistant osteosarcoma through inhibition of MET/Akt signalling. Journal of Drug Targeting 28:204-211. doi: 10.1080/1061186x.2019.1644651

14. Hu T, Fei Z, Su H, Xie R and Chen L (2019) Polydatin inhibits proliferation and promotes apoptosis of doxorubicin-resistant osteosarcoma through LncRNA TUG1 mediated suppression of Akt signaling. Toxicology and Applied Pharmacology 371:55-62. doi: 10.1016/j.taap.2019.04.005

15. Sun Z-Y, Jian Y-K, Zhu H-Y and Li B (2019) lncRNAPVT1 targets miR-152 to enhance chemoresistance of osteosarcoma to gemcitabine through activating c-MET/PI3K/AKT pathway. Pathology - Research and Practice 215:555-563. doi: 10.1016/j.prp.2018.12.013

16. Liu C, Han X, Li B, Huang S, Zhou Z, Wang Z and Wang W (2021) MALAT-1 is Associated with the Doxorubicin Resistance in U-2OS Osteosarcoma Cells. Cancer Manag Res 13:6879-6889. doi: 10.2147/CMAR.S304922

17. Pu Y, Tan Y, Zang C, Zhao F, Cai C, Kong L, Deng H, Chao F, Xia R, Xie M, Ge F, Pan Y, Cai S and Huang D (2021) LAMTOR5-AS1 regulates chemotherapy-induced oxidative stress by controlling the expression level and transcriptional activity of NRF2 in osteosarcoma cells. Cell Death Dis 12:1125. doi: 10.1038/s41419-021-04413-0

18. Shen P and Cheng Y (2020) Long noncoding RNA lncARSR confers resistance to Adriamycin and promotes osteosarcoma progression. Cell Death Dis 11:362. doi: 10.1038/s41419-020-2573-2

19. Wang L, Luo Y, Zheng Y, Zheng L, Lin W, Chen Z, Wu S, Chen J and Xie Y (2020) Long non-coding RNA LINC00426 contributes to doxorubicin resistance by sponging miR-4319 in osteosarcoma. Biology Direct 15. doi: 10.1186/s13062-020-00265-4

20. Zhu K-P, Zhang C-L, Ma X-L, Hu J-P, Cai T and Zhang L (2019) Analyzing the Interactions of mRNAs and ncRNAs to Predict Competing Endogenous RNA Networks in Osteosarcoma Chemo-Resistance. Molecular Therapy 27:518-530. doi: 10.1016/j.ymthe.2019.01.001

21. Fu D, Lu C, Qu X, Li P, Chen K, Shan L and Zhu X (2019) LncRNA TTN-AS1 regulates osteosarcoma cell apoptosis and drug resistance via the miR-134-5p/MBTD1 axis. Aging 11:8374-8385. doi: 10.18632/aging.102325

22. Zhang L, Zhao G, Ji S, Yuan Q and Zhou H (2020) Downregulated Long Non-Coding RNA MSC-AS1 Inhibits Osteosarcoma Progression and Increases Sensitivity to Cisplatin by Binding to MicroRNA-142. Medical Science Monitor 26. doi: 10.12659/msm.921594

23. Liu L and Wang S (2020) Long Non-Coding RNA OIP5-AS1 Knockdown Enhances CDDP Sensitivity in Osteosarcoma via miR-377-3p/FOSL2 Axis. Onco Targets Ther 13:3853-3866. doi: 10.2147/OTT.S232918

24. Gu Z, Zhou Y, Cao C, Wang X, Wu L and Ye Z (2020) TFAP2C-mediated LINC00922 signaling underpins doxorubicin-resistant osteosarcoma. Biomedicine & Pharmacotherapy 129. doi: 10.1016/j.biopha.2020.110363

25. Sun X, Tian C, Zhang H, Han K, Zhou M, Gan Z, Zhu H and Min D (2020) Long noncoding RNA OIP5-AS1 mediates resistance to doxorubicin by regulating miR-137-3p/PTN axis in osteosarcoma. Biomedicine & Pharmacotherapy 128. doi: 10.1016/j.biopha.2020.110201

26. Meng Y, Hao D, Huang Y, Jia S, Zhang J, He X, Sun L and Liu D (2020) Positive feedback loop SP1/MIR17HG/miR-130a-3p promotes osteosarcoma proliferation and cisplatin resistance. Biochemical and Biophysical Research Communications 521:739-745. doi: 10.1016/j.bbrc.2019.10.180

27. Liang R, Liu Z, Chen Z, Yang Y, Li Y, Cui Z, Chen A, Long Z, Chen J, Lu J, Huang B and Li Q (2019) Long noncoding RNA DNAJC3-AS1 promotes osteosarcoma progression via its sense-cognate gene DNAJC3. Cancer Medicine 8:761-772. doi: 10.1002/cam4.1955

28. Shi J, Fu Q, Yang P, Yi Z, Liu S and Wang K (2020) Long noncoding

RNA PWRN1

is lowly expressed in osteosarcoma and modulates cancer proliferation and migration by targeting

hsa‐miR

‐214‐5p. IUBMB Life 72:2444-2453. doi: 10.1002/iub.2370

29. Zhu K, Yuan Y, Wen J, Chen D, Zhu W, Ouyang Z and Wang W (2020) LncRNA Sox2OT-V7 promotes doxorubicin-induced autophagy and chemoresistance in osteosarcoma via tumor-suppressive miR-142/miR-22. Aging 12:6644-6666. doi: 10.18632/aging.103004

30. Wen J-F, Jiang Y-Q, Li C, Dai X-K, Wu T and Yin W-Z (2020) LncRNA-SARCC sensitizes osteosarcoma to cisplatin through the miR-143-mediated glycolysis inhibition by targeting Hexokinase 2. Cancer Biomarkers 28:231-246. doi: 10.3233/cbm-191181

31. Wang Z, Liu Z and Wu S (2017) Long non-coding RNA CTA sensitizes osteosarcoma cells to doxorubicin through inhibition of autophagy. Oncotarget 8:31465-31477. doi: 10.18632/oncotarget.16356

32. Guo J, Dou D, Zhang T and Wang B (2020) HOTAIR Promotes Cisplatin Resistance of Osteosarcoma Cells by Regulating Cell Proliferation, Invasion, and Apoptosis via miR-106a-5p/STAT3 Axis. Cell Transplantation 29. doi: 10.1177/0963689720948447

33. Zhou B, Li L, Li Y, Sun H and Zeng C (2018) Long noncoding RNA SNHG12 mediates doxorubicin resistance of osteosarcoma via miR-320a/MCL1 axis. Biomedicine & Pharmacotherapy 106:850-857. doi: 10.1016/j.biopha.2018.07.003

34. Zhang C-L, Zhu K-P, Shen G-Q and Zhu Z-S (2015) A long non-coding RNA contributes to doxorubicin resistance of osteosarcoma. Tumor Biology 37:2737-2748. doi: 10.1007/s13277-015-4130-7

35. Cheng FH, Zhao ZS and Liu WD (2019) Long non-coding RNA ROR regulated ABCB1 to induce cisplatin resistance in osteosarcoma by sponging miR-153-3p. European review for medical and pharmacological sciences 23:7256-7265. doi: 10.26355/eurrev_201909_18828

36. Zhang CL, Zhu KP and Ma XL (2017) Antisense lncRNA FOXC2-AS1 promotes doxorubicin resistance in osteosarcoma by increasing the expression of FOXC2. Cancer Lett 396:66-75. doi: 10.1016/j.canlet.2017.03.018

37. Kun-Peng Z, Chun-Lin Z, Xiao-Long M and Lei Z (2019) Fibronectin-1 modulated by the long noncoding RNA OIP5-AS1/miR-200b-3p axis contributes to doxorubicin resistance of osteosarcoma cells. J Cell Physiol 234:6927-6939. doi: 10.1002/jcp.27435

38. Li Z, Wang Y, Hu R, Xu R and Xu W (2018) LncRNA B4GALT1-AS1 recruits HuR to promote osteosarcoma cells stemness and migration via

enhancing YAP transcriptional activity. Cell Proliferation 51. doi: 10.1111/cpr.12504

39. Hu Y, Yang Q, Wang L, Wang S, Sun F, Xu D and Jiang J (2018) Knockdown of the oncogene lncRNA NEAT1 restores the availability of miR-34c and improves the sensitivity to cisplatin in osteosarcoma. Bioscience Reports 38. doi: 10.1042/bsr20180375

40. Li Z, Zhao L and Wang Q (2016) Overexpression of long non-coding RNA HOTTIP increases chemoresistance of osteosarcoma cell by activating the Wnt/β-catenin pathway. American journal of translational research 8:2385-2393.

41. Chen Z, Liu Z, Yang Y, Zhu Z, Liang R, Huang B, Wu D, Yang L, Lu H, Jin D and Li Q (2018) Long non-coding RNA prevents osteosarcoma development and progression via its natural antisense transcript. Oncotarget 9:26770-26786. doi: 10.18632/oncotarget.24247

42. Kun-Peng Z, Xiao-Long M and Chun-Lin Z (2017) LncRNA FENDRR sensitizes doxorubicin-resistance of osteosarcoma cells through down-regulating ABCB1 and ABCC1. Oncotarget 8:71881-71893. doi: 10.18632/oncotarget.17985

43. Zhang J, Rao D, Ma H, Kong D, Xu X and Lu H (2020) LncRNA SNHG15 contributes to doxorubicin resistance of osteosarcoma cells through targeting the miR-381-3p/GFRA1 axis. Open Life Sciences 15:871-883. doi: 10.1515/biol-2020-0086

44. Tang J, Zhu Z, Dong S, Wang Y, Wang J, Chen H and Duan G (2022) Long non-coding RNA long intergenic non-coding 00641 mediates cell progression with stimulating cisplatin-resistance in osteosarcoma cells via microRNA-320d/myeloid cell leukemia-1 axis. Bioengineered 13:7238-7252. doi: 10.1080/21655979.2022.2045090

45. Liu M, Wu L, Cai C, Liu L and Xu Y (2020) MicroRNA-187 suppresses the proliferation migration and invasion of human osteosarcoma cells by targeting MAPK7. Journal of B.U.ON. : official journal of the Balkan Union of Oncology 25:472-478.

46. Patil SL, Palat A, Pan Y, Rajapakshe K, Mirchandani R, Bondesson M, Yustein JT, Coarfa C and Gunaratne PH (2019) MicroRNA-509-3p inhibits cellular migration, invasion, and proliferation, and sensitizes osteosarcoma to cisplatin. Scientific Reports 9. doi: 10.1038/s41598-019-55170-2

47. Wang Y, Shang G, Wang W, Qiu E, Pei Y and Zhang X (2020) Magnoflorine inhibits the malignant phenotypes and increases cisplatin sensitivity of osteosarcoma cells via regulating miR-410-3p/HMGB1/NF-κB pathway. Life Sciences 256. doi: 10.1016/j.lfs.2020.117967

48. Luo DJ, Li LJ, Huo HF, Liu XQ, Cui HW and Jiang DM (2019) MicroRNA-29b sensitizes osteosarcoma cells to doxorubicin by targeting matrix metalloproteinase 9 (MMP-9) in osteosarcoma. European review for medical and pharmacological sciences 23:1434-1442. doi: 10.26355/eurrev_201902_17100

49. Li S, Bai H, Chen X, Gong S, Xiao J, Li D, Li L, Jiang Y, Li T, Qin X, Yang H, Wu C, You F and Liu Y (2020) Soft Substrate Promotes Osteosarcoma Cell Self-Renewal, Differentiation, and Drug Resistance Through miR-29b and Its Target Protein Spin 1. ACS Biomaterials Science & Engineering 6:5588-5598. doi: 10.1021/acsbiomaterials.0c00816

50. Vanas V, Haigl B, Stockhammer V and Sutterluty-Fall H (2016) MicroRNA-21 Increases Proliferation and Cisplatin Sensitivity of Osteosarcoma-Derived Cells. PLoS One 11:e0161023. doi: 10.1371/journal.pone.0161023

51. Ziyan W and Yang L (2014) MicroRNA-21 regulates the sensitivity to cisplatin in a human osteosarcoma cell line. Irish Journal of Medical Science (1971 -) 185:85-91. doi: 10.1007/s11845-014-1225-x

52. Bazavar M, Fazli J, Valizadeh A, Ma B, Mohammadi E, Asemi Z, Alemi F, Maleki M, Xing S and Yousefi B (2020) miR-192 enhances sensitivity of methotrexate drug to MG-63 osteosarcoma cancer cells. Pathology - Research and Practice 216. doi: 10.1016/j.prp.2020.153176

53. Xu R, Liu S, Chen H and Lao L (2016) MicroRNA-30a downregulation contributes to chemoresistance of osteosarcoma cells through activating Beclin-1-mediated autophagy. Oncology reports 35:1757-1763. doi: 10.3892/or.2015.4497

54. Wang P, Zhao Zq, Guo Sb, Yang Ty, Chang Zq, Li Dh, Zhao W, Wang Yx, Sun C, Wang Y and Feng W (2019) Roles of microRNA‐22 in Suppressing Proliferation and Promoting Sensitivity of Osteosarcoma Cells

via

Metadherin‐mediated Autophagy. Orthopaedic Surgery 11:285-293. doi: 10.1111/os.12442

55. Li X, Wang S, Chen Y, Liu G and Yang X (2014) miR-22 targets the 3' UTR of HMGB1 and inhibits the HMGB1-associated autophagy in osteosarcoma cells during chemotherapy. Tumour Biol 35:6021-8. doi: 10.1007/s13277-014-1797-0

56. Zhu J, Cui K, Cui Y, Ma C and Zhang Z (2020) PLK1 Knockdown Inhibits Cell Proliferation and Cell Apoptosis, and PLK1 Is Negatively Regulated by miR-4779 in Osteosarcoma Cells. DNA and Cell Biology 39:747-755. doi: 10.1089/dna.2019.5002

57. Zhang X, Guo Q, Chen J and Chen Z (2015) Quercetin Enhances Cisplatin Sensitivity of Human Osteosarcoma Cells by Modulating microRNA-217-KRAS Axis. Molecules and Cells 38:638-642. doi: 10.14348/molcells.2015.0037

58. Song L, Duan P, Gan Y, Li P, Zhao C, Xu J, Zhang Z and Zhou Q (2017) MicroRNA-340-5p modulates cisplatin resistance by targeting LPAATbeta in osteosarcoma. Braz J Med Biol Res 50:e6359. doi: 10.1590/1414-431X20176359

59. Song YD, Li DD, Guan Y, Wang YL and Zheng J (2017) miR-214 modulates cisplatin sensitivity of osteosarcoma cells through regulation of anaerobic glycolysis. Cellular and Molecular Biology 63. doi: 10.14715/cmb/2017.63.9.14

60. Di Fiore R, Drago-Ferrante R, Pentimalli F, Di Marzo D, Forte IM, Carlisi D, De Blasio A, Tesoriere G, Giordano A and Vento R (2016) Let-7d miRNA Shows Both Antioncogenic and Oncogenic Functions in Osteosarcoma-Derived 3AB-OS Cancer Stem Cells. Journal of Cellular Physiology 231:1832-1841. doi: 10.1002/jcp.25291

61. Zhang Y, Duan G and Feng S (2015) MicroRNA-301a modulates doxorubicin resistance in osteosarcoma cells by targeting AMP-activated protein kinase alpha 1. Biochemical and Biophysical Research Communications 459:367-373. doi: 10.1016/j.bbrc.2015.02.101

62. Meng CY, Zhao ZQ, Bai R, Zhao W, Wang YX, Sun L, Sun C, Feng W and Guo SB (2020) MicroRNA‑22 regulates autophagy and apoptosis in cisplatin resistance of osteosarcoma. Molecular Medicine Reports. doi: 10.3892/mmr.2020.11447

63. Meng CY, Zhao ZQ, Bai R, Zhao W, Wang YX, Xue HQ, Sun L, Sun C, Feng W and Guo SB (2020) MicroRNA‑22 mediates the cisplatin resistance of osteosarcoma cells by inhibiting autophagy via the PI3K/Akt/mTOR pathway. Oncology Reports. doi: 10.3892/or.2020.7492

64. Yuan G, Zhao Y, Wu D, Gao C and Jiao Z (2018) miRNA-20a upregulates TAK1 and increases proliferation in osteosarcoma cells. Future oncology (London, England) 14:461-469. doi: 10.2217/fon-2017-0490

65. Lin BC, Huang D, Yu CQ, Mou Y, Liu YH, Zhang DW and Shi FJ (2016) MicroRNA-184 Modulates Doxorubicin Resistance in Osteosarcoma Cells by Targeting BCL2L1. Med Sci Monit 22:1761-5. doi: 10.12659/msm.896451

66. Lei W, Yan C, Ya J, Yong D, Yujun B and Kai L (2018) MiR-199a-3p affects the multi-chemoresistance of osteosarcoma through targeting AK4. BMC Cancer 18:631. doi: 10.1186/s12885-018-4460-0

67. Pu Y, Zhao F, Wang H, Cai W, Gao J, Li Y and Cai S (2016) MiR-34a-5p promotes the multi-drug resistance of osteosarcoma by targeting the CD117 gene. Oncotarget 7:28420-28434. doi: 10.18632/oncotarget.8546

68. Song B, Wang Y, Titmus MA, Botchkina G, Formentini A, Kornmann M and Ju J (2010) Molecular mechanism of chemoresistance by miR-215 in osteosarcoma and colon cancer cells. Molecular cancer 9:96. doi: 10.1186/1476-4598-9-96

69. Li Q-C, Xu H, Wang X, Wang T and Wu J (2017) miR-34a increases cisplatin sensitivity of osteosarcoma cells in vitro through up-regulation of c-Myc and Bim signal. Cancer Biomarkers 21:135-144. doi: 10.3233/cbm-170452

70. Zhou Y, Zhao RH, Tseng KF, Li KP, Lu ZG, Liu Y, Han K, Gan ZH, Lin SC, Hu HY and Min DL (2016) Sirolimus induces apoptosis and reverses multidrug resistance in human osteosarcoma cells in vitro via increasing microRNA-34b expression. Acta Pharmacol Sin 37:519-29. doi: 10.1038/aps.2015.153

71. Chang Z, Huo L, Li K, Wu Y and Hu Z (2014) Blocked autophagy by miR-101 enhances osteosarcoma cell chemosensitivity in vitro. ScientificWorldJournal 2014:794756. doi: 10.1155/2014/794756

72. Gao Y, Feng Y, Shen JK, Lin M, Choy E, Cote GM, Harmon DC, Mankin HJ, Hornicek FJ and Duan Z (2015) CD44 is a direct target of miR-199a-3p and contributes to aggressive progression in osteosarcoma. Scientific Reports 5. doi: 10.1038/srep11365

73. Di Fiore R, Drago-Ferrante R, Pentimalli F, Di Marzo D, Forte IM, D’Anneo A, Carlisi D, De Blasio A, Giuliano M, Tesoriere G, Giordano A and Vento R (2014) MicroRNA-29b-1 impairs in vitro cell proliferation, self-renewal and chemoresistance of human osteosarcoma 3AB-OS cancer stem cells. International Journal of Oncology 45:2013-2023. doi: 10.3892/ijo.2014.2618

74. Wei R, Cao G, Deng Z, Su J and Cai L (2016) miR-140-5p attenuates chemotherapeutic drug-induced cell death by regulating autophagy through inositol 1,4,5-trisphosphate kinase 2 (IP3k2) in human osteosarcoma cells. Bioscience Reports 36. doi: 10.1042/bsr20160238

75. Xie Z, Xu J, Peng L, Gao Y, Zhao H and Qu Y (2018) miR-149 promotes human osteocarcinoma progression via targeting bone morphogenetic protein 9 (BMP9). Biotechnology letters 40:47-55. doi: 10.1007/s10529-017-2445-8

76. Novello C, Pazzaglia L, Conti A, Quattrini I, Pollino S, Perego P, Picci P and Benassi MS (2014) p53-dependent activation of microRNA-34a in response to etoposide-induced DNA damage in osteosarcoma cell lines not impaired by dominant negative p53 expression. PloS one 9:e114757. doi: 10.1371/journal.pone.0114757

77. Chen L, Jiang K, Jiang H and Wei P (2014) miR-155 mediates drug resistance in osteosarcoma cells via inducing autophagy. Exp Ther Med 8:527-532. doi: 10.3892/etm.2014.1752

78. Li Y, Jiang W, Hu Y, Da Z, Zeng C, Tu M, Deng Z and Xiao W (2016) MicroRNA-199a-5p inhibits cisplatin-induced drug resistance via inhibition of autophagy in osteosarcoma cells. Oncol Lett 12:4203-4208. doi: 10.3892/ol.2016.5172

79. Huang Z, Huang L, Liu L, Wang L, Lin W, Zhu X, Su W and Lv C (2021) Knockdown of microRNA-203 reduces cisplatin chemo-sensitivity to osteosarcoma cell lines MG63 and U2OS in vitro by targeting RUNX2. Journal of Chemotherapy 33:328-341. doi: 10.1080/1120009x.2021.1899441

80. Zou Y, Yang J, Wu J, Luo C and Huang Y (2017) miR‑133b induces chemoresistance of osteosarcoma cells to cisplatin treatment by promoting cell death, migration and invasion. Oncology Letters. doi: 10.3892/ol.2017.7432

81. Zhu M, Wu Y, Wang Z, Lin M, Su B, Li C, Liang F and Chen X (2020) miR‑128‑3p serves as an oncogenic microRNA in osteosarcoma cells by downregulating ZC3H12D. Oncology Letters 21. doi: 10.3892/ol.2020.12413

82. Zhang B, Liu Y and Zhang J (2018) Silencing of miR‑19a‑3p enhances osteosarcoma cells chemosensitivity by elevating the expression of tumor suppressor PTEN. Oncology Letters. doi: 10.3892/ol.2018.9592

83. Jiang L, He A, He X and Tao C (2015) MicroRNA-126 enhances the sensitivity of osteosarcoma cells to cisplatin and methotrexate. Oncology Letters 10:3769-3778. doi: 10.3892/ol.2015.3790

84. Chen X, Chen X-G, Hu X, Song T, Ou X, Zhang C, Zhang W and Zhang C (2016) MiR-34a and miR-203 Inhibit Survivin Expression to Control Cell Proliferation and Survival in Human Osteosarcoma Cells. Journal of Cancer 7:1057-1065. doi: 10.7150/jca.15061

85. Yu T, Chen D, Zhang L and Wan D (2019) <p>microRNA-26a-5p Promotes Proliferation and Migration of Osteosarcoma Cells by Targeting <em>HOXA5</em> in vitro and in vivo</p>. OncoTargets and Therapy Volume 12:11555-11565. doi: 10.2147/ott.S232100

86. Pu Y, Zhao F, Wang H and Cai S (2017) MiR-34a-5p promotes multi-chemoresistance of osteosarcoma through down-regulation of the DLL1 gene. Sci Rep 7:44218. doi: 10.1038/srep44218

87. Song B, Wang Y, Xi Y, Kudo K, Bruheim S, Botchkina GI, Gavin E, Wan Y, Formentini A, Kornmann M, Fodstad O and Ju J (2009) Mechanism of chemoresistance mediated by miR-140 in human osteosarcoma and colon cancer cells. Oncogene 28:4065-74. doi: 10.1038/onc.2009.274

88. Pu Y, Zhao F, Li Y, Cui M, Wang H, Meng X and Cai S (2017) The miR-34a-5p promotes the multi-chemoresistance of osteosarcoma via repression of the AGTR1 gene. BMC Cancer 17. doi: 10.1186/s12885-016-3002-x

89. Osaki S, Tazawa H, Hasei J, Yamakawa Y, Omori T, Sugiu K, Komatsubara T, Fujiwara T, Sasaki T, Kunisada T, Yoshida A, Urata Y, Kagawa S, Ozaki T and Fujiwara T (2016) Ablation of MCL1 expression by virally induced microRNA-29 reverses chemoresistance in human osteosarcomas. Scientific Reports 6. doi: 10.1038/srep28953

90. Zhang Q, Wu J, Zhang X, Cao L, Wu Y and Miao X (2021) Transcription factor ELK1 accelerates aerobic glycolysis to enhance osteosarcoma chemoresistance through miR-134/PTBP1 signaling cascade. Aging 13:6804-6819. doi: 10.18632/aging.202538

91. Zhao F, Pu Y, Cui M, Wang H and Cai S (2017) MiR-20a-5p represses the multi-drug resistance of osteosarcoma by targeting the SDC2 gene. Cancer cell international 17:100. doi: 10.1186/s12935-017-0470-2

92. Wang H, Zhao F, Cai S and Pu Y (2019) MiR-193a regulates chemoresistance of human osteosarcoma cells via repression of IRS2. Journal of Bone Oncology 17. doi: 10.1016/j.jbo.2019.100241

93. Guo X, Yu L, Zhang Z, Dai G, Gao T and Guo W (2017) miR-335 negatively regulates osteosarcoma stem cell-like properties by targeting POU5F1. Cancer cell international 17:29. doi: 10.1186/s12935-017-0398-6

94. Cheng M, Duan PG, Gao ZZ and Dai M (2020) MicroRNA‑487b‑3p inhibits osteosarcoma chemoresistance and metastasis by targeting ALDH1A3. Oncology Reports 44:2691-2700. doi: 10.3892/or.2020.7814

95. Yu WC, Chen HH, Qu YY, Xu CW, Yang C and Liu Y (2019) MicroRNA-221 promotes cisplatin resistance in osteosarcoma cells by targeting PPP2R2A. Biosci Rep 39. doi: 10.1042/BSR20190198

96. Tsai HC, Chang AC, Tsai CH, Huang YL, Gan L, Chen CK, Liu SC, Huang TY, Fong YC and Tang CH (2019) CCN2 promotes drug resistance in osteosarcoma by enhancing ABCG2 expression. J Cell Physiol 234:9297-9307. doi: 10.1002/jcp.27611

97. Meng Y, Gao R, Ma J, Zhao J, Xu E, Wang C and Zhou X (2017) MicroRNA-140-5p regulates osteosarcoma chemoresistance by targeting HMGN5 and autophagy. Scientific Reports 7. doi: 10.1038/s41598-017-00405-3

98. Zhang Y, Cai W, Zou Y and Zhang H (2020) Knockdown of KCNQ1OT1 Inhibits Proliferation, Invasion, and Drug Resistance by Regulating miR-129-5p-Mediated LARP1 in Osteosarcoma. Biomed Res Int 2020:7698767. doi: 10.1155/2020/7698767

99. Liu Y, Gu S, Li H, Wang J, Wei C and Liu Q (2019) SNHG16 promotes osteosarcoma progression and enhances cisplatin resistance by sponging miR-16 to upregulate ATG4B expression. Biochemical and Biophysical Research Communications 518:127-133. doi: 10.1016/j.bbrc.2019.08.019

100. Keremu A, Aini A, Maimaitirexiati Y, Liang Z, Aila P, Xierela P, Tusun A, Moming H and Yusufu A (2019) Overcoming cisplatin resistance in osteosarcoma through the miR-199a-modulated inhibition of HIF-1α. Bioscience Reports 39. doi: 10.1042/bsr20170080

101. Yan H, Zhang B, Fang C and Chen L (2018) miR-340 alleviates chemoresistance of osteosarcoma cells by targeting ZEB1. Anti-Cancer Drugs 29:440-448. doi: 10.1097/cad.0000000000000614

102. Xiao Q, Yang Y, An Q and Qi Y (2017) MicroRNA-100 suppresses human osteosarcoma cell proliferation and chemo-resistance via ZNRF2. Oncotarget 8:34678-34686. doi: 10.18632/oncotarget.16149

103. Wang SN, Luo S, Liu C, Piao Z, Gou W, Wang Y, Guan W, Li Q, Zou H, Yang ZZ, Wang D, Wang Y, Xu M, Jin H and Xu CX (2017) miR-491 Inhibits Osteosarcoma Lung Metastasis and Chemoresistance by Targeting alphaB-crystallin. Mol Ther 25:2140-2149. doi: 10.1016/j.ymthe.2017.05.018

104. Zhou X, Natino D, Zhai X, Gao Z and He X (2018) MicroRNA22 inhibits the proliferation and migration, and increases the cisplatin sensitivity, of osteosarcoma cells. Mol Med Rep 17:7209-7217. doi: 10.3892/mmr.2018.8790

105. Zhang L, Yang P, Liu Q, Wang J, Yan F, Duan L and Lin F (2020) KLF8 promotes cancer stem cell-like phenotypes in osteosarcoma through miR-429-SOX2 signaling. Neoplasma 67:519-527. doi: 10.4149/neo_2020_190711N624

106. Xu M, Jin H, Xu CX, Bi WZ and Wang Y (2014) MiR-34c inhibits osteosarcoma metastasis and chemoresistance. Med Oncol 31:972. doi: 10.1007/s12032-014-0972-x

107. Duan Z, Gao Y, Shen J, Choy E, Cote G, Harmon D, Bernstein K, Lozano-Calderon S, Mankin H and Hornicek FJ (2017) miR-15b modulates multidrug resistance in human osteosarcoma in vitro and in vivo. Mol Oncol 11:151-166. doi: 10.1002/1878-0261.12015

108. Maximov VV, Akkawi R, Khawaled S, Salah Z, Jaber L, Barhoum A, Or O, Galasso M, Kurek KC, Yavin E and Aqeilan RI (2019) MiR‐16‐1‐3p and miR‐16‐2‐3p possess strong tumor suppressive and antimetastatic properties in osteosarcoma. International Journal of Cancer 145:3052-3063. doi: 10.1002/ijc.32368

109. Chen R, Li X, He B and Hu W (2017) MicroRNA-410 regulates autophagy-related gene ATG16L1 expression and enhances chemosensitivity via autophagy inhibition in osteosarcoma. Mol Med Rep 15:1326-1334. doi: 10.3892/mmr.2017.6149

110. Lin Z, Song D, Wei H, Yang X, Liu T, Yan W and Xiao J (2016) TGF-β1-induced miR-202 mediates drug resistance by inhibiting apoptosis in human osteosarcoma. Journal of cancer research and clinical oncology 142:239-246. doi: 10.1007/s00432-015-2028-9

111. Zhao G, Cai C, Yang T, Qiu X, Liao B, Li W, Ji Z, Zhao J, Zhao H, Guo M, Ma Q, Xiao C, Fan Q and Ma B (2013) MicroRNA-221 induces cell survival and cisplatin resistance through PI3K/Akt pathway in human osteosarcoma. PLoS One 8:e53906. doi: 10.1371/journal.pone.0053906

112. Zhou C, Tan W, Lv H, Gao F and Sun J (2016) Hypoxia-inducible microRNA-488 regulates apoptosis by targeting Bim in osteosarcoma. Cell Oncol (Dordr) 39:463-471. doi: 10.1007/s13402-016-0288-2

113. Wang Y, Wu Y, Cai A, Ma C, Cai S, Wang H, Que Y, Xu S, Xu T and Hu Y (2020) Cisplatin inhibits the proliferation of Saos-2 osteosarcoma cells via the miR-376c/TGFA pathway. Bosnian Journal of Basic Medical Sciences. doi: 10.17305/bjbms.2020.4485

114. Yang D, Xu T, Fan L, Liu K and Li G (2020) microRNA-216b enhances cisplatin-induced apoptosis in osteosarcoma MG63 and SaOS-2 cells by binding to JMJD2C and regulating the HIF1α/HES1 signaling axis. Journal of Experimental & Clinical Cancer Research 39. doi: 10.1186/s13046-020-01670-3

115. Xu W, Li Z, Zhu X, Xu R and Xu Y (2018) miR-29 Family Inhibits Resistance to Methotrexate and Promotes Cell Apoptosis by Targeting COL3A1 and MCL1 in Osteosarcoma. Med Sci Monit 24:8812-8821. doi: 10.12659/MSM.911972

116. Zhou J, Wu S, Chen Y, Zhao J, Zhang K, Wang J and Chen S (2015) microRNA-143 is associated with the survival of ALDH1+CD133+ osteosarcoma cells and the chemoresistance of osteosarcoma. Exp Biol Med (Maywood) 240:867-75. doi: 10.1177/1535370214563893

117. Liu Y, Zhu ST, Wang X, Deng J, Li WH, Zhang P and Liu BS (2016) MiR-100 Inhibits Osteosarcoma Cell Proliferation, Migration, and Invasion and Enhances Chemosensitivity by Targeting IGFIR. Technol Cancer Res Treat 15:NP40-8. doi: 10.1177/1533034615601281

118. Shao XJ, Miao MH, Xue J, Xue J, Ji XQ and Zhu H (2015) The Down-Regulation of MicroRNA-497 Contributes to Cell Growth and Cisplatin Resistance Through PI3K/Akt Pathway in Osteosarcoma. Cell Physiol Biochem 36:2051-62. doi: 10.1159/000430172

119. Xu E, Zhao J, Ma J, Wang C, Zhang C, Jiang H, Cheng J, Gao R and Zhou X (2016) miR-146b-5p promotes invasion and metastasis contributing to chemoresistance in osteosarcoma by targeting zinc and ring finger 3. Oncol Rep 35:275-83. doi: 10.3892/or.2015.4393

120. Zhou Y, Huang Z, Wu S, Zang X, Liu M and Shi J (2014) miR-33a is up-regulated in chemoresistant osteosarcoma and promotes osteosarcoma cell resistance to cisplatin by down-regulating TWIST. Journal of experimental & clinical cancer research : CR 33:12. doi: 10.1186/1756-9966-33-12

121. Liu Q, Wang Z, Zhou X, Tang M, Tan W, Sun T and Deng Y (2019) miR-342-5p inhibits osteosarcoma cell growth, migration, invasion, and sensitivity to Doxorubicin through targeting Wnt7b. Cell Cycle 18:3325-3336. doi: 10.1080/15384101.2019.1676087

122. Liu Q, Song Y, Duan X, Chang Y and Guo J (2018) MiR-92a Inhibits the Progress of Osteosarcoma Cells and Increases the Cisplatin Sensitivity by Targeting Notch1. Biomed Res Int 2018:9870693. doi: 10.1155/2018/9870693

123. Li G, Li Y and Wang Dy (2021) Overexpression of miR‐329‐3p sensitizes osteosarcoma cells to cisplatin through suppression of glucose metabolism by targeting LDHA. Cell Biology International 45:766-774. doi: 10.1002/cbin.11476

124. Tang Q, Yuan Q, Li H, Wang W, Xie G, Zhu K and Li D (2018) miR-223/Hsp70/JNK/JUN/miR-223 feedback loop modulates the chemoresistance of osteosarcoma to cisplatin. Biochem Biophys Res Commun 497:827-834. doi: 10.1016/j.bbrc.2018.02.091

125. Xu M, Jin H, Xu C-X, Sun B, Mao Z, Bi W-Z and Wang Y (2014) miR-382 inhibits tumor growth and enhance chemosensitivity in osteosarcoma. Oncotarget 5:9472-9483.

126. Liu XG, Xu J, Li F, Li MJ and Hu T (2018) Down-regulation of miR-377 contributes to cisplatin resistance by targeting XIAP in osteosarcoma. European review for medical and pharmacological sciences 22:1249-1257. doi: 10.26355/eurrev_201803_14465

127. Liu Y, Zhu S-T, Wang X, Deng J, Li W-H, Zhang P and Liu B-S (2017) MiR-200c regulates tumor growth and chemosensitivity to cisplatin in osteosarcoma by targeting AKT2. Scientific Reports 7. doi: 10.1038/s41598-017-14088-3

128. Zhu Z, Tang J, Wang J, Duan G, Zhou L and Zhou X (2016) MiR-138 Acts as a Tumor Suppressor by Targeting EZH2 and Enhances Cisplatin-Induced Apoptosis in Osteosarcoma Cells. PLoS One 11:e0150026. doi: 10.1371/journal.pone.0150026

129. Long X and Lin XJ (2019) P65-mediated miR-590 inhibition modulates the chemoresistance of osteosarcoma to doxorubicin through targeting wild-type p53-induced phosphatase 1. J Cell Biochem 120:5652-5665. doi: 10.1002/jcb.27849

130. Li Y, Zhao C, Yu Z, Chen J, She X, Li P, Liu C, Zhang Y, Feng J, Fu H, Wang B, Kuang L, Li L, Lv G and Wu M (2016) Low expression of miR-381 is a favorite prognosis factor and enhances the chemosensitivity of osteosarcoma. Oncotarget 7:68585-68596. doi: 10.18632/oncotarget.11861

131. Wang GC, He QY, Tong DK, Wang CF, Liu K, Ding C, Ji F and Zhang H (2016) MiR-367 negatively regulates apoptosis induced by adriamycin in osteosarcoma cells by targeting KLF4. J Bone Oncol 5:51-6. doi: 10.1016/j.jbo.2016.02.002

132. Li M and Ma W (2021) miR-26a Reverses Multidrug Resistance in Osteosarcoma by Targeting MCL1. Front Cell Dev Biol 9:645381. doi: 10.3389/fcell.2021.645381

133. Jin C, Feng Y, Ni Y and Shan Z (2017) MicroRNA-610 suppresses osteosarcoma oncogenicity via targeting TWIST1 expression. Oncotarget 8:56174-56184. doi: 10.18632/oncotarget.17045

134. Li L, Kong Xa, Zang M, Hu B, Fang X, Gui B and Hu Y (2020) <p>MicroRNA-584 Impairs Cellular Proliferation and Sensitizes Osteosarcoma Cells to Cisplatin and Taxanes by Targeting CCN2</p>. Cancer Management and Research Volume 12:2577-2587. doi: 10.2147/cmar.S246545

135. Chen X, Lv C, Zhu X, Lin W, Wang L, Huang Z, Yang S and Sun J (2018) MicroRNA‑504 modulates osteosarcoma cell chemoresistance to cisplatin by targeting p53. Oncology Letters. doi: 10.3892/ol.2018.9749

136. Zhou S, Xiong M, Dai G, Yu L, Zhang Z, Chen J and Guo W (2018) MicroRNA-192-5p suppresses the initiation and progression of osteosarcoma by targeting USP1. Oncol Lett 15:6947-6956. doi: 10.3892/ol.2018.8180

137. Ling Z, Fan G, Yao D, Zhao J, Zhou Y, Feng J, Zhou G and Chen Y (2020) MicroRNA-150 functions as a tumor suppressor and sensitizes osteosarcoma to doxorubicin-induced apoptosis by targeting RUNX2. Exp Ther Med 19:481-488. doi: 10.3892/etm.2019.8231

138. Sun Y, He N, Dong Y and Jiang C (2016) MiR-24-BIM-Smac/DIABLO axis controls the sensitivity to doxorubicin treatment in osteosarcoma. Scientific Reports 6. doi: 10.1038/srep34238

139. Zhi W, Feng Q and Mingzhu Z (2022) MiR-140 targets Wnt1 to inhibit the proliferation and enhance drug sensitivity in osteosarcoma cells. Cell Mol Biol (Noisy-le-grand) 68:140-146. doi: 10.14715/cmb/2022.68.1.18

140. Zhou X, Wei P, Wang X, Zhang J and Shi Y (2021) miR-141-3p promotes the cisplatin sensitivity of osteosarcoma cell through targeting the Glutaminase (GLS)-mediated glutamine metabolism. Curr Mol Med. doi: 10.2174/1566524021666211004112055

141. Liang W, Li C, Li M, Wang D and Zhong Z (2019) <p>MicroRNA-765 sensitizes osteosarcoma cells to cisplatin via downregulating APE1 expression</p>. OncoTargets and Therapy Volume 12:7203-7214. doi: 10.2147/ott.S194800

142. Gao S, Wang K and Wang X (2020) miR-375 targeting autophagy-related 2B (ATG2B) suppresses autophagy and tumorigenesis in cisplatin-resistant osteosarcoma cells. Neoplasma 67:724-734. doi: 10.4149/neo_2020_190423N366

143. Wang J, Zhang Z, Qiu C and Wang J (2021) MicroRNA‐519d‐3p antagonizes osteosarcoma resistance against cisplatin by targeting PD‐L1. Molecular Carcinogenesis. doi: 10.1002/mc.23370

144. Wang P, Huang Y, Xia X, Han J, Zhang L and Zhao W (2022) Pleckstrin homology-like domain family A, member 3, a miR-19a-3p-regulated gene, suppresses tumor growth in osteosarcoma by downregulating the Akt pathway. Bioengineered 13:3993-4009. doi: 10.1080/21655979.2022.2031404

145. Zhan H, Xiao J, Wang P, Mo F, Li K, Guo F, Yu X, Liu X, Zhang B, Dai M and Liu H (2022) Exosomal CTCF Confers Cisplatin Resistance in Osteosarcoma by Promoting Autophagy via the IGF2-AS/miR-579-3p/MSH6 Axis. J Oncol 2022:9390611. doi: 10.1155/2022/9390611

146. Zhang Z, Zhou Q, Luo F, Zhou R, Xu J, Xiao J, Dai F and Song L (2021) Circular RNA circ-CHI3L1.2 modulates cisplatin resistance of osteosarcoma cells via the miR-340-5p/LPAATβ axis. Human Cell 34:1558-1568. doi: 10.1007/s13577-021-00564-6

147. Zhang J, Ma X, Zhou R and Zhou Y (2020) <p>TRPS1 and YAP1 Regulate Cell Proliferation and Drug Resistance of Osteosarcoma via Competitively Binding to the Target of circTADA2A – miR-129-5p</p>. OncoTargets and Therapy Volume 13:12397-12407. doi: 10.2147/ott.S276953

148. Feng ZH, Zheng L, Yao T, Tao SY, Wei XA, Zheng ZY, Zheng BJ, Zhang XY, Huang B, Liu JH, Chen YL, Shan Z, Yuan PT, Wang CG, Chen J, Shen SY and Zhao FD (2021) EIF4A3-induced circular RNA PRKAR1B promotes osteosarcoma progression by miR-361-3p-mediated induction of FZD4 expression. Cell Death Dis 12:1025. doi: 10.1038/s41419-021-04339-7

149. Dong L and Qu F (2020) CircUBAP2 promotes SEMA6D expression to enhance the cisplatin resistance in osteosarcoma through sponging miR-506-3p by activating Wnt/β-catenin signaling pathway. Journal of Molecular Histology 51:329-340. doi: 10.1007/s10735-020-09883-8

150. Wei W, Ji L, Duan W and Zhu J (2021) Circular RNA circ_0081001 knockdown enhances methotrexate sensitivity in osteosarcoma cells by regulating miR-494-3p/TGM2 axis. J Orthop Surg Res 16:50. doi: 10.1186/s13018-020-02169-5

151. Hu Y, Gu J, Shen H, Shao T, Li S, Wang W and Yu Z (2019) Circular RNA LARP4 correlates with decreased Enneking stage, better histological response, and prolonged survival profiles, and it elevates chemosensitivity to cisplatin and doxorubicin via sponging microRNA‐424 in osteosarcoma. Journal of Clinical Laboratory Analysis 34. doi: 10.1002/jcla.23045

152. Li X, Liu Y, Zhang X, Shen J, Xu R, Liu Y and Yu X (2020) Circular RNA hsa_circ_0000073 contributes to osteosarcoma cell proliferation, migration, invasion and methotrexate resistance by sponging miR-145-5p and miR-151-3p and upregulating NRAS. Aging 12:14157-14173. doi: 10.18632/aging.103423

153. Li D, Huang Y, Wang G and Zhao F (2021) Circular RNA circPVT1 Contributes to Doxorubicin (DXR) Resistance of Osteosarcoma Cells by Regulating TRIAP1 via miR-137. BioMed Research International 2021:1-19. doi: 10.1155/2021/7463867

154. Kun-Peng Z, Xiao-Long M and Chun-Lin Z (2018) Overexpressed circPVT1, a potential new circular RNA biomarker, contributes to doxorubicin and cisplatin resistance of osteosarcoma cells by regulating ABCB1. Int J Biol Sci 14:321-330. doi: 10.7150/ijbs.24360

155. Wang B, Yan L, Shi W, Xie H, Chen R, Shao Y and Liang W (2022) CircRNA PVT1 promotes proliferation and chemoresistance of osteosarcoma cells via the miR-24-3p/KLF8 axis. Int J Clin Oncol 27:811-822. doi: 10.1007/s10147-022-02122-y

156. Pan Y, Lin Y and Mi C (2021) Cisplatin-resistant osteosarcoma cell-derived exosomes confer cisplatin resistance to recipient cells in an exosomal circ_103801-dependent manner. Cell Biol Int 45:858-868. doi: 10.1002/cbin.11532

157. Zhang H, Yan J, Lang X and Zhuang Y (2018) Expression of circ_001569 is upregulated in osteosarcoma and promotes cell proliferation and cisplatin resistance by activating the Wnt/beta-catenin signaling pathway. Oncol Lett 16:5856-5862. doi: 10.3892/ol.2018.9410

158. Xie C, Liang G, Xu Y and Lin E (2020) <p>Circular RNA hsa_circ_0003496 Contributes to Tumorigenesis and Chemoresistance in Osteosarcoma Through Targeting (microRNA) miR-370/Krüppel-Like Factor 12 Axis</p>. Cancer Management and Research Volume 12:8229-8240. doi: 10.2147/cmar.S253969

159. Lin B, Nan J, Lu K, Zong Y and Fan W (2022) Hsa_circ_0001982 promotes the proliferation, invasion, and multidrug resistance of osteosarcoma cells. J Clin Lab Anal 36. doi: 10.1002/jcla.24493

160. Ma XL, Zhan TC, Hu JP, Zhang CL and Zhu KP (2021) Doxorubicin-induced novel circRNA_0004674 facilitates osteosarcoma progression and chemoresistance by upregulating MCL1 through miR-142-5p. Cell Death Discov 7:309. doi: 10.1038/s41420-021-00694-8

161. Yuan J, Liu Y, Zhang Q, Ren Z, Li G and Tian R (2021) CircPRDM2 Contributes to Doxorubicin Resistance of Osteosarcoma by Elevating EZH2 via Sponging miR-760. Cancer Manag Res 13:4433-4445. doi: 10.2147/CMAR.S295147

162. Wei W, Ji L, Duan W and Zhu J (2020) CircSAMD4A contributes to cell doxorubicin resistance in osteosarcoma by regulating the miR-218-5p/KLF8 axis. Open Life Sciences 15:848-859. doi: 10.1515/biol-2020-0079

163. Zhou W, Liu Y and Wu X (2021) Down-regulation of circITCH promotes osteosarcoma development and resistance to doxorubicin via the miR-524/RASSF6 axis. J Gene Med 23:e3373. doi: 10.1002/jgm.3373

164. Bai Y, Li Y, Bai J and Zhang Y (2021) Hsa_circ_0004674 promotes osteosarcoma doxorubicin resistance by regulating the miR-342-3p/FBN1 axis. Journal of Orthopaedic Surgery and Research 16. doi: 10.1186/s13018-021-02631-y

165. Li S, Liu F, Zheng K, Wang W, Qiu E, Pei Y, Wang S, Zhang J and Zhang X (2021) CircDOCK1 promotes the tumorigenesis and cisplatin resistance of osteogenic sarcoma via the miR-339-3p/IGF1R axis. Mol Cancer 20:161. doi: 10.1186/s12943-021-01453-0

166. Tang J, Duan G, Wang Y, Wang B, Li W and Zhu Z (2022) Circular RNA_ANKIB1 accelerates chemo-resistance of osteosarcoma via binding microRNA-26b-5p and modulating enhancer of zeste homolog 2. Bioengineered 13:7351-7366. doi: 10.1080/21655979.2022.2037869

167. Jacques C, Calleja LR, Baud'huin M, Quillard T, Heymann D, Lamoureux F and Ory B (2016) miRNA-193a-5p repression of p73 controls Cisplatin chemoresistance in primary bone tumors. Oncotarget 7:54503-54514. doi: 10.18632/oncotarget.10950

168. Nakatani F, Ferracin M, Manara MC, Ventura S, del Monaco V, Ferrari S, Alberghini M, Grilli A, Knuutila S, Schaefer K-L, Mattia G, Negrini M, Picci P, Serra M and Scotlandi K (2012) miR-34a predicts survival of Ewing's sarcoma patients and directly influences cell chemo-sensitivity and malignancy. The Journal of Pathology 226:796-805. doi: 10.1002/path.3007

169. Robin TP, Smith A, McKinsey E, Reaves L, Jedlicka P and Ford HL (2012) EWS/FLI1 Regulates EYA3 in Ewing Sarcoma via Modulation of miRNA-708, Resulting in Increased Cell Survival and Chemoresistance. Molecular Cancer Research 10:1098-1108. doi: 10.1158/1541-7786.Mcr-12-0086

170. Iida K, Fukushi J-i, Matsumoto Y, Oda Y, Takahashi Y, Fujiwara T, Fujiwara-Okada Y, Hatano M, Nabashima A, Kamura S and Iwamoto Y (2013) miR-125b develops chemoresistance in Ewing sarcoma/primitive neuroectodermal tumor. Cancer Cell International 13. doi: 10.1186/1475-2867-13-21

171. Zhu Z, Wang CP, Zhang YF and Nie L (2014) MicroRNA-100 resensitizes resistant chondrosarcoma cells to cisplatin through direct targeting of mTOR. Asian Pac J Cancer Prev 15:917-23. doi: 10.7314/apjcp.2014.15.2.917

172. Huang K, Chen J, Yang MS, Tang YJ and Pan F (2017) Inhibition of Src by microRNA-23b increases the cisplatin sensitivity of chondrosarcoma cells. Cancer Biomark 18:231-239. doi: 10.3233/CBM-160102

173. Tang XY, Zheng W, Ding M, Guo KJ, Yuan F, Feng H, Deng B, Sun W, Hou Y and Gao L (2016) miR-125b acts as a tumor suppressor in chondrosarcoma cells by the sensitization to doxorubicin through direct targeting the ErbB2-regulated glucose metabolism. Drug Des Devel Ther 10:571-83. doi: 10.2147/DDDT.S90530

174. Bharathy N, Berlow NE, Wang E, Abraham J, Settelmeyer TP, Hooper JE, Svalina MN, Ishikawa Y, Zientek K, Bajwa Z, Goros MW, Hernandez BS, Wolff JE, Rudek MA, Xu L, Anders NM, Pal R, Harrold AP, Davies AM, Ashok A, Bushby D, Mancini M, Noakes C, Goodwin NC, Ordentlich P, Keck J, Hawkins DS, Rudzinski ER, Chatterjee B, Bachinger HP, Barr FG, Liddle J, Garcia BA, Mansoor A, Perkins TJ, Vakoc CR, Michalek JE and Keller C (2018) The HDAC3-SMARCA4-miR-27a axis promotes expression of the PAX3:FOXO1 fusion oncogene in rhabdomyosarcoma. Sci Signal 11. doi: 10.1126/scisignal.aau7632

175. Minami Y, Kohsaka S, Tsuda M, Yachi K, Hatori N, Tanino M, Kimura T, Nishihara H, Minami A, Iwasaki N and Tanaka S (2014) SS

18‐

SSX

‐regulated miR‐17 promotes tumor growth of synovial sarcoma by inhibiting p21

WAF

1/

CIP

1. Cancer Science 105:1152-1159. doi: 10.1111/cas.12479

176. Xu Y, Cheng M, Mi L, Qiu Y, Hao W and Li L (2018) Mir-22-3p Enhances the Chemosensitivity of Gastrointestinal Stromal Tumor Cell Lines to Cisplatin through PTEN/PI3K/Akt Pathway. Iranian journal of allergy, asthma, and immunology 17:318-325. doi: 10.18502/ijaai.v17i4.91

177. Zhang Z, Sun C, Li C, Jiao X, Griffin BB, Dongol S, Wu H, Zhang C, Cao W, Dong R, Yang X, Zhang Q and Kong B (2020) Upregulated MELK Leads to Doxorubicin Chemoresistance and M2 Macrophage Polarization via the miR-34a/JAK2/STAT3 Pathway in Uterine Leiomyosarcoma. Front Oncol 10:453. doi: 10.3389/fonc.2020.00453

178. Jain N, Das B and Mallick B (2022) miR-197-5p increases Doxorubicin-mediated anticancer cytotoxicity of HT1080 fibrosarcoma cells by decreasing drug efflux. DNA Repair (Amst) 109:103259. doi: 10.1016/j.dnarep.2021.103259

179. Li D, Zhao K, Zhao Z, Jiang B, Gong X, Zhang Y, Guo Y, Xiao H, Wang Y, Liu H, Yi C and Gu W (2021) High Expression MicroRNA-206 Inhibits the Growth of Tumor Cells in Human Malignant Fibrous Histiocytoma. Front Cell Dev Biol 9:751833. doi: 10.3389/fcell.2021.751833

180. Wang JH, Zeng Z, Sun J, Chen Y and Gao X (2021) A novel small-molecule antagonist enhances the sensitivity of osteosarcoma to cabozantinib in vitro and in vivo by targeting DNMT-1 correlated with disease severity in human patients. Pharmacol Res 173:105869. doi: 10.1016/j.phrs.2021.105869

181. Wang L, En H, Yang L, Zhang Y, Sun B and Gao J (2019) miR-596 suppresses the expression of Survivin and enhances the sensitivity of osteosarcoma cells to the molecular targeting agent anlotinib. Onco Targets Ther 12:6825-6838. doi: 10.2147/OTT.S215145

182. Wang T, Wang D, Zhang L, Yang P, Wang J, Liu Q, Yan F and Lin F (2019) The TGFbeta-miR-499a-SHKBP1 pathway induces resistance to EGFR inhibitors in osteosarcoma cancer stem cell-like cells. J Exp Clin Cancer Res 38:226. doi: 10.1186/s13046-019-1195-y

183. Cao K, Li M, Miao J, Lu X, Kang X, Zhu H, Du S, Li X, Zhang Q, Guan W, Dong Y and Xia X (2018) CCDC26 knockdown enhances resistance of gastrointestinal stromal tumor cells to imatinib by interacting with c-KIT. American journal of translational research 10:274-282.

184. Yan J, Chen D, Chen X, Sun X, Dong Q, Hu C, Zhou F and Chen W (2019) Downregulation of lncRNA CCDC26 contributes to imatinib resistance in human gastrointestinal stromal tumors through IGF-1R upregulation. Braz J Med Biol Res 52:e8399. doi: 10.1590/1414-431x20198399

185. Zhang J, Chen K, Tang Y, Luan X, Zheng X, Lu X, Mao J, Hu L, Zhang S, Zhang X and Chen W (2021) LncRNA-HOTAIR activates autophagy and promotes the imatinib resistance of gastrointestinal stromal tumor cells through a mechanism involving the miR-130a/ATG2B pathway. Cell Death Dis 12:367. doi: 10.1038/s41419-021-03650-7

186. Shao Y, Lian S, Zheng J, Tong H, Wang J, Xu J, Liu W, Hu G, Zhang Y and He J (2021) RP11-616M22.7 recapitulates imatinib resistance in gastrointestinal stromal tumor. Mol Ther Nucleic Acids 25:264-276. doi: 10.1016/j.omtn.2021.05.017

187. Fan R, Zhong J, Zheng S, Wang Z, Xu Y, Li S, Zhou J and Yuan F (2015) microRNA-218 increase the sensitivity of gastrointestinal stromal tumor to imatinib through PI3K/AKT pathway. Clin Exp Med 15:137-44. doi: 10.1007/s10238-014-0280-y

188. Chen W, Li Z, Liu H, Jiang S, Wang G, Sun L, Li J, Wang X, Yu S, Huang J and Dong Y (2020) MicroRNA-30a targets BECLIN-1 to inactivate autophagy and sensitizes gastrointestinal stromal tumor cells to imatinib. Cell Death & Disease 11. doi: 10.1038/s41419-020-2390-7

189. Shi Y, Gao X, Hu Q, Li X, Xu J, Lu S, Liu Y, Xu C, Jiang D, Lin J, Xue A, Tan Y, Shen K and Hou Y (2016) PIK3C2A is a gene-specific target of microRNA-518a-5p in imatinib mesylate-resistant gastrointestinal stromal tumor. Laboratory Investigation 96:652-660. doi: 10.1038/labinvest.2015.157

190. Huang WK, Akcakaya P, Gangaev A, Lee L, Zeljic K, Hajeri P, Berglund E, Ghaderi M, Ahlen J, Branstrom R, Larsson C and Lui WO (2018) miR-125a-5p regulation increases phosphorylation of FAK that contributes to imatinib resistance in gastrointestinal stromal tumors. Exp Cell Res 371:287-296. doi: 10.1016/j.yexcr.2018.08.028

191. Akcakaya P, Caramuta S, Ahlen J, Ghaderi M, Berglund E, Ostman A, Branstrom R, Larsson C and Lui WO (2014) microRNA expression signatures of gastrointestinal stromal tumours: associations with imatinib resistance and patient outcome. Br J Cancer 111:2091-102. doi: 10.1038/bjc.2014.548

192. Cao CL, Niu HJ, Kang SP, Cong CL and Kang SR (2016) miRNA-21 sensitizes gastrointestinal stromal tumors (GISTs) cells to Imatinib via targeting B-cell lymphoma 2 (Bcl-2). European review for medical and pharmacological sciences 20:3574-3581.

193. Shiozawa K, Shuting J, Yoshioka Y, Ochiya T and Kondo T (2018) Extracellular vesicle-encapsulated microRNA-761 enhances pazopanib resistance in synovial sarcoma. Biochem Biophys Res Commun 495:1322-1327. doi: 10.1016/j.bbrc.2017.11.164

194. Pang B and Hao Y (2021) Integrated Analysis of the Transcriptome Profile Reveals the Potential Roles Played by Long Noncoding RNAs in Immunotherapy for Sarcoma. Frontiers in oncology 11:690486. doi: 10.3389/fonc.2021.690486

195. He P, Xu YQ, Wang ZJ and Sheng B (2020) LncRNA LINC00210 regulated radiosensitivity of osteosarcoma cells via miR-342-3p/GFRA1 axis. J Clin Lab Anal 34:e23540. doi: 10.1002/jcla.23540

196. Yang Z, Wa Q-D, Lu C, Pan W, Lu Z-Μ and Ao J (2018) miR‑328‑3p enhances the radiosensitivity of osteosarcoma and regulates apoptosis and cell viability via H2AX. Oncology reports 39:545-553. doi: 10.3892/or.2017.6112

197. Li Y, Song X, Liu Z, Li Q, Huang M, Su B, Mao Y, Wang Y, Mo W and Chen H (2019) Upregulation of miR-214 Induced Radioresistance of Osteosarcoma by Targeting PHLDA2 via PI3K/Akt Signaling. Front Oncol 9:298. doi: 10.3389/fonc.2019.00298

198. Dai N, Qing Y, Cun Y, Zhong Z, Li C, Zhang S, Shan J, Yang X, Dai X, Cheng Y, Xiao H, Xu C, Li M and Wang D (2018) miR-513a-5p regulates radiosensitivity of osteosarcoma by targeting human apurinic/apyrimidinic endonuclease. Oncotarget 9:25414-25426. doi: 10.18632/oncotarget.11003

199. Vares G, Ahire V, Sunada S, Ho Kim E, Sai S, Chevalier F, Romeo P-H, Yamamoto T, Nakajima T and Saintigny Y (2020) A multimodal treatment of carbon ions irradiation, miRNA-34 and mTOR inhibitor specifically control high-grade chondrosarcoma cancer stem cells. Radiotherapy and Oncology 150:253-261. doi: 10.1016/j.radonc.2020.07.034

200. Lee YY, Yang YP, Huang MC, Wang ML, Yen SH, Huang PI, Chen YW, Chiou SH, Lan YT, Ma HI, Shih YH and Chen MT (2014) MicroRNA142-3p promotes tumor-initiating and radioresistant properties in malignant pediatric brain tumors. Cell Transplant 23:669-90. doi: 10.3727/096368914X678364

201. Polvani S, Martignano F, Scoccianti G, Pasqui A, Palomba AR, Conticello S, Galli A, Palchetti I, Caporalini C, Antonuzzo L, Campanacci DA and Pillozzi S (2022) Growth arrest-specific 5 lncRNA as a valuable biomarker of chemoresistance in osteosarcoma. Anticancer Drugs 33:278-285. doi: 10.1097/CAD.0000000000001263

202. Zhu K-P, Zhang C-L, Shen G-Q and Zhu Z-S (2015) Long noncoding RNA expression profiles of the doxorubicin-resistant human osteosarcoma cell line MG63/DXR and its parental cell line MG63 as ascertained by microarray analysis. International journal of clinical and experimental pathology 8:8754-8773.

203. Yuan J, Chen L, Chen X, Sun W and Zhou X (2012) Identification of serum microRNA-21 as a biomarker for chemosensitivity and prognosis in human osteosarcoma. The Journal of international medical research 40:2090-2097.

204. Luo Z, Liu M, Zhang H and Xia Y (2016) Association of circulating miR-125b and survival in patients with osteosarcoma–A single center experience. Journal of Bone Oncology 5:167-172. doi: 10.1016/j.jbo.2016.06.002

205. Han J, Ye Z, Kong Q, Wu F, Wang G and Wang K (2020) Circular RNA expression pattern in cisplatin-resistant osteosarcoma cells. Transl Cancer Res 9:262-270. doi: 10.21037/tcr.2019.12.80

206. Kun-Peng Z, Xiao-Long M, Lei Z, Chun-Lin Z, Jian-Ping H and Tai-Cheng Z (2018) Screening circular RNA related to chemotherapeutic resistance in osteosarcoma by RNA sequencing. Epigenomics 10:1327-1346. doi: 10.2217/epi-2018-0023

207. Yamada H, Takahashi M, Watanuki M, Watanabe M, Hiraide S, Saijo K, Komine K and Ishioka C (2021) lncRNA HAR1B has potential to be a predictive marker for pazopanib therapy in patients with sarcoma. Oncology Letters 21. doi: 10.3892/ol.2021.12716

208. Yan J, Chen D, Chen X, Sun X, Dong Q, Du Z and Wang T (2018) Identification of imatinib‑resistant long non‑coding RNAs in gastrointestinal stromal tumors. Oncology Letters. doi: 10.3892/ol.2018.9821

209. Amirnasr, Gits, van K, Smid, Vriends, Rutkowski, Sciot, Schöffski, Debiec R, Sleijfer and Wiemer (2019) Molecular Comparison of Imatinib-Naïve and Resistant Gastrointestinal Stromal Tumors: Differentially Expressed microRNAs and mRNAs. Cancers 11. doi: 10.3390/cancers11060882

210. Kou Y, Yang R and Wang Q (2018) Serum miR-518e-5p is a potential biomarker for secondary imatinib-resistant gastrointestinal stromal tumor. Journal of biosciences 43:1015-1023.

211. Gao X, Shen K, Wang C, Ling J, Wang H, Fang Y, Shi Y, Hou Y, Qin J, Sun Y and Qin X (2014) MiR-320a downregulation is associated with imatinib resistance in gastrointestinal stromal tumors. Acta Biochim Biophys Sin (Shanghai) 46:72-5. doi: 10.1093/abbs/gmt118

212. Zhang Z, Jiang Ny, Guan Ry, Zhu Yk, Jiang Fq and Piao D (2018) Identification of critical microRNAs in gastrointestinal stromal tumor patients treated with Imatinib. Neoplasma 65:683-692. doi: 10.4149/neo_2018_170906N575
