# Supplementary material for: Non-coding RNAs in drug and radiation resistance of bone and soft-tissue sarcoma: a systematic review
Source: eLife. 2022 Nov 3;11:e79655. doi: 10.7554/eLife.79655 (PMC9691005; doi:10.7554/eLife.79655)
Supplement: Supplementary file 3. [file elife-79655-supp3.docx]

**Supplementary Table 3. References of studies excluded in the systematic review (N =715).**

| **Records non relevant to this review (N =571)** |
| --- |
| 1: Ross NT, Lohmann F, Carbonneau S, Fazal A, Weihofen WA, Gleim S, Salcius M,Sigoillot F, Henault M, Carl SH, Rodríguez-Molina JB, Miller HR, Brittain SM,Murphy J, Zambrowski M, Boynton G, Wang Y, Chen A, Molind GJ, Wilbertz JH,Artus-Revel CG, Jia M, Akinjiyan FA, Turner J, Knehr J, Carbone W, Schuierer S,Reece-Hoyes JS, Xie K, Saran C, Williams ET, Roma G, Spencer M, Jenkins J,George EL, Thomas JR, Michaud G, Schirle M, Tallarico J, Passmore LA, Chao JA,Beckwith REJ. CPSF3-dependent pre-mRNA processing as a druggable node in AML andEwing's sarcoma. Nat Chem Biol. 2020 Jan;16(1):50-59. doi:10.1038/s41589-019-0424-1. Epub 2019 Dec 9. Erratum in: Nat Chem Biol. 2020Apr;16(4):479. PMID: 31819276; PMCID: PMC7116157.  2: Shen Y, Xu J, Pan X, Zhang Y, Weng Y, Zhou D, He S. LncRNA KCNQ1OT1 spongesmiR-34c-5p to promote osteosarcoma growth via ALDOA enhanced aerobic glycolysis.Cell Death Dis. 2020 Apr 24;11(4):278. doi: 10.1038/s41419-020-2485-1. PMID:32332718; PMCID: PMC7181648.  3: Toptan T, Abere B, Nalesnik MA, Swerdlow SH, Ranganathan S, Lee N, Shair KH,Moore PS, Chang Y. Circular DNA tumor viruses make circular RNAs. Proc Natl AcadSci U S A. 2018 Sep 11;115(37):E8737-E8745. doi: 10.1073/pnas.1811728115. Epub2018 Aug 27. PMID: 30150410; PMCID: PMC6140489.  4: Xie B, Li Y, Zhao R, Xu Y, Wu Y, Wang J, Xia D, Han W, Chen D. Identificationof Key Genes and miRNAs in Osteosarcoma Patients with Chemoresistance byBioinformatics Analysis. Biomed Res Int. 2018 Apr 22;2018:4761064. doi:10.1155/2018/4761064. PMID: 29850522; PMCID: PMC5937522.  5: Huber V, Vallacchi V, Fleming V, Hu X, Cova A, Dugo M, Shahaj E, Sulsenti R,Vergani E, Filipazzi P, De Laurentiis A, Lalli L, Di Guardo L, Patuzzo R,Vergani B, Casiraghi E, Cossa M, Gualeni A, Bollati V, Arienti F, De Braud F,Mariani L, Villa A, Altevogt P, Umansky V, Rodolfo M, Rivoltini L. Tumor-derivedmicroRNAs induce myeloid suppressor cells and predict immunotherapy resistancein melanoma. J Clin Invest. 2018 Dec 3;128(12):5505-5516. doi: 10.1172/JCI98060.Epub 2018 Nov 5. PMID: 30260323; PMCID: PMC6264733.  6: Wang X, Han Y, Li J, Hong D, Xue Z, Huang H, Du Z, Hou Y, Li H, Li H, Liao H,Xie X, Ding C. Multi-omics analysis of copy number variations of RNA regulatory  genes in soft tissue sarcoma. Life Sci. 2021 Jan 15;265:118734. doi:10.1016/j.lfs.2020.118734. Epub 2020 Nov 7. PMID: 33166590.  7: Antonescu CR, Agaram NP, Sung YS, Zhang L, Swanson D, Dickson BC. A DistinctMalignant Epithelioid Neoplasm With GLI1 Gene Rearrangements, Frequent S100Protein Expression, and Metastatic Potential: Expanding the Spectrum ofPathologic Entities With ACTB/MALAT1/PTCH1-GLI1 Fusions. Am J Surg Pathol. 2018Apr;42(4):553-560. doi: 10.1097/PAS.0000000000001010. PMID: 29309307; PMCID:PMC5844813.  8: Li S, Pei Y, Wang W, Liu F, Zheng K, Zhang X. Extracellular nanovesicles-transmitted circular RNA has_circ_0000190 suppresses osteosarcoma progression. JCell Mol Med. 2020 Feb;24(3):2202-2214. doi: 10.1111/jcmm.14877. Epub 2020 Jan10. PMID: 31923350; PMCID: PMC7011131.  9: Yoon JW, Lamm M, Chandler C, Iannaccone P, Walterhouse D. Up-regulation ofGLI1 in vincristine-resistant rhabdomyosarcoma and Ewing sarcoma. BMC Cancer.2020 Jun 3;20(1):511. doi: 10.1186/s12885-020-06985-0. PMID: 32493277; PMCID:PMC7310145.  10: Liang Z, Qin Z, Riker AI, Xi Y. CRISPR/Cas9 ablating viral microRNA promoteslytic reactivation of Kaposi's sarcoma-associated herpesvirus. Biochem BiophysRes Commun. 2020 Dec 17;533(4):1400-1405. doi: 10.1016/j.bbrc.2020.10.030. Epub2020 Oct 19. PMID: 33092788; PMCID: PMC7813130.  11: Godel M, Morena D, Ananthanarayanan P, Buondonno I, Ferrero G, Hattinger CM,Di Nicolantonio F, Serra M, Taulli R, Cordero F, Riganti C, Kopecka J. Small  Nucleolar RNAs Determine Resistance to Doxorubicin in Human Osteosarcoma. Int JMol Sci. 2020 Jun 24;21(12):4500. doi: 10.3390/ijms21124500. PMID: 32599901;  PMCID: PMC7349977.  12: Liu W, Liu PC, Ma K, Wang YY, Chi QB, Yan M. LncRNA DLEU2 promotes tumourgrowth by sponging miR-337-3p in human osteosarcoma. Cell Biochem Funct. 2020Oct;38(7):886-894. doi: 10.1002/cbf.3509. Epub 2020 Mar 20. PMID: 32196715.  13: Yang L, Li H, Huang A. MiR-429 and MiR-143-3p Function as Diagnostic andPrognostic Markers for Osteosarcoma. Clin Lab. 2020 Oct 1;66(10). doi:10.7754/Clin.Lab.2020.191237. PMID: 33073957.  14: Shi L, Xie C, Zhu J, Chen X. Downregulation of serum miR-194 predicts poorprognosis in osteosarcoma patients. Ann Diagn Pathol. 2020 Jun;46:151488. doi:10.1016/j.anndiagpath.2020.151488. Epub 2020 Mar 3. PMID: 32172218.  15: Palombo R, Verdile V, Paronetto MP. Poison-Exon Inclusion in DHX9 ReducesIts Expression and Sensitizes Ewing Sarcoma Cells to Chemotherapeutic Treatment.Cells. 2020 Jan 31;9(2):328. doi: 10.3390/cells9020328. PMID: 32023846; PMCID:PMC7072589.  16: Huang X, Yang W, Zhang Z, Shao Z. Dysregulated circRNAs serve as prognosticand diagnostic markers in osteosarcoma by sponging microRNA to regulate thedownstream signaling pathway. J Cell Biochem. 2020 Feb;121(2):1834-1841. doi:10.1002/jcb.29418. Epub 2019 Oct 23. PMID: 31642106.  17: Luo X, Tang J, Xuan H, Liu J, Li X. Identification and Validation of aPotent Multi-miRNA Signature for Prediction of Prognosis of OsteosarcomaPatients. Med Sci Monit. 2020 Feb 26;26:e919272. doi: 10.12659/MSM.919272. PMID:32098942; PMCID: PMC7060510.  18: Fu J, Jiang H, Wu C, Jiang Y, Xiao L, Tian Y. Overcoming cetuximabresistance in Ewing's sarcoma by inhibiting lactate dehydrogenase-A. Mol MedRep. 2016 Jul;14(1):995-1001. doi: 10.3892/mmr.2016.5290. Epub 2016 May 17.PMID: 27222229.  19: Ren X, He J, Qi L, Li S, Zhang C, Duan Z, Wang W, Tu C, Li Z. Prognostic andclinicopathologic significance of long non-coding RNA opa-interacting protein  5-antisense RNA 1 in multiple human cancers. Artif Cells Nanomed Biotechnol.2020 Dec;48(1):353-361. doi: 10.1080/21691401.2019.1709854. PMID: 31899963.  20: Abere B, Zhou H, Li J, Cao S, Toptan T, Grundhoff A, Fischer N, Moore PS,Chang Y. Merkel Cell Polyomavirus Encodes Circular RNAs (circRNAs) Enabling aDynamic circRNA/microRNA/mRNA Regulatory Network. mBio. 2020 Dec15;11(6):e03059-20. doi: 10.1128/mBio.03059-20. PMID: 33323517; PMCID:PMC7773998.  21: Vishnubalaji R, Elango R, Al-Toub M, Manikandan M, Al-Rikabi A, Harkness L,Ditzel N, Atteya M, Hamam R, Alfayez M, Aldahmash A, Kassem M, Alajez NM.Neoplastic Transformation of Human Mesenchymal Stromal Cells Mediated viaLIN28B. Sci Rep. 2019 May 30;9(1):8101. doi: 10.1038/s41598-019-44536-1. PMID:31147574; PMCID: PMC6542832.  22: Yoshikawa R, Heishima K, Ueno Y, Kawade M, Maeda Y, Yoshida K, Murakami M,Sakai H, Akao Y, Mori T. Development of synthetic microRNA-214 showing enhancedcytotoxicity and RNase resistance for treatment of canine hemangiosarcoma. VetComp Oncol. 2020 Dec;18(4):570-579. doi: 10.1111/vco.12580. Epub 2020 Apr 1.PMID: 32072720.  23: Yu B, Jiang K, Zhang J. MicroRNA-124 suppresses growth and aggressiveness ofosteosarcoma and inhibits TGF-β-mediated AKT/GSK-3β/SNAIL-1 signaling. Mol MedRep. 2018 May;17(5):6736-6744. doi: 10.3892/mmr.2018.8637. Epub 2018 Feb 27.PMID: 29488603.  24: Mercatelli N, Fortini D, Palombo R, Paronetto MP. Small molecule inhibitionof Ewing sarcoma cell growth via targeting the long non coding RNA HULC. CancerLett. 2020 Jan 28;469:111-123. doi: 10.1016/j.canlet.2019.10.026. Epub 2019 Oct19. PMID: 31639426.  25: Wei R, Thanindratarn P, Dean DC, Hornicek FJ, Guo W, Duan Z. Cyclin E1 is aprognostic biomarker and potential therapeutic target in osteosarcoma. J Orthop  Res. 2020 Sep;38(9):1952-1964. doi: 10.1002/jor.24659. Epub 2020 Mar 23. PMID:32162720.  26: Lee EY, Yu JY, Paek AR, Lee SH, Jang H, Cho SY, Kim JH, Kang HG, Yun T, OhSE, Park SY, You HJ. Targeting TJP1 attenuates cell-cell aggregation andmodulates chemosensitivity against doxorubicin in leiomyosarcoma. J Mol Med(Berl). 2020 May;98(5):761-773. doi: 10.1007/s00109-020-01909-8. Epub 2020 Apr21. PMID: 32318747.  27: Chen Y, Zhang K, Li Y, Guo R, Zhang K, Zhong G, He Q. Oestrogen-relatedreceptor alpha mediates chemotherapy resistance of osteosarcoma cells viaregulation of ABCB1. J Cell Mol Med. 2019 Mar;23(3):2115-2124. doi:10.1111/jcmm.14123. Epub 2019 Jan 4. PMID: 30609256; PMCID: PMC6378180.  28: Li MH, Wu ZY, Wang Y, Chen FZ, Liu Y. Expression of miR-29 and STAT3 inosteosarcoma and its effect on proliferation regulation of osteosarcoma cells.  Eur Rev Med Pharmacol Sci. 2019 Sep;23(17):7275-7282. doi:10.26355/eurrev_201909_18832. PMID: 31539114.  29: Dai P, He Y, Luo G, Deng J, Jiang N, Fang T, Li Y, Cheng Y. Screeningcandidate microRNA-mRNA network for predicting the response to chemoresistance  in osteosarcoma by bioinformatics analysis. J Cell Biochem. 2019Oct;120(10):16798-16810. doi: 10.1002/jcb.28938. Epub 2019 May 14. PMID:31090103.  30: Yarapureddy S, Abril J, Foote J, Kumar S, Asad O, Sharath V, Faraj J, DanielD, Dickman P, White-Collins A, Hingorani P, Sertil AR. ATF6α Activation Enhances  Survival against Chemotherapy and Serves as a Prognostic Indicator inOsteosarcoma. Neoplasia. 2019 Jun;21(6):516-532. doi: 10.1016/j.neo.2019.02.004.Epub 2019 Apr 25. PMID: 31029032; PMCID: PMC6484364.  31: Wang H, Xing D, Ren D, Feng W, Chen Y, Zhao Z, Xiao Z, Peng Z. MicroRNA‑643regulates the expression of ZEB1 and inhibits tumorigenesis in osteosarcoma. MolMed Rep. 2017 Oct;16(4):5157-5164. doi: 10.3892/mmr.2017.7273. Epub 2017 Aug 17.PMID: 28849077; PMCID: PMC5647050.  32: Asano N, Matsuzaki J, Ichikawa M, Kawauchi J, Takizawa S, Aoki Y, SakamotoH, Yoshida A, Kobayashi E, Tanzawa Y, Nakayama R, Morioka H, Matsumoto M,Nakamura M, Kondo T, Kato K, Tsuchiya N, Kawai A, Ochiya T. A serum microRNAclassifier for the diagnosis of sarcomas of various histological subtypes. NatCommun. 2019 Mar 21;10(1):1299. doi: 10.1038/s41467-019-09143-8. PMID: 30898996;PMCID: PMC6428849.  33: Vergani E, Dugo M, Cossa M, Frigerio S, Di Guardo L, Gallino G, MattavelliI, Vergani B, Lalli L, Tamborini E, Valeri B, Gargiuli C, Shahaj E, Ferrarini M,  Ferrero E, Gomez Lira M, Huber V, Vecchio MD, Sensi M, Leone BE, Santinami M,Rivoltini L, Rodolfo M, Vallacchi V. miR-146a-5p impairs melanoma resistance to  kinase inhibitors by targeting COX2 and regulating NFkB-mediated inflammatorymediators. Cell Commun Signal. 2020 Sep 23;18(1):156. doi:10.1186/s12964-020-00601-1. PMID: 32967672; PMCID: PMC7510138.  34: Du YJ, Yu QQ, Zheng XF, Wang SP. LncRNA TUG1 positively regulates osteoclastdifferentiation by targeting v-maf musculoaponeurotic fibrosarcoma oncogene  homolog B. Autoimmunity. 2020 Dec;53(8):443-449. doi:10.1080/08916934.2020.1839891. Epub 2020 Nov 4. PMID: 33146047.  35: Yao ZS, Li C, Liang D, Jiang XB, Tang JJ, Ye LQ, Yuan K, Ren H, Yang ZD, JinDX, Zhang SC, Ding JY, Tang YC, Xu JX, Chen K, Xie WX, Guo DQ, Cui JC.  Diagnostic and prognostic implications of serum miR-101 in osteosarcoma. CancerBiomark. 2018;22(1):127-133. doi: 10.3233/CBM-171103. PMID: 29630525; PMCID:  PMC6004928.  36: Ma B, Li M, Zhang L, Huang M, Lei JB, Fu GH, Liu CX, Lai QW, Chen QQ, WangYL. Upregulation of long non-coding RNA TUG1 correlates with poor prognosis anddisease status in osteosarcoma. Tumour Biol. 2016 Apr;37(4):4445-55. doi:10.1007/s13277-015-4301-6. Epub 2015 Oct 25. PMID: 26499949.  37: Oko LM, Kimball AK, Kaspar RE, Knox AN, Coleman CB, Rochford R, Chang T,Alderete B, van Dyk LF, Clambey ET. Multidimensional analysis ofGammaherpesvirus RNA expression reveals unexpected heterogeneity of geneexpression. PLoS Pathog. 2019 Jun 5;15(6):e1007849. doi:10.1371/journal.ppat.1007849. PMID: 31166996; PMCID: PMC6576797.  38: Tao SC, Huang JY, Wei ZY, Li ZX, Guo SC. EWSAT1 Acts in Concert withExosomes in Osteosarcoma Progression and Tumor-Induced Angiogenesis: The "Double  Stacking Effect". Adv Biosyst. 2020 Sep;4(9):e2000152. doi:10.1002/adbi.202000152. Epub 2020 Aug 16. PMID: 32803878.  39: Sin SH, Eason AB, Bigi R, Kim Y, Kang S, Tan K, Seltzer TA, VenkataramananR, An H, Dittmer DP. Kaposi's Sarcoma-Associated Herpesvirus Latency Locus  Renders B Cells Hyperresponsive to Secondary Infections. J Virol. 2018 Sep12;92(19):e01138-18. doi: 10.1128/JVI.01138-18. PMID: 30021906; PMCID:PMC6146794.  40: Xu N, Li Z, Yu Z, Yan F, Liu Y, Lu X, Yang W. MicroRNA-33b suppressesmigration and invasion by targeting c-Myc in osteosarcoma cells. PLoS One. 2014Dec 29;9(12):e115300. doi: 10.1371/journal.pone.0115300. PMID: 25546234; PMCID:PMC4278906.  41: Ying T, Dong JL, Yuan C, Li P, Guo Q. The lncRNAs RP1-261G23.7, RP11-69E11.4and SATB2-AS1 are a novel clinical signature for predicting recurrentosteosarcoma. Biosci Rep. 2020 Jan 31;40(1):BSR20191251. doi:10.1042/BSR20191251. PMID: 31850493; PMCID: PMC6944665.  42: Zhang RM, Tang T, Yu HM, Yao XD. LncRNA DLX6-AS1/miR-129-5p/DLK1 axisaggravates stemness of osteosarcoma through Wnt signaling. Biochem Biophys ResCommun. 2018 Dec 9;507(1-4):260-266. doi: 10.1016/j.bbrc.2018.11.019. Epub 2018Nov 13. PMID: 30442366.  43: King CA, Li X, Barbachano-Guerrero A, Bhaduri-McIntosh S. STAT3 RegulatesLytic Activation of Kaposi's Sarcoma-Associated Herpesvirus. J Virol. 2015Nov;89(22):11347-55. doi: 10.1128/JVI.02008-15. Epub 2015 Sep 2. PMID: 26339061;PMCID: PMC4645641.  44: Cai L, Lv J, Zhang Y, Li J, Wang Y, Yang H. The lncRNA HNF1A-AS1 is anegative prognostic factor and promotes tumorigenesis in osteosarcoma. J CellMol Med. 2017 Nov;21(11):2654-2662. doi: 10.1111/jcmm.12944. Epub 2017 Sep 2.PMID: 28866868; PMCID: PMC5661255.  45: Liu Y, Luo G, He D. Clinical importance of S100A9 in osteosarcomadevelopment and as a diagnostic marker and therapeutic target. Bioengineered.2019 Dec;10(1):133-141. doi: 10.1080/21655979.2019.1607709. PMID: 31055998;PMCID: PMC6527076.  46: Lu S, Liao QS, Tang L. MiR-155 affects osteosarcoma cell proliferation andinvasion through regulating NF-κB signaling pathway. Eur Rev Med Pharmacol Sci.2018 Nov;22(22):7633-7639. doi: 10.26355/eurrev_201811_16380. PMID: 30536304.  47: Chen SM, Chou WC, Hu LY, Hsiung CN, Chu HW, Huang YL, Hsu HM, Yu JC, ShenCY. The Effect of MicroRNA-124 Overexpression on Anti-Tumor Drug Sensitivity.PLoS One. 2015 Jun 26;10(6):e0128472. doi: 10.1371/journal.pone.0128472. PMID:26115122; PMCID: PMC4482746.  48: Greve B, Sheikh-Mounessi F, Kemper B, Ernst I, Götte M, Eich HT. Survivin, atarget to modulate the radiosensitivity of Ewing's sarcoma. Strahlenther Onkol.2012 Nov;188(11):1038-47. doi: 10.1007/s00066-012-0223-z. Epub 2012 Oct 10.PMID: 23053158.  49: Yuan H, Gao Y. MicroRNA-1908 is upregulated in human osteosarcoma andregulates cell proliferation and migration by repressing PTEN expression. OncolRep. 2015 Nov;34(5):2706-14. doi: 10.3892/or.2015.4242. Epub 2015 Sep 1. PMID:26328886.  50: Kim M, Jung JY, Choi S, Lee H, Morales LD, Koh JT, Kim SH, Choi YD, Choi C,Slaga TJ, Kim WJ, Kim DJ. GFRA1 promotes cisplatin-induced chemoresistance in  osteosarcoma by inducing autophagy. Autophagy. 2017 Jan 2;13(1):149-168. doi:10.1080/15548627.2016.1239676. Epub 2016 Oct 18. PMID: 27754745; PMCID:PMC5240831.  51: Jones SE, Fleuren EDG, Frankum J, Konde A, Williamson CT, Krastev DB,Pemberton HN, Campbell J, Gulati A, Elliott R, Menon M, Selfe JL, Brough R,Pettitt SJ, Niedzwiedz W, van der Graaf WTA, Shipley J, Ashworth A, Lord CJ. ATRIs a Therapeutic Target in Synovial Sarcoma. Cancer Res. 2017 Dec15;77(24):7014-7026. doi: 10.1158/0008-5472.CAN-17-2056. Epub 2017 Oct 16. PMID:29038346; PMCID: PMC6155488.  52: Shen L, Wang P, Yang J, Li X. MicroRNA-217 regulates WASF3 expression andsuppresses tumor growth and metastasis in osteosarcoma. PLoS One. 2014 Oct7;9(10):e109138. doi: 10.1371/journal.pone.0109138. PMID: 25289936; PMCID:PMC4188591.  53: Mutlu S, Mutlu H, Kirkbes S, Eroglu S, Kabukcuoglu YS, Kabukcuoglu F, DuymusTM, ISık M, Ulasli M. The expression of miR-181a-5p and miR-371b-5p inchondrosarcoma. Eur Rev Med Pharmacol Sci. 2015 Jul;19(13):2384-8. PMID:26214773.  54: Jiang K, Li S, Li L, Wang X, Gu Y, Jin Z. WNT6 is an effective marker forosteosarcoma diagnosis and prognosis. Medicine (Baltimore). 2018Nov;97(46):e13011. doi: 10.1097/MD.0000000000013011. PMID: 30431574; PMCID:PMC6257418.  55: Chen L, Cao H, Feng Y. MiR-199a suppresses prostate cancer paclitaxelresistance by targeting YES1. World J Urol. 2018 Mar;36(3):357-365. doi:10.1007/s00345-017-2143-0. Epub 2017 Dec 4. PMID: 29204706.  56: Tian X, Zhang J, Yan L, Dong JM, Guo Q. MiRNA-15a inhibits proliferation,migration and invasion by targeting TNFAIP1 in human osteosarcoma cells. Int JClin Exp Pathol. 2015 Jun 1;8(6):6442-9. PMID: 26261520; PMCID: PMC4525854.  57: Wang Z, Lei H, Sun Q. MicroRNA-141 and its associated gene FUS modulateproliferation, migration and cisplatin chemosensitivity in neuroblastoma celllines. Oncol Rep. 2016 May;35(5):2943-51. doi: 10.3892/or.2016.4640. Epub 2016Feb 26. PMID: 26936280; PMCID: PMC4811401.  58: Jamil NS, Azfer A, Worrell H, Salter DM. Functional roles of CSPG4/NG2 inchondrosarcoma. Int J Exp Pathol. 2016 Apr;97(2):178-86. doi: 10.1111/iep.12189.Epub 2016 Jun 12. PMID: 27292772; PMCID: PMC4926050.  59: Liu W, Zhao X, Zhang YJ, Fang GW, Xue Y. MicroRNA-375 as a potential serumbiomarker for the diagnosis, prognosis, and chemosensitivity prediction of  osteosarcoma. J Int Med Res. 2018 Mar;46(3):975-983. doi:10.1177/0300060517734114. Epub 2017 Nov 8. PMID: 29115164; PMCID: PMC5972241.  60: Wu DP, Yan XB, Liu LG, Tian C, Han K, Zhang H, Min DL. TopBP1 promotesmalignant progression and correlates with poor prognosis in osteosarcoma. EurRev Med Pharmacol Sci. 2017 Sep;21(18):4022-4031. PMID: 29028101.  61: Yoshida A, Fujiwara T, Uotani K, Morita T, Kiyono M, Yokoo S, Hasei J,Nakata E, Kunisada T, Ozaki T. Clinical and Functional Significance ofIntracellular and Extracellular microRNA-25-3p in Osteosarcoma. Acta MedOkayama. 2018 Apr;72(2):165-174. doi: 10.18926/AMO/55857. PMID: 29674765.  62: Urdinez J, Boro A, Mazumdar A, Arlt MJ, Muff R, Botter SM, Bode-LesniewskaB, Fuchs B, Snedeker JG, Gvozdenovic A. The miR-143/145 Cluster, a Novel  Diagnostic Biomarker in Chondrosarcoma, Acts as a Tumor Suppressor and DirectlyInhibits Fascin-1. J Bone Miner Res. 2020 Jun;35(6):1077-1091. doi:10.1002/jbmr.3976. Epub 2020 Mar 10. PMID: 32027760.  63: Fan L, Zhu C, Qiu R, Zan P, Zheng Z, Xu T, Li G. MicroRNA-661 Enhances TRAILor STS Induced Osteosarcoma Cell Apoptosis by Modulating the Expression ofCytochrome c1. Cell Physiol Biochem. 2017;41(5):1935-1946. doi:10.1159/000472380. Epub 2017 Apr 7. PMID: 28391262.  64: Liu K, Sun X, Zhang Y, Liu L, Yuan Q. MiR-598: A tumor suppressor withbiomarker significance in osteosarcoma. Life Sci. 2017 Nov 1;188:141-148. doi:10.1016/j.lfs.2017.09.003. Epub 2017 Sep 4. PMID: 28882648.  65: Kun-Peng Z, Chun-Lin Z, Xiao-Long M. Antisense lncRNA FOXF1-AS1 PromotesMigration and Invasion of Osteosarcoma Cells Through the FOXF1/MMP-2/-9 Pathway.Int J Biol Sci. 2017 Sep 5;13(9):1180-1191. doi: 10.7150/ijbs.21722. PMID:29104509; PMCID: PMC5666333.  66: Lee HJ, Yoon C, Schmidt B, Park DJ, Zhang AY, Erkizan HV, Toretsky JA,Kirsch DG, Yoon SS. Combining PARP-1 inhibition and radiation in Ewing sarcomaresults in lethal DNA damage. Mol Cancer Ther. 2013 Nov;12(11):2591-600. doi:10.1158/1535-7163.MCT-13-0338. Epub 2013 Aug 21. PMID: 23966622; PMCID:PMC3823674.  67: Yang Z, Zhang Y, Zhang X, Zhang M, Liu H, Zhang S, Qi B, Sun X. SerummicroRNA-221 functions as a potential diagnostic and prognostic marker forpatients with osteosarcoma. Biomed Pharmacother. 2015 Oct;75:153-8. doi:10.1016/j.biopha.2015.07.018. Epub 2015 Sep 28. PMID: 26422796.  68: Yang W, Liu X, Choy E, Mankin H, Hornicek FJ, Duan Z. Targeting hedgehog-GLI-2 pathway in osteosarcoma. J Orthop Res. 2013 Mar;31(3):502-9. doi:10.1002/jor.22230. Epub 2012 Sep 11. PMID: 22968906.  69: Gits CM, van Kuijk PF, de Rijck JC, Muskens N, Jonkers MB, van IJcken WF,Mathijssen RH, Verweij J, Sleijfer S, Wiemer EA. MicroRNA response to hypoxicstress in soft tissue sarcoma cells: microRNA mediated regulation of HIF3α. BMCCancer. 2014 Jun 13;14:429. doi: 10.1186/1471-2407-14-429. PMID: 24927770;PMCID: PMC4065608.  70: Du LQ, Wang Y, Wang H, Cao J, Liu Q, Fan FY. Knockdown of Rad51 expressioninduces radiation- and chemo-sensitivity in osteosarcoma cells. Med Oncol. 2011Dec;28(4):1481-7. doi: 10.1007/s12032-010-9605-1. Epub 2010 Jul 13. PMID:20625943.  71: Venkataramani V, Küffer S, Cheung KCP, Jiang X, Trümper L, Wulf GG, StröbelP. CD31 Expression Determines Redox Status and Chemoresistance in Human  Angiosarcomas. Clin Cancer Res. 2018 Jan 15;24(2):460-473. doi:10.1158/1078-0432.CCR-17-1778. Epub 2017 Oct 30. PMID: 29084920; PMCID:PMC7464789.  72: Javidi-Sharifi N, Traer E, Martinez J, Gupta A, Taguchi T, Dunlap J,Heinrich MC, Corless CL, Rubin BP, Druker BJ, Tyner JW. Crosstalk between KITand FGFR3Promotes Gastrointestinal Stromal Tumor Cell Growth and DrugResistance. Cancer Res. 2015 Mar 1;75(5):880-91. doi:10.1158/0008-5472.CAN-14-0573. Epub 2014 Nov 28. PMID: 25432174; PMCID:PMC4348216.  73: Wang Z, Dai J, Yan J, Zhang Y, Yin Z. Targeting EZH2 as a novel therapeuticstrategy for sorafenib-resistant thyroid carcinoma. J Cell Mol Med. 2019Jul;23(7):4770-4778. doi: 10.1111/jcmm.14365. Epub 2019 May 13. PMID: 31087496;PMCID: PMC6584518.  74: Andersen GB, Knudsen A, Hager H, Hansen LL, Tost J. miRNA profilingidentifies deregulated miRNAs associated with osteosarcoma development and timeto metastasis in two large cohorts. Mol Oncol. 2018 Jan;12(1):114-131. doi:10.1002/1878-0261.12154. Epub 2017 Dec 1. PMID: 29120535; PMCID: PMC5748490.  75: Zhu KP, Ma XL, Zhang CL. LncRNA ODRUL Contributes to OsteosarcomaProgression through the miR-3182/MMP2 Axis. Mol Ther. 2017 Oct4;25(10):2383-2393. doi: 10.1016/j.ymthe.2017.06.027. Epub 2017 Jul 5. PMID:28750740; PMCID: PMC5628795.  76: Lei X, Zhu Y, Jones T, Bai Z, Huang Y, Gao SJ. A Kaposi's sarcoma-associatedherpesvirus microRNA and its variants target the transforming growth factor βpathway to promote cell survival. J Virol. 2012 Nov;86(21):11698-711. doi:10.1128/JVI.06855-11. Epub 2012 Aug 22. PMID: 22915806; PMCID: PMC3486299.  77: Pan W, Wang H, Jianwei R, Ye Z. MicroRNA-27a promotes proliferation,migration and invasion by targeting MAP2K4 in human osteosarcoma cells. CellPhysiol Biochem. 2014;33(2):402-12. doi: 10.1159/000356679. Epub 2014 Feb 11.PMID: 24556602.  78: Qi XT, Li YL, Zhang YQ, Xu T, Lu B, Fang L, Gao JQ, Yu LS, Zhu DF, Yang B,He QJ, Ying MD. KLF4 functions as an oncogene in promoting cancer stem cell-likecharacteristics in osteosarcoma cells. Acta Pharmacol Sin. 2019Apr;40(4):546-555. doi: 10.1038/s41401-018-0050-6. Epub 2018 Jun 21. PMID:29930276; PMCID: PMC6461873.  79: Mihály D, Nagy N, Papp G, Pápai Z, Sápi Z. Release of circulating tumorcells and cell-free nucleic acids is an infrequent event in synovial sarcoma:liquid biopsy analysis of 15 patients diagnosed with synovial sarcoma. DiagnPathol. 2018 Oct 17;13(1):81. doi: 10.1186/s13000-018-0756-2. PMID: 30326929;PMCID: PMC6191904.  80: Ware KE, Gilja S, Xu S, Shetler S, Jolly MK, Wang X, Bartholf Dewitt S, HishAJ, Jordan S, Eward W, Levine H, Armstrong AJ, Somarelli JA. Induction ofMesenchymal-Epithelial Transitions in Sarcoma Cells. J Vis Exp. 2017 Apr7;(122):55520. doi: 10.3791/55520. PMID: 28448023; PMCID: PMC5564486.  81: Gits CM, van Kuijk PF, Jonkers MB, Boersma AW, van Ijcken WF, Wozniak A,Sciot R, Rutkowski P, Schöffski P, Taguchi T, Mathijssen RH, Verweij J, SleijferS, Debiec-Rychter M, Wiemer EA. MiR-17-92 and miR-221/222 cluster members targetKIT and ETV1 in human gastrointestinal stromal tumours. Br J Cancer. 2013 Sep17;109(6):1625-35. doi: 10.1038/bjc.2013.483. Epub 2013 Aug 22. PMID: 23969726;PMCID: PMC3776993.  82: Marampon F, Codenotti S, Megiorni F, Del Fattore A, Camero S, Gravina GL,Festuccia C, Musio D, De Felice F, Nardone V, Santoro AN, Dominici C, Fanzani A,  Pirtoli L, Fioravanti A, Tombolini V, Cheleschi S, Tini P. NRF2 orchestrates theredox regulation induced by radiation therapy, sustaining embryonal and alveolar  rhabdomyosarcoma cells radioresistance. J Cancer Res Clin Oncol. 2019Apr;145(4):881-893. doi: 10.1007/s00432-019-02851-0. Epub 2019 Jan 30. PMID:  30701326.  83: Guo S, Bai R, Liu W, Zhao A, Zhao Z, Wang Y, Wang Y, Zhao W, Wang W. miR-22inhibits osteosarcoma cell proliferation and migration by targeting HMGB1 andinhibiting HMGB1-mediated autophagy. Tumour Biol. 2014 Jul;35(7):7025-34. doi:10.1007/s13277-014-1965-2. Epub 2014 Apr 22. PMID: 24752578.  84: Chu F, Chou PM, Zheng X, Mirkin BL, Rebbaa A. Control of multidrugresistance gene mdr1 and cancer resistance to chemotherapy by the longevity genesirt1. Cancer Res. 2005 Nov 15;65(22):10183-7. doi:10.1158/0008-5472.CAN-05-2002. PMID: 16288004.  85: Sun K, Zhao J. A risk assessment model for the prognosis of osteosarcomautilizing differentially expressed lncRNAs. Mol Med Rep. 2019Feb;19(2):1128-1138. doi: 10.3892/mmr.2018.9768. Epub 2018 Dec 14. PMID:30569146; PMCID: PMC6323200.  86: Cai CK, Zhao GY, Tian LY, Liu L, Yan K, Ma YL, Ji ZW, Li XX, Han K, Gao J,Qiu XC, Fan QY, Yang TT, Ma BA. miR-15a and miR-16-1 downregulate CCND1 andinduce apoptosis and cell cycle arrest in osteosarcoma. Oncol Rep. 2012Nov;28(5):1764-70. doi: 10.3892/or.2012.1995. Epub 2012 Aug 24. PMID: 22922827.  87: Gu R, Sun YF, Wu MF, Liu JB, Jiang JL, Wang SH, Wang XL, Guo Q. Biologicalroles of microRNA-140 in tumor growth, migration, and metastasis of osteosarcoma  in vivo and in vitro. Tumour Biol. 2016 Jan;37(1):353-60. doi:10.1007/s13277-015-3801-8. Epub 2015 Jul 29. PMID: 26219893.  88: Vallabhaneni KC, Hassler MY, Abraham A, Whitt J, Mo YY, Atfi A, PochampallyR. Mesenchymal Stem/Stromal Cells under Stress Increase Osteosarcoma Migrationand Apoptosis Resistance via Extracellular Vesicle Mediated Communication. PLoSOne. 2016 Nov 3;11(11):e0166027. doi: 10.1371/journal.pone.0166027. PMID:27812189; PMCID: PMC5094708.  89: Gougelet A, Pissaloux D, Besse A, Perez J, Duc A, Dutour A, Blay JY, AlbertiL. Micro-RNA profiles in osteosarcoma as a predictive tool for ifosfamideresponse. Int J Cancer. 2011 Aug 1;129(3):680-90. doi: 10.1002/ijc.25715. Epub2010 Nov 23. PMID: 20949564.  90: Hill KE, Kelly AD, Kuijjer ML, Barry W, Rattani A, Garbutt CC, Kissick H,Janeway K, Perez-Atayde A, Goldsmith J, Gebhardt MC, Arredouani MS, Cote G,Hornicek F, Choy E, Duan Z, Quackenbush J, Haibe-Kains B, Spentzos D. Animprinted non-coding genomic cluster at 14q32 defines clinically relevantmolecular subtypes in osteosarcoma across multiple independent datasets. JHematol Oncol. 2017 May 15;10(1):107. doi: 10.1186/s13045-017-0465-4. PMID:28506242; PMCID: PMC5433149.  91: Ouyang L, Liu P, Yang S, Ye S, Xu W, Liu X. A three-plasma miRNA signatureserves as novel biomarkers for osteosarcoma. Med Oncol. 2013 Mar;30(1):340. doi:  10.1007/s12032-012-0340-7. Epub 2012 Dec 27. PMID: 23269581.  92: Molina-Ortiz D, Torres-Zárate C, Cárdenas-Cardós R, Palacios-Acosta JM,Hernández-Arrazola D, Shalkow-Klincovstein J, Díaz-Díaz E, Vences-Mejía A. MDR1not CYP3A4 gene expression is the predominant mechanism of innate drugresistance in pediatric soft tissue sarcoma patients. Cancer Biomark.2018;22(2):317-324. doi: 10.3233/CBM-171027. PMID: 29689707.  93: Zhao D, Yuan H, Yi F, Meng C, Zhu Q. Autophagy prevents doxorubicin‑inducedapoptosis in osteosarcoma. Mol Med Rep. 2014 May;9(5):1975-81. doi:10.3892/mmr.2014.2055. Epub 2014 Mar 14. PMID: 24639013.  94: Seo SW, Lee H, Lee HI, Kim HS. The role of TLE1 in synovial sarcoma. JOrthop Res. 2011 Jul;29(7):1131-6. doi: 10.1002/jor.21318. Epub 2011 Feb 11.PMID: 21319215.  95: Nie CL, Ren WH, Ma Y, Xi JS, Han B. Circulating miR-125b as a biomarker ofEwing's sarcoma in Chinese children. Genet Mol Res. 2015 Dec 29;14(4):19049-56.doi: 10.4238/2015.December.29.12. PMID: 26782555.  96: Kong L, Li X, Wang H, He G, Tang A. Calycosin inhibits nasopharyngealcarcinoma cells by influencing EWSAT1 expression to regulate the TRAF6-relatedpathways. Biomed Pharmacother. 2018 Oct;106:342-348. doi:10.1016/j.biopha.2018.06.143. Epub 2018 Jul 11. PMID: 29966979.  97: Huang C, Wang Q, Ma S, Sun Y, Vadamootoo AS, Jin C. A four serum-miRNApanel serves as a potential diagnostic biomarker of osteosarcoma. Int J ClinOncol. 2019 Aug;24(8):976-982. doi: 10.1007/s10147-019-01433-x. Epub 2019 May20. PMID: 31111286.  98: Lin ST, May EW, Chang JF, Hu RY, Wang LH, Chan HL. PGRMC1 contributes todoxorubicin-induced chemoresistance in MES-SA uterine sarcoma. Cell Mol LifeSci. 2015 Jun;72(12):2395-409. doi: 10.1007/s00018-014-1831-9. Epub 2015 Jan 18.Erratum in: Cell Mol Life Sci. 2015 Jun;72(12):2411-3. PMID: 25596698.  99: Pehserl AM, Ress AL, Stanzer S, Resel M, Karbiener M, Stadelmeyer E,Stiegelbauer V, Gerger A, Mayr C, Scheideler M, Hutterer GC, Bauernhofer T,Kiesslich T, Pichler M. Comprehensive Analysis of miRNome Alterations inResponse to Sorafenib Treatment in Colorectal Cancer Cells. Int J Mol Sci. 2016Dec 1;17(12):2011. doi: 10.3390/ijms17122011. PMID: 27916938; PMCID: PMC5187811.  100: Huo Y, Li Q, Wang X, Jiao X, Zheng J, Li Z, Pan X. MALAT1 predicts poorsurvival in osteosarcoma patients and promotes cell metastasis throughassociating with EZH2. Oncotarget. 2017 Jul 18;8(29):46993-47006. doi:10.18632/oncotarget.16551. PMID: 28388584; PMCID: PMC5564539.  101: Fattore L, Mancini R, Acunzo M, Romano G, Laganà A, Pisanu ME, Malpicci D,Madonna G, Mallardo D, Capone M, Fulciniti F, Mazzucchelli L, Botti G, Croce CM,Ascierto PA, Ciliberto G. miR-579-3p controls melanoma progression andresistance to target therapy. Proc Natl Acad Sci U S A. 2016 Aug23;113(34):E5005-13. doi: 10.1073/pnas.1607753113. Epub 2016 Aug 8. PMID:27503895; PMCID: PMC5003278.  102: Li Q, Song S, Ni G, Li Y, Wang X. Serum miR-542-3p as a prognosticbiomarker in osteosarcoma. Cancer Biomark. 2018 Feb 14;21(3):521-526. doi:10.3233/CBM-170255. PMID: 29103020.  103: Xu H, Liu X, Zhao J. Down-regulation of miR-3928 promoted osteosarcomagrowth. Cell Physiol Biochem. 2014;33(5):1547-56. doi: 10.1159/000358718. Epub2014 May 14. PMID: 24854843.  104: Dietrich P, Koch A, Fritz V, Hartmann A, Bosserhoff AK, Hellerbrand C. Wildtype Kirsten rat sarcoma is a novel microRNA-622-regulated therapeutic targetfor hepatocellular carcinoma and contributes to sorafenib resistance. Gut. 2018Jul;67(7):1328-1341. doi: 10.1136/gutjnl-2017-315402. Epub 2017 Dec 23. PMID:29275358.  105: Rink L, Ochs MF, Zhou Y, von Mehren M, Godwin AK. ZNF-mediated resistanceto imatinib mesylate in gastrointestinal stromal tumor. PLoS One.2013;8(1):e54477. doi: 10.1371/journal.pone.0054477. Epub 2013 Jan 25. PMID:23372733; PMCID: PMC3556080.  106: Xuan P, Jia L, Zhang T, Sheng N, Li X, Li J. LDAPred: A Method Based onInformation Flow Propagation and a Convolutional Neural Network for thePrediction of Disease-Associated lncRNAs. Int J Mol Sci. 2019 Sep10;20(18):4458. doi: 10.3390/ijms20184458. PMID: 31510011; PMCID: PMC6771133.  107: Wang Y, Huang JW, Li M, Cavenee WK, Mitchell PS, Zhou X, Tewari M, FurnariFB, Taniguchi T. MicroRNA-138 modulates DNA damage response by repressinghistone H2AX expression. Mol Cancer Res. 2011 Aug;9(8):1100-11. doi:10.1158/1541-7786.MCR-11-0007. Epub 2011 Jun 21. PMID: 21693595; PMCID:PMC3157593.  108: Vergani E, Di Guardo L, Dugo M, Rigoletto S, Tragni G, Ruggeri R, PerroneF, Tamborini E, Gloghini A, Arienti F, Vergani B, Deho P, De Cecco L, VallacchiV, Frati P, Shahaj E, Villa A, Santinami M, De Braud F, Rivoltini L, Rodolfo M.Overcoming melanoma resistance to vemurafenib by targeting CCL2-induced miR-34a,miR-100 and miR-125b. Oncotarget. 2016 Jan 26;7(4):4428-41. doi:10.18632/oncotarget.6599. PMID: 26684239; PMCID: PMC4826216.  109: Lechler P, Renkawitz T, Campean V, Balakrishnan S, Tingart M, Grifka J,Schaumburger J. The antiapoptotic gene survivin is highly expressed in humanchondrosarcoma and promotes drug resistance in chondrosarcoma cells in vitro.BMC Cancer. 2011 Apr 2;11:120. doi: 10.1186/1471-2407-11-120. PMID: 21457573;PMCID: PMC3076263.  110: Johnson KM, Taslim C, Saund RS, Lessnick SL. Identification of two types ofGGAA-microsatellites and their roles in EWS/FLI binding and gene regulation inEwing sarcoma. PLoS One. 2017 Nov 1;12(11):e0186275. doi:10.1371/journal.pone.0186275. PMID: 29091716; PMCID: PMC5665490.  111: Xia YZ, Yang L, Xue GM, Zhang C, Guo C, Yang YW, Li SS, Zhang LY, Guo QL,Kong LY. Combining GRP78 suppression and MK2206-induced Akt inhibition decreasesdoxorubicin-induced P-glycoprotein expression and mitigates chemoresistance inhuman osteosarcoma. Oncotarget. 2016 Aug 30;7(35):56371-56382. doi:10.18632/oncotarget.10890. PMID: 27486760; PMCID: PMC5302920.  112: Torreggiani E, Roncuzzi L, Perut F, Zini N, Baldini N. Multimodal transferof MDR by exosomes in human osteosarcoma. Int J Oncol. 2016 Jul;49(1):189-96.doi: 10.3892/ijo.2016.3509. Epub 2016 May 9. PMID: 27176642.  113: Hong Q, Fang J, Pang Y, Zheng J. Prognostic value of the microRNA-29 familyin patients with primary osteosarcomas. Med Oncol. 2014 Aug;31(8):37. doi:10.1007/s12032-014-0037-1. Epub 2014 Jul 12. PMID: 25015394.  114: Liu F, Xing L, Zhang X, Zhang X. A Four-Pseudogene Classifier Identified byMachine Learning Serves as a Novel Prognostic Marker for Survival ofOsteosarcoma.Genes (Basel). 2019 May 29;10(6):414. doi: 10.3390/genes10060414.PMID: 31146489; PMCID: PMC6628621.  115: Li D, Wang X, Yang M, Kan Q, Duan Z. miR3609 sensitizes breast cancer cellsto adriamycin by blocking the programmed death-ligand 1 immune checkpoint. ExpCell Res. 2019 Jul 1;380(1):20-28. doi: 10.1016/j.yexcr.2019.03.025. Epub 2019Mar 20. PMID: 30904483.  116: Liu W, Zhao ZY, Shi L, Yuan WD. Tissue microRNA-126 expression levelpredicts outcome in human osteosarcoma. Diagn Pathol. 2015 Jul 21;10:116. doi:10.1186/s13000-015-0329-6. PMID: 26194657; PMCID: PMC4509614.  117: Ma J, Huang H, Han Z, Zhu C, Yue B. RLN2 Is a Positive Regulator ofAKT-2-Induced Gene Expression Required for Osteosarcoma Cells Invasion andChemoresistance. Biomed Res Int. 2015;2015:147468. doi: 10.1155/2015/147468.Epub 2015 Jul 1. PMID: 26229955; PMCID: PMC4503584.  118: Gasparini P, Cascione L, Landi L, Carasi S, Lovat F, Tibaldi C, Alì G,D'Incecco A, Minuti G, Chella A, Fontanini G, Fassan M, Cappuzzo F, Croce CM.microRNA classifiers are powerful diagnostic/prognostic tools in ALK-, EGFR-,and KRAS-driven lung cancers. Proc Natl Acad Sci U S A. 2015 Dec1;112(48):14924-9. doi: 10.1073/pnas.1520329112. Epub 2015 Nov 16. PMID:26627242; PMCID: PMC4672770.  119: Liu Y, Sun R, Lin X, Liang D, Deng Q, Lan K. Kaposi's sarcoma-associatedherpesvirus-encoded microRNA miR-K12-11 attenuates transforming growth factorbeta signaling through suppression of SMAD5. J Virol. 2012 Feb;86(3):1372-81.doi: 10.1128/JVI.06245-11. Epub 2011 Oct 19. Erratum in: J Virol. 2021 Jan28;95(4): PMID: 22013049; PMCID: PMC3264391.  120: Zhang CL, Li LB, She C, Xie Y, Ge DW, Dong QR. MiR-299-5p targets cellcycle to promote cell proliferation and progression of osteosarcoma. Eur Rev MedPharmacol Sci. 2018 May;22(9):2606-2613. doi: 10.26355/eurrev_201805_14954.PMID: 29771409.  121: Huang C, Sun Y, Ma S, Vadamootoo AS, Wang L, Jin C. Identification ofcirculating miR-663a as a potential biomarker for diagnosing osteosarcoma.Pathol Res Pract. 2019 Jun;215(6):152411. doi: 10.1016/j.prp.2019.04.003. Epub2019 Apr 6. PMID: 30987831.  122: Fujiwara T, Uotani K, Yoshida A, Morita T, Nezu Y, Kobayashi E, Yoshida A,Uehara T, Omori T, Sugiu K, Komatsubara T, Takeda K, Kunisada T, Kawamura M,  Kawai A, Ochiya T, Ozaki T. Clinical significance of circulating miR-25-3p as anovel diagnostic and prognostic biomarker in osteosarcoma. Oncotarget. 2017 May16;8(20):33375-33392. doi: 10.18632/oncotarget.16498. PMID: 28380419; PMCID:PMC5464875.  123: Zhang H, Su X, Guo L, Zhong L, Li W, Yue Z, Wang X, Mu Y, Li X, Li R, WangZ. Silencing SATB1 inhibits the malignant phenotype and increases sensitivity ofhuman osteosarcoma U2OS cells to arsenic trioxide. Int J Med Sci. 2014 Oct2;11(12):1262-9. doi: 10.7150/ijms.10038. PMID: 25317073; PMCID: PMC4196128.  124: Brill E, Gobble R, Angeles C, Lagos-Quintana M, Crago A, Laxa B, DecarolisP, Zhang L, Antonescu C, Socci ND, Taylor BS, Sander C, Koff A, Singer S. ZIC1  overexpression is oncogenic in liposarcoma. Cancer Res. 2010 Sep1;70(17):6891-901. doi: 10.1158/0008-5472.CAN-10-0745. Epub 2010 Aug 16. PMID:20713527; PMCID: PMC3266950.  125: Cong C, Wang W, Tian J, Gao T, Zheng W, Zhou C. Identification of serummiR-124 as a biomarker for diagnosis and prognosis in osteosarcoma. CancerBiomark. 2018 Feb 6;21(2):449-454. doi: 10.3233/CBM-170672. PMID: 29125481.  126: Li C, Guo D, Tang B, Zhang Y, Zhang K, Nie L. Notch1 is associated with themultidrug resistance of hypoxic osteosarcoma by regulating MRP1 gene expression.Neoplasma. 2016;63(5):734-42. doi: 10.4149/neo_2016_510. PMID: 27468877.  127: Yamamoto T, Ohno T, Wakahara K, Nagano A, Kawai G, Saitou M, Takigami I,Matsuhashi A, Yamada K, Shimizu K. Simultaneous inhibition of mitogen-activatedprotein kinase and phosphatidylinositol 3-kinase pathways augment thesensitivity to actinomycin D in Ewing sarcoma. J Cancer Res Clin Oncol. 2009Aug;135(8):112536. doi: 10.1007/s00432-009-0554-z. Epub 2009 Feb 10. PMID:19205734.  128: Zou J, Gan M, Mao N, Zhu X, Shi Q, Yang H. Sensitization of osteosarcomacell line SaOS-2 to chemotherapy by downregulating survivin. Arch Med Res. 2010Apr;41(3):162-9. doi: 10.1016/j.arcmed.2010.04.006. PMID: 20682173.  129: Jiang L, Tao C, He A, He X. Overexpression of miR-126 sensitizesosteosarcoma cells to apoptosis induced by epigallocatechin-3-gallate. World JSurg Oncol. 2014 Dec 16;12:383. doi: 10.1186/1477-7819-12-383. PMID: 25510179;PMCID: PMC4300611.  130: Ou WB, Zhu MJ, Demetri GD, Fletcher CD, Fletcher JA. Protein kinase C-thetaregulates KIT expression and proliferation in gastrointestinal stromal tumors.Oncogene. 2008 Sep 18;27(42):5624-34. doi: 10.1038/onc.2008.177. Epub 2008 Jun2. PMID: 18521081; PMCID: PMC2811224.  131: Wang W, Chen D, Zhu K. SOX2OT variant 7 contributes to the synergisticinteraction between EGCG and Doxorubicin to kill osteosarcoma via autophagy and  stemness inhibition. J Exp Clin Cancer Res. 2018 Feb 23;37(1):37. doi:10.1186/s13046-018-0689-3. PMID: 29475441; PMCID: PMC6389193.  132: Wu X, Ruan Y, Jiang H, Xu C. MicroRNA-424 inhibits cell migration,invasion, and epithelial mesenchymal transition by downregulating doublecortin-like kinase 1 in ovarian clear cell carcinoma. Int J Biochem Cell Biol. 2017Apr;85:66-74. doi: 10.1016/j.biocel.2017.01.020. Epub 2017 Feb 2. PMID:28161486.  133: Koelz M, Lense J, Wrba F, Scheffler M, Dienes HP, Odenthal M. Down-regulation of miR-221 and miR-222 correlates with pronounced Kit expression ingastrointestinal stromal tumors. Int J Oncol. 2011 Feb;38(2):503-11. doi:10.3892/ijo.2010.857. Epub 2010 Dec 3. PMID: 21132270.  134: Huang HJ, Angelo LS, Rodon J, Sun M, Kuenkele KP, Parsons HA, Trent JC,Kurzrock R. R1507, an anti-insulin-like growth factor-1 receptor (IGF-1R)antibody, and EWS/FLI-1 siRNA in Ewing's sarcoma: convergence at theIGF/IGFR/Akt axis. PLoS One. 2011;6(10):e26060. doi:10.1371/journal.pone.0026060. Epub 2011 Oct 11. PMID: 22022506; PMCID:PMC3191161.  135: Tsukamoto S, Shimada K, Honoki K, Kido A, Akahane M, Tanaka Y, Konishi N.Ubiquilin 2 enhances osteosarcoma progression through resistance to hypoxicstress. Oncol Rep. 2015 Apr;33(4):1799-806. doi: 10.3892/or.2015.3788. Epub 2015Feb 9. PMID: 25672654.  136: Xie X, Ye Z, Yang D, Tao H. Effects of combined c-myc and Bmi-1 siRNAs onthe growth and chemosensitivity of MG-63 osteosarcoma cells. Mol Med Rep. 2013Jul;8(1):168-72. doi: 10.3892/mmr.2013.1484. Epub 2013 May 20. PMID: 23685757.  137: Grohar PJ, Segars LE, Yeung C, Pommier Y, D'Incalci M, Mendoza A, HelmanLJ. Dual targeting of EWS-FLI1 activity and the associated DNA damage responsewith trabectedin and SN38 synergistically inhibits Ewing sarcoma cell growth.Clin Cancer Res. 2014 Mar 1;20(5):1190-203. doi: 10.1158/1078-0432.CCR-13-0901.Epub 2013 Nov 25. PMID: 24277455; PMCID: PMC5510643.  138: Min XS, Huang P, Liu X, Dong C, Jiang XL, Yuan ZT, Mao LF, Chang S.Bioinformatics analyses of significant prognostic risk markers for thyroidpapillary carcinoma. Tumour Biol. 2015 Sep;36(10):7457-63. doi:10.1007/s13277-015-3410-6. Epub 2015 Apr 24. PMID: 25908172.  139: Tsai CH, Yang DY, Lin CY, Chen TM, Tang CH, Huang YL.Sphingosine-1-phosphate suppresses chondrosarcoma metastasis by upregulation oftissue inhibitor of metalloproteinase 3 through suppressing miR-101 expression.Mol Oncol. 2017 Oct;11(10):1380-1398. doi: 10.1002/1878-0261.12106. Epub 2017Aug 8. PMID: 28672103; PMCID: PMC5623823.  140: Song L, Duan P, Gan Y, Li P, Zhao C, Xu J, Zhang Z, Zhou Q. SilencingLPAATβ inhibits tumor growth of cisplatin-resistant human osteosarcoma in vivoand in vitro. Int J Oncol. 2017 Feb;50(2):535-544. doi: 10.3892/ijo.2016.3820.Epub 2016 Dec 28. PMID: 28035350.  141: Wang NG, Wang DC, Tan BY, Wang F, Yuan ZN. Down-regulation of microRNA152is associated with the diagnosis and prognosis of patients with osteosarcoma.Int J Clin Exp Pathol. 2015 Aug 1;8(8):9314-9. PMID: 26464682; PMCID:PMC4583914.  142: Marino MT, Grilli A, Baricordi C, Manara MC, Ventura S, Pinca RS, BellenghiM, Calvaruso M, Mattia G, Donati D, Tripodo C, Picci P, Ferrari S, Scotlandi K.Prognostic significance of miR-34a in Ewing sarcoma is associated with cyclin D1and ki-67 expression. Ann Oncol. 2014 Oct;25(10):2080-2086. doi:10.1093/annonc/mdu249. Epub 2014 Jul 10. PMID: 25015333.  143: Garofalo M, Romano G, Di Leva G, Nuovo G, Jeon YJ, Ngankeu A, Sun J, LovatF, Alder H, Condorelli G, Engelman JA, Ono M, Rho JK, Cascione L, Volinia S,Nephew KP, Croce CM. EGFR and MET receptor tyrosine kinase-altered microRNAexpression induces tumorigenesis and gefitinib resistance in lung cancers. NatMed. 2011 Dec 11;18(1):74-82. doi: 10.1038/nm.2577. Erratum in: Nat Med. 2014Jan;20(1):103. PMID: 22157681; PMCID: PMC3467100.  144: Zhu X, Du X, Deng X, Yi H, Cui S, Liu W, Shen A, Cui Z. C6 ceramidesensitizes pemetrexed-induced apoptosis and cytotoxicity in osteosarcoma cells.Biochem Biophys Res Commun. 2014 Sep 12;452(1):72-8. doi:10.1016/j.bbrc.2014.08.065. Epub 2014 Aug 22. PMID: 25152399.  145: Thayanithy V, Dickson EL, Steer C, Subramanian S, Lou E. Tumor-stromalcross talk: direct cell-to-cell transfer of oncogenic microRNAs via tunnelingnanotubes. Transl Res. 2014 Nov;164(5):359-65. doi: 10.1016/j.trsl.2014.05.011.Epub 2014 May 24. PMID: 24929208; PMCID: PMC4242806.  146: Tu L, Wang M, Zhao WY, Zhang ZZ, Tang DF, Zhang YQ, Cao H, Zhang ZG.miRNA-218-loaded carboxymethyl chitosan - Tocopherol nanoparticle to suppressthe proliferation of gastrointestinal stromal tumor growth. Mater Sci Eng CMater Biol Appl. 2017 Mar 1;72:177-184. doi: 10.1016/j.msec.2016.10.052. Epub2016 Oct 26. PMID: 28024574.  147: Mensah-Osman EJ, Thomas DG, Tabb MM, Larios JM, Hughes DP, Giordano TJ,Lizyness ML, Rae JM, Blumberg B, Hollenberg PF, Baker LH. Expression levels andactivation of a PXR variant are directly related to drug resistance inosteosarcoma cell lines. Cancer. 2007 Mar 1;109(5):957-65. doi:10.1002/cncr.22479. Erratum in: Cancer. 2013 Mar 1;119(5):1115. PMID: 17279585;PMCID: PMC3125968.  148: Lian F, Cui Y, Zhou C, Gao K, Wu L. Identification of a plasma four-microRNA panel as potential noninvasive biomarker for osteosarcoma. PLoS One.2015 Mar 16;10(3):e0121499. doi: 10.1371/journal.pone.0121499. PMID: 25775010;PMCID: PMC4361617.  149: Wiemer EAC, Wozniak A, Burger H, Smid M, Floris G, Nzokirantevye A, SciotR, Sleijfer S, Schöffski P. Identification of microRNA biomarkers for responseof advanced soft tissue sarcomas to eribulin: Translational results of the EORTC62052 trial. Eur J Cancer. 2017 Apr;75:33-40. doi: 10.1016/j.ejca.2016.12.018.Epub 2017 Feb 16. PMID: 28214655.  150: Bao Y, Chen B, Wu Q, Hu K, Xi X, Zhu W, Zhong X, Chen J. Overexpression ofmiR-664 is associated with enhanced osteosarcoma cell migration and invasionability via targeting SOX7. Clin Exp Med. 2017 Feb;17(1):51-58. doi:10.1007/s10238-015-0398-6. Epub 2015 Oct 29. PMID: 26515813.  151: Klug LR, Bannon AE, Javidi-Sharifi N, Town A, Fleming WH, VanSlyke JK,Musil LS, Fletcher JA, Tyner JW, Heinrich MC. LMTK3 is essential for oncogenicKIT expression in KIT-mutant GIST and melanoma. Oncogene. 2019Feb;38(8):1200-1210. doi: 10.1038/s41388-018-0508-5. Epub 2018 Sep 21. PMID:30242244; PMCID: PMC6365197.  152: Rahman MT, Nakayama K, Ishikawa M, Rahman M, Katagiri H, Katagiri A,Ishibashi T, Iida K, Yamada T, Miyazaki K. NAC1, a BTB/POZ protein overexpressedin uterine sarcomas. Anticancer Res. 2012 Sep;32(9):3841-5. PMID: 22993327.  153: Kun-Peng Z, Chun-Lin Z, Jian-Ping H, Lei Z. A novel circulatinghsa_circ_0081001 act as a potential biomarker for diagnosis and prognosis ofosteosarcoma. Int J Biol Sci. 2018 Sep 1;14(11):1513-1520. doi:10.7150/ijbs.27523. PMID: 30263004; PMCID: PMC6158732.  154: Susa M, Iyer AK, Ryu K, Choy E, Hornicek FJ, Mankin H, Milane L, Amiji MM,Duan Z. Inhibition of ABCB1 (MDR1) expression by an siRNA nanoparticulatedelivery system to overcome drug resistance in osteosarcoma. PLoS One. 2010 May24;5(5):e10764. doi: 10.1371/journal.pone.0010764. PMID: 20520719; PMCID:PMC2875382.  155: Wang B, Howel P, Bruheim S, Ju J, Owen LB, Fodstad O, Xi Y. Systematicevaluation of three microRNA profiling platforms: microarray, beads array, andquantitative real-time PCR array. PLoS One. 2011 Feb 11;6(2):e17167. doi:10.1371/journal.pone.0017167. PMID: 21347261; PMCID: PMC3037970.  156: Fujiwara T, Katsuda T, Hagiwara K, Kosaka N, Yoshioka Y, Takahashi RU,Takeshita F, Kubota D, Kondo T, Ichikawa H, Yoshida A, Kobayashi E, Kawai A,Ozaki T, Ochiya T. Clinical relevance and therapeutic significance ofmicroRNA-133a expression profiles and functions in malignant osteosarcoma-initiating cells. Stem Cells. 2014 Apr;32(4):959-73. doi: 10.1002/stem.1618.PMID: 24715690.  157: Jullien N, Dieudonné FX, Habel N, Marty C, Modrowski D, Patino A, LecandaF, Sévère N, Marie PJ. ErbB3 silencing reduces osteosarcoma cell proliferationand tumor growth in vivo. Gene. 2013 May 25;521(1):55-61. doi:10.1016/j.gene.2013.03.031. Epub 2013 Mar 27. PMID: 23541807.  158: Li S, Cui Z, Meng X. Knockdown of PARP-1 Inhibits Proliferation and ERKSignals, Increasing Drug Sensitivity in Osteosarcoma U2OS Cells. Oncol Res.2016;24(4):279-86. doi: 10.3727/096504016X14666990347554. PMID: 27656839; PMCID:PMC7838669.  159: Huang G, Mills L, Worth LL. Expression of human glutathione S-transferaseP1 mediates the chemosensitivity of osteosarcoma cells. Mol Cancer Ther. 2007May;6(5):1610-9. doi: 10.1158/1535-7163.MCT-06-0580. PMID: 17513610.  160: Zhou L, Ma X, Yue J, Chen T, Wang XY, Wang ZW, Pan J, Lin Y. The diagnosticeffect of serum miR-139-5p as an indicator in osteosarcoma. Cancer Biomark.2018;23(4):561-567. doi: 10.3233/CBM-181744. PMID: 30347602.  161: Sero V, Tavanti E, Vella S, Hattinger CM, Fanelli M, Michelacci F, VersteegR, Valsasina B, Gudeman B, Picci P, Serra M. Targeting polo-like kinase 1 byNMS-P937 in osteosarcoma cell lines inhibits tumor cell growth and partiallyovercomes drug resistance. Invest New Drugs. 2014 Dec;32(6):1167-80. doi:10.1007/s10637-014-0158-6. Epub 2014 Sep 7. PMID: 25193492.  162: Senturk T, Antal A, Gunel T. Potential function of microRNAs in thoracicaortic aneurysm and thoracic aortic dissection pathogenesis. Mol Med Rep. 2019Dec;20(6):5353-5362. doi: 10.3892/mmr.2019.10761. Epub 2019 Oct 22. PMID:31638233.  163: Myatt SS, Burchill SA. The sensitivity of the Ewing's sarcoma family oftumours to fenretinide-induced cell death is increased by EWS-Fli1-dependentmodulation of p38(MAPK) activity. Oncogene. 2008 Feb 7;27(7):985-96. doi:10.1038/sj.onc.1210705. Epub 2007 Aug 13. PMID: 17700534.  164: Goss KL, Gordon DJ. Gene expression signature based screening identifiesribonucleotide reductase as a candidate therapeutic target in Ewing sarcoma.Oncotarget. 2016 Sep 27;7(39):63003-63019. doi: 10.18632/oncotarget.11416. PMID:27557498; PMCID: PMC5325343.  165: Zheng L, Kang Y, Zhang L, Zou W. MiR-133a-5p inhibits androgen receptor(AR)-induced proliferation in prostate cancer cells via targeting FUsed inSarcoma (FUS) and AR. Cancer Biol Ther. 2020;21(1):34-42. doi:10.1080/15384047.2019.1665393. Epub 2019 Nov 17. PMID: 31736422; PMCID:PMC7012176.  166: Jin Z, Aixi Y, Baiwen Q, Zonghuan L, Xiang H. Inhibition of hypoxia-inducible factor-1 alpha radiosensitized MG-63 human osteosarcoma cells invitro. Tumori. 2015 Sep-Oct;101(5):578-84. doi: 10.5301/tj.5000243. Epub 2015Aug 4. PMID: 26350185.  167: Nieto-Soler M, Morgado-Palacin I, Lafarga V, Lecona E, Murga M, Callen E,Azorin D, Alonso J, Lopez-Contreras AJ, Nussenzweig A, Fernandez-Capetillo O.Efficacy of ATR inhibitors as single agents in Ewing sarcoma. Oncotarget. 2016Sep 13;7(37):58759-58767. doi: 10.18632/oncotarget.11643. PMID: 27577084; PMCID:PMC5312273.  168: Wu Z, Min L, Chen D, Hao D, Duan Y, Qiu G, Wang Y. Overexpression of BMI-1promotes cell growth and resistance to cisplatin treatment in osteosarcoma. PLoSOne. 2011 Feb 2;6(2):e14648. doi: 10.1371/journal.pone.0014648. PMID: 21311599;PMCID: PMC3032734.  169: Wang D, Luo M, Kelley MR. Human apurinic endonuclease 1 (APE1) expressionand prognostic significance in osteosarcoma: enhanced sensitivity ofosteosarcoma to DNA damaging agents using silencing RNA APE1 expressioninhibition. Mol Cancer Ther. 2004 Jun;3(6):679-86. PMID: 15210853.  170: Zhang H, Wang Y, Xing F, Wang J, Wang Y, Wang H, Yang Y, Gao Z.Overexpression of LIMK1 promotes migration ability of multidrug-resistantosteosarcoma cells. Oncol Res. 2011;19(10-11):501-9. doi:10.3727/096504012x13286534482511. PMID: 22715593.  171: Hsueh YS, Chang HH, Chiang NJ, Yen CC, Li CF, Chen LT. MTOR inhibitionenhances NVP-AUY922-induced autophagy-mediated KIT degradation and cytotoxicityin imatinib-resistant gastrointestinal stromal tumors. Oncotarget. 2014 Nov30;5(22):11723-36. doi: 10.18632/oncotarget.2607. PMID: 25375091; PMCID:PMC4294368.  172: Wu MH, Huang PH, Hsieh M, Tsai CH, Chen HT, Tang CH. Endothelin-1 promotesepithelial-mesenchymal transition in human chondrosarcoma cells by repressingmiR-300. Oncotarget. 2016 Oct 25;7(43):70232-70246. doi:10.18632/oncotarget.11835. PMID: 27602960; PMCID: PMC5342549.  173: Zhen YF, Li ST, Zhu YR, Wang XD, Zhou XZ, Zhu LQ. Identification of DNA-PKcs as a primary resistance factor of salinomycin in osteosarcoma cells.Oncotarget. 2016 Nov 29;7(48):79417-79427. doi: 10.18632/oncotarget.12712. PMID:27765904; PMCID: PMC5346724.  174: Potratz J, Tillmanns A, Berning P, Korsching E, Schaefer C, Lechtape B,Schleithoff C, Unland R, Schäfer KL, Müller-Tidow C, Jürgens H, Dirksen U.Receptor tyrosine kinase gene expression profiles of Ewing sarcomas reveal ROR1as a potential therapeutic target in metastatic disease. Mol Oncol. 2016May;10(5):677-92. doi: 10.1016/j.molonc.2015.12.009. Epub 2015 Dec 20. PMID:26739507; PMCID: PMC5423155.  175: Chaudhry MA, Kreger B, Omaruddin RA. Transcriptional modulation of micro-RNA in human cells differing in radiation sensitivity. Int J Radiat Biol. 2010Jul;86(7):569-83. doi: 10.3109/09553001003734568. PMID: 20545570.  176: Moneo V, Serelde BG, Fominaya J, Leal JF, Blanco-Aparicio C, Romero L,Sánchez-Beato M, Cigudosa JC, Tercero JC, Piris MA, Jimeno J, Carnero A. Extremesensitivity to Yondelis (Trabectedin, ET-743) in low passaged sarcoma cell linescorrelates with mutated p53. J Cell Biochem. 2007 Feb 1;100(2):339-48. doi:10.1002/jcb.21073. PMID: 16888811.  177: Grunewald TG, Diebold I, Esposito I, Plehm S, Hauer K, Thiel U, da Silva-Buttkus P, Neff F, Unland R, Müller-Tidow C, Zobywalski C, Lohrig K,Lewandrowski U, Sickmann A, Prazeres da Costa O, Görlach A, Cossarizza A, ButtE, Richter GH, Burdach S. STEAP1 is associated with the invasive and oxidativestress phenotype of Ewing tumors. Mol Cancer Res. 2012 Jan;10(1):52-65. doi:10.1158/1541-7786.MCR-11-0524. Epub 2011 Nov 11. PMID: 22080479.  178: Zheng H, Shen H, Oprea I, Worrall C, Stefanescu R, Girnita A, Girnita L.β-Arrestin-biased agonism as the central mechanism of action for insulin-likegrowth factor 1 receptor-targeting antibodies in Ewing's sarcoma. Proc Natl AcadSci U S A. 2012 Dec 11;109(50):20620-5. doi: 10.1073/pnas.1216348110. Epub 2012Nov 27. PMID: 23188799; PMCID: PMC3528604.  179: Thao le B, Vu HA, Yasuda K, Taniguchi S, Yagasaki F, Taguchi T, Watanabe T,Sato Y. Cas-L was overexpressed in imatinib-resistant gastrointestinal stromaltumor cells. Cancer Biol Ther. 2009 Apr;8(8):683-8. doi: 10.4161/cbt.8.8.7779.PMID: 19417561.  180: Wong RP, Tsang WP, Chau PY, Co NN, Tsang TY, Kwok TT. p53-R273H gains newfunction in induction of drug resistance through down-regulation ofprocaspase-3. Mol Cancer Ther. 2007 Mar;6(3):1054-61. doi:10.1158/1535-7163.MCT-06-0336. PMID: 17363498.  181: Mackintosh C, García-Domínguez DJ, Ordóñez JL, Ginel-Picardo A, Smith PG,Sacristán MP, de Álava E. WEE1 accumulation and deregulation of S-phase proteinsmediate MLN4924 potent inhibitory effect on Ewing sarcoma cells. Oncogene. 2013Mar 14;32(11):1441-51. doi: 10.1038/onc.2012.153. Epub 2012 May 28. PMID:22641220.  182: Posthumadeboer J, van Egmond PW, Helder MN, de Menezes RX, Cleton-JansenAM, Beliën JA, Verheul HM, van Royen BJ, Kaspers GJ, van Beusechem VW. TargetingJNK-interacting-protein-1 (JIP1) sensitises osteosarcoma to doxorubicin.Oncotarget. 2012 Oct;3(10):1169-81. doi: 10.18632/oncotarget.600. PMID:23045411; PMCID: PMC3717953.  183: Sápi Z, Papp G, Szendrői M, Pápai Z, Plótár V, Krausz T, Fletcher CD.Epigenetic regulation of SMARCB1 By miR-206, -381 and -671-5p is evident in avariety of SMARCB1 immunonegative soft tissue sarcomas, while miR-765 appearsspecific for epithelioid sarcoma. A miRNA study of 223 soft tissue sarcomas.Genes Chromosomes Cancer. 2016 Oct;55(10):786-802. doi: 10.1002/gcc.22379. Epub2016 Jun 24. PMID: 27223121.  184: Zhao Z, Tao L, Shen C, Liu B, Yang Z, Tao H. Silencing of Barkor/ATG14sensitizes osteosarcoma cells to cisplatin‑induced apoptosis. Int J Mol Med.2014 Feb;33(2):271-6. doi: 10.3892/ijmm.2013.1578. Epub 2013 Dec 9. PMID:24337183; PMCID: PMC3896476.  185: Hu K, Liao D, Wu W, Han AJ, Shi HJ, Wang F, Wang X, Zhong L, Duan T, Wu Y,Cao J, Tang J, Sang Y, Wang L, Lv X, Xu S, Zhang RH, Deng WG, Li SP, Zeng YX,Kang T. Targeting the anaphase-promoting complex/cyclosome (APC/C)- bromodomaincontaining 7 (BRD7) pathway for human osteosarcoma. Oncotarget. 2014 May30;5(10):3088-100. doi: 10.18632/oncotarget.1816. PMID: 24840027; PMCID:PMC4102794.  186: Gopinath SC, Wadhwa R, Kumar PK. Expression of noncoding vault RNA in humanmalignant cells and its importance in mitoxantrone resistance. Mol Cancer Res.2010 Nov;8(11):1536-46. doi: 10.1158/1541-7786.MCR-10-0242. Epub 2010 Oct 8.PMID: 20881010.  187: Liu Y, Zhao L, Ju Y, Li W, Zhang M, Jiao Y, Zhang J, Wang S, Wang Y, ZhaoM, Zhang B, Zhao Y. A novel androstenedione derivative induces ROS-mediatedautophagy and attenuates drug resistance in osteosarcoma by inhibitingmacrophage migration inhibitory factor (MIF). Cell Death Dis. 2014 Aug7;5(8):e1361. doi: 10.1038/cddis.2014.300. PMID: 25101674; PMCID: PMC4454296.  188: Yang C, Shogren KL, Goyal R, Bravo D, Yaszemski MJ, Maran A. RNA-dependentprotein kinase is essential for 2-methoxyestradiol-induced autophagy inosteosarcoma cells. PLoS One. 2013;8(3):e59406. doi:10.1371/journal.pone.0059406. Epub 2013 Mar 19. PMID: 23527187; PMCID:PMC3602192.  189: Haghiralsadat F, Amoabediny G, Naderinezhad S, Zandieh-Doulabi B,Forouzanfar T, Helder MN. Codelivery of doxorubicin and JIP1 siRNA with novelEphA2-targeted PEGylated cationic nanoliposomes to overcome osteosarcomamultidrug resistance. Int J Nanomedicine. 2018 Jul 3;13:3853-3866. doi:10.2147/IJN.S150017. PMID: 30013340; PMCID: PMC6039070.  190: Mo H, Guan J, Mo L, He J, Wu Z, Lin X, Liu B, Yuan Z. ATF4 regulated by MYChas an important function in anoikis resistance in human osteosarcoma cells. MolMed Rep. 2018 Mar;17(3):3658-3666. doi: 10.3892/mmr.2017.8296. Epub 2017 Dec 18.PMID: 29257326; PMCID: PMC5802171.  191: Parnas O, Corcoran DL, Cullen BR. Analysis of the mRNA targetome ofmicroRNAs expressed by Marek's disease virus. mBio. 2014 Jan 21;5(1):e01060-13.doi: 10.1128/mBio.01060-13. PMID: 24449754; PMCID: PMC3903288.  192: Perez J, Bardin C, Rigal C, Anthony B, Rousseau R, Dutour A. Anti-MDR1siRNA restores chemosensitivity in chemoresistant breast carcinoma andosteosarcoma cell lines. Anticancer Res. 2011 Sep;31(9):2813-20. PMID: 21868524.  193: Vera-Puente O, Rodriguez-Antolin C, Salgado-Figueroa A, Michalska P, PerniaO, Reid BM, Rosas R, Garcia-Guede A, SacristÁn S, Jimenez J, Esteban-RodriguezI, Martin ME, Sellers TA, León R, Gonzalez VM, De Castro J, Ibanez de Caceres I.MAFG is a potential therapeutic target to restore chemosensitivity in cisplatin-resistant cancer cells by increasing reactive oxygen species. Transl Res. 2018Oct;200:1-17. doi: 10.1016/j.trsl.2018.06.005. Epub 2018 Jun 30. PMID: 30053382;PMCID: PMC7787305.  194: Hu K, Lee C, Qiu D, Fotovati A, Davies A, Abu-Ali S, Wai D, Lawlor ER,Triche TJ, Pallen CJ, Dunn SE. Small interfering RNA library screen of humankinases and phosphatases identifies polo-like kinase 1 as a promising new targetfor the treatment of pediatric rhabdomyosarcomas. Mol Cancer Ther. 2009Nov;8(11):3024-35. doi: 10.1158/1535-7163.MCT-09-0365. Epub 2009 Nov 3. PMID:19887553; PMCID: PMC2783569.  195: Huang H, Wang L, Li M, Wang X, Zhang L. Secreted clusterin (sCLU) regulatescell proliferation and chemosensitivity to cisplatin by modulating ERK1/2signals in human osteosarcoma cells. World J Surg Oncol. 2014 Aug 9;12:255. doi:10.1186/1477-7819-12-255. PMID: 25106434; PMCID: PMC4249734.  196: Song J, Li Y, An RF. Identification of Early-Onset Preeclampsia-RelatedGenes and MicroRNAs by Bioinformatics Approaches. Reprod Sci. 2015Aug;22(8):954-63. doi: 10.1177/1933719115570898. Epub 2015 Feb 24. PMID:25717061.  197: Feng T, Qiao G, Feng L, Qi W, Huang Y, Yao Y, Shen Z. Stathmin is key inreversion of doxorubicin resistance by arsenic trioxide in osteosarcoma cells.Mol Med Rep. 2014 Dec;10(6):2985-92. doi: 10.3892/mmr.2014.2618. Epub 2014 Oct9. PMID: 25310700.  198: Huang JW, Wang Y, Dhillon KK, Calses P, Villegas E, Mitchell PS, Tewari M,Kemp CJ, Taniguchi T. Systematic screen identifies miRNAs that target RAD51 andRAD51D to enhance chemosensitivity. Mol Cancer Res. 2013 Dec;11(12):1564-73.doi: 10.1158/1541-7786.MCR-13-0292. Epub 2013 Oct 2. PMID: 24088786; PMCID:PMC3869885.  199: Ory B, Moriceau G, Trichet V, Blanchard F, Berreur M, Rédini F, Rogers M,Heymann D. Farnesyl diphosphate synthase is involved in the resistance tozoledronic acid of osteosarcoma cells. J Cell Mol Med. 2008 Jun;12(3):928-41.doi: 10.1111/j.1582-4934.2008.00141.x. PMID: 18494934; PMCID: PMC4401135.  200: Durso M, Gaglione M, Piras L, Mercurio ME, Terreri S, Olivieri M, MarinelliL, Novellino E, Incoronato M, Grieco P, Orsini G, Tonon G, Messere A, Cimmino A.Chemical modifications in the seed region of miRNAs 221/222 increase thesilencing performances in gastrointestinal stromal tumor cells. Eur J Med Chem.2016 Mar 23;111:15-25. doi: 10.1016/j.ejmech.2016.01.047. Epub 2016 Jan 27.PMID: 26854374.  201: Kim Y, Kang YS, Lee NY, Kim KY, Hwang YJ, Kim HW, Rhyu IJ, Her S, Jung MK,Kim S, Lee CJ, Ko S, Kowall NW, Lee SB, Lee J, Ryu H. Uvrag targeting by Mir125aand Mir351 modulates autophagy associated with Ewsr1 deficiency. Autophagy.2015;11(5):796-811. doi: 10.1080/15548627.2015.1035503. PMID: 25946189; PMCID:PMC4509448.  202: Gupte A, Baker EK, Wan SS, Stewart E, Loh A, Shelat AA, Gould CM, Chalk AM,Taylor S, Lackovic K, Karlström Å, Mutsaers AJ, Desai J, Madhamshettiwar PB,Zannettino AC, Burns C, Huang DC, Dyer MA, Simpson KJ, Walkley CR. SystematicScreening Identifies Dual PI3K and mTOR Inhibition as a Conserved TherapeuticVulnerability in Osteosarcoma. Clin Cancer Res. 2015 Jul 15;21(14):3216-29. doi:10.1158/1078-0432.CCR-14-3026. Epub 2015 Apr 10. PMID: 25862761; PMCID:PMC4506243.  203: Allen-Rhoades W, Kurenbekova L, Satterfield L, Parikh N, Fuja D, Shuck RL,Rainusso N, Trucco M, Barkauskas DA, Jo E, Ahern C, Hilsenbeck S, Donehower LA,Yustein JT. Cross-species identification of a plasma microRNA signature fordetection, therapeutic monitoring, and prognosis in osteosarcoma. Cancer Med.2015 Jul;4(7):977-88. doi: 10.1002/cam4.438. Epub 2015 Mar 17. PMID: 25784290;PMCID: PMC4529336.  204: Ohya S, Kajikuri J, Endo K, Kito H, Elboray EE, Suzuki T. Ca<sup>2+</sup>-activated K<sup>+</sup> channel K<sub>Ca</sub> 1.1 as a therapeutic target toovercome chemoresistance in three-dimensional sarcoma spheroid models. CancerSci. 2021 Sep;112(9):3769-3783. doi: 10.1111/cas.15046. Epub 2021 Jul 16. PMID:34181803; PMCID: PMC8409426.  205: Magwere T, Burchill SA. Heterogeneous role of the glutathione antioxidantsystem in modulating the response of ESFT to fenretinide in normoxia andhypoxia. PLoS One. 2011;6(12):e28558. doi: 10.1371/journal.pone.0028558. Epub2011 Dec 8. PMID: 22174837; PMCID: PMC3234283.  206: Aryee DN, Niedan S, Ban J, Schwentner R, Muehlbacher K, Kauer M, Kofler R,Kovar H. Variability in functional p53 reactivation by PRIMA-1(Met)/APR-246 inEwing sarcoma. Br J Cancer. 2013 Nov 12;109(10):2696-704. doi:10.1038/bjc.2013.635. Epub 2013 Oct 15. PMID: 24129240; PMCID: PMC3833220.  207: Rambow F, Job B, Petit V, Gesbert F, Delmas V, Seberg H, Meurice G, VanOtterloo E, Dessen P, Robert C, Gautheret D, Cornell RA, Sarasin A, Larue L. NewFunctional Signatures for Understanding Melanoma Biology from Tumor CellLineage-Specific Analysis. Cell Rep. 2015 Oct 27;13(4):840-853. doi:10.1016/j.celrep.2015.09.037. Epub 2015 Oct 17. PMID: 26489459; PMCID:PMC5970542.  208: Wang Z, Zhou JY, Kanakapalli D, Buck S, Wu GS, Ravindranath Y. High levelof mitogen-activated protein kinase phosphatase-1 expression is associated withcisplatin resistance in osteosarcoma. Pediatr Blood Cancer. 2008Dec;51(6):754-9. doi: 10.1002/pbc.21727. PMID: 18726921; PMCID: PMC2728603.  209: Pagliuca A, Valvo C, Fabrizi E, di Martino S, Biffoni M, Runci D, Forte S,De Maria R, Ricci-Vitiani L. Analysis of the combined action of miR-143 andmiR-145 on oncogenic pathways in colorectal cancer cells reveals a coordinateprogram of gene repression. Oncogene. 2013 Oct;32(40):4806-13. doi:10.1038/onc.2012.495. Epub 2012 Nov 5. PMID: 23128394.  210: Tong HX, Zhou YH, Hou YY, Zhang Y, Huang Y, Xie B, Wang JY, Jiang Q, He JY,Shao YB, Han WM, Tan RY, Zhu J, Lu WQ. Expression profile of microRNAs ingastrointestinal stromal tumors revealed by high throughput quantitative RT-PCRmicroarray. World J Gastroenterol. 2015 May 21;21(19):5843-55. doi:10.3748/wjg.v21.i19.5843. PMID: 26019448; PMCID: PMC4438018.  211: Luo Z, Li D, Luo X, Li L, Gu S, Yu L, Ma Y. Decreased Expression ofmiR-548c-3p in Osteosarcoma Contributes to Cell Proliferation Via TargetingITGAV. Cancer Biother Radiopharm. 2016 Jun;31(5):153-8. doi:10.1089/cbr.2016.1995. PMID: 27310302.  212: Magalhães M, Almeida M, Tavares-da-Silva E, Roleira FMF, Varela C, Jorge J,Gonçalves AC, Carvalho RA, Veiga F, Santos AC, Figueiras A. miR-145-loadedmicelleplexes as a novel therapeutic strategy to inhibit proliferation andmigration of osteosarcoma cells. Eur J Pharm Sci. 2018 Oct 15;123:28-42. doi:10.1016/j.ejps.2018.07.021. Epub 2018 Jul 18. PMID: 30010029.  213: Mine T, Matsueda S, Gao H, Li Y, Wong KK, Peoples GE, Ferrone S, IoannidesCG. Created Gli-1 duplex short-RNA (i-Gli-RNA) eliminates CD44 Hi progenitors oftaxol-resistant ovarian cancer cells. Oncol Rep. 2010 Jun;23(6):1537-43. doi:10.3892/or_00000793. PMID: 20428807; PMCID: PMC3426036.  214: Kapodistrias N, Mavridis K, Batistatou A, Gogou P, Karavasilis V, Sainis I,Briasoulis E, Scorilas A. Assessing the clinical value of microRNAs in formalin-fixed paraffin-embedded liposarcoma tissues: Overexpressed miR-155 is anindicator of poor prognosis. Oncotarget. 2017 Jan 24;8(4):6896-6913. doi:10.18632/oncotarget.14320. PMID: 28036291; PMCID: PMC5351678.  215: Wang YH, Xiong J, Wang SF, Yu Y, Wang B, Chen YX, Shi HF, Qiu Y.Lentivirus-mediated shRNA targeting insulin-like growth factor-1 receptor(IGF-1R) enhances chemosensitivity of osteosarcoma cells in vitro and in vivo.Mol Cell Biochem. 2010 Aug;341(1-2):225-33. doi: 10.1007/s11010-010-0453-2. Epub2010 Apr 8. PMID: 20376536.  216: Ma W, Zhang X, Chai J, Chen P, Ren P, Gong M. Circulating miR-148a is asignificant diagnostic and prognostic biomarker for patients with osteosarcoma.Tumour Biol. 2014 Dec;35(12):12467-72. doi: 10.1007/s13277-014-2565-x. Epub 2014Sep 4. PMID: 25185654.  217: Takigami I, Ohno T, Kitade Y, Hara A, Nagano A, Kawai G, Saitou M,Matsuhashi A, Yamada K, Shimizu K. Synthetic siRNA targeting the breakpoint ofEWS/Fli-1 inhibits growth of Ewing sarcoma xenografts in a mouse model. Int JCancer. 2011 Jan 1;128(1):216-26. doi: 10.1002/ijc.25564. PMID: 20648560.  218: Li PC, Tu MJ, Ho PY, Jilek JL, Duan Z, Zhang QY, Yu AX, Yu AM.Bioengineered NRF2-siRNA Is Effective to Interfere with NRF2 Pathways andImprove Chemosensitivity of Human Cancer Cells. Drug Metab Dispos. 2018Jan;46(1):2-10. doi: 10.1124/dmd.117.078741. Epub 2017 Oct 23. PMID: 29061583;PMCID: PMC5733456.  219: Kikuchi K, Soundararajan A, Zarzabal LA, Weems CR, Nelon LD, Hampton ST,Michalek JE, Rubin BP, Fields AP, Keller C. Protein kinase C iota as atherapeutic target in alveolar rhabdomyosarcoma. Oncogene. 2013 Jan17;32(3):286-95. doi: 10.1038/onc.2012.46. Epub 2012 Feb 20. PMID: 22349825;PMCID: PMC3360112.  220: Zhu MJ, Ou WB, Fletcher CD, Cohen PS, Demetri GD, Fletcher JA. KIToncoprotein interactions in gastrointestinal stromal tumors: therapeuticrelevance. Oncogene. 2007 Sep 27;26(44):6386-95. doi: 10.1038/sj.onc.1210464.Epub 2007 Apr 23. PMID: 17452978.  221: Carmo CR, Lyons-Lewis J, Seckl MJ, Costa-Pereira AP. A novel requirementfor Janus kinases as mediators of drug resistance induced by fibroblast growthfactor-2 in human cancer cells. PLoS One. 2011;6(5):e19861. doi:10.1371/journal.pone.0019861. Epub 2011 May 20. PMID: 21625473; PMCID:PMC3098828.  222: Duan Z, Choy E, Harmon D, Yang C, Ryu K, Schwab J, Mankin H, Hornicek FJ.Insulin-like growth factor-I receptor tyrosine kinase inhibitor cyclolignanpicropodophyllin inhibits proliferation and induces apoptosis in multidrugresistant osteosarcoma cell lines. Mol Cancer Ther. 2009 Aug;8(8):2122-30. doi:10.1158/1535-7163.MCT-09-0115. Epub 2009 Jul 28. PMID: 19638450; PMCID:PMC2766237.  223: Bersani F, Lingua MF, Morena D, Foglizzo V, Miretti S, Lanzetti L, Carrà G,Morotti A, Ala U, Provero P, Chiarle R, Singer S, Ladanyi M, Tuschl T, PonzettoC, Taulli R. Deep Sequencing Reveals a Novel miR-22 Regulatory Network withTherapeutic Potential in Rhabdomyosarcoma. Cancer Res. 2016 Oct15;76(20):6095-6106. doi: 10.1158/0008-5472.CAN-16-0709. Epub 2016 Aug 28. PMID:27569217; PMCID: PMC5207039.  224: Kim DW, Kim KO, Shin MJ, Ha JH, Seo SW, Yang J, Lee FY. siRNA-basedtargeting of antiapoptotic genes can reverse chemoresistance in P-glycoproteinexpressing chondrosarcoma cells. Mol Cancer. 2009 May 15;8:28. doi:10.1186/1476-4598-8-28. PMID: 19445670; PMCID: PMC2689171.  225: Pashler AL, Towler BP, Jones CI, Haime HJ, Burgess T, Newbury SF. Genome-wide analyses of XRN1-sensitive targets in osteosarcoma cells identify disease-relevant transcripts containing G-rich motifs. RNA. 2021 Oct;27(10):1265-1280.doi: 10.1261/rna.078872.121. Epub 2021 Jul 15. PMID: 34266995; PMCID:PMC8457002.  226: Kubota D, Mukaihara K, Yoshida A, Tsuda H, Kawai A, Kondo T. Proteomicsstudy of open biopsy samples identifies peroxiredoxin 2 as a predictivebiomarker of response to induction chemotherapy in osteosarcoma. J Proteomics.2013 Oct 8;91:393-404. doi: 10.1016/j.jprot.2013.07.022. Epub 2013 Aug 2. PMID:23911960.  227: Haynes KA, Ceroni F, Flicker D, Younger A, Silver PA. A sensitive switchfor visualizing natural gene silencing in single cells. ACS Synth Biol. 2012 Mar16;1(3):99-106. doi: 10.1021/sb3000035. PMID: 22530199; PMCID: PMC3331714.  228: Miller RC, Murley JS, Rademaker AW, Woloschak GE, Li JJ, Weichselbaum RR,Grdina DJ. Very low doses of ionizing radiation and redox associated modifiersaffect survivin-associated changes in radiation sensitivity. Free Radic BiolMed. 2016 Oct;99:110-119. doi: 10.1016/j.freeradbiomed.2016.07.009. Epub 2016Jul 15. PMID: 27427516; PMCID: PMC6764831.  229: Mariño-Enríquez A, Ou WB, Cowley G, Luo B, Jonker AH, Mayeda M, Okamoto M,Eilers G, Czaplinski JT, Sicinska E, Wang Y, Taguchi T, Demetri GD, Root DE,Fletcher JA. Genome-wide functional screening identifies CDC37 as a crucialHSP90-cofactor for KIT oncogenic expression in gastrointestinal stromal tumors.Oncogene. 2014 Apr 3;33(14):1872-6. doi: 10.1038/onc.2013.127. Epub 2013 Apr 15.PMID: 23584476; PMCID: PMC4310725.  230: Zhang F, Chen A, Chen J, Yu T, Guo F. SiRNA-mediated silencing of beta-catenin suppresses invasion and chemosensitivity to doxorubicin in MG-63osteosarcoma cells. Asian Pac J Cancer Prev. 2011;12(1):239-45. PMID: 21517265.  231: Qu W, Li D, Wang Y, Wu Q, Hao D. Activation of Sonic Hedgehog Signaling IsAssociated with Human Osteosarcoma Cells Radioresistance Characterized byIncreased Proliferation, Migration, and Invasion. Med Sci Monit. 2018 Jun4;24:3764-3771. doi: 10.12659/MSM.908278. PMID: 29864766; PMCID: PMC6016436.  232: Kim DW, Seo SW, Cho SK, Chang SS, Lee HW, Lee SE, Block JA, Hei TK, Lee FY.Targeting of cell survival genes using small interfering RNAs (siRNAs) enhancesradiosensitivity of Grade II chondrosarcoma cells. J Orthop Res. 2007Jun;25(6):820-8. doi: 10.1002/jor.20377. PMID: 17343283.  233: McDermott U, Ames RY, Iafrate AJ, Maheswaran S, Stubbs H, Greninger P,McCutcheon K, Milano R, Tam A, Lee DY, Lucien L, Brannigan BW, Ulkus LE, Ma XJ,Erlander MG, Haber DA, Sharma SV, Settleman J. Ligand-dependent platelet-derivedgrowth factor receptor (PDGFR)-alpha activation sensitizes rare lung cancer andsarcoma cells to PDGFR kinase inhibitors. Cancer Res. 2009 May 1;69(9):3937-46.doi: 10.1158/0008-5472.CAN-08-4327. Epub 2009 Apr 14. PMID: 19366796; PMCID:PMC2676215.  234: Creighton CJ, Fountain MD, Yu Z, Nagaraja AK, Zhu H, Khan M, Olokpa E,Zariff A, Gunaratne PH, Matzuk MM, Anderson ML. Molecular profiling uncovers ap53-associated role for microRNA-31 in inhibiting the proliferation of serousovarian carcinomas and other cancers. Cancer Res. 2010 Mar 1;70(5):1906-15. doi:10.1158/0008-5472.CAN-09-3875. Epub 2010 Feb 23. PMID: 20179198; PMCID:PMC2831102.  235: Miyachi M, Tsuchiya K, Yoshida H, Yagyu S, Kikuchi K, Misawa A, Iehara T,Hosoi H. Circulating muscle-specific microRNA, miR-206, as a potentialdiagnostic marker for rhabdomyosarcoma. Biochem Biophys Res Commun. 2010 Sep10;400(1):89-93. doi: 10.1016/j.bbrc.2010.08.015. Epub 2010 Aug 7. PMID:20696132.  236: Uboldi S, Calura E, Beltrame L, Fuso Nerini I, Marchini S, Cavalieri D,Erba E, Chiorino G, Ostano P, D'Angelo D, D'Incalci M, Romualdi C. A systemsbiology approach to characterize the regulatory networks leading to trabectedinresistance in an in vitro model of myxoid liposarcoma. PLoS One.2012;7(4):e35423. doi: 10.1371/journal.pone.0035423. Epub 2012 Apr 16. PMID:22523595; PMCID: PMC3327679.  237: Zhao Y, Xu K, Liu P. Post-Transcriptional Control of Angiotensin II Type 1Receptor Regulates Osteosarcoma Cell Death. Cell Physiol Biochem.2018;45(4):1581-1589. doi: 10.1159/000487719. Epub 2018 Feb 21. PMID: 29482191.  238: Yang S, Quaresma AJ, Nickerson JA, Green KM, Shaffer SA, Imbalzano AN,Martin-Buley LA, Lian JB, Stein JL, van Wijnen AJ, Stein GS. Subnuclear domainproteins in cancer cells support the functions of RUNX2 in the DNA damageresponse. J Cell Sci. 2015 Feb 15;128(4):728-40. doi: 10.1242/jcs.160051. Epub2015 Jan 20. PMID: 25609707; PMCID: PMC4327387.  239: Dong P, Kaneuchi M, Watari H, Hamada J, Sudo S, Ju J, Sakuragi N.MicroRNA-194 inhibits epithelial to mesenchymal transition of endometrial cancercells by targeting oncogene BMI-1. Mol Cancer. 2011 Aug 18;10:99. doi:10.1186/1476-4598-10-99. PMID: 21851624; PMCID: PMC3173388.  240: Chen CF, He X, Arslan AD, Mo YY, Reinhold WC, Pommier Y, Beck WT. Novelregulation of nuclear factor-YB by miR-485-3p affects the expression of DNAtopoisomerase IIα and drug responsiveness. Mol Pharmacol. 2011 Apr;79(4):735-41.doi: 10.1124/mol.110.069633. Epub 2011 Jan 20. PMID: 21252292; PMCID:PMC3063729.  241: Tang X, Yuan F, Guo K. Repair of radiation damage of U2OS osteosarcomacells is related to DNA-dependent protein kinase catalytic subunit (DNA-PKcs)activity. Mol Cell Biochem. 2014 May;390(1-2):51-9. doi:10.1007/s11010-013-1955-5. Epub 2014 Jan 5. PMID: 24390088.  242: Lee DH, Forscher C, Di Vizio D, Koeffler HP. Induction of p53-independentapoptosis by ectopic expression of HOXA5 in human liposarcomas. Sci Rep. 2015Jul 29;5:12580. doi: 10.1038/srep12580. PMID: 26219418; PMCID: PMC4518222.  243: Izeradjene K, Douglas L, Delaney A, Houghton JA. Influence of casein kinaseII in tumor necrosis factor-related apoptosis-inducing ligand-induced apoptosisin human rhabdomyosarcoma cells. Clin Cancer Res. 2004 Oct 1;10(19):6650-60.doi: 10.1158/1078-0432.CCR-04-0576. PMID: 15475455.  244: Benassi MS, Chiechi A, Ponticelli F, Pazzaglia L, Gamberi G, Zanella L,Manara MC, Perego P, Ferrari S, Picci P. Growth inhibition and sensitization tocisplatin by zoledronic acid in osteosarcoma cells. Cancer Lett. 2007 Jun8;250(2):194-205. doi: 10.1016/j.canlet.2006.10.004. Epub 2006 Nov 20. PMID:17113707.  245: Gobble RM, Qin LX, Brill ER, Angeles CV, Ugras S, O'Connor RB, Moraco NH,Decarolis PL, Antonescu C, Singer S. Expression profiling of liposarcoma yieldsa multigene predictor of patient outcome and identifies genes that contribute toliposarcomagenesis. Cancer Res. 2011 Apr 1;71(7):2697-705. doi:10.1158/0008-5472.CAN-10-3588. Epub 2011 Feb 18. PMID: 21335544; PMCID:PMC3070774.  246: Imam JS, Buddavarapu K, Lee-Chang JS, Ganapathy S, Camosy C, Chen Y, RaoMK. MicroRNA-185 suppresses tumor growth and progression by targeting the Six1oncogene in human cancers. Oncogene. 2010 Sep 2;29(35):4971-9. doi:10.1038/onc.2010.233. Epub 2010 Jul 5. PMID: 20603620.  247: Zimmerman KM, Jones RM, Petermann E, Jeggo PA. Diminished origin-licensingcapacity specifically sensitizes tumor cells to replication stress. Mol CancerRes. 2013 Apr;11(4):370-80. doi: 10.1158/1541-7786.MCR-12-0491. Epub 2013 Jan30. PMID: 23364533; PMCID: PMC3797919.  248: Jaaks P, Meier G, Alijaj N, Brack E, Bode P, Koscielniak E, Wachtel M,Schäfer BW, Bernasconi M. The proprotein convertase furin is required tomaintain viability of alveolar rhabdomyosarcoma cells. Oncotarget. 2016 Nov22;7(47):76743-76755. doi: 10.18632/oncotarget.11648. PMID: 27572312; PMCID:PMC5363546.  249: Stratford EW, Daffinrud J, Munthe E, Castro R, Waaler J, Krauss S,Myklebost O. The tankyrase-specific inhibitor JW74 affects cell cycleprogression and induces apoptosis and differentiation in osteosarcoma celllines. Cancer Med. 2014 Feb;3(1):36-46. doi: 10.1002/cam4.170. Epub 2013 Dec 17.PMID: 24403055; PMCID: PMC3930388.  250: Chuang TD, Ho M, Khorram O. The regulatory function of miR-200c oninflammatory and cell-cycle associated genes in SK-LMS-1, a leiomyosarcoma cellline. Reprod Sci. 2015 May;22(5):563-71. doi: 10.1177/1933719114553450. Epub2014 Oct 9. PMID: 25305131.  251: Rømer MU, Larsen L, Offenberg H, Brünner N, Lademann UA. Plasminogenactivator inhibitor 1 protects fibrosarcoma cells from etoposide-inducedapoptosis through activation of the PI3K/Akt cell survival pathway. Neoplasia.2008 Oct;10(10):1083-91. doi: 10.1593/neo.08486. PMID: 18813358; PMCID:PMC2546595.  252: Nikitovic D, Berdiaki A, Zafiropoulos A, Katonis P, Tsatsakis A, KaramanosNK, Tzanakakis GN. Lumican expression is positively correlated with thedifferentiation and negatively with the growth of human osteosarcoma cells. FEBSJ. 2008 Jan;275(2):350-61. doi: 10.1111/j.1742-4658.2007.06205.x. Epub 2007 Dec17. PMID: 18093185.  253: Du JY, Wang LF, Wang Q, Yu LD. miR-26b inhibits proliferation, migration,invasion and apoptosis induction via the downregulation of6-phosphofructo-2-kinase/fructose-2,6-bisphosphatase-3 driven glycolysis inosteosarcoma cells. Oncol Rep. 2015 Apr;33(4):1890-8. doi: 10.3892/or.2015.3797.Epub 2015 Feb 12. PMID: 25672572.  254: Wichmann H, Güttler A, Bache M, Taubert H, Vetter M, Würl P, Holzhausen HJ,Eckert AW, Kappler M, Vordermark D. Inverse prognostic impact of ErbB2 mRNA andprotein expression level in tumors of soft tissue sarcoma patients. StrahlentherOnkol. 2014 Oct;190(10):912-8. doi: 10.1007/s00066-014-0655-8. Epub 2014 Apr 9.PMID: 24715245.  255: Huang G, Nishimoto K, Zhou Z, Hughes D, Kleinerman ES. miR-20a encoded bythe miR-17-92 cluster increases the metastatic potential of osteosarcoma cellsby regulating Fas expression. Cancer Res. 2012 Feb 15;72(4):908-16. doi:10.1158/0008-5472.CAN-11-1460. Epub 2011 Dec 20. PMID: 22186140; PMCID:PMC3288434.  256: Di Maira G, Brustolon F, Bertacchini J, Tosoni K, Marmiroli S, Pinna LA,Ruzzene M. Pharmacological inhibition of protein kinase CK2 reverts themultidrug resistance phenotype of a CEM cell line characterized by high CK2level. Oncogene. 2007 Oct 18;26(48):6915-26. doi: 10.1038/sj.onc.1210495. Epub2007 May 7. PMID: 17486073.  257: Ohnuma-Ishikawa K, Morio T, Yamada T, Sugawara Y, Ono M, Nagasawa M, YasudaA, Morimoto C, Ohnuma K, Dang NH, Hosoi H, Verdin E, Mizutani S. Knockdown ofXAB2 enhances all-trans retinoic acid-induced cellular differentiation in all-trans retinoic acid-sensitive and -resistant cancer cells. Cancer Res. 2007 Feb1;67(3):1019-29. doi: 10.1158/0008-5472.CAN-06-1638. PMID: 17283134.  258: Kook I, Ziegelbauer JM. Monocyte chemoattractant protein-induced protein 1directly degrades viral miRNAs with a specific motif and inhibits KSHVinfection. Nucleic Acids Res. 2021 May 7;49(8):4456-4471. doi:10.1093/nar/gkab215. PMID: 33823555; PMCID: PMC8096232.  259: Zeng FY, Cui J, Liu L, Chen T. PAX3-FKHR sensitizes human alveolarrhabdomyosarcoma cells to camptothecin-mediated growth inhibition and apoptosis.Cancer Lett. 2009 Nov 1;284(2):157-64. doi: 10.1016/j.canlet.2009.04.016. Epub2009 May 12. PMID: 19442434; PMCID: PMC2739888.  260: Ozaki T, Sugimoto H, Nakamura M, Hiraoka K, Yoda H, Sang M, Fujiwara K,Nagase H. Runt-related transcription factor 2 attenuates the transcriptionalactivity as well as DNA damage-mediated induction of pro-apoptotic TAp73 toregulate chemosensitivity. FEBS J. 2015 Jan;282(1):114-28. doi:10.1111/febs.13108. Epub 2014 Nov 10. PMID: 25331851; PMCID: PMC4368372.  261: Hannay JA, Liu J, Zhu QS, Bolshakov SV, Li L, Pisters PW, Lazar AJ, Yu D,Pollock RE, Lev D. Rad51 overexpression contributes to chemoresistance in humansoft tissue sarcoma cells: a role for p53/activator protein 2 transcriptionalregulation. Mol Cancer Ther. 2007 May;6(5):1650-60. doi:10.1158/1535-7163.MCT-06-0636. PMID: 17513613.  262: Funasaka T, Hu H, Hogan V, Raz A. Down-regulation of phosphoglucoseisomerase/autocrine motility factor expression sensitizes human fibrosarcomacells to oxidative stress leading to cellular senescence. J Biol Chem. 2007 Dec14;282(50):36362-9. doi: 10.1074/jbc.M706301200. Epub 2007 Oct 9. PMID:17925402.  263: Chang Y, Zhao Y, Gu W, Cao Y, Wang S, Pang J, Shi Y. Bufalin Inhibits theDifferentiation and Proliferation of Cancer Stem Cells Derived from PrimaryOsteosarcoma Cells through Mir-148a. Cell Physiol Biochem. 2015;36(3):1186-96.doi: 10.1159/000430289. Epub 2015 Jun 25. PMID: 26111756.  264: Sokolova M, Turunen M, Mortusewicz O, Kivioja T, Herr P, Vähärautio A,Björklund M, Taipale M, Helleday T, Taipale J. Genome-wide screen of cell-cycleregulators in normal and tumor cells identifies a differential response tonucleosome depletion. Cell Cycle. 2017 Jan 17;16(2):189-199. doi:10.1080/15384101.2016.1261765. Epub 2016 Dec 8. PMID: 27929715; PMCID:PMC5283814.  265: Singh TR, Ali AM, Busygina V, Raynard S, Fan Q, Du CH, Andreassen PR, SungP, Meetei AR. BLAP18/RMI2, a novel OB-fold-containing protein, is an essentialcomponent of the Bloom helicase-double Holliday junction dissolvasome. GenesDev. 2008 Oct 15;22(20):2856-68. doi: 10.1101/gad.1725108. PMID: 18923083;PMCID: PMC2569884.  266: Meschini S, Condello M, Calcabrini A, Marra M, Formisano G, Lista P, DeMilito A, Federici E, Arancia G. The plant alkaloid voacamine induces apoptosis-independent autophagic cell death on both sensitive and multidrug resistanthuman osteosarcoma cells. Autophagy. 2008 Nov;4(8):1020-33. doi:10.4161/auto.6952. Epub 2008 Nov 9. PMID: 18838862.  267: Garnett TO, Filippova M, Duerksen-Hughes PJ. Bid is cleaved upstream ofcaspase-8 activation during TRAIL-mediated apoptosis in human osteosarcomacells. Apoptosis. 2007 Jul;12(7):1299-315. doi: 10.1007/s10495-007-0058-8. PMID:17431792.  268: Gu W, Jia Z, Truong NP, Prasadam I, Xiao Y, Monteiro MJ. Polymernanocarrier system for endosome escape and timed release of siRNA with completegene silencing and cell death in cancer cells. Biomacromolecules. 2013 Oct14;14(10):3386-9. doi: 10.1021/bm401139e. Epub 2013 Sep 4. PMID: 23992391.  269: Piccoli M, Palazzolo G, Conforti E, Lamorte G, Papini N, Creo P, Fania C,Scaringi R, Bergante S, Tringali C, Roncoroni L, Mazzoleni S, Doneda L, Galli R,Venerando B, Tettamanti G, Gelfi C, Anastasia L. The synthetic purine reversineselectively induces cell death of cancer cells. J Cell Biochem. 2012Oct;113(10):3207-17. doi: 10.1002/jcb.24197. PMID: 22615034.  270: Date O, Katsura M, Ishida M, Yoshihara T, Kinomura A, Sueda T, Miyagawa K.Haploinsufficiency of RAD51B causes centrosome fragmentation and aneuploidy inhuman cells. Cancer Res. 2006 Jun 15;66(12):6018-24. doi:10.1158/0008-5472.CAN-05-2803. PMID: 16778173.  271: Li Y, Xian M, Yang B, Ying M, He Q. Inhibition of KLF4 by Statins ReversesAdriamycin-Induced Metastasis and Cancer Stemness in Osteosarcoma Cells. StemCell Reports. 2017 Jun 6;8(6):1617-1629. doi: 10.1016/j.stemcr.2017.04.025. Epub2017 May 25. Erratum in: Stem Cell Reports. 2020 May 12;14(5):989-990. PMID:28552603; PMCID: PMC5470096.  272: Michaelis M, Rothweiler F, Löschmann N, Sharifi M, Ghafourian T, Cinatl JJr. Enzastaurin inhibits ABCB1-mediated drug efflux independently of effects onprotein kinase C signalling and the cellular p53 status. Oncotarget. 2015 Jul10;6(19):17605-20. doi: 10.18632/oncotarget.2889. PMID: 25749379; PMCID:PMC4627332.  273: Wise-Draper TM, Allen HV, Jones EE, Habash KB, Matsuo H, Wells SI.Apoptosis inhibition by the human DEK oncoprotein involves interference with p53functions. Mol Cell Biol. 2006 Oct;26(20):7506-19. doi: 10.1128/MCB.00430-06.Epub 2006 Aug 7. PMID: 16894028; PMCID: PMC1636856.  274: Yue B, Ma JF, Yao G, Yang MD, Cheng H, Liu GY. Knockdown of neuropilin-1suppresses invasion, angiogenesis, and increases the chemosensitivity todoxorubicin in osteosarcoma cells - an in vitro study. Eur Rev Med PharmacolSci. 2014;18(12):1735-41. PMID: 24992616.  275: Cao J, Wei J, Yang P, Zhang T, Chen Z, He F, Wei F, Chen H, Hu H, Zhong J,Yang Z, Cai W, Li W, Wang Q. Genome-scale CRISPR-Cas9 knockout screening ingastrointestinal stromal tumor with Imatinib resistance. Mol Cancer. 2018 Aug13;17(1):121. doi: 10.1186/s12943-018-0865-2. PMID: 30103756; PMCID: PMC6090611.  276: Wang X, Yu Y, Zang L, Zhang P, Ma J, Chen D. Targeting Clusterin InducesApoptosis, Reduces Growth Ability and Invasion and Mediates Sensitivity toChemotherapy in Human Osteosarcoma Cells. Curr Pharm Biotechnol.2020;21(2):131-139. doi: 10.2174/1389201020666190821151120. PMID: 31433751.  277: Zhang C, Zhao Y, Zeng B. Enhanced chemosensitivity by simultaneouslyinhibiting cell cycle progression and promoting apoptosis of drug-resistantosteosarcoma MG63/DXR cells by targeting Cyclin D1 and Bcl-2. Cancer Biomark.2012;12(4):155-67. doi: 10.3233/CBM-130305. PMID: 23568006.  278: Hua J, Mutch DG, Herzog TJ. Stable suppression of MDR-1 gene using siRNAexpression vector to reverse drug resistance in a human uterine sarcoma cellline. Gynecol Oncol. 2005 Jul;98(1):31-8. doi: 10.1016/j.ygyno.2005.03.042.PMID: 15921732.  279: von Wronski MA, Harris LC, Tano K, Mitra S, Bigner DD, Brent TP. Cytosinemethylation and suppression of O6-methylguanine-DNA methyltransferase expressionin human rhabdomyosarcoma cell lines and xenografts. Oncol Res.1992;4(4-5):167-74. PMID: 1504376.  280: He J, Zhang J, Wang Y, Liu W, Gou K, Liu Z, Cui S. MiR-7 Mediates theZearalenone Signaling Pathway Regulating FSH Synthesis and Secretion byTargeting FOS in Female Pigs. Endocrinology. 2018 Aug 1;159(8):2993-3006. doi:10.1210/en.2018-00097. PMID: 29796618.  281: Watanabe K, Okamoto K, Yonehara S. Sensitization of osteosarcoma cells todeath receptor-mediated apoptosis by HDAC inhibitors through downregulation ofcellular FLIP. Cell Death Differ. 2005 Jan;12(1):10-8. doi:10.1038/sj.cdd.4401507. PMID: 15540114.  282: du Rieu MC, Torrisani J, Selves J, Al Saati T, Souque A, Dufresne M,Tsongalis GJ, Suriawinata AA, Carrère N, Buscail L, Cordelier P. MicroRNA-21 isinduced early in pancreatic ductal adenocarcinoma precursor lesions. Clin Chem.2010 Apr;56(4):603-12. doi: 10.1373/clinchem.2009.137364. Epub 2010 Jan 21.PMID: 20093556.  283: Zhu M, Wei Y, Geißler C, Abschlag K, Corbalán Campos J, Hristov M, MöllmannJ, Lehrke M, Karshovska E, Schober A. Hyperlipidemia-Induced MicroRNA-155-5pImproves β-Cell Function by Targeting <i>Mafb</i>. Diabetes. 2017Dec;66(12):3072-3084. doi: 10.2337/db17-0313. Epub 2017 Sep 29. PMID: 28970282.  284: Xu HY, Fang W, Huang ZW, Lu JC, Wang YQ, Tang QL, Song GH, Kang Y, Zhu XJ,Zou CY, Yang HL, Shen JN, Wang J. Metformin reduces SATB2-mediated osteosarcomastem cell-like phenotype and tumor growth via inhibition of N-cadherin/NF-kBsignaling. Eur Rev Med Pharmacol Sci. 2017 Oct;21(20):4516-4528. PMID: 29131265.  285: Wu J, Zeng T, Wu X, Gao Q, Zhai W, Ding Z. Ether à go-go 1 silencing incombination with TRAIL overexpression has synergistic antitumor effects onosteosarcoma. Cancer Biother Radiopharm. 2013 Feb;28(1):65-70. doi:10.1089/cbr.2012.1283. Epub 2012 Nov 12. PMID: 23145797.  286: Wang D, Zhong ZY, Li MX, Xiang DB, Li ZP. Vector-based Ape1 smallinterfering RNA enhances the sensitivity of human osteosarcoma cells toendostatin in vivo. Cancer Sci. 2007 Dec;98(12):1993-2001. doi:10.1111/j.1349-7006.2007.00616.x. Epub 2007 Sep 24. PMID: 17892509.  287: Kasiappan R, Jutooru I, Mohankumar K, Karki K, Lacey A, Safe S. ReactiveOxygen Species (ROS)-Inducing Triterpenoid Inhibits Rhabdomyosarcoma Cell andTumor Growth through Targeting Sp Transcription Factors. Mol Cancer Res. 2019Mar;17(3):794-805. doi: 10.1158/1541-7786.MCR-18-1071. Epub 2019 Jan 4. PMID:30610105; PMCID: PMC6397684.  288: Papoutsidakis A, Giatagana EM, Berdiaki A, Spyridaki I, Spandidos DA,Tsatsakis A, Tzanakakis GN, Nikitovic D. Lumican mediates HTB94 chondrosarcomacell growth via an IGF‑IR/Erk1/2 axis. Int J Oncol. 2020 Sep;57(3):791-803. doi:10.3892/ijo.2020.5094. Epub 2020 Jul 6. PMID: 32705211; PMCID: PMC7384848.  289: Hasei J, Sasaki T, Tazawa H, Osaki S, Yamakawa Y, Kunisada T, Yoshida A,Hashimoto Y, Onishi T, Uno F, Kagawa S, Urata Y, Ozaki T, Fujiwara T. Dualprogrammed cell death pathways induced by p53 transactivation overcomeresistance to oncolytic adenovirus in human osteosarcoma cells. Mol Cancer Ther.2013 Mar;12(3):314-25. doi: 10.1158/1535-7163.MCT-12-0869. Epub 2013 Jan 11.Erratum in: Mol Cancer Ther. 2013 Sep;12(9):1920-1. PMID: 23315976.  290: Yang D, Wang P, Ren X. Apoptosis induced by chamaejasmine in humanosteosarcoma cells through p53 pathway. Tumour Biol. 2015 Jul;36(7):5433-9. doi:10.1007/s13277-015-3209-5. Epub 2015 Feb 14. PMID: 25677904.  291: Duffy S, Fam HK, Wang YK, Styles EB, Kim JH, Ang JS, Singh T, Larionov V,Shah SP, Andrews B, Boerkoel CF, Hieter P. Overexpression screens identifyconserved dosage chromosome instability genes in yeast and human cancer. ProcNatl Acad Sci U S A. 2016 Sep 6;113(36):9967-76. doi: 10.1073/pnas.1611839113.Epub 2016 Aug 22. PMID: 27551064; PMCID: PMC5018746.  292: Tanaka H, Hoshikawa Y, Oh-hara T, Koike S, Naito M, Noda T, Arai H, TsuruoT, Fujita N. PRMT5, a novel TRAIL receptor-binding protein, inhibits TRAIL-induced apoptosis via nuclear factor-kappaB activation. Mol Cancer Res. 2009Apr;7(4):557-69. doi: 10.1158/1541-7786.MCR-08-0197. PMID: 19372584.  293: Gravina GL, Festuccia C, Popov VM, Di Rocco A, Colapietro A, Sanità P,Monache SD, Musio D, De Felice F, Di Cesare E, Tombolini V, Marampon F. c-MycSustains Transformed Phenotype and Promotes Radioresistance of EmbryonalRhabdomyosarcoma Cell Lines. Radiat Res. 2016 Apr;185(4):411-22. doi:10.1667/RR14237.1. PMID: 27104757.  294: Filleur S, Courtin A, Ait-Si-Ali S, Guglielmi J, Merle C, Harel-Bellan A,Clézardin P, Cabon F. SiRNA-mediated inhibition of vascular endothelial growthfactor severely limits tumor resistance to antiangiogenic thrombospondin-1 andslows tumor vascularization and growth. Cancer Res. 2003 Jul 15;63(14):3919-22.PMID: 12873985.  295: Zhao Z, Wu MS, Zou C, Tang Q, Lu J, Liu D, Wu Y, Yin J, Xie X, Shen J, KangT, Wang J. Downregulation of MCT1 inhibits tumor growth, metastasis and enhanceschemotherapeutic efficacy in osteosarcoma through regulation of the NF-κBpathway. Cancer Lett. 2014 Jan 1;342(1):150-8. doi:10.1016/j.canlet.2013.08.042. Epub 2013 Sep 3. Erratum in: Cancer Lett. 2021 Dec1;522:281. PMID: 24012639.  296: Kiran S, Oddi V, Ramakrishna G. Sirtuin 7 promotes cellular survivalfollowing genomic stress by attenuation of DNA damage, SAPK activation and p53response. Exp Cell Res. 2015 Feb 1;331(1):123-141. doi:10.1016/j.yexcr.2014.11.001. Epub 2014 Nov 12. PMID: 25445786.  297: Zhang T, Kastrenopoulou A, Larrouture Q, Athanasou NA, Knowles HJ.Angiopoietin-like 4 promotes osteosarcoma cell proliferation and migration andstimulates osteoclastogenesis. BMC Cancer. 2018 May 8;18(1):536. doi:10.1186/s12885-018-4468-5. PMID: 29739381; PMCID: PMC5941625.  298: Lin P, Mobasher ME, Alawi F. Acute dyskerin depletion triggers cellularsenescence and renders osteosarcoma cells resistant to genotoxic stress-inducedapoptosis. Biochem Biophys Res Commun. 2014 Apr 18;446(4):1268-75. doi:10.1016/j.bbrc.2014.03.114. Epub 2014 Mar 29. PMID: 24690175; PMCID: PMC4096983.  299: Boll K, Reiche K, Kasack K, Mörbt N, Kretzschmar AK, Tomm JM, Verhaegh G,Schalken J, von Bergen M, Horn F, Hackermüller J. MiR-130a, miR-203 and miR-205jointly repress key oncogenic pathways and are downregulated in prostatecarcinoma. Oncogene. 2013 Jan 17;32(3):277-85. doi: 10.1038/onc.2012.55. Epub2012 Mar 5. PMID: 22391564.  300: Dietrich P, Kuphal S, Spruss T, Hellerbrand C, Bosserhoff AK. MicroRNA-622is a novel mediator of tumorigenicity in melanoma by targeting Kirsten ratsarcoma. Pigment Cell Melanoma Res. 2018 Sep;31(5):614-629. doi:10.1111/pcmr.12698. Epub 2018 Mar 25. PMID: 29495114.  301: Szpechcinski A, Florczuk M, Duk K, Zdral A, Rudzinski S, Bryl M, CzyzewiczG, Rudzinski P, Kupis W, Wojda E, Giedronowicz D, Langfort R, Barinow-WojewodzkiA, Orlowski T, Chorostowska-Wynimko J. The expression of circulating miR-504 inplasma is associated with EGFR mutation status in non-small-cell lung carcinomapatients. Cell Mol Life Sci. 2019 Sep;76(18):3641-3656. doi:10.1007/s00018-019-03089-2. Epub 2019 Apr 5. PMID: 30953094; PMCID: PMC6697756.  302: Blandamura S, Alessandrini L, Bertorelle R, Simonato F, Guzzardo V,Valentini E, Angriman I, Fassina A. Multiple sporadic gastrointestinal stromaltumors concomitant with ampullary adenocarcinoma: a case report with KIT andPDGFRA mutational analysis and miR-221/222 expression profile. Pathol Res Pract.2014 Jun;210(6):392-6. doi: 10.1016/j.prp.2014.01.019. Epub 2014 Mar 5. PMID:24674454.  303: Gravel S, Chapman JR, Magill C, Jackson SP. DNA helicases Sgs1 and BLMpromote DNA double-strand break resection. Genes Dev. 2008 Oct15;22(20):2767-72. doi: 10.1101/gad.503108. PMID: 18923075; PMCID: PMC2569880.  304: Zhang YP, Kong QH, Huang Y, Wang GL, Chang KJ. Inhibition of c-FLIP by RNAienhances sensitivity of the human osteogenic sarcoma cell line U2OS to TRAIL-induced apoptosis. Asian Pac J Cancer Prev. 2015;16(6):2251-6. doi:10.7314/apjcp.2015.16.6.2251. PMID: 25824746.  305: Hu Y, Li S, Yang M, Yan C, Fan D, Zhou Y, Zhang Y, Yagüe E, Xiong D. Sorcinsilencing inhibits epithelial-to-mesenchymal transition and suppresses breastcancer metastasis in vivo. Breast Cancer Res Treat. 2014 Jan;143(2):287-99. doi:10.1007/s10549-013-2809-2. Epub 2013 Dec 15. PMID: 24337682.  306: Kondelin J, Tuupanen S, Gylfe AE, Aavikko M, Renkonen-Sinisalo L, JärvinenH, Böhm J, Mecklin JP, Andersen CL, Vahteristo P, Pitkänen E, Aaltonen LA.3'-UTR poly(T/U) repeat of EWSR1 is altered in microsatellite unstablecolorectal cancer with nearly perfect sensitivity. Fam Cancer. 2015Sep;14(3):449-53. doi: 10.1007/s10689-015-9804-1. PMID: 25930744.  307: Moss EL, Mourtada-Maarabouni M, Pickard MR, Redman CW, Williams GT. FAUregulates carboplatin resistance in ovarian cancer. Genes Chromosomes Cancer.2010 Jan;49(1):70-7. doi: 10.1002/gcc.20721. PMID: 19830698.  308: Zhang D, Yu K, Yang Z, Li Y, Ma X, Bian X, Liu F, Li L, Liu X, Wu W.Silencing Ubc9 expression suppresses osteosarcoma tumorigenesis and enhanceschemosensitivity to HSV-TK/GCV by regulating connexin 43 SUMOylation. Int JOncol. 2018 Sep;53(3):1323-1331. doi: 10.3892/ijo.2018.4448. Epub 2018 Jun 21.PMID: 29956745.  309: Hou CH, Lin FL, Hou SM, Liu JF. Hyperthermia induces apoptosis throughendoplasmic reticulum and reactive oxygen species in human osteosarcoma cells.Int J Mol Sci. 2014 Sep 29;15(10):17380-95. doi: 10.3390/ijms151017380. PMID:25268613; PMCID: PMC4227168.  310: Deryugina EI, Zijlstra A, Partridge JJ, Kupriyanova TA, Madsen MA,Papagiannakopoulos T, Quigley JP. Unexpected effect of matrix metalloproteinasedown-regulation on vascular intravasation and metastasis of human fibrosarcomacells selected in vivo for high rates of dissemination. Cancer Res. 2005 Dec1;65(23):10959-69. doi: 10.1158/0008-5472.CAN-05-2228. PMID: 16322244.  311: Spina A, Sorvillo L, Di Maiolo F, Esposito A, D'Auria R, Di Gesto D, ChiosiE, Naviglio S. Inorganic phosphate enhances sensitivity of human osteosarcomaU2OS cells to doxorubicin via a p53-dependent pathway. J Cell Physiol. 2013Jan;228(1):198-206. doi: 10.1002/jcp.24124. PMID: 22674530.  312: Wiltshire TD, Lovejoy CA, Wang T, Xia F, O'Connor MJ, Cortez D. Sensitivityto poly(ADP-ribose) polymerase (PARP) inhibition identifies ubiquitin-specificpeptidase 11 (USP11) as a regulator of DNA double-strand break repair. J BiolChem. 2010 May 7;285(19):14565-71. doi: 10.1074/jbc.M110.104745. Epub 2010 Mar15. PMID: 20233726; PMCID: PMC2863164.  313: Liu F, Li L, Li Y, Ma X, Bian X, Liu X, Wang G, Zhang D. Overexpression ofSENP1 reduces the stemness capacity of osteosarcoma stem cells and increasestheir sensitivity to HSVtk/GCV. Int J Oncol. 2018 Nov;53(5):2010-2020. doi:10.3892/ijo.2018.4537. Epub 2018 Aug 23. Erratum in: Int J Oncol. 2019Jun;54(6):2258. PMID: 30226577; PMCID: PMC6192779.  314: Yang R, Qin J, Hoang BH, Healey JH, Gorlick R. Polymorphisms andmethylation of the reduced folate carrier in osteosarcoma. Clin Orthop RelatRes. 2008 Sep;466(9):2046-51. doi: 10.1007/s11999-008-0323-3. Epub 2008 Jun 5.PMID: 18528741; PMCID: PMC2493020.  315: Kawano M, Tanaka K, Itonaga I, Iwasaki T, Tsumura H. MicroRNA-181c preventsapoptosis by targeting of FAS receptor in Ewing's sarcoma cells. Cancer CellInt. 2018 Mar 12;18:37. doi: 10.1186/s12935-018-0536-9. PMID: 29563856; PMCID:PMC5848431.  316: Sergei B, Pavel D, Aigul G, Firyuza B, Ilmira N, Ilshat M, Aida A, Refat K,Natalia A, Elena S, Vera G. Inhibition of FGFR2-Signaling Attenuates a Homology-Mediated DNA Repair in GIST and Sensitizes Them to DNA-Topoisomerase IIInhibitors. Int J Mol Sci. 2020 Jan 5;21(1):352. doi: 10.3390/ijms21010352.PMID: 31948066; PMCID: PMC6982350.  317: Vanhaesebroeck B, Decoster E, Van Ostade X, Van Bladel S, Lenaerts A, VanRoy F, Fiers W. Expression of an exogenous tumor necrosis factor (TNF) gene inTNF-sensitive cell lines confers resistance to TNF-mediated cell lysis. JImmunol. 1992 May 1;148(9):2785-94. PMID: 1374099.  318: Wijdeven RH, Pang B, van der Zanden SY, Qiao X, Blomen V, Hoogstraat M,Lips EH, Janssen L, Wessels L, Brummelkamp TR, Neefjes J. Genome-WideIdentification and Characterization of Novel Factors Conferring Resistance toTopoisomerase II Poisons in Cancer. Cancer Res. 2015 Oct 1;75(19):4176-87. doi:10.1158/0008-5472.CAN-15-0380. Epub 2015 Aug 10. PMID: 26260527.  319: Wierdl M, Tsurkan L, Chi L, Hatfield MJ, Tollemar V, Bradley C, Chen X, QuC, Potter PM. Targeting ALK in pediatric RMS does not induce antitumor activityin vivo. Cancer Chemother Pharmacol. 2018 Aug;82(2):251-263. doi:10.1007/s00280-018-3615-7. Epub 2018 May 31. PMID: 29855693; PMCID: PMC6054567.  320: Kobayashi E, Iyer AK, Hornicek FJ, Amiji MM, Duan Z. Lipid-functionalizeddextran nanosystems to overcome multidrug resistance in cancer: a pilot study.Clin Orthop Relat Res. 2013 Mar;471(3):915-25. doi: 10.1007/s11999-012-2610-2.PMID: 23011844; PMCID: PMC3563790.  321: Oikawa K, Ishida T, Imamura T, Yoshida K, Takanashi M, Hattori H, IshikawaA, Fujita K, Yamamoto K, Matsubayashi J, Kuroda M, Mukai K. Generation of thenovel monoclonal antibody against TLS/EWS-CHOP chimeric oncoproteins that isapplicable to one of the most sensitive assays for myxoid and round cellliposarcomas. Am J Surg Pathol. 2006 Mar;30(3):351-6. doi:10.1097/01.pas.0000194043.01104.eb. PMID: 16538055.  322: Lee CG, Jeang KT, Martin MA, Pastan I, Gottesman MM. Efficient long-termcoexpression of a hammerhead ribozyme targeted to the U5 region of HIV-1 LTR bylinkage to the multidrug-resistance gene. Antisense Nucleic Acid Drug Dev. 1997Oct;7(5):511-22. doi: 10.1089/oli.1.1997.7.511. PMID: 9361910.  323: Berti M, Teloni F, Mijic S, Ursich S, Fuchs J, Palumbieri MD, Krietsch J,Schmid JA, Garcin EB, Gon S, Modesti M, Altmeyer M, Lopes M. Sequential role ofRAD51 paralog complexes in replication fork remodeling and restart. Nat Commun.2020 Jul 15;11(1):3531. doi: 10.1038/s41467-020-17324-z. PMID: 32669601; PMCID:PMC7363682.  324: Wang B, Xu W, Cai Y, Liu K, Wu J, Guo C, Yuan C. The Functional Role ofOncogenic LncRNA BCAR4 for Cancer Outcome. Curr Pharm Des.2021;27(39):4107-4113. doi: 10.2174/1381612827666210604114955. PMID: 34086548.  325: Chitpatima ST, Brawerman G. Shifts in configuration of the 5'-noncodingregion of a mouse messenger RNA under translational control. J Biol Chem. 1988May 25;263(15):7164-9. PMID: 3130378.  326: Holland D, Hoppe-Seyler K, Schuller B, Lohrey C, Maroldt J, Dürst M, Hoppe-Seyler F. Activation of the enhancer of zeste homologue 2 gene by the humanpapillomavirus E7 oncoprotein. Cancer Res. 2008 Dec 1;68(23):9964-72. doi:10.1158/0008-5472.CAN-08-1134. Erratum in: Cancer Res. 2009 Apr 15;69(8):3721.PMID: 19047178.  327: Shi P, Li Y, Guo Q. Circular RNA circPIP5K1A contributes to cancer stemnessof osteosarcoma by miR-515-5p/YAP axis. J Transl Med. 2021 Nov 13;19(1):464.doi: 10.1186/s12967-021-03124-6. PMID: 34774083; PMCID: PMC8590363.  328: Krump-Konvalinkova V, Yasuda S, Rubic T, Makarova N, Mages J, Erl W,Vosseler C, Kirkpatrick CJ, Tigyi G, Siess W. Stable knock-down of thesphingosine 1-phosphate receptor S1P1 influences multiple functions of humanendothelial cells. Arterioscler Thromb Vasc Biol. 2005 Mar;25(3):546-52. doi:10.1161/01.ATV.0000154360.36106.d9. Epub 2004 Dec 23. PMID: 15618544.  329: Carragher NO, Walker SM, Scott Carragher LA, Harris F, Sawyer TK, BruntonVG, Ozanne BW, Frame MC. Calpain 2 and Src dependence distinguishes mesenchymaland amoeboid modes of tumour cell invasion: a link to integrin function.Oncogene. 2006 Sep 21;25(42):5726-40. doi: 10.1038/sj.onc.1209582. Epub 2006 May1. PMID: 16652152.  330: Watanabe T, Sullenger BA. Induction of wild-type p53 activity in humancancer cells by ribozymes that repair mutant p53 transcripts. Proc Natl Acad SciU S A. 2000 Jul 18;97(15):8490-4. doi: 10.1073/pnas.150104097. PMID: 10890910;PMCID: PMC26975.  331: Rothzerg E, Ho XD, Xu J, Wood D, Märtson A, Kõks S. Upregulation of 15Antisense Long Non-Coding RNAs in Osteosarcoma. Genes (Basel). 2021 Jul26;12(8):1132. doi: 10.3390/genes12081132. PMID: 34440306; PMCID: PMC8394133.  332: Kappler M, Taubert H, Bartel F, Blümke K, Panian M, Schmidt H, Dunst J,Bache M. Radiosensitization, after a combined treatment of survivin siRNA andirradiation, is correlated with the activation of caspases 3 and 7 in a wt-p53sarcoma cell line, but not in a mt-p53 sarcoma cell line. Oncol Rep. 2005Jan;13(1):167-72. PMID: 15583820.  333: Cagnon L, Rossi JJ. Downregulation of the CCR5 beta-chemokine receptor andinhibition of HIV-1 infection by stable VA1-ribozyme chimeric transcripts.Antisense Nucleic Acid Drug Dev. 2000 Aug;10(4):251-61. doi:10.1089/108729000421439. PMID: 10984119.  334: Zhong X, He X, Wang Y, Hu Z, Yu D, Huang H, Zhao S, Wei P, Li D. A Hypoxia-Related lncRNA Signature Correlates with Survival and Tumor Microenvironment in Colorectal Cancer. J Immunol Res. 2022. 2022:9935705. doi: 10.1155/2022/9935705. PMID: 35846431; PMCID: PMC9286950.  335: Yang X, Iyer AK, Singh A, Milane L, Choy E, Hornicek FJ, Amiji MM, Duan Z.Cluster of Differentiation 44 Targeted Hyaluronic Acid Based Nanoparticles forMDR1 siRNA Delivery to Overcome Drug Resistance in Ovarian Cancer. Pharm Res.2015 Jun;32(6):2097-109. doi: 10.1007/s11095-014-1602-1. Epub 2014 Dec 17. PMID:25515492; PMCID: PMC4425596.  336: Sorensen DR, Leirdal M, Iversen PO, Sioud M. Combination of endostatin anda protein kinase Calpha DNA enzyme improves the survival of rats with malignantglioma. Neoplasia. 2002 Nov-Dec;4(6):474-9. doi: 10.1038/sj.neo.7900271. PMID:12407440; PMCID: PMC1503660.  337: Xiao-Long M, Kun-Peng Z, Chun-Lin Z. Circular RNA circ_HIPK3 is down-regulated and suppresses cell proliferation, migration and invasion inosteosarcoma. J Cancer. 2018 Apr 26;9(10):1856-1862. doi: 10.7150/jca.24619.PMID: 29805712; PMCID: PMC5968774.  338: Wu I, Shin SC, Cao Y, Bender IK, Jafari N, Feng G, Lin S, Cidlowski JA,Schleimer RP, Lu NZ. Selective glucocorticoid receptor translational isoformsreveal glucocorticoid-induced apoptotic transcriptomes. Cell Death Dis. 2013 Jan10;4(1):e453. doi: 10.1038/cddis.2012.193. PMID: 23303127; PMCID: PMC3563981.  339: Guay D, Garand C, Reddy S, Schmutte C, Lebel M. The human endonuclease IIIenzyme is a relevant target to potentiate cisplatin cytotoxicity in Y-box-binding protein-1 overexpressing tumor cells. Cancer Sci. 2008 Apr;99(4):762-9.doi: 10.1111/j.1349-7006.2008.00739.x. Epub 2008 Feb 27. PMID: 18307537.  340: Vujic I, Posch C, Sanlorenzo M, Yen AJ, Tsumura A, Kwong A,Feichtenschlager V, Lai K, Arneson DV, Rappersberger K, Ortiz-Urda SM. MutantNRASQ61 shares signaling similarities across various cancer types--potentialimplications for future therapies. Oncotarget. 2014 Sep 15;5(17):7936-44. doi:10.18632/oncotarget.2326. PMID: 25277205; PMCID: PMC4202171.  341: Bayer A, Lennemann NJ, Ouyang Y, Sadovsky E, Sheridan MA, Roberts RM, CoyneCB, Sadovsky Y. Chromosome 19 microRNAs exert antiviral activity independentfrom type III interferon signaling. Placenta. 2018 Jan;61:33-38. doi:10.1016/j.placenta.2017.11.004. Epub 2017 Nov 10. PMID: 29277269; PMCID:PMC5745809.  342: Yang D, Wang S, Brooks C, Dong Z, Schoenlein PV, Kumar V, Ouyang X, XiongH, Lahat G, Hayes-Jordan A, Lazar A, Pollock R, Lev D, Liu K. IFN regulatoryfactor 8 sensitizes soft tissue sarcoma cells to death receptor-initiatedapoptosis via repression of FLICE-like protein expression. Cancer Res. 2009 Feb1;69(3):1080-8. doi: 10.1158/0008-5472.CAN-08-2520. Epub 2009 Jan 20. PMID:19155307; PMCID: PMC2633427.  343: West J, Damania B. Upregulation of the TLR3 pathway by Kaposi's sarcoma-associated herpesvirus during primary infection. J Virol. 2008Jun;82(11):5440-9. doi: 10.1128/JVI.02590-07. Epub 2008 Mar 26. PMID: 18367536;PMCID: PMC2395190.  344: Yang X, Iyer AK, Singh A, Choy E, Hornicek FJ, Amiji MM, Duan Z. MDR1 siRNAloaded hyaluronic acid-based CD44 targeted nanoparticle systems circumventpaclitaxel resistance in ovarian cancer. Sci Rep. 2015 Feb 17;5:8509. doi:10.1038/srep08509. PMID: 25687880; PMCID: PMC4330541.  345: Yanaihara N, Anglesio MS, Ochiai K, Hirata Y, Saito M, Nagata C, Iida Y,Takakura S, Yamada K, Tanaka T, Okamoto A. Cytokine gene expression signature inovarian clear cell carcinoma. Int J Oncol. 2012 Sep;41(3):1094-100. doi:10.3892/ijo.2012.1533. Epub 2012 Jun 26. PMID: 22751940.  346: Kishore S, Piscuoglio S, Kovac MB, Gylling A, Wenzel F, Trapani F,Altermatt HJ, Mele V, Marra G, Peltomäki P, Terracciano L, Zavolan M, HeinimannK. 3'-UTR poly(T/U) tract deletions and altered expression of EWSR1 are ahallmark of mismatch repair-deficient cancers. Cancer Res. 2014 Jan1;74(1):224-34. doi: 10.1158/0008-5472.CAN-13-2100. Epub 2013 Oct 24. PMID:24158095.  347: Grohar PJ, Kim S, Rangel Rivera GO, Sen N, Haddock S, Harlow ML, MaloneyNK, Zhu J, O'Neill M, Jones TL, Huppi K, Grandin M, Gehlhaus K, Klumpp-ThomasCA, Buehler E, Helman LJ, Martin SE, Caplen NJ. Functional Genomic ScreeningReveals Splicing of the EWS-FLI1 Fusion Transcript as a Vulnerability in EwingSarcoma. Cell Rep. 2016 Jan 26;14(3):598-610. doi: 10.1016/j.celrep.2015.12.063.Epub 2016 Jan 14. PMID: 26776507; PMCID: PMC4755295.  348: Lewis CW, Bukhari AB, Xiao EJ, Choi WS, Smith JD, Homola E, Mackey JR,Campbell SD, Gamper AM, Chan GK. Upregulation of Myt1 Promotes AcquiredResistance of Cancer Cells to Wee1 Inhibition. Cancer Res. 2019 Dec1;79(23):5971-5985. doi: 10.1158/0008-5472.CAN-19-1961. Epub 2019 Oct 8. PMID:31594837.  349: Albers J, Danzer C, Rechsteiner M, Lehmann H, Brandt LP, Hejhal T, CatalanoA, Busenhart P, Gonçalves AF, Brandt S, Bode PK, Bode-Lesniewska B, Wild PJ,Frew IJ. A versatile modular vector system for rapid combinatorial mammaliangenetics. J Clin Invest. 2015 Apr;125(4):1603-19. doi: 10.1172/JCI79743. Epub2015 Mar 9. PMID: 25751063; PMCID: PMC4396471.  350: Yu S, Shao F, Liu H, Liu Q. A five metastasis-related long noncoding RNArisk signature for osteosarcoma survival prediction. BMC Med Genomics. 2021 May8;14(1):124. doi: 10.1186/s12920-021-00972-5. PMID: 33964903; PMCID: PMC8105989.  351: Nair JS, Sheikh T, Ho AL, Schwartz GK. PTEN regulates sensitivity ofmelanoma cells to RO4929097, the γ-secretase inhibitor. Anticancer Res. 2013Apr;33(4):1307-16. PMID: 23564767.  352: Bednarski BK, Ding X, Coombe K, Baldwin AS, Kim HJ. Active roles forinhibitory kappaB kinases alpha and beta in nuclear factor-kappaB-mediatedchemoresistance to doxorubicin. Mol Cancer Ther. 2008 Jul;7(7):1827-35. doi:10.1158/1535-7163.MCT-08-0321. PMID: 18644995; PMCID: PMC2581801.  353: Blomqvist S, Savolainen-Kopra C, Paananen A, Hovi T, Roivainen M. Molecularcharacterization of human rhinovirus field strains isolated during surveillanceof enteroviruses. J Gen Virol. 2009 Jun;90(Pt 6):1371-1381. doi:10.1099/vir.0.008508-0. Epub 2009 Mar 4. PMID: 19264616.  354: Hu Y, Zhang X, Zai HY, Jiang W, Xiao L, Zhu Q. lncRNA DUXAP8 FacilitatesMultiple Malignant Phenotypes and Resistance to PARP Inhibitor in HCC viaUpregulating FOXM1. Mol Ther Oncolytics. 2020 Oct 22;19:308-322. doi:10.1016/j.omto.2020.10.010. PMID: 33313387; PMCID: PMC7701012.  355: Jones RE, DeFeo D, Piatigorsky J. Initial studies on cultured embryonicchick lens epithelial cells infected with a temperature-sensitive Rous sarcomavirus. Vision Res. 1981;21(1):5-9. doi: 10.1016/0042-6989(81)90130-9. PMID:6168103.  356: To RY, Booth SC, Turk G, Neiman PE. Completion of avian retroviral DNAreplication intermediates inhibited by antisense RNA. Virology. 1994 May1;200(2):336-46. doi: 10.1006/viro.1994.1198. PMID: 8178425.  357: Song HM, Meng D, Wang JP, Zhang XY. circRNA hsa_circ_0005909 Predicts PoorPrognosis and Promotes the Growth, Metastasis, and Drug Resistance of Non-Small-Cell Lung Cancer via the miRNA-338-3p/SOX4 Pathway. Dis Markers. 2021 Aug6;2021:8388512. doi: 10.1155/2021/8388512. PMID: 34413915; PMCID: PMC8369175.  358: Goda AE, Yoshida T, Horinaka M, Yasuda T, Shiraishi T, Wakada M, Sakai T.Mechanisms of enhancement of TRAIL tumoricidal activity against human cancercells of different origin by dipyridamole. Oncogene. 2008 May 29;27(24):3435-45.doi: 10.1038/sj.onc.1211008. Epub 2008 Jan 14. PMID: 18193086.  359: Qi M, Ganapathy S, Zeng W, Zhang J, Little JB, Yuan ZM. UXT, a novel MDMX-binding protein, promotes glycolysis by mitigating p53-mediated restriction ofNF-κB activity. Oncotarget. 2015 Jul 10;6(19):17584-93. doi:10.18632/oncotarget.3770. PMID: 25974965; PMCID: PMC4627330.  360: Blum W, Schwaller B. Calretinin is essential for mesothelioma cellgrowth/survival in vitro: a potential new target for malignant mesotheliomatherapy? Int J Cancer. 2013 Nov;133(9):2077-88. doi: 10.1002/ijc.28218. Epub2013 Jul 9. PMID: 23595591.  361: Claveau S, Kindermann M, Papine A, Díaz-Riascos ZV, Délen X, Georges P,López-Alemany R, Tirado ÒM, Bertrand JR, Abasolo I, Cigler P, Treussart F.Harnessing subcellular-resolved organ distribution of cationic copolymer-functionalized fluorescent nanodiamonds for optimal delivery of active siRNA toa xenografted tumor in mice. Nanoscale. 2021 May 27;13(20):9280-9292. doi:10.1039/d1nr00146a. PMID: 33982741.  362: Boddul SV, Meng J, Dolly JO, Wang J. SNAP-23 and VAMP-3 contribute to therelease of IL-6 and TNFα from a human synovial sarcoma cell line. FEBS J. 2014Feb;281(3):750-65. doi: 10.1111/febs.12620. Epub 2013 Dec 13. PMID: 24373201.  363: Pan S, Wang Q, Zhang Q, Zhou M, Li L, Zhou X. A novel circular RNA,circPUS7 promotes cadmium-induced transformation of human bronchial epithelialcells by regulating Kirsten rat sarcoma viral oncogene homolog expression viasponging miR-770. Metallomics. 2021 Jul 24;13(7):mfab043. doi:10.1093/mtomcs/mfab043. PMID: 34232319.  364: Zhu X, Ouyang Y, Zhong F, Wang Q, Ding L, Zhang P, Chen L, Liu H, He S.Silencing of CKIP-1 promotes tumor proliferation and cell adhesion-mediated drugresistance via regulating AKT activity in non-Hodgkin's lymphoma. Oncol Rep.2017 Jan;37(1):622-630. doi: 10.3892/or.2016.5233. Epub 2016 Nov 8. PMID:27840970.  365: Wroblewski D, Mijatov B, Mohana-Kumaran N, Lai F, Gallagher SJ, Haass NK,Zhang XD, Hersey P. The BH3-mimetic ABT-737 sensitizes human melanoma cells toapoptosis induced by selective BRAF inhibitors but does not reverse acquiredresistance. Carcinogenesis. 2013 Feb;34(2):237-47. doi: 10.1093/carcin/bgs330.Epub 2012 Oct 20. PMID: 23087082.  366: Ge Z, Quek BL, Beemon KL, Hogg JR. Polypyrimidine tract binding protein 1protects mRNAs from recognition by the nonsense-mediated mRNA decay pathway.Elife. 2016 Jan 8;5:e11155. doi: 10.7554/eLife.11155. PMID: 26744779; PMCID:PMC4764554.  367: Peng Y, Gao Y, Yang C, Guo R, Shi X, Cao X. Low-Molecular-WeightPoly(ethylenimine) Nanogels Loaded with Ultrasmall Iron Oxide Nanoparticles for<i>T</i><sub>1</sub>-Weighted MR Imaging-Guided Gene Therapy of Sarcoma. ACSAppl Mater Interfaces. 2021 Jun 23;13(24):27806-27813. doi:10.1021/acsami.1c04081. Epub 2021 Jun 9. PMID: 34105346.  368: Lorenzo-Morales J, Martín-Navarro CM, López-Arencibia A, Santana-MoralesMA, Afonso-Lehmann RN, Maciver SK, Valladares B, Martínez-Carretero E.Therapeutic potential of a combination of two gene-specific small interferingRNAs against clinical strains of Acanthamoeba. Antimicrob Agents Chemother. 2010Dec;54(12):5151-5. doi: 10.1128/AAC.00329-10. Epub 2010 Sep 20. PMID: 20855732;PMCID: PMC2981231.  369: Hu W, Jin L, Jiang CC, Long GV, Scolyer RA, Wu Q, Zhang XD, Mei Y, Wu M.AEBP1 upregulation confers acquired resistance to BRAF (V600E) inhibition inmelanoma. Cell Death Dis. 2013 Nov 7;4(11):e914. doi: 10.1038/cddis.2013.441.PMID: 24201813; PMCID: PMC3847319.  370: Miller SJ, Rangwala F, Williams J, Ackerman P, Kong S, Jegga AG, Kaiser S,Aronow BJ, Frahm S, Kluwe L, Mautner V, Upadhyaya M, Muir D, Wallace M, Hagen J,Quelle DE, Watson MA, Perry A, Gutmann DH, Ratner N. Large-scale molecularcomparison of human schwann cells to malignant peripheral nerve sheath tumorcell lines and tissues. Cancer Res. 2006 Mar 1;66(5):2584-91. doi:10.1158/0008-5472.CAN-05-3330. PMID: 16510576.  371: Sayan M, Shukla A, MacPherson MB, Macura SL, Hillegass JM, Perkins TN,Thompson JK, Beuschel SL, Miller JM, Mossman BT. Extracellular signal-regulatedkinase 5 and cyclic AMP response element binding protein are novel pathwaysinhibited by vandetanib (ZD6474) and doxorubicin in mesotheliomas. Am J RespirCell Mol Biol. 2014 Nov;51(5):595-603. doi: 10.1165/rcmb.2013-0373TR. PMID:24940987; PMCID: PMC4224081.  372: Xiao Y, Sun L, Fu Y, Huang Y, Zhou R, Hu X, Zhou P, Quan J, Li N, Fan XG.High mobility group box 1 promotes sorafenib resistance in HepG2 cells and invivo. BMC Cancer. 2017 Dec 15;17(1):857. doi: 10.1186/s12885-017-3868-2. PMID:29246127; PMCID: PMC5731191.  373: Ravegnini G, Sammarini G, Moran S, Calice G, Indio V, Urbini M, Astolfi A,Zanotti F, Pantaleo MA, Hrelia P, Angelini S. Mechanisms of resistance to a PI3Kinhibitor in gastrointestinal stromal tumors: an <i>omic</i> approach toidentify novel druggable targets. Cancer Manag Res. 2019 Jul 5;11:6229-6244.doi: 10.2147/CMAR.S189661. PMID: 31308757; PMCID: PMC6615718.  374: Liu X, Gao Y, Shen J, Yang W, Choy E, Mankin H, Hornicek FJ, Duan Z.Cyclin-Dependent Kinase 11 (CDK11) Is Required for Ovarian Cancer Cell Growth InVitro and In Vivo, and Its Inhibition Causes Apoptosis and Sensitizes Cells toPaclitaxel. Mol Cancer Ther. 2016 Jul;15(7):1691-701. doi:10.1158/1535-7163.MCT-16-0032. Epub 2016 May 20. PMID: 27207777; PMCID:PMC4936930.  375: Adang LA, Tomescu C, Law WK, Kedes DH. Intracellular Kaposi's sarcoma-associated herpesvirus load determines early loss of immune synapse components.J Virol. 2007 May;81(10):5079-90. doi: 10.1128/JVI.02738-06. Epub 2007 Feb 28.PMID: 17329329; PMCID: PMC1900224.  376: Zhao Z, Lin X, Tong Y, Li W. Silencing lncRNA ZFAS1 or elevatedmicroRNA-135a represses proliferation, migration, invasion and resistance toapoptosis of osteosarcoma cells. Cancer Cell Int. 2019 Dec 3;19:326. doi:10.1186/s12935-019-1049-x. PMID: 31827400; PMCID: PMC6892223.  377: Goffinet C, Schmidt S, Kern C, Oberbremer L, Keppler OT. EndogenousCD317/Tetherin limits replication of HIV-1 and murine leukemia virus in rodentcells and is resistant to antagonists from primate viruses. J Virol. 2010Nov;84(21):11374-84. doi: 10.1128/JVI.01067-10. Epub 2010 Aug 11. PMID:20702620; PMCID: PMC2953199.  378: Middleton RB, Bulleid NJ. Reconstitution of the folding pathway of collagenin a cell-free system: formation of correctly aligned and hydroxylated triplehelices. Biochem J. 1993 Dec 1;296 ( Pt 2)(Pt 2):511-7. doi: 10.1042/bj2960511.PMID: 8257444; PMCID: PMC1137724.  379: de Carvalho Bittencourt M, Saas P, Fresnay S, Yerly-Motta V, Ferrand C,Perruche S, Duperrier A, Hervé P, Tiberghien P, Chalmers DE. Exposure toexogenous DNA can modify the sensitivity of the Fas apoptotic pathway. J GeneMed. 2002 Jan-Feb;4(1):14-24. doi: 10.1002/jgm.226. PMID: 11828383.  380: Bijwaard K, Hearing VJ, Sontheimer R, Lieu TS, Vieira WD, Gersten DM.Studies on the expression and immunogenicity of the B50 melanoma antigen and itsrelationship with calreticulin. Melanoma Res. 1995 Oct;5(5):327-36. doi:10.1097/00008390-199510000-00005. PMID: 8541723.  381: Tang S, Tang X, Jin Z, Liu C, Huang Q, Wang L, Diaty DM, Ye Z. Circular RNAcircPDSS1 promotes osteosarcoma progression by sponging miR-502-3p andmiR-4436a. Anticancer Drugs. 2021 Nov 5. doi: 10.1097/CAD.0000000000001261. Epubahead of print. PMID: 34744154.  382: Duan L, Perez RE, Hansen M, Gitelis S, Maki CG. Increasing cisplatinsensitivity by schedule-dependent inhibition of AKT and Chk1. Cancer Biol Ther.2014;15(12):1600-12. doi: 10.4161/15384047.2014.961876. PMID: 25482935; PMCID:PMC4623033.  383: Milhem MM, Knutson T, Yang S, Zhu D, Wang X, Leslie KK, Meng X. Correlationof MTDH/AEG-1 and HOTAIR Expression with Metastasis and Response to Treatment inSarcoma Patients. J Cancer Sci Ther. 2011 Dec 29;S5(4):004. PMID: 23543869;PMCID: PMC3612017.  384: Guoan X, Hanning W, Kaiyun C, Hao L. Adenovirus-mediated siRNA targetingMcl-1 gene increases radiosensitivity of pancreatic carcinoma cells in vitro andin vivo. Surgery. 2010 Apr;147(4):553-61. doi: 10.1016/j.surg.2009.10.033. Epub2009 Dec 11. PMID: 20004446.  385: Yuan Y, Song JX, Zhang MN, Yuan BS. A multiple drug loaded, functionalizedpH-sensitive nanocarrier as therapeutic and epigenetic modulator forosteosarcoma. Sci Rep. 2020 Sep 23;10(1):15497. doi: 10.1038/s41598-020-72552-z.PMID: 32968136; PMCID: PMC7511925.  386: Haghiralsadat F, Amoabediny G, Helder MN, Naderinezhad S, Sheikhha MH,Forouzanfar T, Zandieh-Doulabi B. A comprehensive mathematical model of drugrelease kinetics from nano-liposomes, derived from optimization studies ofcationic PEGylated liposomal doxorubicin formulations for drug-gene delivery.Artif Cells Nanomed Biotechnol. 2018 Feb;46(1):169-177. doi:10.1080/21691401.2017.1304403. Epub 2017 Apr 4. PMID: 28376641.  387: Schwermer M, Lee S, Köster J, van Maerken T, Stephan H, Eggert A, Morik K,Schulte JH, Schramm A. Sensitivity to cdk1-inhibition is modulated by p53 statusin preclinical models of embryonal tumors. Oncotarget. 2015 Jun20;6(17):15425-35. doi: 10.18632/oncotarget.3908. PMID: 26029996; PMCID:PMC4558161.  388: Sudo H, Maru Y. LAPSER1 is a putative cytokinetic tumor suppressor thatshows the same centrosome and midbody subcellular localization pattern as p80katanin. FASEB J. 2007 Jul;21(9):2086-100. doi: 10.1096/fj.06-7254com. Epub 2007Mar 9. PMID: 17351128.  389: Li J, Li Q, Xie C, Zhou H, Wang Y, Zhang N, Shao H, Chan SC, Peng X, LinSC, Han J. Beta-actin is required for mitochondria clustering and ROS generationin TNF-induced, caspase-independent cell death. J Cell Sci. 2004 Sep 15;117(Pt20):4673-80. doi: 10.1242/jcs.01339. PMID: 15371523.  390: Ma W, Teng Y, Hua H, Hou J, Luo T, Jiang Y. Upregulation of heat shockprotein 27 confers resistance to actinomycin D-induced apoptosis in cancercells. FEBS J. 2013 Sep;280(18):4612-24. doi: 10.1111/febs.12432. Epub 2013 Jul31. PMID: 23848600.  391: Tsou YL, Lin YW, Chang HW, Lin HY, Shao HY, Yu SL, Liu CC, Chitra E, Sia C,Chow YH. Heat shock protein 90: role in enterovirus 71 entry and assembly andpotential target for therapy. PLoS One. 2013 Oct 2;8(10):e77133. doi:10.1371/journal.pone.0077133. PMID: 24098578; PMCID: PMC3788750.  392: Hu WY, Myers CP, Kilzer JM, Pfaff SL, Bushman FD. Inhibition of retroviralpathogenesis by RNA interference. Curr Biol. 2002 Aug 6;12(15):1301-11. doi:10.1016/s0960-9822(02)00975-2. PMID: 12176358.  393: Wang W, Jiang Q, Wu J, Tang W, Xu M. Upregulation of bone morphogeneticprotein 2 ( Bmp2) in dorsal root ganglion in a rat model of bone cancer pain.Mol Pain. 2019 Jan-Dec;15:1744806918824250. doi: 10.1177/1744806918824250. PMID:30799697; PMCID: PMC6329035.  394: Niecknig H, Tug S, Reyes BD, Kirsch M, Fandrey J, Berchner-Pfannschmidt U.Role of reactive oxygen species in the regulation of HIF-1 by prolyl hydroxylase2 under mild hypoxia. Free Radic Res. 2012 Jun;46(6):705-17. doi:10.3109/10715762.2012.669041. Epub 2012 Apr 3. PMID: 22360728.  395: Crameri M, Bauer M, Caduff N, Walker R, Steiner F, Franzoso FD, Gujer C,Boucke K, Kucera T, Zbinden A, Münz C, Fraefel C, Greber UF, Pavlovic J. MxB isan interferon-induced restriction factor of human herpesviruses. Nat Commun.2018 May 17;9(1):1980. doi: 10.1038/s41467-018-04379-2. PMID: 29773792; PMCID:PMC5958057.  396: Oh J, Chang KW, Hughes SH. Mutations in the U5 sequences adjacent to theprimer binding site do not affect tRNA cleavage by rous sarcoma virus RNase Hbut do cause aberrant integrations in vivo. J Virol. 2006 Jan;80(1):451-9. doi:10.1128/JVI.80.1.451-459.2006. PMID: 16352569; PMCID: PMC1317513.  397: Zhang J, Ding R, Wu T, Jia J, Cheng X. Autophagy-Related Genes and LongNoncoding RNAs Signatures as Predictive Biomarkers for Osteosarcoma Survival.Front Cell Dev Biol. 2021 Aug 26;9:705291. doi: 10.3389/fcell.2021.705291. PMID:34513835; PMCID: PMC8427445.  398: Rinaldi G, Pranzini E, Van Elsen J, Broekaert D, Funk CM, Planque M,Doglioni G, Altea-Manzano P, Rossi M, Geldhof V, Teoh ST, Ross C, Hunter KW,Lunt SY, Grünewald TGP, Fendt SM. In Vivo Evidence for Serine Biosynthesis-Defined Sensitivity of Lung Metastasis, but Not of Primary Breast Tumors, tomTORC1 Inhibition. Mol Cell. 2021 Jan 21;81(2):386-397.e7. doi:10.1016/j.molcel.2020.11.027. Epub 2020 Dec 18. PMID: 33340488.  399: Murley JS, Nantajit D, Baker KL, Kataoka Y, Li JJ, Grdina DJ. Maintenanceof manganese superoxide dismutase (SOD2)-mediated delayed radioprotectioninduced by repeated administration of the free thiol form of amifostine. RadiatRes. 2008 May;169(5):495-505. doi: 10.1667/RR1194.1. PMID: 18439041; PMCID:PMC2384167.  400: Yu L, Nian Z, Sang J. Osteosarcoma amplified 9 is highly expressed in mouseadipocytes and controls lipid storage. Mol Med Rep. 2011 Jul-Aug;4(4):687-92.doi: 10.3892/mmr.2011.480. Epub 2011 Apr 26. PMID: 21523322.  401: Kilic M, Kasperczyk H, Fulda S, Debatin KM. Role of hypoxia induciblefactor-1 alpha in modulation of apoptosis resistance. Oncogene. 2007 Mar29;26(14):2027-38. doi: 10.1038/sj.onc.1210008. Epub 2006 Oct 16. PMID:17043658.  402: Duesberg PH, Bister K, Vogt PK. The RNA of avian acute leukemia virus MC29.Proc Natl Acad Sci U S A. 1977 Oct;74(10):4320-4. doi: 10.1073/pnas.74.10.4320.PMID: 200913; PMCID: PMC431932.  403: Lisse TS, Liu T, Irmler M, Beckers J, Chen H, Adams JS, Hewison M. Genetargeting by the vitamin D response element binding protein reveals a role forvitamin D in osteoblast mTOR signaling. FASEB J. 2011 Mar;25(3):937-47. doi:10.1096/fj.10-172577. Epub 2010 Dec 1. PMID: 21123297; PMCID: PMC3042839.  404: Alpsoy A, Utturkar SM, Carter BC, Dhiman A, Torregrosa-Allen SE, Currie MP,Elzey BD, Dykhuizen EC. BRD9 Is a Critical Regulator of Androgen ReceptorSignaling and Prostate Cancer Progression. Cancer Res. 2021 Feb15;81(4):820-833. doi: 10.1158/0008-5472.CAN-20-1417. Epub 2020 Dec 21. PMID:33355184; PMCID: PMC8026650.  405: Grant CV, Cai S, Risinger AL, Liang H, O'Keefe BR, Doench JG, Cichewicz RH,Mooberry SL. CRISPR-Cas9 Genome-Wide Knockout Screen Identifies Mechanism ofSelective Activity of Dehydrofalcarinol in Mesenchymal Stem-like Triple-NegativeBreast Cancer Cells. J Nat Prod. 2020 Oct 23;83(10):3080-3092. doi:10.1021/acs.jnatprod.0c00642. Epub 2020 Oct 6. PMID: 33021790; PMCID:PMC7722265.  406: Li F, Xu J, Zhu Y, Sun L, Zhou R. Analysis of Cells Proliferation andMicroRNAs Expression Profile in Human Chondrosarcoma SW1353 Cells Exposed toIodine-125 Seeds Irradiation. Dose Response. 2020 Apr 23;18(2):1559325820920525.doi: 10.1177/1559325820920525. PMID: 32362797; PMCID: PMC7180315.  407: Rengaswamy V, Kontny U, Rössler J. New approaches for pediatricrhabdomyosarcoma drug discovery: targeting combinatorial signaling. Expert OpinDrug Discov. 2011 Oct;6(10):1103-25. doi: 10.1517/17460441.2011.611498. Epub2011 Sep 6. PMID: 22646865.  408: Gougelet A, Perez J, Pissaloux D, Besse A, Duc A, Decouvelaere AV,Ranchere-Vince D, Blay JY, Alberti L. miRNA Profiling: How to Bypass the CurrentDifficulties in the Diagnosis and Treatment of Sarcomas. Sarcoma.2011;2011:460650. doi: 10.1155/2011/460650. Epub 2011 Feb 22. PMID: 21437224;PMCID: PMC3061295.  409: Iaea DB, Spahr ZR, Singh RK, Chan RB, Zhou B, Bareja R, Elemento O, DiPaolo G, Zhang X, Maxfield FR. Stable reduction of STARD4 alters cholesterolregulation and lipid homeostasis. Biochim Biophys Acta Mol Cell Biol Lipids.2020 Apr;1865(4):158609. doi: 10.1016/j.bbalip.2020.158609. Epub 2020 Jan 7.PMID: 31917335; PMCID: PMC6996790.  410: Foreman HC, Kirillov V, Paniccia G, Catalano D, Andrunik T, Gupta S, KrugLT, Zhang Y. RNA-guided gene editing of the murine gammaherpesvirus 68 genomereduces infectious virus production. PLoS One. 2021 Jun 4;16(6):e0252313. doi:10.1371/journal.pone.0252313. PMID: 34086743; PMCID: PMC8177658.  411: Liang Q, Dexheimer TS, Zhang P, Rosenthal AS, Villamil MA, You C, Zhang Q,Chen J, Ott CA, Sun H, Luci DK, Yuan B, Simeonov A, Jadhav A, Xiao H, Wang Y,Maloney DJ, Zhuang Z. A selective USP1-UAF1 inhibitor links deubiquitination toDNA damage responses. Nat Chem Biol. 2014 Apr;10(4):298-304. doi:10.1038/nchembio.1455. Epub 2014 Feb 16. PMID: 24531842; PMCID: PMC4144829.  412: Oh J, Chang KW, Wierzchoslawski R, Alvord WG, Hughes SH. Rous sarcoma virus(RSV) integration in vivo: a CA dinucleotide is not required in U3, and RSVlinear DNA does not autointegrate. J Virol. 2008 Jan;82(1):503-12. doi:10.1128/JVI.01441-07. Epub 2007 Oct 24. PMID: 17959663; PMCID: PMC2224354.  413: Barakat S, Demeule M, Pilorget A, Régina A, Gingras D, Baggetto LG,Béliveau R. Modulation of p-glycoprotein function by caveolin-1 phosphorylation.J Neurochem. 2007 Apr;101(1):1-8. doi: 10.1111/j.1471-4159.2006.04410.x. Epub2007 Feb 26. PMID: 17326770.  414: Miller JT, Ge Z, Morris S, Das K, Leis J. Multiple biological rolesassociated with the Rous sarcoma virus 5' untranslated RNA U5-IR stem and loop.J Virol. 1997 Oct;71(10):7648-56. doi: 10.1128/JVI.71.10.7648-7656.1997. PMID:9311847; PMCID: PMC192114.  415: Zhai Y, Liu M, Zheng Y. MicroRNA-451 dictates the anoikis resistance ofosteosarcoma by targeting Rab14. Int J Clin Exp Pathol. 2017 Nov1;10(11):10989-10997. PMID: 31966443; PMCID: PMC6965860.  416: Yang Y, Yang Y, Xie X, Xu X, Xia X, Wang H, Li L, Dong W, Ma P, Liu Y. Dualstimulus of hyperthermia and intracellular redox environment triggered releaseof siRNA for tumor-specific therapy. Int J Pharm. 2016 Jun 15;506(1-2):158-73.doi: 10.1016/j.ijpharm.2016.04.035. Epub 2016 Apr 19. PMID: 27106526.  417: Chen Z, Xu W, Zhang D, Chu J, Shen S, Ma Y, Wang Q, Liu G, Yao T, Huang Y,Ye H, Wang J, Ma J, Fan S. circCAMSAP1 promotes osteosarcoma progression andmetastasis by sponging miR-145-5p and regulating FLI1 expression. Mol TherNucleic Acids. 2020 Dec 23;23:1120-1135. doi: 10.1016/j.omtn.2020.12.013. PMID:33664993; PMCID: PMC7901030.  418: Gandy SZ, Linnstaedt SD, Muralidhar S, Cashman KA, Rosenthal LJ, Casey JL.RNA editing of the human herpesvirus 8 kaposin transcript eliminates itstransforming activity and is induced during lytic replication. J Virol. 2007Dec;81(24):13544-51. doi: 10.1128/JVI.01521-07. Epub 2007 Oct 3. PMID: 17913828;PMCID: PMC2168827.  419: Lee SK, Nagashima K, Hu WS. Cooperative effect of gag proteins p12 andcapsid during early events of murine leukemia virus replication. J Virol. 2005Apr;79(7):4159-69. doi: 10.1128/JVI.79.7.4159-4169.2005. PMID: 15767417; PMCID:PMC1061564.  420: Zhang C, He J, Qi L, Wan L, Wang W, Tu C, Li Z. Diagnostic and PrognosticSignificance of Dysregulated Expression of Circular RNAs in Osteosarcoma. ExpertRev Mol Diagn. 2021 Feb;21(2):235-244. doi: 10.1080/14737159.2021.1874922. Epub2021 Jan 22. PMID: 33428501.  421: Olfa G, Christophe C, Philippe L, Romain S, Khaled H, Pierre H, Odile B,Jean-Christophe D. RUNX2 regulates the effects of TNFalpha on proliferation andapoptosis in SaOs-2 cells. Bone. 2010 Apr;46(4):901-10. doi:10.1016/j.bone.2009.12.027. Epub 2010 Jan 4. PMID: 20053387.  422: Bednarski BK, Baldwin AS Jr, Kim HJ. Addressing reported pro-apoptoticfunctions of NF-kappaB: targeted inhibition of canonical NF-kappaB enhances theapoptotic effects of doxorubicin. PLoS One. 2009 Sep 10;4(9):e6992. doi:10.1371/journal.pone.0006992. PMID: 19746155; PMCID: PMC2734988.  423: Yin P, Xu Q, Duan C. Paradoxical actions of endogenous and exogenousinsulin-like growth factor-binding protein-5 revealed by RNA interferenceanalysis. J Biol Chem. 2004 Jul 30;279(31):32660-6. doi: 10.1074/jbc.M401378200.Epub 2004 May 19. PMID: 15155755.  424: Siew EL, Chan KM, Williams GT, Ross D, Inayat-Hussain SH. Protection ofhydroquinone-induced apoptosis by downregulation of Fau is mediated by NQO1.Free Radic Biol Med. 2012 Oct 15;53(8):1616-24. doi:10.1016/j.freeradbiomed.2012.05.046. Epub 2012 Jun 9. PMID: 22687461.  425: Hussain KM, Leong KL, Ng MM, Chu JJ. The essential role of clathrin-mediated endocytosis in the infectious entry of human enterovirus 71. J BiolChem. 2011 Jan 7;286(1):309-21. doi: 10.1074/jbc.M110.168468. Epub 2010 Oct 18.PMID: 20956521; PMCID: PMC3012988.  426: Shibui Y, Kohashi K, Tamaki A, Kinoshita I, Yamada Y, Yamamoto H, TaguchiT, Oda Y. The forkhead box M1 (FOXM1) expression and antitumor effect of FOXM1inhibition in malignant rhabdoid tumor. J Cancer Res Clin Oncol. 2021May;147(5):1499-1518. doi: 10.1007/s00432-020-03438-w. Epub 2020 Nov 21. PMID:33221995.  427: Samore WR, Gondi CS. Brief overview of selected approaches in targetingpancreatic adenocarcinoma. Expert Opin Investig Drugs. 2014 Jun;23(6):793-807.doi: 10.1517/13543784.2014.902933. Epub 2014 Mar 27. PMID: 24673265.  428: Zheng BH, He ZX, Zhang J, Ma JJ, Zhang HW, Zhu W, Shao ZM, Ni XJ. TheBiological Function of TUSC7/miR-1224-3p Axis in Triple-Negative Breast Cancer.Cancer Manag Res. 2021 Jul 17;13:5763-5774. doi: 10.2147/CMAR.S305865. PMID:34305410; PMCID: PMC8296971.  429: Wei J, Fang DL, Huang CK, Hua SL, Lu XS. Screening a novel signature andpredicting the immune landscape of metastatic osteosarcoma in children viaimmune-related lncRNAs. Transl Pediatr. 2021 Jul;10(7):1851-1866. doi:10.21037/tp-21-226. PMID: 34430433; PMCID: PMC8349967.  430: Iko Y, Kodama TS, Kasai N, Oyama T, Morita EH, Muto T, Okumura M, Fujii R,Takumi T, Tate S, Morikawa K. Domain architectures and characterization of anRNA-binding protein, TLS. J Biol Chem. 2004 Oct 22;279(43):44834-40. doi:10.1074/jbc.M408552200. Epub 2004 Aug 6. PMID: 15299008.  431: Tu C, Ren X, He J, Zhang C, Chen R, Wang W, Li Z. The Value of LncRNA BCAR4as a Prognostic Biomarker on Clinical Outcomes in Human Cancers. J Cancer. 2019Oct 15;10(24):5992-6002. doi: 10.7150/jca.35113. PMID: 31762809; PMCID:PMC6856575.  432: Capparelli C, Rosenbaum S, Berger AC, Aplin AE. Fibroblast-derivedneuregulin 1 promotes compensatory ErbB3 receptor signaling in mutant BRAFmelanoma. J Biol Chem. 2015 Oct 2;290(40):24267-77. doi:10.1074/jbc.M115.657270. Epub 2015 Aug 12. PMID: 26269601; PMCID: PMC4591813.  433: Xu JF, Wang YP, Zhang SJ, Chen Y, Gu HF, Dou XF, Xia B, Bi Q, Fan SW.Exosomes containing differential expression of microRNA and mRNA in osteosarcomathat can predict response to chemotherapy. Oncotarget. 2017 Jun6;8(44):75968-75978. doi: 10.18632/oncotarget.18373. PMID: 29100284; PMCID:PMC5652678.  434: Liao C, Beveridge R, Hudson JJR, Parker JD, Chiang SC, Ray S, Ashour ME,Sudbery I, Dickman MJ, El-Khamisy SF. UCHL3 Regulates Topoisomerase-InducedChromosomal Break Repair by Controlling TDP1 Proteostasis. Cell Rep. 2018 Jun12;23(11):3352-3365. doi: 10.1016/j.celrep.2018.05.033. PMID: 29898404; PMCID:PMC6019701.  435: Yang Q, McDermott PJ, Duzic E, Pleij CW, Sherlock JD, Lanier SM. The3'-untranslated region of the alpha2C-adrenergic receptor mRNA impedestranslation of the receptor message. J Biol Chem. 1997 Jun 13;272(24):15466-73.doi: 10.1074/jbc.272.24.15466. PMID: 9182579.  436: Takahashi R, Nagayama S, Furu M, Kajita Y, Jin Y, Kato T, Imoto S, Sakai Y,Toguchida J. AFAP1L1, a novel associating partner with vinculin, modulatescellular morphology and motility, and promotes the progression of colorectalcancers. Cancer Med. 2014 Aug;3(4):759-74. doi: 10.1002/cam4.237. Epub 2014 Apr10. PMID: 24723436; PMCID: PMC4303145.  437: Wang L, Liu Y, Li H, Zhang C, Wang H, Dai S, Cheng W, Sun Y, Zheng X.<i>miR-4478</i> sensitizes ovarian cancer cells to irradiation by inhibiting<i>Fus</i> and attenuating autophagy. Mol Ther Nucleic Acids. 2020 Dec3;23:1110-1119. doi: 10.1016/j.omtn.2020.11.024. PMID: 33664992; PMCID:PMC7901029.  438: Liu S, Yang J, Wang L, Jiang M, Qiu Q, Ma Z, Liu L, Li C, Ren C, Zhou J, LiW. Tibia tumor-induced cancer pain involves spinal p38 mitogen-activated proteinkinase activation via TLR4-dependent mechanisms. Brain Res. 2010 Jul30;1346:213-23. doi: 10.1016/j.brainres.2010.05.014. Epub 2010 May 15. PMID:20478276.  439: Yi W, Tu MJ, Liu Z, Zhang C, Batra N, Yu AX, Yu AM. BioengineeredmiR-328-3p modulates GLUT1-mediated glucose uptake and metabolism to exertsynergistic antiproliferative effects with chemotherapeutics. Acta Pharm Sin B.2020 Jan;10(1):159-170. doi: 10.1016/j.apsb.2019.11.001. Epub 2019 Nov 7. PMID:31993313; PMCID: PMC6976971.  440: Shu X, Liu W, Liu H, Qi H, Wu C, Ran YL. Analysis of microRNA expression inCD133 positive cancer stem‑like cells of human osteosarcoma cell line MG-63.PeerJ. 2021 Sep 3;9:e12115. doi: 10.7717/peerj.12115. PMID: 34557357; PMCID:PMC8420872.  441: Mongre RK, Sodhi SS, Ghosh M, Kim JH, Kim N, Sharma N, Jeong DK. A NewParadigm to Mitigate Osteosarcoma by Regulation of MicroRNAs and Suppression ofthe NF-κB Signaling Cascade. Dev Reprod. 2014 Dec;18(4):197-212. doi:10.12717/devrep.2014.18.4.197. PMID: 25949190; PMCID: PMC4415640.  442: Huang WT, Liu AG, Cai KT, He RQ, Li Z, Wei QJ, Chen MY, Huang JY, Yan WY,Zhou H, Chen G, Ma J. Exploration and validation of downregulatedmicroRNA-199a-3p, downstream messenger RNA targets and transcriptionalregulation in osteosarcoma. Am J Transl Res. 2019 Dec 15;11(12):7538-7554. PMID:31934299; PMCID: PMC6943471.  443: Xiao Y, Deng T, Su C, Shang Z. MicroRNA 217 inhibits cell proliferation andenhances chemosensitivity to doxorubicin in acute myeloid leukemia by targetingKRAS. Oncol Lett. 2017 Jun;13(6):4986-4994. doi: 10.3892/ol.2017.6076. Epub 2017Apr 24. PMID: 28599501; PMCID: PMC5453027.  444: Jaquish DV, Yu PT, Shields DJ, French RP, Maruyama KP, Niessen S, Hoover H,A Cheresh D, Cravatt B, Lowy AM. IGF1-R signals through the RON receptor tomediate pancreatic cancer cell migration. Carcinogenesis. 2011 Aug;32(8):1151-6.doi: 10.1093/carcin/bgr086. Epub 2011 May 11. PMID: 21565828; PMCID: PMC3149203.  445: Horiuchi M, Kuga T, Saito Y, Nagano M, Adachi J, Tomonaga T, Yamaguchi N,Nakayama Y. The tyrosine kinase v-Src causes mitotic slippage by phosphorylatingan inhibitory tyrosine residue of Cdk1. J Biol Chem. 2018 Oct5;293(40):15524-15537. doi: 10.1074/jbc.RA118.002784. Epub 2018 Aug 22. PMID:30135207; PMCID: PMC6177586.  446: Wang PT, Garcin PO, Fu M, Masoudi M, St-Pierre P, Panté N, Nabi IR.Distinct mechanisms controlling rough and smooth endoplasmic reticulum contactswith mitochondria. J Cell Sci. 2015 Aug 1;128(15):2759-65. doi:10.1242/jcs.171132. Epub 2015 Jun 11. PMID: 26065430.  447: Hirayoshi K, Kudo H, Takechi H, Nakai A, Iwamatsu A, Yamada KM, Nagata K.HSP47: a tissue-specific, transformation-sensitive, collagen-binding heat shockprotein of chicken embryo fibroblasts. Mol Cell Biol. 1991 Aug;11(8):4036-44.doi: 10.1128/mcb.11.8.4036-4044.1991. PMID: 2072906; PMCID: PMC361208.  448: Zhang L, Zhang W. Knockdown of NUDT21 inhibits proliferation and promotesapoptosis of human K562 leukemia cells through ERK pathway. Cancer Manag Res.2018 Oct 8;10:4311-4323. doi: 10.2147/CMAR.S173496. PMID: 30349365; PMCID:PMC6183658.  449: Berdyshev EV, Gorshkova IA, Usatyuk P, Zhao Y, Saatian B, Hubbard W,Natarajan V. De novo biosynthesis of dihydrosphingosine-1-phosphate bysphingosine kinase 1 in mammalian cells. Cell Signal. 2006 Oct;18(10):1779-92.doi: 10.1016/j.cellsig.2006.01.018. Epub 2006 Mar 10. PMID: 16529909.  450: Lei S, Xiang L. Up-Regulation of circRNA hsa_circ_0003074 Expression is aReliable Diagnostic and Prognostic Biomarker in Patients with Osteosarcoma.Cancer Manag Res. 2020 Sep 29;12:9315-9325. doi: 10.2147/CMAR.S262093. PMID:33061621; PMCID: PMC7532912.  451: Kubota D, Kosaka N, Fujiwara T, Yoshida A, Arai Y, Qiao Z, Takeshita F,Ochiya T, Kawai A, Kondo T. miR-125b and miR-100 Are Predictive Biomarkers ofResponse to Induction Chemotherapy in Osteosarcoma. Sarcoma. 2016;2016:1390571.doi: 10.1155/2016/1390571. Epub 2016 Nov 21. PMID: 27990096; PMCID: PMC5136640.  452: Nwaneri AC, McBeth L, Hinds TD Jr. Sweet-P inhibition of glucocorticoidreceptor β as a potential cancer therapy. Cancer Cell Microenviron.2016;3(3):e1362. Epub 2016 Jul 5. PMID: 27468424; PMCID: PMC4959805.  453: Martínez-Bosch N, Fernández-Barrena MG, Moreno M, Ortiz-Zapater E, Munné-Collado J, Iglesias M, André S, Gabius HJ, Hwang RF, Poirier F, Navas C, GuerraC, Fernández-Zapico ME, Navarro P. Galectin-1 drives pancreatic carcinogenesisthrough stroma remodeling and Hedgehog signaling activation. Cancer Res. 2014Jul 1;74(13):3512-24. doi: 10.1158/0008-5472.CAN-13-3013. Epub 2014 May 8. PMID:24812270; PMCID: PMC4332591.  454: Kook SH, Son YO, Jang YS, Lee KY, Lee SA, Kim BS, Lee HJ, Lee JC.Inhibition of c-Jun N-terminal kinase sensitizes tumor cells to flavonoid-induced apoptosis through down-regulation of JunD. Toxicol Appl Pharmacol. 2008Mar 15;227(3):468-76. doi: 10.1016/j.taap.2007.11.004. Epub 2007 Nov 17. PMID:18078968.  455: Hiraki M, Nishimura J, Takahashi H, Wu X, Takahashi Y, Miyo M, Nishida N,Uemura M, Hata T, Takemasa I, Mizushima T, Soh JW, Doki Y, Mori M, Yamamoto H.Concurrent Targeting of KRAS and AKT by MiR-4689 Is a Novel Treatment AgainstMutant KRAS Colorectal Cancer. Mol Ther Nucleic Acids. 2015 Mar 10;4(3):e231.doi: 10.1038/mtna.2015.5. PMID: 25756961; PMCID: PMC4354340.  456: Keßler J, Rot S, Bache M, Kappler M, Würl P, Vordermark D, Taubert H,Greither T. miR-199a-5p regulates <i>HIF-1α</i> and <i>OSGIN2</i> and itsexpression is correlated to soft-tissue sarcoma patients' outcome. Oncol Lett.2016 Dec;12(6):5281-5288. doi: 10.3892/ol.2016.5320. Epub 2016 Oct 26. PMID:28101243; PMCID: PMC5228330.  457: Guzmán-Pérez V, Bumke-Vogt C, Schreiner M, Mewis I, Borchert A, PfeifferAF. Benzylglucosinolate Derived Isothiocyanate from Tropaeolum majus ReducesGluconeogenic Gene and Protein Expression in Human Cells. PLoS One. 2016 Sep13;11(9):e0162397. doi: 10.1371/journal.pone.0162397. PMID: 27622707; PMCID:PMC5021297.  458: Hashimoto K, Inada M, Yamamoto Y, Ochiya T. Preliminary evaluation ofmiR-1307-3p in human serum for detection of 13 types of solid cancer usingmicroRNA chip. Heliyon. 2021 Sep 2;7(9):e07919. doi:10.1016/j.heliyon.2021.e07919. PMID: 34541347; PMCID: PMC8441074.  459: Chen K, Tu Y, Zhang Y, Blair HC, Zhang L, Wu C. PINCH-1 regulates the ERK-Bim pathway and contributes to apoptosis resistance in cancer cells. J BiolChem. 2008 Feb 1;283(5):2508-17. doi: 10.1074/jbc.M707307200. Epub 2007 Dec 6.PMID: 18063582.  460: Pickard MR, Green AR, Ellis IO, Caldas C, Hedge VL, Mourtada-Maarabouni M,Williams GT. Dysregulated expression of Fau and MELK is associated with poorprognosis in breast cancer. Breast Cancer Res. 2009;11(4):R60. doi:10.1186/bcr2350. Epub 2009 Aug 11. PMID: 19671159; PMCID: PMC2750122.  461: Deng X, Ewton DZ, Li S, Naqvi A, Mercer SE, Landas S, Friedman E. Thekinase Mirk/Dyrk1B mediates cell survival in pancreatic ductal adenocarcinoma.Cancer Res. 2006 Apr 15;66(8):4149-58. doi: 10.1158/0008-5472.CAN-05-3089. PMID:16618736.  462: Katz RA, Cullen BR, Malavarca R, Skalka AM. Role of the avian retrovirusmRNA leader in expression: evidence for novel translational control. Mol CellBiol. 1986 Feb;6(2):372-9. doi: 10.1128/mcb.6.2.372-379.1986. PMID: 3023842;PMCID: PMC367526.  463: Santra M, Danielson KG, Iozzo RV. Structural and functionalcharacterization of the human decorin gene promoter. A homopurine-homopyrimidineS1 nuclease-sensitive region is involved in transcriptional control. J BiolChem. 1994 Jan 7;269(1):579-87. PMID: 8276854.  464: Grez M, Akgün E, Hilberg F, Ostertag W. Embryonic stem cell virus, arecombinant murine retrovirus with expression in embryonic stem cells. Proc NatlAcad Sci U S A. 1990 Dec;87(23):9202-6. doi: 10.1073/pnas.87.23.9202. PMID:2251265; PMCID: PMC55132.  465: Wang T, Wu J, Liu X, Li S. Serum miR-34a is a potential diagnostic andprognostic marker for osteosarcoma. Int J Clin Exp Pathol. 2017 Sep1;10(9):9683-9689. PMID: 31966849; PMCID: PMC6965930.  466: Ahronian LG, Lewis BC. Using the RCAS-TVA system to model human cancer inmice. Cold Spring Harb Protoc. 2014 Nov 3;2014(11):1128-35. doi:10.1101/pdb.top069831. PMID: 25368315.  467: Ajmone-Cat MA, Onori A, Toselli C, Stronati E, Morlando M, Bozzoni I, MonniE, Kokaia Z, Lupo G, Minghetti L, Biagioni S, Cacci E. Increased FUS levels inastrocytes leads to astrocyte and microglia activation and neuronal death. SciRep. 2019 Mar 14;9(1):4572. doi: 10.1038/s41598-019-41040-4. PMID: 30872738;PMCID: PMC6418113.  468: Cheng D, Qiu X, Zhuang M, Zhu C, Zou H, Zhang A. Development and validationof nomogram based on miR-203 and clinicopathological characteristics predictingsurvival after neoadjuvant chemotherapy and surgery for patients with non-metastatic osteosarcoma. Oncotarget. 2017 Jun 17;8(57):96935-96944. doi:10.18632/oncotarget.18534. PMID: 29228583; PMCID: PMC5722535.  469: Mao L, Wu J, Shen L, Yang J, Chen J, Xu H. Enterovirus 71 transmission byexosomes establishes a productive infection in human neuroblastoma cells. VirusGenes. 2016 Apr;52(2):189-94. doi: 10.1007/s11262-016-1292-3. Epub 2016 Feb 2.PMID: 26837894.  470: Altwasser R, Paz A, Korol A, Manov I, Avivi A, Shams I. The transcriptomelandscape of the carcinogenic treatment response in the blind mole rat: insightsinto cancer resistance mechanisms. BMC Genomics. 2019 Jan 8;20(1):17. doi:10.1186/s12864-018-5417-z. PMID: 30621584; PMCID: PMC6323709.  471: Subhash VV, Huang L, Kamili A, Wong M, Chen D, Venn NC, Atkinson C, MayohC, Venkat P, Tyrrell V, Marshall GM, Cowley MJ, Ekert PG, Norris MD, Haber M,Henderson MJ, Sutton R, Fletcher JI, Trahair TN. Whole-genome sequencingfacilitates patient-specific quantitative PCR-based minimal residual diseasemonitoring in acute lymphoblastic leukaemia, neuroblastoma and Ewing sarcoma. BrJ Cancer. 2021 Sep 1. doi: 10.1038/s41416-021-01538-z. Epub ahead of print.PMID: 34471258.  472: Liu W, Wang D, Liu L, Wang L, Yan M. miR-140 inhibits osteosarcomaprogression by impairing USP22-mediated LSD1 stabilization and promoting p21expression. Mol Ther Nucleic Acids. 2021 Feb 3;24:436-448. doi:10.1016/j.omtn.2021.01.029. PMID: 33868787; PMCID: PMC8040122.  473: Guan M, Fousek K, Chow WA. Nelfinavir inhibits regulated intramembraneproteolysis of sterol regulatory element binding protein-1 and activatingtranscription factor 6 in castration-resistant prostate cancer. FEBS J. 2012Jul;279(13):2399-411. doi: 10.1111/j.1742-4658.2012.08619.x. Epub 2012 May 21.PMID: 22540830.  474: Fenstermaker RA, Milsted A, Virgin JB, Miller WL, Nilson JH. Thetranscriptional response of the human chorionic gonadotropin beta-subunit geneto cAMP is cycloheximide sensitive and is mediated by cis-acting sequencesdifferent from that found in the alpha-subunit gene. Mol Endocrinol. 1989Jul;3(7):1070-6. doi: 10.1210/mend-3-7-1070. PMID: 2477692.  475: Lei J, He MY, Li J, Li H, Wang W, Gopinath SCB, Xu LZ. miRNA identificationby nuclease digestion in ELISA for diagnosis of osteosarcoma. Biotechnol ApplBiochem. 2021 Jun 3. doi: 10.1002/bab.2209. Epub ahead of print. PMID: 34081808.  476: Lin Z, Pang K, Li H, Zhang X, Wan J, Zheng T, Liu T, Peng W.Characterization of Immune-Related Long Non-coding RNAs to Construct a NovelSignature and Predict the Prognosis and Immune Landscape of Soft Tissue Sarcoma.Front Cell Dev Biol. 2021 Sep 24;9:709241. doi: 10.3389/fcell.2021.709241. PMID:34631703; PMCID: PMC8497898.  477: Wang Y, Zhang L, Pang Y, Song L, Shang H, Li Z, Liu Q, Zhang Y, Wang X, LiQ, Zhang Q, Liu C, Li F. MicroRNA-29 family inhibits rhabdomyosarcoma formationand progression by regulating GEFT function. Am J Transl Res. 2020 Mar15;12(3):1136-1154. PMID: 32269740; PMCID: PMC7137044.  478: Vishnubalaji R, Elango R, Manikandan M, Siyal AA, Ali D, Al-Rikabi A, HamamD, Hamam R, Benabdelkamel H, Masood A, Alanazi IO, Alfadda AA, Alfayez M,Aldahmash A, Kassem M, Alajez NM. MicroRNA-3148 acts as molecular switchpromoting malignant transformation and adipocytic differentiation ofimmortalized human bone marrow stromal cells via direct targeting of theSMAD2/TGFβ pathway. Cell Death Discov. 2020 Sep 1;6(1):79. doi:10.1038/s41420-020-00312-z. PMID: 34465732.  479: Yoneda R, Ueda N, Kurokawa R. m<sup>6</sup>A Modified Short RNA FragmentsInhibit Cytoplasmic TLS/FUS Aggregation Induced by Hyperosmotic Stress. Int JMol Sci. 2021 Oct 12;22(20):11014. doi: 10.3390/ijms222011014. PMID: 34681673;PMCID: PMC8539258.  480: Kozakowska M, Dobrowolska-Glazar B, Okoń K, Józkowicz A, Dobrowolski Z,Dulak J. Preliminary Analysis of the Expression of Selected Proangiogenic andAntioxidant Genes and MicroRNAs in Patients with Non-Muscle-Invasive BladderCancer. J Clin Med. 2016 Feb 25;5(3):29. doi: 10.3390/jcm5030029. PMID:26927195; PMCID: PMC4810100.  481: Vishnubalaji R, Elango R, Manikandan M, Siyal AA, Ali D, Al-Rikabi A, HamamD, Hamam R, Benabdelkamel H, Masood A, Alanazi IO, Alfadda AA, Alfayez M,Aldahmash A, Kassem M, Alajez NM. MicroRNA-3148 acts as molecular switchpromoting malignant transformation and adipocytic differentiation ofimmortalized human bone marrow stromal cells via direct targeting of theSMAD2/TGFβ pathway. Cell Death Discov. 2020 Sep 1;6:79. doi:10.1038/s41420-020-00312-z. PMID: 32922961; PMCID: PMC7462980.  482: Yin Z, Ren W. MicroRNA-217 acts as a tumor suppressor and correlates withthe chemoresistance of cervical carcinoma to cisplatin. Onco Targets Ther. 2019Jan 23;12:759-771. doi: 10.2147/OTT.S176618. PMID: 30774364; PMCID: PMC6352857.  483: Fahs A, Ramadan F, Ghamloush F, Ayoub AJ, Ahmad FA, Kobeissy F, Mechref Y,Zhao J, Zhu R, Hussein N, Saab R, Ghayad SE. Effects of the OncoproteinPAX3-FOXO1 on Modulation of Exosomes Function and Protein Content: Implicationson Oxidative Stress Protection and Enhanced Plasticity. Front Oncol. 2020 Oct1;10:1784. doi: 10.3389/fonc.2020.01784. PMID: 33117671; PMCID: PMC7560303.  484: McKenzie JA, Mbofung RM, Malu S, Zhang M, Ashkin E, Devi S, Williams L,Tieu T, Peng W, Pradeep S, Xu C, Zorro Manrique S, Liu C, Huang L, Chen Y,Forget MA, Haymaker C, Bernatchez C, Satani N, Muller F, Roszik J, Kalra A,Heffernan T, Sood A, Hu J, Amaria R, Davis RE, Hwu P. The Effect ofTopoisomerase I Inhibitors on the Efficacy of T-Cell-Based Cancer Immunotherapy.J Natl Cancer Inst. 2018 Jul 1;110(7):777-786. doi: 10.1093/jnci/djx257. PMID:29267866; PMCID: PMC6037061.  485: Tian G, Hu K, Qiu S, Xie Y, Cao Y, Ni S, Zhang L. Exosomes derived fromPC-3 cells suppress osteoclast differentiation by downregulating miR-148a andblocking the PI3K/AKT/mTOR pathway. Exp Ther Med. 2021 Nov;22(5):1304. doi:10.3892/etm.2021.10739. Epub 2021 Sep 16. PMID: 34630659; PMCID: PMC8461599.  486: de Wit NJ, Weidle UH, Ruiter DJ, van Muijen GN. Expression profiling ofMMA-1a and splice variant MMA-1b: new cancer/testis antigens identified in humanmelanoma. Int J Cancer. 2002 Apr 1;98(4):547-53. doi: 10.1002/ijc.10241. PMID:11920614.  487: Piha-Paul SA, Dumbrava EE, Nair BC, Xiong W, Xu L, Mostorino R, Subbiah V,Tannir N, Fu S, Naing A, Janku F, Karp DD, Patel S, Daw NC, Hong D, Meric-Bernstam F, Zinner R. A Phase I Trial of the MET/<i>ALK/ROS1</i> InhibitorCrizotinib Combined with the VEGF Inhibitor Pazopanib in Patients with AdvancedSolid Malignancies. Onco Targets Ther. 2021 May 7;14:3037-3049. doi:10.2147/OTT.S291801. PMID: 33994796; PMCID: PMC8114359.  488: Baskin R, Sayeski PP. Angiotensin II mediates cell survival throughupregulation and activation of the serum and glucocorticoid inducible kinase 1.Cell Signal. 2012 Feb;24(2):435-442. doi: 10.1016/j.cellsig.2011.09.016. Epub2011 Sep 22. PMID: 21963429; PMCID: PMC3237851.  489: Potratz JC, Saunders DN, Wai DH, Ng TL, McKinney SE, Carboni JM, GottardisMM, Triche TJ, Jürgens H, Pollak MN, Aparicio SA, Sorensen PH. Syntheticlethality screens reveal RPS6 and MST1R as modifiers of insulin-like growthfactor-1 receptor inhibitor activity in childhood sarcomas. Cancer Res. 2010 Nov1;70(21):8770-81. doi: 10.1158/0008-5472.CAN-10-1093. Epub 2010 Oct 19. PMID:20959493.  490: Mori Y, Terauchi R, Shirai T, Tsuchida S, Mizoshiri N, Arai Y, Kishida T,Fujiwara H, Mazda O, Kubo T. Suppression of heat shock protein 70 by siRNAenhances the antitumor effects of cisplatin in cultured human osteosarcomacells. Cell Stress Chaperones. 2017 Sep;22(5):699-706. doi:10.1007/s12192-017-0793-x. Epub 2017 May 2. PMID: 28466152; PMCID: PMC5573688.  491: Zhang J, Li N, Lu S, Chen Y, Shan L, Zhao X, Xu Y. The role of Notch ligandJagged1 in osteosarcoma proliferation, metastasis, and recurrence. J Orthop SurgRes. 2021 Mar 29;16(1):226. doi: 10.1186/s13018-021-02372-y. PMID: 33781318;PMCID: PMC8006358.  492: Nativel B, Marimoutou M, Thon-Hon VG, Gunasekaran MK, Andries J, StanislasG, Planesse C, Da Silva CR, Césari M, Iwema T, Gasque P, Viranaicken W. SolubleHMGB1 is a novel adipokine stimulating IL-6 secretion through RAGE receptor inSW872 preadipocyte cell line: contribution to chronic inflammation in fattissue. PLoS One. 2013 Sep 20;8(9):e76039. doi: 10.1371/journal.pone.0076039.PMID: 24073286; PMCID: PMC3779194.  493: Hu Y, Jiao B, Chen L, Wang M, Han X. Long non-coding RNA GASL1 may inhibitthe proliferation of glioma cells by inactivating the TGF-β signaling pathway.Oncol Lett. 2019 Jun;17(6):5754-5760. doi: 10.3892/ol.2019.10273. Epub 2019 Apr18. PMID: 31186801; PMCID: PMC6507436.  494: May EW, Lin ST, Lin CC, Chang JF, Hung E, Lo YW, Lin LH, Hu RY, Feng CL,Lin DY, Wu SB, Lee WC, Lyu KW, Chou HC, Chan HL. Identification of up- and down-regulated proteins in doxorubicin-resistant uterine cancer cells:reticulocalbin-1 plays a key role in the development of doxorubicin-associatedresistance. Pharmacol Res. 2014 Dec;90:1-17. doi: 10.1016/j.phrs.2014.08.007.Epub 2014 Sep 19. PMID: 25242635.  495: Amara FM, Chen FY, Wright JA. Defining a novel cis element in the3'-untranslated region of mammalian ribonucleotide reductase component R2 mRNA:role in transforming growth factor-beta 1 induced mRNA stabilization. NucleicAcids Res. 1995 May 11;23(9):1461-7. doi: 10.1093/nar/23.9.1461. PMID: 7784197;PMCID: PMC306883.  496: Susa M, Choy E, Liu X, Schwab J, Hornicek FJ, Mankin H, Duan Z. CyclinG-associated kinase is necessary for osteosarcoma cell proliferation andreceptor trafficking. Mol Cancer Ther. 2010 Dec;9(12):3342-50. doi:10.1158/1535-7163.MCT-10-0637. Epub 2010 Sep 29. PMID: 20881269.  497: Kouvidi K, Berdiaki A, Nikitovic D, Katonis P, Afratis N, Hascall VC,Karamanos NK, Tzanakakis GN. Role of receptor for hyaluronic acid-mediatedmotility (RHAMM) in low molecular weight hyaluronan (LMWHA)-mediatedfibrosarcoma cell adhesion. J Biol Chem. 2011 Nov 4;286(44):38509-38520. doi:10.1074/jbc.M111.275875. Epub 2011 Sep 13. PMID: 21914806; PMCID: PMC3207394.  498: Kumari R, Li H, Haudenschild DR, Fierro F, Carlson CS, Overn P, Gupta L,Gupta K, Nolta J, Yik JH, Di Cesare PE. The oncogene LRF is a survival factor inchondrosarcoma and contributes to tumor malignancy and drug resistance.Carcinogenesis. 2012 Nov;33(11):2076-83. doi: 10.1093/carcin/bgs254. Epub 2012Jul 30. PMID: 22847180.  499: Raaijmakers JA, van Heesbeen RG, Meaders JL, Geers EF, Fernandez-Garcia B,Medema RH, Tanenbaum ME. Nuclear envelope-associated dynein drives prophasecentrosome separation and enables Eg5-independent bipolar spindle formation.EMBO J. 2012 Nov 5;31(21):4179-90. doi: 10.1038/emboj.2012.272. Epub 2012 Oct 2.PMID: 23034402; PMCID: PMC3492733.  500: Cheng L, Ke Y, Yu S, Jing J. Co-delivery of doxorubicin and recombinantplasmid pHSP70-Plk1-shRNA by bacterial magnetosomes for osteosarcoma therapy.Int J Nanomedicine. 2016 Oct 25;11:5277-5286. doi: 10.2147/IJN.S115364. PMID:27822032; PMCID: PMC5087786.  501: Whirledge SD, Jewell CM, Barber LM, Xu X, Katen KS, Garantziotis S, CidlowskiJA. Generating diversity in human glucocorticoid signaling through a raciallydiverse polymorphism in the beta isoform of the glucocorticoid receptor. LabInvest. 2017 Nov;97(11):1282-1295. doi: 10.1038/labinvest.2017.76. Epub 2017 Jul   1. PMID: 28759007; PMCID: PMC5759773.   502: Zhang X, Zu H, Zhao D, Yang K, Tian S, Yu X, Lu F, Liu B, Yu X, Wang B, WangW, Huang S, Wang Y, Wang Z, Zhang Z. Ion channel functional protein kinase TRPM7regulates Mg ions to promote the osteoinduction of human osteoblast via PI3Kpathway: In vitro simulation of the bone-repairing effect of Mg-based alloyimplant. Acta Biomater. 2017 Nov;63:369-382. doi: 10.1016/j.actbio.2017.08.051.Epub 2017 Sep 4. PMID: 28882757.  503:Zhang K, Shi H, Xi H, Wu X, Cui J, Gao Y, Liang W, Hu C, Liu Y, Li J, Wang N, Wei B, Chen L. Genome-Wide lncRNA Microarray Profiling Identifies Novel Circulating lncRNAs for Detection of Gastric Cancer. Theranostics. 2017. 7(1):213-27. doi: 10.7150/thno.16044. PMID: 28042329; PMCID: PMC5196898.  504: Oliveira-Mateos C, Sánchez-Castillo A, Soler M, Obiols-Guardia A, Piñeyro D,Boque-Sastre R, Calleja-Cervantes ME, Castro de Moura M, Martínez-Cardús A,Rubio T, Pelletier J, Martínez-Iniesta M, Herrero-Martín D, Tirado OM,Gentilella A, Villanueva A, Esteller M, Farré L, Guil S. The transcribedpseudogene RPSAP52 enhances the oncofetal HMGA2-IGF2BP2-RAS axis throughLIN28B-dependent and independent let-7 inhibition. Nat Commun. 2019 Sep4;10(1):3979. doi: 10.1038/s41467-019-11910-6. PMID: 31484926; PMCID:PMC6726650.  505: Veys C, Benmoussa A, Contentin R, Duchemin A, Brotin E, Lafont JE, SaintignyY, Poulain L, Denoyelle C, Demoor M, Legendre F, Galéra P. Tumor SuppressiveRole of miR-342-5p in Human Chondrosarcoma Cells and 3D Organoids. Int J MolSci. 2021 May 25;22(11):5590. doi: 10.3390/ijms22115590. PMID: 34070455; PMCID:PMC8197525.  506: Campbell J, Ryan CJ, Brough R, Bajrami I, Pemberton HN, Chong IY, Costa-Cabral S, Frankum J, Gulati A, Holme H, Miller R, Postel-Vinay S, Rafiq R, WeiW, Williamson CT, Quigley DA, Tym J, Al-Lazikani B, Fenton T, Natrajan R,Strauss SJ, Ashworth A, Lord CJ. Large-Scale Profiling of Kinase Dependencies inCancer Cell Lines. Cell Rep. 2016 Mar 15;14(10):2490-501. doi:10.1016/j.celrep.2016.02.023. Epub 2016 Mar 3. PMID: 26947069; PMCID:PMC4802229.  507: Chen W, Li N, Chen T, Han Y, Li C, Wang Y, He W, Zhang L, Wan T, Cao X. Thelysosome-associated apoptosis-inducing protein containing the pleckstrinhomology (PH) and FYVE domains (LAPF), representative of a novel family of PHand FYVE domain-containing proteins, induces caspase-independent apoptosis viathe lysosomal-mitochondrial pathway. J Biol Chem. 2005 Dec 9;280(49):40985-95.doi: 10.1074/jbc.M502190200. Epub 2005 Sep 27. Erratum in: J Biol Chem. 2021Jan-Jun;296:100764. PMID: 16188880.  508: Hu Y, Li X, Xue W, Pang J, Meng Y, Shen Y, Xu Q. TP53INP2-related basalautophagy is involved in the growth and malignant progression in humanliposarcoma cells. Biomed Pharmacother. 2017 Apr;88:562-568. doi:10.1016/j.biopha.2017.01.110. Epub 2017 Feb 24. PMID: 28131096.  509: Gong J, Zhang H, He L, Wang L, Wang J. Increased Expression of Long Non-Coding RNA BCAR4 Is Predictive of Poor Prognosis in Patients with Non-Small CellLung Cancer. Tohoku J Exp Med. 2017 Jan;241(1):29-34. doi: 10.1620/tjem.241.29.PMID: 28077810.  510: Human genome meeting 2016 : Houston, TX, USA. 28 February - 2 March 2016. Hum Genomics. 2016 May 26; 10 Suppl 1:12. doi: 10.1186/s40246-016-0063-5. PMID: 27294413; PMCID: PMC4896275.  511: Akara-Amornthum P, Lomphithak T, Choksi S, Tohtong R, Jitkaew S. Key necroptotic proteins are required for Smac mimetic-mediated sensitization of cholangiocarcinoma cells to TNF-alpha and chemotherapeutic gemcitabine-induced necroptosis. PLoS One. 2020; 15(1):e0227454. doi: 10.1371/journal.pone.0227454. PMID: 31914150; PMCID: PMC6948742.  512: Akimoto M, Susa T, Okudaira N, Hisaki H, Iizuka M, Okinaga H, Okazaki T, Tamamori-Adachi M. A novel LncRNA PTH-AS upregulates interferon-related DNA damage resistance signature genes and promotes metastasis in human breast cancer xenografts. J Biol Chem. 2022 Jul. 298(7):102065. doi: 10.1016/j.jbc.2022.102065. PMID: 35618021; PMCID: PMC9198338.  513: Alimova I, Birks DK, Harris PS, Knipstein JA, Venkataraman S, Marquez VE, Foreman NK, Vibhakar R. Inhibition of EZH2 suppresses self-renewal and induces radiation sensitivity in atypical rhabdoid teratoid tumor cells. Neuro Oncol. 2013 Feb. 15(2):149-60. doi: 10.1093/neuonc/nos285. PMID: 23190500; PMCID: PMC3548579.  514: Amirnasr A, Verdijk RM, van Kuijk PF, Kartal P, Vriends ALM, French PJ, van Royen ME, Taal W, Sleijfer S, Wiemer EAC. Deregulated microRNAs in neurofibromatosis type 1 derived malignant peripheral nerve sheath tumors. Sci Rep. 2020 Feb 19. 10(1):2927. doi: 10.1038/s41598-020-59789-4. PMID: 32076030; PMCID: PMC7031337.  515: Arcaro A, Doepfner KT, Boller D, Guerreiro AS, Shalaby T, Jackson SP, Schoenwaelder SM, Delattre O, Grotzer MA, Fischer B. Novel role for insulin as an autocrine growth factor for malignant brain tumour cells. Biochem J. 2007 Aug 15. 406(1):57-66. doi: 10.1042/bj20070309. PMID: 17506723; PMCID: PMC1948991.  516: Bai C, Yang W, Ouyang R, Li Z, Zhang L. Study of hsa_circRNA_000121 and hsa_circRNA_004183 in papillary thyroid microcarcinoma. Open Life Sci. 2022. 17(1):726-34. doi: 10.1515/biol-2022-0080. PMID: 35891968; PMCID: PMC9281586.  517: Barboro P, Benelli R, Tosetti F, Costa D, Capaia M, Astigiano S, Vene R, Poggi A, Ferrari N. Aspartate beta-hydroxylase targeting in castration-resistant prostate cancer modulates the NOTCH/HIF1alpha/GSK3beta crosstalk. Carcinogenesis. 2020 Sep 24. 41(9):1246-52. doi: 10.1093/carcin/bgaa053. PMID: 32525968.  518: Beltran AS, Rivenbark AG, Richardson BT, Yuan X, Quian H, Hunt JP, Zimmerman E, Graves LM, Blancafort P. Generation of tumor-initiating cells by exogenous delivery of OCT4 transcription factor. Breast Cancer Res. 2011 Sep 27. 13(5):R94. doi: 10.1186/bcr3019. PMID: 21952072; PMCID: PMC3262206.  519: Brock M, Hottinger S, Diebold M, Soltermann A, Jochum W, Kohler M, Huber LC, Franzen DP. Low tissue levels of miR-125b predict malignancy in solitary fibrous tumors of the pleura. Respir Res. 2017 Mar 2. 18(1):43. doi: 10.1186/s12931-017-0528-7. PMID: 28253927; PMCID: PMC5335791.  520: Calderaro J, Masliah-Planchon J, Richer W, Maillot L, Maille P, Mansuy L, Bastien C, de la Taille A, Boussion H, Charpy C, Jourdain A, Blechet C, Pierron G, Gentien D, Choudat L, Tournigand C, Delattre O, Allory Y, Bourdeaut F. Balanced Translocations Disrupting SMARCB1 Are Hallmark Recurrent Genetic Alterations in Renal Medullary Carcinomas. Eur Urol. 2016 Jun. 69(6):1055-61. doi: 10.1016/j.eururo.2015.09.027. PMID: 26433572.  521: Chasseur AS, Trozzi G, Istasse C, Petit A, Rasschaert P, Denesvre C, Kaufer BB, Bertzbach LD, Muylkens B, Coupeau D. Marek's Disease Virus Virulence Genes Encode Circular RNAs. J Virol. 2022 May 11. 96(9):e0032122. doi: 10.1128/jvi.00321-22. PMID: 35412345; PMCID: PMC9093110.  522: Chen H, Fu Y, Guo Z, Zhou X. MicroRNA-29c-3p participates in insulin function to modulate polycystic ovary syndrome via targeting Forkhead box O 3. Bioengineered. 2022 Feb. 13(2):4361-71. doi: 10.1080/21655979.2022.2033014. PMID: 35142592; PMCID: PMC8973910.  523: Chen J, Cui JD, Guo XT, Cao X, Li Q. Increased expression of miR-641 contributes to erlotinib resistance in non-small-cell lung cancer cells by targeting NF1. Cancer Med. 2018 Apr. 7(4):1394-403. doi: 10.1002/cam4.1326. PMID: 29493886; PMCID: PMC5911582.  524: Chen W, Chen Y, Liu L, Wu Y, Fu P, Cao Y, Xiong J, Tu Y, Li Z, Liu Y, Jie Z. Comprehensive Analysis of Immune Infiltrates of Ferroptosis-Related Long Noncoding RNA and Prediction of Colon Cancer Patient Prognoses. J Immunol Res. 2022. 2022:9480628. doi: 10.1155/2022/9480628. PMID: 35265722; PMCID: PMC8898846.  525: Chen Y, Zhai LY, Zhang LM, Ma XS, Liu Z, Li MM, Chen JX, Duan WJ. Breast cancer plasma biopsy by in situ determination of exosomal microRNA-1246 with a molecular beacon. Analyst. 2021 Apr 7. 146(7):2264-76. doi: 10.1039/d0an02224a. PMID: 33599630.  526: Chiou SH, Kao CL, Chen YW, Chien CS, Hung SC, Lo JF, Chen YJ, Ku HH, Hsu MT, Wong TT. Identification of CD133-positive radioresistant cells in atypical teratoid/rhabdoid tumor. PLoS One. 2008 May 7; 3(5):e2090. doi: 10.1371/journal.pone.0002090. PMID: 18509505; PMCID: PMC2396792.  527: Cui J, Dean D, Hornicek FJ, Yi G, Duan Z. Expression and Clinical Significance of High-Mobility Group AT-hook 2 (HMGA2) in Osteosarcoma. Orthop Surg. 2022 May. 14(5):955-66. doi: 10.1111/os.13167. PMID: 35388973; PMCID: PMC9087380.  528: Das B, Jain N, Mallick B. piR-39980 mediates doxorubicin resistance in fibrosarcoma by regulating drug accumulation and DNA repair. Commun Biol. 2021 Nov 19. 4(1):1312. doi: 10.1038/s42003-021-02844-1. PMID: 34799689; PMCID: PMC8605029.  529: Delort L, Cholet J, Decombat C, Vermerie M, Dumontet C, Castelli FA, Fenaille F, Auxenfans C, Rossary A, Caldefie-Chezet F. The Adipose Microenvironment Dysregulates the Mammary Myoepithelial Cells and Could Participate to the Progression of Breast Cancer. Front Cell Dev Biol. 2020. 8:571948. doi: 10.3389/fcell.2020.571948. PMID: 33505957; PMCID: PMC7829501.  530: Gheidari F, Arefian E, Jamshidi Adegani F, Fallah Atanaki F, Soleimani M. The miR-142 Suppresses U-87 Glioblastoma Cell Growth by Targeting EGFR Oncogenic Signaling Pathway. Iran J Pharm Res. 2021 Fall. 20(4):202-12. doi: 10.22037/ijpr.2021.115089.15193. PMID: 35194440; PMCID: PMC8842594.  531: Griffiths S, Thompson P, Frayling I, Upadhyaya M. Molecular diagnosis of neurofibromatosis type 1: 2 years experience. Fam Cancer. 2007. 6(1):21-34. doi: 10.1007/s10689-006-9001-3. PMID: 16944272.  532: Han J, Hu Y, Liu S, Jiang J, Wang H. A Newly Established Cuproptosis-Associated Long Non-Coding RNA Signature for Predicting Prognosis and Indicating Immune Microenvironment Features in Soft Tissue Sarcoma. J Oncol. 2022. 2022:8489387. doi: 10.1155/2022/8489387. PMID: 35847354; PMCID: PMC9279026.  533: He Q, Hao P, He G, Mai H, Liu W, Zhang W, Zhang K, Zhong G, Guo R, Yu C, Li Y, Wong C, Chen Q, Chen Y. IGF2BP1-regulated expression of ERRalpha is involved in metabolic reprogramming of chemotherapy resistant osteosarcoma cells. J Transl Med. 2022 Aug 2. 20(1):348. doi: 10.1186/s12967-022-03549-7. PMID: 35918761; PMCID: PMC9344706.  534: Hosseini F, Shanehbandi D, Soleimanpour J, Yousefi B, Alemi F. Melatonin Increases the Sensitivity of Osteosarcoma Cells to Chemotherapy Drug Cisplatin. Drug Res (Stuttg). 2022 Jul. 72(6):312-8. doi: 10.1055/a-1830-8716. PMID: 35636434.  535: Hu X, Wen Y, Tan LY, Wang J, Tang F, Wang YT, Zheng CX, Zhang YQ, Gong TJ, Min L. Exosomal Long Non-Coding RNA ANCR Mediates Drug Resistance in Osteosarcoma. Front Oncol. 2021. 11:735254. doi: 10.3389/fonc.2021.735254. PMID: 35096563; PMCID: PMC8789737.  536: James MF, Han S, Polizzano C, Plotkin SR, Manning BD, Stemmer-Rachamimov AO, Gusella JF, Ramesh V. NF2/merlin is a novel negative regulator of mTOR complex 1, and activation of mTORC1 is associated with meningioma and schwannoma growth. Mol Cell Biol. 2009 Aug. 29(15):4250-61. doi: 10.1128/mcb.01581-08. PMID: 19451225; PMCID: PMC2715803.  537: Korshunov A, Ryzhova M, Jones DT, Northcott PA, van Sluis P, Volckmann R, Koster J, Versteeg R, Cowdrey C, Perry A, Picard D, Rosenblum M, Giangaspero F, Aronica E, Schuller U, Hasselblatt M, Collins VP, von Deimling A, Lichter P, Huang A, Pfister SM, Kool M. LIN28A immunoreactivity is a potent diagnostic marker of embryonal tumor with multilayered rosettes (ETMR). Acta Neuropathol. 2012 Dec. 124(6):875-81. doi: 10.1007/s00401-012-1068-3. PMID: 23161096; PMCID: PMC3508282.  538: Kwon J, Lee JH, Lee YH, Lee J, Ahn JH, Kim SH, Kim SH, Kim TI, Yun KH, Park YS, Kim JE, Lee KS, Choi JK, Kim HS. Whole-genome and Transcriptome Sequencing Identified NOTCH2 and HES1 as Potential Markers of Response to Imatinib in Desmoid Tumor (Aggressive Fibromatosis): A Phase II Trial Study. Cancer Res Treat. 2022 Jan 17. doi: 10.4143/crt.2021.1194. PMID: 35038826.  539: Labourier E, Shifrin A, Busseniers AE, Lupo MA, Manganelli ML, Andruss B, Wylie D, Beaudenon-Huibregtse S. Molecular Testing for miRNA, mRNA, and DNA on Fine-Needle Aspiration Improves the Preoperative Diagnosis of Thyroid Nodules With Indeterminate Cytology. J Clin Endocrinol Metab. 2015 Jul. 100(7):2743-50. doi: 10.1210/jc.2015-1158. PMID: 25965083; PMCID: PMC4490308.  540: Landis JT, Tuck R, Pan Y, Mosso CN, Eason AB, Moorad R, Marron JS, Dittmer DP. Evidence for Multiple Subpopulations of Herpesvirus-Latently Infected Cells. mBio. 2022 Jan. 4:e0347321. doi: 10.1128/mbio.03473-21. PMID: 35089062; PMCID: PMC8725583.  541: Levy JM, Thorburn A. Modulation of pediatric brain tumor autophagy and chemosensitivity. J Neurooncol. 2012 Jan. 106(2):281-90. doi: 10.1007/s11060-011-0684-4. PMID: 21842312; PMCID: PMC3811079.  542: Li J, Xiang R, Song W, Wu J, Kong C, Fu T. A Novel Ferroptosis-Related LncRNA Pair Prognostic Signature Predicts Immune Landscapes and Treatment Responses for Gastric Cancer Patients. Front Genet. 2022; 13:899419. doi: 10.3389/fgene.2022.899419. PMID: 35795206; PMCID: PMC9250987  543: Lin L, Wang H, Guo W, He E, Huang K, Zhao Q. Osteosarcoma-derived exosomal miR-501-3p promotes osteoclastogenesis and aggravates bone loss. Cell Signal. 2021 Jun. 82:109935. doi: 10.1016/j.cellsig.2021.109935. PMID: 33529755.  544: Liu B, Liu Z, Feng C, Tu C. A Necroptosis-Related lncRNA Signature Predicts Prognosis and Indicates the Immune Microenvironment in Soft Tissue Sarcomas. Front Genet. 2022. 13:899545. doi: 10.3389/fgene.2022.899545. PMID: 35795204; PMCID: PMC9251335.  545: Macveigh-Fierro D, Cicerchia A, Cadorette A, Sharma V, Muller M. The m(6)A reader YTHDC2 is essential for escape from KSHV SOX-induced RNA decay. Proc Natl Acad Sci U S A. 2022 Feb 22. 119(8). doi: 10.1073/pnas.2116662119. PMID: 35177478; PMCID: PMC8872733.  546: Murray MJ, Bell E, Raby KL, Rijlaarsdam MA, Gillis AJ, Looijenga LH, Brown H, Destenaves B, Nicholson JC, Coleman N. A pipeline to quantify serum and cerebrospinal fluid microRNAs for diagnosis and detection of relapse in paediatric malignant germ-cell tumours. Br J Cancer. 2016 Jan 19. 114(2):151-62. doi: 10.1038/bjc.2015.429. PMID: 26671749; PMCID: PMC4815809.  547: Pan YZ, Wang X, Bai H, Wang CB, Zhang Q, Xi R. Autophagy in drug resistance of the multiple myeloma cell line RPMI8226 to doxorubicin. Genet Mol Res. 2015 May 25. 14(2):5621-9. doi: 10.4238/2015.May.25.14. PMID: 26125760.  548: Peng DH, Kundu ST, Fradette JJ, Diao L, Tong P, Byers LA, Wang J, Canales JR, Villalobos PA, Mino B, Yang Y, Minelli R, Peoples MD, Bristow CA, Heffernan TP, Carugo A, Wistuba I, Gibbons DL. ZEB1 suppression sensitizes KRAS mutant cancers to MEK inhibition by an IL17RD-dependent mechanism. Sci Transl Med. 2019 Mar 13. 11(483). doi: 10.1126/scitranslmed.aaq1238. PMID: 30867319; PMCID: PMC6878763.  549: Rossi F, Beltran M, Damizia M, Grelloni C, Colantoni A, Setti A, Di Timoteo G, Dattilo D, Centron-Broco A, Nicoletti C, Fanciulli M, Lavia P, Bozzoni I. Circular RNA ZNF609/CKAP5 mRNA interaction regulates microtubule dynamics and tumorigenicity. Mol Cell. 2022 Jan 6. 82(1):75-89 e9. doi: 10.1016/j.molcel.2021.11.032. PMID: 34942120; PMCID: PMC8751636.  550: Sato K, Osaka E, Fujiwara K, Fujii R, Takayama T, Tokuhashi Y, Nakanishi K. miRNA218 targets multiple oncogenes and is a therapeutic target for osteosarcoma. Oncol Rep. 2022 May. 47(5). doi: 10.3892/or.2022.8303. PMID: 35293593; PMCID: PMC8968766.  551: Sharma H, Niveditha D, Chowdhury R, Mukherjee S, Chowdhury S. A genome-wide expression profile of noncoding RNAs in human osteosarcoma cells as they acquire resistance to cisplatin. Discov Oncol. 2021 Oct 20. 12(1):43. doi: 10.1007/s12672-021-00441-6. PMID: 35201486; PMCID: PMC8777531.  552: Sistrunk JW, Shifrin A, Frager M, Bardales RH, Thomas J, Fishman N, Goldberg P, Guttler R, Grant E. Clinical impact of testing for mutations and microRNAs in thyroid nodules. Diagn Cytopathol. 2019 Aug. 47(8):758-64. doi: 10.1002/dc.24190. PMID: 31013001; PMCID: PMC6766884.  553: Song Y, Park IS, Kim J, Seo HR. Actinomycin D inhibits the expression of the cystine/glutamate transporter xCT via attenuation of CD133 synthesis in CD133(+) HCC. Chem Biol Interact. 2019 Aug 25. 309:108713. doi: 10.1016/j.cbi.2019.06.026. PMID: 31226288.  554: Umezu T, Tadokoro H, Azuma K, Yoshizawa S, Ohyashiki K, Ohyashiki JH. Exosomal miR-135b shed from hypoxic multiple myeloma cells enhances angiogenesis by targeting factor-inhibiting HIF-1. Blood. 2014 Dec 11. 124(25):3748-57. doi: 10.1182/blood-2014-05-576116. PMID: 25320245; PMCID: PMC4263983.  555: Verbeke S, Richard E, Monceau E, Schmidt X, Rousseau B, Velasco V, Bernard D, Bonnefoi H, MacGrogan G, Iggo RD. Humanization of the mouse mammary gland by replacement of the luminal layer with genetically engineered preneoplastic human cells. Breast Cancer Res. 2014 Dec 20. 16(6):504. doi: 10.1186/s13058-014-0504-9. PMID: 25527189; PMCID: PMC4407301.  556: Wang J, Yang K, Yuan W, Gao Z. Determination of Serum Exosomal H19 as a Noninvasive Biomarker for Bladder Cancer Diagnosis and Prognosis. Med Sci Monit. 2018 Dec 21. 24:9307-16. doi: 10.12659/msm.912018. PMID: 30576305; PMCID: PMC6320644.  557: Wei G, Peng Z, Liu J, Yang K, Zhao C, Xie W, Huang T, Liu J, Li J, An G. Accurate Identification and Early Diagnosis of Osteosarcoma through CRISPR-Cas12a-Based Average Telomerase Activity Detection. ACS Synth Biol. 2021 Sep 17. 10(9):2409-16. doi: 10.1021/acssynbio.1c00389. PMID: 34495650.  558: Weng Y, Chen Y, Chen J, Liu Y, Bao T. Identification of serum microRNAs in genome-wide serum microRNA expression profiles as novel noninvasive biomarkers for malignant peripheral nerve sheath tumor diagnosis. Med Oncol. 2013 Jun. 30(2):531. doi: 10.1007/s12032-013-0531-x. PMID: 23483452.  559: Wu AC, Yang WB, Chang KY, Lee JS, Liou JP, Su RY, Cheng SM, Hwang DY, Kikkawa U, Hsu TI, Wang CY, Chang WC, Chen PY, Chuang JY. HDAC6 involves in regulating the lncRNA-microRNA-mRNA network to promote the proliferation of glioblastoma cells. J Exp Clin Cancer Res. 2022 Feb 2. 41(1):47. doi: 10.1186/s13046-022-02257-w. PMID: 35109908; PMCID: PMC8809020  560: Wu Z, Zhang X, Chen D, Li Z, Wu X, Wang J, Deng Y. N6-Methyladenosine-Related LncRNAs Are Potential Remodeling Indicators in the Tumor Microenvironment and Prognostic Markers in Osteosarcoma. Front Immunol. 2021. 12:806189. doi: 10.3389/fimmu.2021.806189. PMID: 35095893; PMCID: PMC8790065.  561: Xie G, Zhu Y, Lin Z, Sun Y, Gu G, Wang W, Chen H. HOPMCLDA: predicting lncRNA-disease associations based on high-order proximity and matrix completion. Mol Omics. 2021 Oct 11. 17(5):760-8. doi: 10.1039/d1mo00138h. PMID: 34251001.  562: Xu K, Zhang Q, Chen M, Li B, Wang N, Li C, Gao Z, Zhang D, Yang L, Xu Z, Li X, Xu H. N(6)-methyladenosine modification regulates imatinib resistance of gastrointestinal stromal tumor by enhancing the expression of multidrug transporter MRP1. Cancer Lett. 2022 Apr 1. 530:85-99. doi: 10.1016/j.canlet.2022.01.008. PMID: 35032557.  563: Xu R, Greening DW, Chen M, Rai A, Ji H, Takahashi N, Simpson RJ. Surfaceome of Exosomes Secreted from the Colorectal Cancer Cell Line SW480: Peripheral and Integral Membrane Proteins Analyzed by Proteolysis and TX114. Proteomics. 2019 Apr. 19(8):e1700453. doi: 10.1002/pmic.201700453. PMID: 30865381.  564: Xue Y, Zhu X, Meehan B, Venneti S, Martinez D, Morin G, Maiga RI, Chen H, Papadakis AI, Johnson RM, O'Sullivan MJ, Erdreich-Epstein A, Gotlieb WH, Park M, Judkins AR, Pelletier J, Foulkes WD, Rak J, Huang S. SMARCB1 loss induces druggable cyclin D1 deficiency via upregulation of MIR17HG in atypical teratoid rhabdoid tumors. J Pathol. 2020 Sep. 252(1):77-87. doi: 10.1002/path.5493. PMID: 32558936.  565: Yang M, Zheng H, Xu K, Yuan Q, Aihaiti Y, Cai Y, Xu P. A novel signature to guide osteosarcoma prognosis and immune microenvironment: Cuproptosis-related lncRNA. Front Immunol. 2022. 13:919231. doi: 10.3389/fimmu.2022.919231. PMID: 35967366; PMCID: PMC9373797.  566: Zhang JT, Qin H, Man Cheung FK, Su J, Zhang DD, Liu SY, Li XF, Qin J, Lin JT, Jiang BY, Song D, Liao RQ, Qiang N, Yang XN, Tu HY, Zhou Q, Yang JJ, Zhang XC, Zhang YN, Wu YL, Zhong WZ. Plasma extracellular vesicle microRNAs for pulmonary ground-glass nodules. J Extracell Vesicles. 2019. 8(1):1663666. doi: 10.1080/20013078.2019.1663666. PMID: 31579436; PMCID: PMC6758624.  567: Yin G, Fu B, Xu B, Han J, Xue Y, Chen H, Zhang X, Wang G. Identification of osteosarcoma by microRNA-coupled nuclease digestion on interdigitated electrode sensor. Biotechnol Appl Biochem. 2022 Jun. 69(3):1094-100. doi: 10.1002/bab.2180. PMID: 33987861.  568: Yoshikawa R, Maeda A, Ueno Y, Sakai H, Kimura S, Sawadaishi T, Kohgo S, Yamada K, Mori T. Intraperitoneal administration of synthetic microRNA-214 elicits tumor suppression in an intraperitoneal dissemination mouse model of canine hemangiosarcoma. Vet Res Commun. 2022 Jun. 46(2):447-57. doi: 10.1007/s11259-021-09869-1. PMID: 34988875.  569: Zhan T, Zhu K, Hu J, Ma X, Zhu Y, Zhang C. LncRNA ODRUL regulates progression of osteosarcoma by regulating IL-6 via sponging miR-6874-3p. Exp Cell Res. 2022 Mar 15. 412(2):113050. doi: 10.1016/j.yexcr.2022.113050. PMID: 35114192.  570: Zhang A, Hu H. A Novel Blood-Based microRNA Diagnostic Model with High Accuracy for Multi-Cancer Early Detection. Cancers (Basel). 2022 Mar 11. 14(6). doi: 10.3390/cancers14061450. PMID: 35326599; PMCID: PMC8946599.  571: Zhang DW, Wang HG, Zhang KB, Guo YQ, Yang LJ, Lv H. LncRNA XIST facilitates S1P-mediated osteoclast differentiation via interacting with FUS. J Bone Miner Metab. 2022 Mar. 40(2):240-50. doi: 10.1007/s00774-021-01294-3. PMID: 35066669. |
| **Reviews (N = 127)** |
| 572: Czarnecka AM, Synoradzki K, Firlej W, Bartnik E, Sobczuk P, Fiedorowicz M,Grieb P, Rutkowski P. Molecular Biology of Osteosarcoma. Cancers (Basel). 2020Jul 31;12(8):2130. doi: 10.3390/cancers12082130. PMID: 32751922; PMCID:PMC7463657.  573: Argenziano M, Tortora C, Pota E, Di Paola A, Di Martino M, Di Leva C, DiPinto D, Rossi F. Osteosarcoma in Children: Not Only Chemotherapy.Pharmaceuticals (Basel). 2021 Sep 13;14(9):923. doi: 10.3390/ph14090923. PMID:34577623; PMCID: PMC8471047.  574: Kok VC, Yu CC. Cancer-Derived Exosomes: Their Role in Cancer Biology andBiomarker Development. Int J Nanomedicine. 2020 Oct 19;15:8019-8036. doi:10.2147/IJN.S272378. PMID: 33116515; PMCID: PMC7585279.  575: Gentile M, Centonza A, Lovero D, Palmirotta R, Porta C, Silvestris F,D'Oronzo S. Application of "omics" sciences to the prediction of bone metastasesfrom breast cancer: State of the art. J Bone Oncol. 2020 Nov 5;26:100337. doi:10.1016/j.jbo.2020.100337. PMID: 33240786; PMCID: PMC7672315.  576: Yang L, Chen S, Luo P, Yan W, Wang C. Liposarcoma: Advances in Cellular andMolecular Genetics Alterations and Corresponding Clinical Treatment. J Cancer.2020 Jan 1;11(1):100-107. doi: 10.7150/jca.36380. PMID: 31892977; PMCID:PMC6930414.  577: D'Oronzo S, Brown J, Coleman R. The role of biomarkers in the management ofbone-homing malignancies. J Bone Oncol. 2017 Sep 11;9:1-9. doi:10.1016/j.jbo.2017.09.001. PMID: 28948139; PMCID: PMC5602513.  578: Sarkar D, Leung EY, Baguley BC, Finlay GJ, Askarian-Amiri ME. Epigeneticregulation in human melanoma: past and future. Epigenetics. 2015;10(2):103-21.doi: 10.1080/15592294.2014.1003746. PMID: 25587943; PMCID: PMC4622872.  579: Wang JY, Yang Y, Ma Y, Wang F, Xue A, Zhu J, Yang H, Chen Q, Chen M, Ye L, WuH, Zhang Q. Potential regulatory role of lncRNA-miRNA-mRNA axis in osteosarcoma.Biomed Pharmacother. 2020 Jan;121:109627. doi: 10.1016/j.biopha.2019.109627.Epub 2019 Nov 20. PMID: 31810120.  580: Jeong W, Kim HJ. Biomarkers of chondrosarcoma. J Clin Pathol. 2018Jul;71(7):579-583. doi: 10.1136/jclinpath-2018-205071. Epub 2018 Mar 28. PMID:29593061; PMCID: PMC6204964.  581: Elton TS, Selemon H, Elton SM, Parinandi NL. Regulation of the MIR155 hostgene in physiological and pathological processes. Gene. 2013 Dec 10;532(1):1-12.doi: 10.1016/j.gene.2012.12.009. Epub 2012 Dec 14. PMID: 23246696.  582: Tang P, Xiong Q, Ge W, Zhang L. The role of microRNAs in osteoclasts andosteoporosis. RNA Biol. 2014;11(11):1355-63. doi: 10.1080/15476286.2014.996462.PMID: 25692234; PMCID: PMC4615571.  583: Chen R, Wang G, Zheng Y, Hua Y, Cai Z. Long non-coding RNAs in osteosarcoma.Oncotarget. 2017 Mar 21;8(12):20462-20475. doi: 10.18632/oncotarget.14726. PMID:28103585; PMCID: PMC5386777.  584: Guo CM, Liu SQ, Sun MZ. miR-429 as biomarker for diagnosis, treatment andprognosis of cancers and its potential action mechanisms: A systematicliterature review. Neoplasma. 2020 Mar;67(2):215-228. doi:10.4149/neo_2019_190401N282. Epub 2019 Dec 23. PMID: 31884798.  585: Lambrou GI, Hatziagapiou K, Zaravinos A. The Non-Coding RNA <i>GAS5</i> andIts Role in Tumor Therapy-Induced Resistance. Int J Mol Sci. 2020 Oct15;21(20):7633. doi: 10.3390/ijms21207633. PMID: 33076450; PMCID: PMC7588928.  586: Zhang J, Yan YG, Wang C, Zhang SJ, Yu XH, Wang WJ. MicroRNAs in osteosarcoma.Clin Chim Acta. 2015 Apr 15;444:9-17. doi: 10.1016/j.cca.2015.01.025. Epub 2015Feb 4. PMID: 25661090.  587: Li X, Shen JK, Hornicek FJ, Xiao T, Duan Z. Noncoding RNA in drug resistantsarcoma. Oncotarget. 2017 Jul 6;8(40):69086-69104. doi:10.18632/oncotarget.19029. PMID: 28978183; PMCID: PMC5620323.  588: Zhang Y, Pu Y, Wang J, Li Z, Wang H. Research progress regarding the role oflong non-coding RNAs in osteosarcoma. Oncol Lett. 2020 Sep;20(3):2606-2612. doi:10.3892/ol.2020.11807. Epub 2020 Jul 3. PMID: 32782578; PMCID: PMC7400499.  589: Tu C, He J, Qi L, Ren X, Zhang C, Duan Z, Yang K, Wang W, Lu Q, Li Z.Emerging landscape of circular RNAs as biomarkers and pivotal regulators inosteosarcoma. J Cell Physiol. 2020 Dec;235(12):9037-9058. doi:10.1002/jcp.29754. Epub 2020 May 26. PMID: 32452026.  590: Amirnasr A, Sleijfer S, Wiemer EAC. Non-Coding RNAs, a Novel Paradigm forthe Management of Gastrointestinal Stromal Tumors. Int J Mol Sci. 2020 Sep22;21(18):6975. doi: 10.3390/ijms21186975. PMID: 32972022; PMCID: PMC7555847.  591: Ahmad O, Chan M, Savage P, Watabe K, Lo HW, Qasem S. Biology and treatmentof metastasis of sarcoma to the brain. Front Biosci (Elite Ed). 2016 Jan1;8:233-44. doi: 10.2741/E764. PMID: 26709659.  592: Chang L, Shrestha S, LaChaud G, Scott MA, James AW. Review of microRNA inosteosarcoma and chondrosarcoma. Med Oncol. 2015 Jun;32(6):613. doi:10.1007/s12032-015-0613-z. Epub 2015 Apr 26. PMID: 25920607.  593: Bure I, Haller F, Zaletaev DV. Coding and Non-coding: Molecular Portrait ofGIST and its Clinical Implication. Curr Mol Med. 2018;18(4):252-259. doi:10.2174/1566524018666181004113436. PMID: 30289069.  594: Chen C, Shu L, Zou W. Role of long non-coding RNA TP73-AS1 in cancer. BiosciRep. 2019 Oct 30;39(10):BSR20192274. doi: 10.1042/BSR20192274. PMID: 31652459;PMCID: PMC6822500.  595: Li Z, Yu X, Shen J, Wu WK, Chan MT. MicroRNA expression and its clinicalimplications in Ewing's sarcoma. Cell Prolif. 2015 Feb;48(1):1-6. doi:10.1111/cpr.12160. Epub 2014 Dec 22. PMID: 25530497; PMCID: PMC6496123.  596: Matsuzaki J, Ochiya T. Circulating microRNAs: Next-generation CancerDetection. Keio J Med. 2020 Dec 25;69(4):88-96. doi: 10.2302/kjm.2019-0011-OA.Epub 2020 May 13. PMID: 32404538.  597: Kwan JY, Psarianos P, Bruce JP, Yip KW, Liu FF. The complexity of microRNAsin human cancer. J Radiat Res. 2016 Aug;57 Suppl 1(Suppl 1):i106-i111. doi:10.1093/jrr/rrw009. Epub 2016 Mar 16. PMID: 26983984; PMCID: PMC4990105.  598: Palmini G, Marini F, Brandi ML. What Is New in the miRNA World RegardingOsteosarcoma and Chondrosarcoma? Molecules. 2017 Mar 7;22(3):417. doi:10.3390/molecules22030417. PMID: 28272374; PMCID: PMC6155266.  599: Papatsirou M, Artemaki PI, Scorilas A, Kontos CK. The role of circular RNAsin therapy resistance of patients with solid tumors. Per Med. 2020Nov;17(6):469-490. doi: 10.2217/pme-2020-0103. Epub 2020 Oct 14. PMID: 33052780.  600: Chen R, Wang G, Zheng Y, Hua Y, Cai Z. Drug resistance-related microRNAs inosteosarcoma: Translating basic evidence into therapeutic strategies. J Cell MolMed. 2019 Apr;23(4):2280-2292. doi: 10.1111/jcmm.14064. Epub 2019 Feb 5. PMID:30724027; PMCID: PMC6433687.  601: Jasek K, Kasubova I, Holubekova V, Stanclova A, Plank L, Lasabova Z.Epigenetics: an alternative pathway in GISTs tumorigenesis. Neoplasma.2018;65(4):477-493. doi: 10.4149/neo_2018_170726N504. PMID: 29940762.  602: Li Z, Dou P, Liu T, He S. Application of Long Noncoding RNAs inOsteosarcoma: Biomarkers and Therapeutic Targets. Cell Physiol Biochem.2017;42(4):1407-1419. doi: 10.1159/000479205. Epub 2017 Jul 17. PMID: 28715796.  603: Li J, Yang Z, Li Y, Xia J, Li D, Li H, Ren M, Liao Y, Yu S, Chen Y, Yang Y,Zhang Y. Cell apoptosis, autophagy and necroptosis in osteosarcoma treatment.Oncotarget. 2016 Jul 12;7(28):44763-44778. doi: 10.18632/oncotarget.8206. PMID:27007056; PMCID: PMC5190133.  604: Yamamoto H, Oda Y. Gastrointestinal stromal tumor: recent advances inpathology and genetics. Pathol Int. 2015 Jan;65(1):9-18. doi: 10.1111/pin.12230.Epub 2014 Nov 20. PMID: 25414046.  605: Yang Z, Li X, Yang Y, He Z, Qu X, Zhang Y. Long noncoding RNAs in theprogression, metastasis, and prognosis of osteosarcoma. Cell Death Dis. 2016 Sep29;7(9):e2389. doi: 10.1038/cddis.2016.272. PMID: 27685633; PMCID: PMC5059871.  606: Chen D, Liu D, Chen Z. Potential therapeutic implications of miRNAs inosteosarcoma chemotherapy. Tumour Biol. 2017 Sep;39(9):1010428317705762. doi:10.1177/1010428317705762. PMID: 28933259.  607: Miao J, Wu S, Peng Z, Tania M, Zhang C. MicroRNAs in osteosarcoma:diagnostic and therapeutic aspects. Tumour Biol. 2013 Aug;34(4):2093-8. doi:10.1007/s13277-013-0940-7. PMID: 23797816.  608: Xu S, Gong Y, Yin Y, Xing H, Zhang N. The multiple function of longnoncoding RNAs in osteosarcoma progression, drug resistance and prognosis.Biomed Pharmacother. 2020 Jul;127:110141. doi: 10.1016/j.biopha.2020.110141.Epub 2020 Apr 22. PMID: 32334375.  609: Min L, Garbutt C, Tu C, Hornicek F, Duan Z. Potentials of Long NoncodingRNAs (LncRNAs) in Sarcoma: From Biomarkers to Therapeutic Targets. Int J MolSci. 2017 Mar 29;18(4):731. doi: 10.3390/ijms18040731. PMID: 28353666; PMCID:PMC5412317.  610: Fudalej MM, Badowska-Kozakiewicz AM. Improved understanding ofgastrointestinal stromal tumors biology as a step for developing new diagnosticand therapeutic schemes. Oncol Lett. 2021 May;21(5):417. doi:10.3892/ol.2021.12678. Epub 2021 Mar 28. PMID: 33841578; PMCID: PMC8020393.  611: Hosseini F, Alemi F, Malakoti F, Mahmoodpoor A, Younesi S, Yousefi B, AsemiZ. Targeting Wnt/β-catenin signaling by microRNAs as a therapeutic approach inchemoresistant osteosarcoma. Biochem Pharmacol. 2021 Nov;193:114758. doi:10.1016/j.bcp.2021.114758. Epub 2021 Sep 3. PMID: 34481813.  612: Gao SS, Wang YJ, Zhang GX, Zhang WT. Potential diagnostic value of miRNAs inperipheral blood for osteosarcoma: A meta-analysis. J Bone Oncol. 2020 Jul15;23:100307. doi: 10.1016/j.jbo.2020.100307. PMID: 32742918; PMCID: PMC7385506.  613: Colombo M, Platonova N, Giannandrea D, Palano MT, Basile A, Chiaramonte R.Re-establishing Apoptosis Competence in Bone Associated Cancers viaCommunicative Reprogramming Induced Through Notch Signaling Inhibition. FrontPharmacol. 2019 Feb 27;10:145. doi: 10.3389/fphar.2019.00145. PMID: 30873026;PMCID: PMC6400837.  614: Wang D, Yang S, Wang H, Wang J, Zhang Q, Zhou S, He Y, Zhang H, Deng F, XuH, Zhong S, Fu L, Tang J. The progress of circular RNAs in various tumors. Am JTransl Res. 2018 Jun 15;10(6):1571-1582. PMID: 30018701; PMCID: PMC6038087.  615: Miller IV, Grunewald TG. Tumour-derived exosomes: Tiny envelopes for bigstories. Biol Cell. 2015 Sep;107(9):287-305. doi: 10.1111/boc.201400095. Epub2015 Jun 9. PMID: 25923825.  616: Yang J, Li S, Wang B, Wu Y, Chen Z, Lv M, Lin Y, Yang J. Potentialbiomarkers for anti-EGFR therapy in metastatic colorectal cancer. Tumour Biol.2016 Sep;37(9):11645-11655. doi: 10.1007/s13277-016-5140-9. Epub 2016 Jul 16.PMID: 27422777.  617: Yang J, Zhang W. New molecular insights into osteosarcoma targeted therapy.Curr Opin Oncol. 2013 Jul;25(4):398-406. doi: 10.1097/CCO.0b013e3283622c1b.PMID: 23666471.  618: Xu Y, Yu X, Zhang M, Zheng Q, Sun Z, He Y, Guo W. Promising Advances inLINC01116 Related to Cancer. Front Cell Dev Biol. 2021 Oct 14;9:736927. doi:10.3389/fcell.2021.736927. PMID: 34722518; PMCID: PMC8553226.  619: Basset-Séguin N. Quoi de neuf en dermato-cancérologie ? [What's new indermato-oncology?]. Ann Dermatol Venereol. 2011 Dec;138 Suppl 4:S253-62. French.doi: 10.1016/S0151-9638(11)70099-0. PMID: 22202647.  620: Gnoni A, Licchetta A, Memeo R, Argentiero A, Solimando AG, Longo V,Delcuratolo S, Brunetti O. Role of BRAF in Hepatocellular Carcinoma: A Rationalefor Future Targeted Cancer Therapies. Medicina (Kaunas). 2019 Nov 21;55(12):754.doi: 10.3390/medicina55120754. PMID: 31766556; PMCID: PMC6956203.  621: Liu Y, Zhang Y, Chen C, Li Y. lncRNA HIF1A-AS2: A potential oncogene inhuman cancers (Review). Biomed Rep. 2021 Oct;15(4):85. doi:10.3892/br.2021.1461. Epub 2021 Aug 13. PMID: 34512973; PMCID: PMC8411487.  622: Akiyama BM, Eiler D, Kieft JS. Structured RNAs that evade or confoundexonucleases: function follows form. Curr Opin Struct Biol. 2016 Feb;36:40-7.doi: 10.1016/j.sbi.2015.12.006. Epub 2016 Jan 12. PMID: 26797676; PMCID:PMC5388000.  623: Huang W, Wu Y, Qiao M, Xie Z, Cen X, Huang X, Zhao Z. CircRNA-miRNA networksin regulating bone disease. J Cell Physiol. 2021 Nov 19. doi: 10.1002/jcp.30625.Epub ahead of print. PMID: 34796958.  624: Fratini L, Jaeger M, de Farias CB, Brunetto AT, Brunetto AL, Shaw L, RoeslerR. Oncogenic functions of ZEB1 in pediatric solid cancers: interplays withmicroRNAs and long noncoding RNAs. Mol Cell Biochem. 2021 Nov;476(11):4107-4116.doi: 10.1007/s11010-021-04226-x. Epub 2021 Jul 22. PMID: 34292482.  625: Zhong J, Zhang G, Yao W. Clinicopathologic significance and prognostic valueof circRNAs in osteosarcoma: a systematic review and meta-analysis. J OrthopSurg Res. 2021 Oct 7;16(1):578. doi: 10.1186/s13018-021-02568-2. PMID: 34620208;PMCID: PMC8495992.  626: Dreyfus DH. Herpesviruses and the microbiome. J Allergy Clin Immunol. 2013Dec;132(6):1278-86. doi: 10.1016/j.jaci.2013.02.039. Epub 2013 Apr 20. PMID:23611298.  627: Zeng X, Cao Z, Luo W, Zheng L, Zhang T. MicroRNA-381-A Key TranscriptionalRegulator: Its Biological Function and Clinical Application Prospects in Cancer.Front Oncol. 2020 Nov 26;10:535665. doi: 10.3389/fonc.2020.535665. PMID:33324542; PMCID: PMC7726430.  628: Ebrahimi N, Aslani S, Babaie F, Hemmatzadeh M, Pourmoghadam Z, Azizi G,Jadidi-Niaragh F, Mohammadi H. MicroRNAs Implications in the Onset, Diagnosis,and Prognosis of Osteosarcoma. Curr Mol Med. 2021;21(7):573-588. doi:10.2174/1566524020999201203212824. PMID: 33272173.  629: Tang Z, Lu Y, Chen Y, Zhang J, Chen Z, Wang Q. Research Progress of MicroRNAin Chemotherapy Resistance of Osteosarcoma. Technol Cancer Res Treat. 2021 Jan-Dec;20:15330338211034262. doi: 10.1177/15330338211034262. PMID: 34323141; PMCID:PMC8326994.  630: He H, Ni J, Huang J. Molecular mechanisms of chemoresistance in osteosarcoma(Review). Oncol Lett. 2014 May;7(5):1352-1362. doi: 10.3892/ol.2014.1935. Epub2014 Mar 4. PMID: 24765137; PMCID: PMC3997672.  631: Li Y, Liu YH, Chen X, Zhu YJ, Zhang HB, Li Y, Bai JP, Liu LR, Qu YC, Qu X.Effect of long non-coding RNA highly up-regulated in liver cancer (HULC) on theprognosis of cancer: a meta-analysis. Oncotarget. 2017 Jun 13;8(47):83246-83250.doi: 10.18632/oncotarget.18452. PMID: 29137338; PMCID: PMC5669964.  632: Taheri M, Shoorei H, Tondro Anamag F, Ghafouri-Fard S, Dinger ME. LncRNAsand miRNAs participate in determination of sensitivity of cancer cells tocisplatin. Exp Mol Pathol. 2021 Dec;123:104602. doi:10.1016/j.yexmp.2021.104602. Epub 2021 Jan 8. PMID: 33422487.  633: Kobayashi E, Hornicek FJ, Duan Z. MicroRNA Involvement in Osteosarcoma.Sarcoma. 2012;2012:359739. doi: 10.1155/2012/359739. Epub 2012 Apr 3. PMID:22550419; PMCID: PMC3329862.  634: Ozaki T, Yu M, Yin D, Sun D, Zhu Y, Bu Y, Sang M. Impact of RUNX2 on drug-resistant human pancreatic cancer cells with p53 mutations. BMC Cancer. 2018 Mar20;18(1):309. doi: 10.1186/s12885-018-4217-9. PMID: 29558908; PMCID: PMC5861661.  635: Coomans de Brachène A, Demoulin JB. FOXO transcription factors in cancerdevelopment and therapy. Cell Mol Life Sci. 2016 Mar;73(6):1159-72. doi:10.1007/s00018-015-2112-y. Epub 2015 Dec 19. PMID: 26686861.  636: Hassanein SS, Ibrahim SA, Abdel-Mawgood AL. Cell Behavior of Non-Small CellLung Cancer Is at EGFR and MicroRNAs Hands. Int J Mol Sci. 2021 Nov19;22(22):12496. doi: 10.3390/ijms222212496. PMID: 34830377; PMCID: PMC8621388.  637: Zhang XB, Zhang RH, Su X, Qi J, Hu YC, Shi JT, Zhang K, Wang KP, Zhou HY.Exosomes in osteosarcoma research and preclinical practice. Am J Transl Res.2021 Mar 15;13(3):882-897. PMID: 33841628; PMCID: PMC8014357.  638: Ferretti VA, León IE. Long Non-coding RNAs in Cisplatin Resistance inOsteosarcoma. Curr Treat Options Oncol. 2021 Mar 20;22(5):41. doi:10.1007/s11864-021-00839-y. PMID: 33745006.  639: Menéndez ST, Gallego B, Murillo D, Rodríguez A, Rodríguez R. Cancer StemCells as a Source of Drug Resistance in Bone Sarcomas. J Clin Med. 2021 Jun14;10(12):2621. doi: 10.3390/jcm10122621. PMID: 34198693; PMCID: PMC8232081.  640: Agrawal S, Kandimalla ER. Role of Toll-like receptors in antisense and siRNA[corrected]. Nat Biotechnol. 2004 Dec;22(12):1533-7. doi: 10.1038/nbt1042.Erratum in: Nat Biotechnol. 2005 Jan;23(1):117. PMID: 15583662.  641: Liu J, Yang L, Fu Q, Liu S. Emerging Roles and Potential Biological Value ofCircRNA in Osteosarcoma. Front Oncol. 2020 Oct 28;10:552236. doi:10.3389/fonc.2020.552236. PMID: 33251132; PMCID: PMC7673402.  642: Melim C, Jarak I, Veiga F, Figueiras A. The potential of micelleplexes as atherapeutic strategy for osteosarcoma disease. 3 Biotech. 2020 Apr;10(4):147.doi: 10.1007/s13205-020-2142-5. Epub 2020 Mar 2. PMID: 32181109; PMCID:PMC7052088.  643: Yu X, Yustein JT, Xu J. Research models and mesenchymal/epithelialplasticity of osteosarcoma. Cell Biosci. 2021 May 22;11(1):94. doi:10.1186/s13578-021-00600-w. PMID: 34022967; PMCID: PMC8141200.  644: Llobat L, Gourbault O. Role of MicroRNAs in Human Osteosarcoma: FuturePerspectives. Biomedicines. 2021 Apr 23;9(5):463. doi:10.3390/biomedicines9050463. PMID: 33922820; PMCID: PMC8146779.  645: Bottani M, Banfi G, Lombardi G. Circulating miRNAs as Diagnostic andPrognostic Biomarkers in Common Solid Tumors: Focus on Lung, Breast, ProstateCancers, and Osteosarcoma. J Clin Med. 2019 Oct 11;8(10):1661. doi:10.3390/jcm8101661. PMID: 31614612; PMCID: PMC6833074.  646: Chai X, Yinwang E, Wang Z, Wang Z, Xue Y, Li B, Zhou H, Zhang W, Wang S,Zhang Y, Li H, Mou H, Sun L, Qu H, Wang F, Zhang Z, Chen T, Ye Z. Predictive andPrognostic Biomarkers for Lung Cancer Bone Metastasis and Their TherapeuticValue. Front Oncol. 2021 Oct 14;11:692788. doi: 10.3389/fonc.2021.692788. PMID:34722241; PMCID: PMC8552022.  647: Liu H, Li P, Chen L, Jian C, Li Z, Yu A. MicroRNAs as a novel class ofdiagnostic biomarkers for the detection of osteosarcoma: a meta-analysis. OncoTargets Ther. 2017 Nov 1;10:5229-5236. doi: 10.2147/OTT.S143974. PMID: 29138575;PMCID: PMC5677380.  648: He J, Ling L, Liu Z, Ren X, Wan L, Tu C, Li Z. Functional interplay betweenlong non-coding RNAs and the Wnt signaling cascade in osteosarcoma. Cancer CellInt. 2021 Jun 15;21(1):313. doi: 10.1186/s12935-021-02013-8. PMID: 34130697;PMCID: PMC8207720.  649: In: Sergi CM, editor. Metastasis. Brisbane (AU)2022.  650: Ahmed EA, Rajendran P, Scherthan H. The microRNA-202 as a Diagnostic Biomarker and a Potential Tumor Suppressor. Int J Mol Sci. 2022 May 24. 23(11). doi: 10.3390/ijms23115870. PMID: 35682549; PMCID: PMC9180238.  651: Aryee DNT, Fock V, Kapoor U, Radic-Sarikas B, Kovar H. Zooming in on Long Non-Coding RNAs in Ewing Sarcoma Pathogenesis. Cells. 2022 Apr 8. 11(8). doi: 10.3390/cells11081267. PMID: 35455947; PMCID: PMC9032025.  652: Dong Z, Liao Z, He Y, Wu C, Meng Z, Qin B, Xu G, Li Z, Sun T, Wen Y, Li G. Advances in the Biological Functions and Mechanisms of miRNAs in the Development of Osteosarcoma. Technol Cancer Res Treat. 2022 Jan-Dec. 21:15330338221117386. doi: 10.1177/15330338221117386. PMID: 35950243; PMCID: PMC9379803.  653: Farzaneh M, Najafi S, Anbiyaee O, Azizidoost S, Khoshnam SE. LncRNA MALAT1-related signaling pathways in osteosarcoma. Clin Transl Oncol. 2022 Jul 6. doi: 10.1007/s12094-022-02876-x. PMID: 35790599.  654: Zhong C, Dong Y, Zhang Q, Yuan C, Duan S. Aberrant Expression of miR-1301 in Human Cancer. Front Oncol. 2021. 11:789626. doi: 10.3389/fonc.2021.789626. PMID: 35070996; PMCID: PMC8767067.  655: Lakiotaki E, Kanakoglou DS, Pampalou A, Karatrasoglou EA, Piperi C, Korkolopoulou P. Dissecting the Role of Circular RNAs in Sarcomas with Emphasis on Osteosarcomas. Biomedicines. 2021 Nov 8. 9(11). doi: 10.3390/biomedicines9111642. PMID: 34829872; PMCID: PMC8615931  656: Li J, Guo S, Sun Z, Fu Y. Noncoding RNAs in Drug Resistance of Gastrointestinal Stromal Tumor. Front Cell Dev Biol. 2022. 10:808591. doi: 10.3389/fcell.2022.808591. PMID: 35174150; PMCID: PMC8841737.  657: Liu J, Shang G. The Roles of Noncoding RNAs in the Development of Osteosarcoma Stem Cells and Potential Therapeutic Targets. Front Cell Dev Biol. 2022. 10:773038. doi: 10.3389/fcell.2022.773038. PMID: 35252166; PMCID: PMC8888953.  658: Mardekian SK, Bombonati A, Palazzo JP. Ductal carcinoma in situ of the breast: the importance of morphologic and molecular interactions. Hum Pathol. 2016 Mar. 49:114-23. doi: 10.1016/j.humpath.2015.11.003. PMID: 26826418.  659: Odri GA, Tchicaya-Bouanga J, Yoon DJY, Modrowski D. Metastatic Progression of Osteosarcomas: A Review of Current Knowledge of Environmental versus Oncogenic Drivers. Cancers (Basel). 2022 Jan 12. 14(2). doi: 10.3390/cancers14020360. PMID: 35053522; PMCID: PMC8774233.  660: Paskeh MDA, Mehrabi A, Gholami MH, Zabolian A, Ranjbar E, Saleki H, Ranjbar A, Hashemi M, Ertas YN, Hushmandi K, Mirzaei S, Ashrafizadeh M, Zarrabi A, Samarghandian S. EZH2 as a new therapeutic target in brain tumors: Molecular landscape, therapeutic targeting and future prospects. Biomed Pharmacother. 2022 Feb. 146:112532. doi: 10.1016/j.biopha.2021.112532. PMID: 34906772.  661: Peng Z, Liu C, Wu M. New insights into long noncoding RNAs and their roles in glioma. Mol Cancer. 2018 Feb 19. 17(1):61. doi: 10.1186/s12943-018-0812-2. PMID: 29458374; PMCID: PMC5817731.  662: Pfister SM, Korshunov A, Kool M, Hasselblatt M, Eberhart C, Taylor MD. Molecular diagnostics of CNS embryonal tumors. Acta Neuropathol. 2010 Nov. 120(5):553-66. doi: 10.1007/s00401-010-0751-5. PMID: 20882288; PMCID: PMC4512653.  663: Ren DY, Yuan XR, Tu CX, Shen JL, Li YW, Yan AH, Ru Y, Han HY, Yang YM, Liu Y, Li HY. Long Noncoding RNA 00472: A Novel Biomarker in Human Diseases. Front Pharmacol. 2021. 12:726908. doi: 10.3389/fphar.2021.726908. PMID: 34987381; PMCID: PMC8722734.  664: Soghli N, Ferns GA, Sadeghsoltani F, Qujeq D, Yousefi T, Vaghari-Tabari M. MicroRNAs and osteosarcoma: Potential targets for inhibiting metastasis and increasing chemosensitivity. Biochem Pharmacol. 2022 Jul. 201:115094. doi: 10.1016/j.bcp.2022.115094. PMID: 35588853.  665: Song XJ, Bi MC, Zhu QS, Liu XL. The emerging role of lncRNAs in the regulation of osteosarcoma stem cells. Eur Rev Med Pharmacol Sci. 2022 Feb. 26(3):966-74. doi: 10.26355/eurrev_202202_28006. PMID: 35179763.  666: Tang J, He J, Feng C, Tu C. Exosomal MiRNAs in Osteosarcoma: Biogenesis and Biological Functions. Front Pharmacol. 2022. 13:902049. doi: 10.3389/fphar.2022.902049. PMID: 35592419; PMCID: PMC9110813.  667: Tu K, Lee S, Roy S, Sawant A, Shukla H. Dysregulated Epigenetics of Chordoma: Prognostic Markers and Therapeutic Targets. Curr Cancer Drug Targets. 2022 Aug 15. 22(8):678-90. doi: 10.2174/1568009622666220419122716. PMID: 35440334.  668: Wen J, Li H, Li D, Dong X. Clinicopathological and prognostic significance of long non-coding RNA EWSAT1 in human cancers: A review and meta analysis. PLoS One. 2022. 17(3):e0265264. doi: 10.1371/journal.pone.0265264. PMID: 35286362; PMCID: PMC8920262.  669: Yao P, Lu Y, Cai Z, Yu T, Kang Y, Zhang Y, Wang X. Research Progress of Exosome-Loaded miRNA in Osteosarcoma. Cancer Control. 2022 Jan-Dec. 29:10732748221076683. doi: 10.1177/10732748221076683. PMID: 35179996; PMCID: PMC8859673.  670: Tu C, Yang K, Wan L, He J, Qi L, Wang W, Lu Q, Li Z. The crosstalk betweenlncRNAs and the Hippo signalling pathway in cancer progression. Cell Prolif.2020 Sep;53(9):e12887. doi: 10.1111/cpr.12887. Epub 2020 Aug 10. PMID: 32779318;PMCID: PMC7507458.  671: Meng Y, Liu YL, Li K, Fu T. Prognostic value of long non-coding RNA breastcancer anti-estrogen resistance 4 in human cancers: A meta-analysis. Medicine(Baltimore). 2019 May;98(21):e15793. doi: 10.1097/MD.0000000000015793. PMID:31124974; PMCID: PMC6571273.  672: Jamali Z, Taheri-Anganeh M, Shabaninejad Z, Keshavarzi A, Taghizadeh H,Razavi ZS, Mottaghi R, Abolhassan M, Movahedpour A, Mirzaei H. Autophagyregulation by microRNAs: Novel insights into osteosarcoma therapy. IUBMB Life.2020 Jul;72(7):1306-1321. doi: 10.1002/iub.2277. Epub 2020 Mar 31. PMID:32233112.  673: Ravegnini G, Sammarini G, Nannini M, Pantaleo MA, Biasco G, Hrelia P,Angelini S. Gastrointestinal stromal tumors (GIST): Facing cell death betweenautophagy and apoptosis. Autophagy. 2017 Mar 4;13(3):452-463. doi:10.1080/15548627.2016.1256522. Epub 2017 Jan 5. PMID: 28055310; PMCID:PMC5361605.  674: Subramanian S, Kartha RV. MicroRNA-mediated gene regulations in humansarcomas. Cell Mol Life Sci. 2012 Nov;69(21):3571-85. doi:10.1007/s00018-012-1127-x. Epub 2012 Aug 25. PMID: 22922987.  675: Han C, Shen JK, Hornicek FJ, Kan Q, Duan Z. Regulation of microRNA-1 (miR-1)expression in human cancer. Biochim Biophys Acta Gene Regul Mech. 2017Feb;1860(2):227-232. doi: 10.1016/j.bbagrm.2016.12.004. Epub 2016 Dec 5. PMID:27923712.  676: Nannini M, Ravegnini G, Angelini S, Astolfi A, Biasco G, Pantaleo MA. miRNAprofiling in gastrointestinal stromal tumors: implication as diagnostic andprognostic markers. Epigenomics. 2015;7(6):1033-49. doi: 10.2217/epi.15.52. Epub2015 Oct 8. PMID: 26447534.  677: Kim WK, Yang HK, Kim H. MicroRNA involvement in gastrointestinal stromaltumor tumorigenesis. Curr Pharm Des. 2013;19(7):1227-35. doi:10.2174/138161213804805748. PMID: 23092343.  678: Wang C, Jing J, Cheng L. Emerging roles of non-coding RNAs in thepathogenesis, diagnosis and prognosis of osteosarcoma. Invest New Drugs. 2018Dec;36(6):1116-1132. doi: 10.1007/s10637-018-0624-7. Epub 2018 Aug 6. PMID:30079443.  679: Ren F, Shen J, Shi H, Hornicek FJ, Kan Q, Duan Z. Novel mechanisms andapproaches to overcome multidrug resistance in the treatment of ovarian cancer.Biochim Biophys Acta. 2016 Dec;1866(2):266-275. doi:10.1016/j.bbcan.2016.10.001. Epub 2016 Oct 4. PMID: 27717733.  680: Zhao W, Wang Z, Fang X, Li N, Fang J. Long noncoding RNA Breast cancerantiestrogen resistance 4 is associated with cancer progression and itssignificant prognostic value. J Cell Physiol. 2019 Aug;234(8):12956-12963. doi:10.1002/jcp.27962. Epub 2018 Dec 7. PMID: 30537165.  681: Chang J, Yao M, Li Y, Zhao D, Hu S, Cui X, Liu G, Shi Q, Wang Y, Yang Y.MicroRNAs for osteosarcoma in the mouse: a meta-analysis. Oncotarget. 2016 Dec20;7(51):85650-85674. doi: 10.18632/oncotarget.13333. PMID: 27852052; PMCID:PMC5356766.  682: Tian S, Liu J, Kong S, Peng L. LncRNA DLX6-AS1 as a potential molecularbiomarker in the clinicopathology and prognosis of various cancers: a meta-analysis. Biosci Rep. 2020 Aug 28;40(8):BSR20193532. doi: 10.1042/BSR20193532.PMID: 32785606; PMCID: PMC7447853.  683: Han C, Yu Z, Duan Z, Kan Q. Role of microRNA-1 in human cancer and itstherapeutic potentials. Biomed Res Int. 2014;2014:428371. doi:10.1155/2014/428371. Epub 2014 May 18. PMID: 24949449; PMCID: PMC4052501.  684: Sun R, Shen JK, Choy E, Yu Z, Hornicek FJ, Duan Z. The emerging roles andtherapeutic potential of microRNAs (miRs) in liposarcoma. Discov Med. 2015Nov;20(111):311-24. PMID: 26645903.  685: Zhong Y, Zhao M, Yu Y, Li Q, Wang F, Wu P, Zhang W, Miao L. Prognostic valueand therapeutic potential of the long noncoding RNA TP73-AS1 in cancers: Asystematic review and meta-analysis. Sci Rep. 2020 Jun 3;10(1):9053. doi:10.1038/s41598-020-65726-2. PMID: 32493915; PMCID: PMC7271165.  686: Zhou W, Hao M, Du X, Chen K, Wang G, Yang J. Advances in targeted therapyfor osteosarcoma. Discov Med. 2014 Jun;17(96):301-7. PMID: 24979249.  687: Zhou G, Shi X, Zhang J, Wu S, Zhao J. MicroRNAs in osteosarcoma: frombiological players to clinical contributors, a review. J Int Med Res. 2013Feb;41(1):1-12. doi: 10.1177/0300060513475959. Epub 2013 Jan 24. PMID: 23569124.  688: Lin Z, Xie X, Lu S, Liu T. Noncoding RNAs in osteosarcoma: Implications fordrug resistance. Cancer Lett. 2021 Apr 28;504:91-103. doi:10.1016/j.canlet.2021.02.007. Epub 2021 Feb 12. PMID: 33587978.  689: Sinkovics JG. Molecular biology of oncogenic inflammatory processes. I. Non-oncogenic and oncogenic pathogens, intrinsic inflammatory reactions withoutpathogens, and microRNA/DNA interactions (Review). Int J Oncol. 2012Feb;40(2):305-49. doi: 10.3892/ijo.2011.1248. Epub 2011 Nov 4. PMID: 22076306.  690: Susa M, Milane L, Amiji MM, Hornicek FJ, Duan Z. Nanoparticles: a promisingmodality in the treatment of sarcomas. Pharm Res. 2011 Feb;28(2):260-72. doi:10.1007/s11095-010-0173-z. Epub 2010 May 27. PMID: 20505985.  691: Gasparini C, Vecchi Brumatti L, Monasta L, Zauli G. TRAIL-based therapeuticapproaches for the treatment of pediatric malignancies. Curr Med Chem.2013;20(17):2254-71. doi: 10.2174/0929867311320170009. PMID: 23458616.  692: Li S, Wang X. The potential roles of exosomal noncoding RNAs inosteosarcoma. J Cell Physiol. 2021 May;236(5):3354-3365. doi: 10.1002/jcp.30101.Epub 2020 Oct 12. PMID: 33044018.  693: Awan FM, Yang BB, Naz A, Hanif A, Ikram A, Obaid A, Malik A, Janjua HA, AliA, Sharif S. The emerging role and significance of circular RNAs in viralinfections and antiviral immune responses: possible implication as theranosticagents. RNA Biol. 2021 Jan;18(1):1-15. doi: 10.1080/15476286.2020.1790198. Epub2020 Jul 13. PMID: 32615049; PMCID: PMC7833768.  694: Palmini G, Brandi ML. microRNAs and bone tumours: Role of tiny molecules inthe development and progression of chondrosarcoma, of giant cell tumour of boneand of Ewing's sarcoma. Bone. 2021 Aug;149:115968. doi:10.1016/j.bone.2021.115968. Epub 2021 Apr 21. PMID: 33892177.  695: Peng Y, Huang D, Ma K, Deng X, Shao Z. MiR-19a as a prognostic indicator forcancer patients: a meta-analysis. Biosci Rep. 2019 May 14;39(5):BSR20182370.doi: 10.1042/BSR20182370. PMID: 31015372; PMCID: PMC6522715.  696: Wang Y, Wang Y, Qin Z, Cai S, Yu L, Hu H, Zeng S. The role of non-codingRNAs in ABC transporters regulation and their clinical implications of multidrugresistance in cancer. Expert Opin Drug Metab Toxicol. 2021 Mar;17(3):291-306.doi: 10.1080/17425255.2021.1887139. Epub 2021 Feb 22. PMID: 33544643.  697: Plaisance-Bonstaff K, Choi HS, Beals T, Krueger BJ, Boss IW, Gay LA, HaeckerI, Hu J, Renne R. KSHV miRNAs decrease expression of lytic genes in latentlyinfected PEL and endothelial cells by targeting host transcription factors.Viruses. 2014 Oct 23;6(10):4005-23. doi: 10.3390/v6104005. PMID: 25341664;PMCID: PMC4213575.  698: Wang X, Ning Y, Yang L, Liu H, Wu C, Wang S, Guo X. Diagnostic value ofcirculating microRNAs for osteosarcoma in Asian populations: a meta-analysis.Clin Exp Med. 2017 May;17(2):175-183. doi: 10.1007/s10238-016-0422-5. Epub 2016Apr 22. PMID: 27106278. |
| **Case report (N=1)** |
| 699: Ghisoli M, Rutledge M, Stephens PJ, Mennel R, Barve M, Manley M, Oliai BR,Murphy KM, Manning L, Gutierrez B, Rangadass P, Walker A, Wang Z, Rao D, AdamsN, Wallraven G, Senzer N, Nemunaitis J. Case Report: Immune-mediated CompleteResponse in a Patient With Recurrent Advanced Ewing Sarcoma (EWS) After VigilImmunotherapy. J Pediatr Hematol Oncol. 2017 May;39(4):e183-e186. doi:10.1097/MPH.0000000000000822. PMID: 28338569. |
| **Publications not in English (N = 4)** |
| 700: Xia Q, Ni J, Huang J, Pan B, Yan M, Li W. [Suppression ofmiR-30a/HMGA2-mediated autophagy in osteosarcoma cells impactschemotherapeutics-inducedapoptosis]. Zhong Nan Da Xue Xue Bao Yi Xue Ban. 2019Jul 28;44(7):757-766. Chinese. doi: 10.11817/j.issn.1672-7347.2019.180449. PMID:  31413213.  701: Zhu KP, Zhang CL. [Sensitivity of doxorubicin-resistant osteosarcoma cells todoxorubicin regulated by long non-coding RNA NR_036444]. Zhonghua Zhong Liu ZaZhi. 2017 Apr 23;39(4):250-255. Chinese. doi:10.3760/cma.j.issn.0253-3766.2017.04.003. PMID: 28550663.  702: Song Y. Mechanism of the Notch signaling pathway in enhancing the efficacy ofchemotherapy drugs in osteosarcoma. Zhong Nan Da Xue Xue Bao Yi Xue Ban. 2020Oct 28;45(10):1234-1240. English, Chinese. doi:10.11817/j.issn.1672-7347.2020.190147. PMID: 33268586.  703: Cao F, Kang XH, Wang DF, Ma L, Cao XJ, Wang Y, Gao YY, Miao ZH, Deng HB, GongYB. [Mechanism of lncRNA-SRLR induced invasion and metastasis in U2OSosteosarcoma cells]. Zhonghua Zhong Liu Za Zhi. 2020 Dec 23;42(12):1007-1013.Chinese. doi: 10.3760/cma.j.cn112152-20190404-00216. PMID: 33342156. |
| **No full-text available (N = 2)** |
| 704: Lu B, Feng Z, Fan B, Shi Y. Blocking miR-27a-3p sensitises Taxol resistantosteosarcoma cells through targeting Fbxw7. Bull Cancer. 2021Jun;108(6):596-604. doi: 10.1016/j.bulcan.2021.01.006. Epub 2021 Apr 15. PMID:33863546.  705: Liu Y, Liu X, Yang S. MicroRNA-221 Upregulates the Expression of P-gp andBcl-2 by Activating the Stat3 Pathway to Promote Doxorubicin Resistance inOsteosarcoma Cells. Biol Pharm Bull. 2021 Jun 1;44(6):861-868. doi:10.1248/bpb.b21-00163. Epub 2021 Apr 8. PMID: 33828027. |
| **Retracted (N=10)** |
| 706: Geng S, Gu L, Ju F, Zhang H, Wang Y, Tang H, Bi Z, Yang C. MicroRNA-224promotes the sensitivity of osteosarcoma cells to cisplatin by targeting Rac1. JCell Mol Med. 2016 Sep;20(9):1611-9. doi: 10.1111/jcmm.12852. Epub 2016 May 25.Retraction in: J Cell Mol Med. 2019 Aug;23(8):5832. PMID: 27222381; PMCID:PMC4884199.  707: Kong D, Ying B, Zhang J, Ying H. The anti-osteosarcoma property ofailanthone through regulation of miR-126/VEGF-A axis. Artif Cells NanomedBiotechnol. 2019 Dec;47(1):3913-3919. doi: 10.1080/21691401.2019.1669622.Retraction in: Artif Cells Nanomed Biotechnol. 2020 Dec;48(1):1254. PMID:31571500.  708: Wu BQ, Cao Y, Bi ZG. Suppression of adriamycin resistance in osteosarcoma byblocking Wnt/β-catenin signal pathway. Eur Rev Med Pharmacol Sci. 2017Jul;21(14):3185-3192. Retraction in: Eur Rev Med Pharmacol Sci. 2020Jul;24(14):7548. PMID: 28770967.  709: Qu Y, Xia P, Zhang S, Pan S, Zhao J. Silencing XIAP suppresses osteosarcomacell growth, and enhances the sensitivity of osteosarcoma cells to doxorubicinand cisplatin. Oncol Rep. 2015 Mar;33(3):1177-84. doi: 10.3892/or.2014.3698.Epub 2014 Dec 23. Retraction in: Oncol Rep. 2022 Jan;47(1): PMID: 25572427.  710: He QY, Wang GC, Zhang H, Tong DK, Ding C, Liu K, Ji F, Zhu X, Yang S.miR-106a-5p Suppresses the Proliferation, Migration, and Invasion ofOsteosarcoma Cells by Targeting HMGA2. DNA Cell Biol. 2016 Sep;35(9):506-20.doi: 10.1089/dna.2015.3121. Epub 2016 Jul 6. Retraction in: DNA Cell Biol. 2020Oct;39(10):1912. PMID: 27383537.  711: Taheriazam A, Talaei AJ, Jamshidi M, Shakeri M, Khoshbakht S, Yahaghi E,Shokrani M. Up-regulation of miR-130b expression level and down-regulation ofmiR-218 serve as potential biomarker in the early detection of humanosteosarcoma. Diagn Pathol. 2015 Oct 7;10:184. doi: 10.1186/s13000-015-0422-x.Retraction in: Diagn Pathol. 2016 Nov 2;11(1):112. PMID: 26446495; PMCID:PMC4596511.  712: Guasparri I, Keller SA, Cesarman E. KSHV vFLIP is essential for thesurvival of infected lymphoma cells. J Exp Med. 2004 Apr 5;199(7):993-1003. doi:10.1084/jem.20031467. Retraction in: J Exp Med. 2021 Mar 1;218(3): Erratum in: JExp Med. 2006 May 15;203(5):1383. PMID: 15067035; PMCID: PMC2211879.  713: Yu Z, Li N, Jiang K, Zhang N, Yao LL. MiR-100 up-regulation enhanced cell autophagy and apoptosis induced by cisplatin in osteosarcoma by targeting mTOR. Eur Rev Med Pharmacol Sci. 2018 Sep;22(18):5867-5873. doi: 10.26355/eurrev_201809_15913. Retraction in: Eur Rev Med Pharmacol Sci. 2020 Jul;24(14):7570. PMID: 30280766.  714:Wang L, Tang B, Han H, Mao D, Chen J, Zeng Y, Xiong M. miR-155 Affects Osteosarcoma MG-63 Cell Autophagy Induced by Adriamycin Through Regulating PTEN-PI3K/AKT/mTOR Signaling Pathway. Cancer Biother Radiopharm. 2018 Feb;33(1):32-38. doi: 10.1089/cbr.2017.2306. Epub 2018 Feb 7. Retraction in: Cancer Biother Radiopharm. 2021 Sep;36(7):614. PMID: 29412697.  715: 1: Qi X, Yu XJ, Wang XM, Song TN, Zhang J, Guo XZ, Li GJ, Shao M. Knockdown of KCNQ1OT1 Suppresses Cell Invasion and Sensitizes Osteosarcoma Cells to CDDP by Upregulating DNMT1-Mediated Kcnq1 Expression. Mol Ther Nucleic Acids. 2019 Sep 6;17:804-818. doi: 10.1016/j.omtn.2019.06.010. Epub 2019 Jun 27. Retraction in: Mol Ther Nucleic Acids. 2022 May 10;28:589. PMID: 31454677; PMCID: PMC6716066. |
